# Microsporidian infections in the species complex *Gammarus roeselii* (Amphipoda) over its geographical range: evidence for both host–parasite co-diversification and recent host shifts

**DOI:** 10.1186/s13071-019-3571-z

**Published:** 2019-06-28

**Authors:** Adrien Quiles, Karolina Bacela-Spychalska, Maria Teixeira, Nicolas Lambin, Michal Grabowski, Thierry Rigaud, Rémi André Wattier

**Affiliations:** 1 Université Bourgogne Franche-Comté, Laboratoire Biogéosciences, UMR CNRS 6282, 6 Boulevard Gabriel, 21000 Dijon, France; 2 Department of Invertebrate Zoology and Hydrobiology, University of Lodz, 12/16 Banacha Street, 90-237, Lodz, Poland

**Keywords:** Host–parasite interactions, Biogeography, Phylogeography, Microsporidia, Amphipods

## Abstract

**Background:**

Microsporidians are obligate endoparasites infecting taxonomically diverse hosts. Both vertical (from mother to eggs) and horizontal (between conspecifics or between species) transmission routes are known. While the former may promote co-speciation and host-specificity, the latter may promote shifts between host species. Among aquatic arthropods, freshwater amphipod crustaceans are hosts for many microsporidian species. However, despite numerous studies, no general pattern emerged about host specificity and co-diversification. In south-eastern Europe, the gammarid *Gammarus roeselii* is composed of 13 cryptic lineages of Miocene to Pleistocene age but few genotypes of one lineage have spread postglacially throughout north-western Europe. Based on nearly 100 sampling sites covering its entire range, we aim to: (i) explore the microsporidian diversity present in *G. roeselii* and their phylogenetic relationships, especially in relation to the parasites infecting other Gammaridae; (ii) test if the host phylogeographical history might have impacted host–parasite association (e.g. co-diversifications or recent host shifts from local fauna).

**Methods:**

We used part of the small subunit rRNA gene as source of sequences to identify and determine the phylogenetic position of the microsporidian taxa infecting *G. roeselii*.

**Results:**

Microsporidian diversity was high in *G. roeselii* with 24 detected haplogroups, clustered into 18 species-level taxa. Ten microsporidian species were rare, infecting a few individual hosts in a few populations, and were mostly phylogenetically related to parasites from other amphipods or various crustaceans. Other microsporidians were represented by widespread genera with high prevalence: *Nosema*, *Cucumispora* and *Dictyocoela.* Two contrasting host association patterns could be observed. First, two vertically transmitted microsporidian species, *Nosema granulosis* and *Dictyocoela roeselum*, share the pattern of infecting *G. roeselii* over most of its range and are specific to this host suggesting the co-diversification scenario. This pattern contrasted with that of *Dictyocoela muelleri*, the three species of *Cucumispora*, and the rare parasites, present only in the recently colonised region by the host. These patterns suggest recent acquisitions from local host species, after the recent spread of *G. roeselii*.

**Conclusions:**

Microsporidians infecting *G. roeselii* revealed two scenarios of host–parasite associations: (i) ancient associations with vertically transmitted parasites that probably co-diversified with their hosts, and (ii) host shifts from local host species, after the postglacial spread of *G. roeselii*.

**Electronic supplementary material:**

The online version of this article (10.1186/s13071-019-3571-z) contains supplementary material, which is available to authorized users.

## Background

Microsporidians are obligate endoparasites infecting taxonomically diverse hosts inhabiting various environments [1]. These unicellular eukaryotes form a very old and phylogenetically highly diverse phylum, with more than 1300 species in 160 genera [2]. They exhibit different transmission and host exploitation strategies, such as horizontal transmission (HT) often linked to high virulence, or vertical transmission (VT), often associated with low or presumably no virulence, or a combination of both VT and HT [3, 4]. Microsporidians are particularly frequent in aquatic ecosystems [5]. Among the aquatic arthropods, freshwater amphipod crustaceans, and especially species of the Gammaridae, are regular hosts for microsporidians (for overviews see [6, 7]). Since the early descriptions of microsporidians in amphipods (e.g. [6, 8]) there is a constantly increasing number of full descriptions of new genera and species, combining ultrastructural and molecular phylogenetic support [9–11]. Species of three major microsporidian genera (*Nosema* [12], *Cucumispora* [9] and *Dictyocoela* [6]) are infecting freshwater amphipods. They infect many host species across Europe and are highly prevalent. *Nosema granulosis* is so far the only species of its genus known to infect gammarids. This parasite is transovarially-transmitted, feminises host offspring and in consequence induces excess of females in the infected populations [6, 13–15]. This parasite causes limited pathology and shows little evidence of horizontal transmission [3, 8, 15, 16]. *Nosema granulosis* was found to infect other gammarid species such as *Dikerogammarus villosus* [17], *Gammarus fossarum* [16, 18], *Gammarus roeselii* [19] and *Niphargus schellenbergi* [16]. The second frequent genus, *Cucumispora* [9], was found to date only in gammarids. Three species have been described: *C. dikerogammari* infecting *Dikerogammarus villosus* [9], *C. ornata* infecting *Dikerogammarus haemobaphes* [10] and *C. roeselii* infecting *Gammarus roeselii* [20]. These parasites are infecting mostly muscles and show high rates of horizontal trophic transmission [6, 9, 21]. *Cucumispora dikerogammari* was also observed to manipulate predatory behaviour of their hosts [22, 23]. Finally, *Dictyocoela* was found to be the dominant microsporidian genus infecting freshwater amphipods [6]. Its phylogeny was recently reassessed using an integrative approach (molecular and ultrastructural traits, see [24]). On one hand, four species were fully described: *Dictyocoela duebenum*, *D. muelleri*, *D. berillonum* and *D. roeselum.* On the other hand, at least four other species belonging to this genus await formal description, as only ribosomal sequences are available to date [24]. *Dictyocoela* spp. are usually described as vertically-transmitted [6, 8], inducing sex ratio distortion by feminising males, similar to that induced by *N. granulosis*. They have been found, often at high prevalence, in numerous *Gammarus* spp. (*Gammarus aequicauda* [24], *G. balcanicus* [24], *G. duebeni* [25], *G. fossarum* [25, 26], *G. lacustris* [25], *G. pulex* [20, 25, 27], *G. roeselii* [6, 19, 24], *G. setosus* [24, 25], *G. varsoviensis* [24]) and in 15 other amphipod species [6, 17, 20, 24, 25, 28, 29]. In addition to these three major genera, a dozen of additional microsporidian lineages have been identified based on molecular divergence. They were not fully described (i.e. lacking anatomical and ultrastructural descriptions) and, in most cases, they were only observed sporadically [5, 6, 18, 25, 30, 31].

The nature of the studies investigating microsporidian infections in European gammarids is diverse. For example, *G. duebeni* and *D. villosus* host populations were investigated at both small [9, 32] and large geographical scales [17, 18]. However, other studies focusing on single hosts were more limited geographically, restricted to one or a few populations. This was the case for *Gammarus roeselii* [19, 33] or *D. haemobaphes* [21]. Other studies were targeting one parasite in several host species such as *Dictyocoela* spp. [24, 25] or *Pleistophora muelleri* [30]. Finally, recent studies explored host and microsporidian assemblages at local geographical scales, such as part of the Ruhr drainage [5] or in Lake Baikal [28, 34, 35]. From all these studies, no clear specificity pattern emerged. In restrained geographical areas, a single host species may be infected by numerous parasite species (e.g. [5, 34]). Conversely, parasite species of a single genus may infect several host species (e.g. [25]). Furthermore, invasive amphipod species may introduce novel pathogens to a colonised area [21], which may promote parasite adaptation to novel hosts and, therefore, emergence of a new disease [36]. These patterns are sometimes obscured by poor resolution in parasite identification: it is often difficult to assert if microsporidians infecting several hosts belong to single or several “species” [5]. An additional level of complexity comes from many recent studies pointing out that most widespread gammarid species are in fact species complexes characterised by high cryptic diversity. Indeed, individuals ascribed to a single ‘species’ based on shared diagnostic morphological features may belong to highly divergent phylogenetic lineages (e.g. [37–44]). In most studies upon microsporidia-amphipod relationships, this cryptic diversity was not taken into account. For example, it remains unknown if a single microsporidian species is specific to one of the cryptic host species or can infect the whole species complex. Indeed, in gammarids, the cryptic diversity of the host seems not cryptic at all for their parasites, e.g. acanthocephalans [45, 46].

**Fig. 1:**
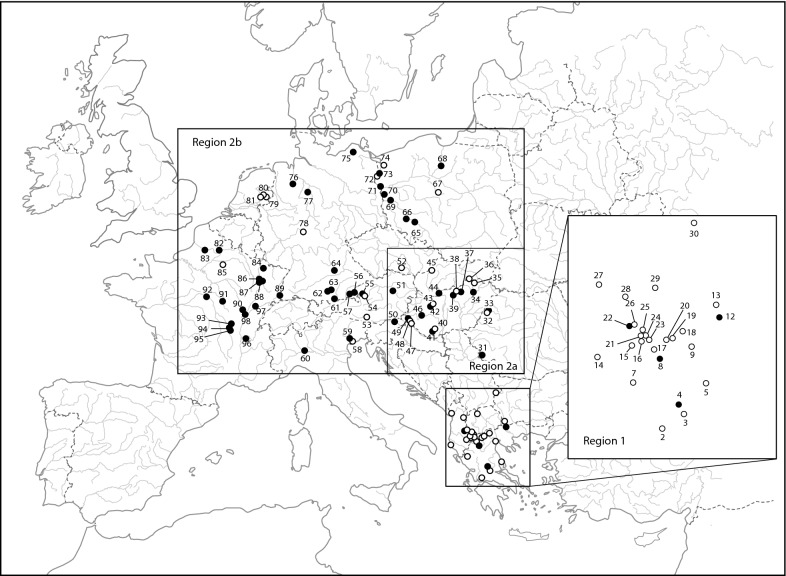
*Gammarus roeselii* sampling sites in this study. Empty dots refer to sites with no microsporidian infections. Black dots refer to sites where at least one infection was found in a *G. roeselii* individual and for which a sequence was obtained. Site labels refer to Additional file 1: Table S1. The enlarged geographical zone corresponds to area of ancient diversification of *G. roeselii* (thereafter named Region 1). Other sites are located either in the geographical zone of secondary diversification of *G. roeselii* (Region 2a) or in the area of recent, post-glacial, expansion (Region 2b)

The present study aimed to investigate the microsporidian infection patterns in a host with high cryptic diversity. We used *Gammarus roeselii* as a biological model because its biogeography and diversification patterns have recently been investigated [41, 47]. *Gammarus roeselii* populations are widely distributed across European freshwater ecosystems, but this morphospecies is characterised by extensive cryptic diversity with at least 13 highly divergent phylogenetic lineages (molecular operational taxonomic units, MOTUs, named A-M) [41]. These MOTUs diversified mostly over Miocene (starting *c.*18 Mya) in south-east Europe, predominantly in the Balkan Peninsula [41]. We define this area as primary diversification region (Region 1 in Fig. 1). However, one of these MOTUs (MOTU C) diversified further during Pleistocene in the Pannonian basin, north from the Balkans. We define it as secondary diversification area (Region 2a, Fig. 1). One of the lineages of MOTU C expanded postglacially its geographical range in northern and western Europe (Region 2b, Fig. 1), probably by a natural range expansion facilitated occasionally by human activities [47]. Some *G. roeselii* populations were shown to harbour microsporidian infections belonging to species of the three main genera infecting amphipods: *Nosema*, *Dictyocoela* and *Cucumispora* [5, 19, 20, 33]. These studies were conducted only in the area recently colonised by *G. roeselii* (France, Germany and Poland), thus providing a limited overview of the microsporidian assemblage associated with this host. In addition, the extent of infections in the host cryptic lineages remains unknown, with only *G. roeselii* MOTU C being studied so far. In these studies, *Nosema granulosis*, *Dictyocoela roeselum* and *D. muelleri* showed vertical transmission, and induced sex-ratio bias in host populations [19, 48], while the muscle-infecting *Cucumispora roeselum* was shown to be pathogenic, suggesting horizontal transmission [20].

The main aim of our study was to characterise extensively the diversity of microsporidians infecting *G. roeselii* over its entire geographical range and across the highly divergent host MOTUs. This will allow addressing the following issues: (i) clarify the infection pattern and specificity, by exploring the phylogenetic relationships of the detected microsporidia, and comparing these parasites with those of other Gammaridae; and (ii) disentangle the ecological or evolutionary scenarios leading to the observed infection pattern. The following questions were explored: (i) Are all parasite clades present in the host diversification zone in south-east Europe and do these clades show a diversification pattern matching that of the host (suggesting possibilities of host–parasite co-diversifications)? We predict that vertically-transmitted *Nosema* spp. and *Dictyocoela* spp., intimately linked to their hosts, correspond to such a pattern; (ii) Are some parasites restricted to the recently colonised region, and are they taxonomically identical to microsprodian infections found in local gammarids (suggesting possibilities for recent host shifts from local fauna in a given zone)? We predict that most horizontally-transmitted parasites may show such a pattern.

## Methods

### Sampling

*Gammarus roeselii* individuals were collected using hand nets and kick-sampling method, at 94 sites in 19 countries, during several sampling campaigns between 2004 and 2016, covering the area of western, central and south-eastern Europe; *c.*4 million km^2^ (Additional file 1: Table S1). Sites were plotted on a map (Fig. 1) using Qgis 2.18.4 [49]. The Balkans, hereafter referred to Region 1 (Fig. 1, 26 sites sampled), is known as an area of ancient (mostly Miocene) diversification of the host [41]. *Gammarus roeselii* secondarily (in Pleistocene) diversified in the Pannonian Plain, north from the Balkans [47], hereafter referred to as Region 2a (Fig. 1, 22 sites sampled). The rest of the distribution of *G. roeselii*, hereafter referred to as Region 2b (Fig. 1, 46 sites sampled), is the area colonised postglacially as a result of natural and anthropogenic processes [47]. Individuals were fixed in 96% ethanol directly in the field, and stored at room temperature after returning to the laboratory. Amphipods were identified to the species level using morphological characters described in available keys (e.g. [50, 51]). All the material was stored at the Department of Invertebrate Zoology and Hydrobiology, University of Lodz, Poland, and the laboratory of Biogeosciences, University Bourgogne Franche-Comté, Dijon, France.

### Host dissection and total DNA extraction

Dissection of each gammarid was performed under stereomicroscope taking *c.*2 mm^3^ of animal tissue (including muscles and gonads), from the thoracic segments 6 and 7. As microsporidia are intracellular parasites, their DNA was co-extracted with host DNA. Among the 1904 individuals used in the present study, DNA of 1108 individuals was already extracted as within the study by Grabowski et al. [41, 47] and 796 were newly extracted. While the sex of host was not recorded at the time of dissection for already extracted samples, it was determined for the newly extracted individuals for 10 sites, mainly from Region 1, we newly extracted DNA for up to 24 males and 24 females in addition to the initial set of DNA samples (Additional file 1: Table S1). Finally, *G. roeselii* individuals from additional sites (relative to Grabowski et al. [41, 47]) were dissected and newly extracted, for Region 1 and 2b. Altogether, Region 1, 2a and 2b accounted, respectively, for 931, 327 and 646 individuals. DNA extraction was performed using either (i) standard phenol-chloroform protocol or (ii) Biobasic EZ-10 96 Well Plate Animal Genomic DNA Isolation Kit and eluted in 100 µl of TE (pH 8). The DNA samples were kept at 4 °C until amplification and subsequently at −20 °C for long-term storage.

### Molecular screening for microsporidians

All 1904 individuals were screened for the presence of microsporidians using a short (*c.*350 bp long) diagnostic fragment of the small ribosomal subunit (*SSU* rDNA) marker. The microsporidia-specific primer V1f (forward) (5′-CAC CAG GTT GAT TCT GCC TGA C-3′) [52] paired with newly designed UNIr (reverse) (5′-TCA GGC TCC CTC TCC GGA AT-3′) was used. The use of this short fragment maximised the ability to detect the presence of microsporidians even in case of low infection intensity. As negative and positive controls in PCR reactions, we used, respectively, water and microsporidian DNA (*Dictyocoela roeselum*). The PCR program consisted of an initial denaturing phase at 95 °C for 2 min, followed by 35 cycles of 95 °C for 20 s, 57 °C for 20 s and 72 °C for 20 s, and a final extension at 72 °C for 5 min. The PCR products were visualised by electrophoresis after 20 min migration under 100V in 2% agarose gel.

### Sequencing of the microsporidian *SSU* rDNA

For all individuals positively diagnosed for microsporidian infection by PCR screening (see above) our target was to sequence the *c*.800 bp long fragment matching the 5′ part of the *SSU* rRNA gene. This target was tentatively achieved following two strategies: one based on a single amplicon using V1f as the forward primer and HG4r (5′-GCG GCT TAA TTT GAC TCAA C-3′) as the reverse primer (amplicon of *c.*850 bp) and one based on two amplicons with overlapping region i.e. V1F as the forward primer and 530r (5′-CCG CGG CTG CTG GCA C-3′) as the reverse primer; in addition to MC2F as the forward primer (5′-TCC GGA GAG GGA GCC TGA GAG A-3′) and 964r as the reverse primer (5′-CGC GTT GAG TCA AAT TAA GCC GCA CA-3′). When the 800 bp target was not reachable, either a V1f-530r fragment (*c*.530 bp long) or even a V1f-UNIr fragment (*c*.350 bp long) was used. Although the V1f-UNIr fragment is short, it contained enough phylogenetic information to ascribe sequences to, at least, the species level (see “Results”, Additional file 2: Table S2 and Additional file 3: Data S1). PCR products were purified and sequenced directly with the BigDye technology by Genewiz, Inc., UK, using the forward primers used in the PCR. Using Geneious 10.2.2 [53], raw sequences were edited, trimmed and checked for being microsporidian sequences *via* BlastN [54] search against the sequences available on GenBank.

### Phylogeny reconstruction for microsporidians

Four types of microsporidian sequences constituted our dataset: (i) sequences newly produced from our collection of infected *G. roeselii* individuals; (ii) published *SSU* sequences representing diversity and divergence of microsporidians already found to infect European freshwater or brackish water amphipods (we did not include sequences outside Europe, e.g. the recently published parasites from Lake Baikal [34, 35]); (iii) published *SSU* sequences for microsporidians infecting other taxa, prioritising freshwater or brackish water invertebrates, when closely related amphipod sequences relative to newly produced sequences were not found; (iv) published *SSU* sequences representative of the five microsporidian clades (Clades I–V), as determined in the integrative phylogenies presented in literature [55, 56]. All sequences were aligned using MAFFT7.388 software [57], with the E-IONS-I algorithm using legacy gap penalty option, incorporated in Geneious 10.2.2 [53]. Our dataset consisted of sequences of variable lengths depending on both the success in producing new sequences (from 180 to 826 bp) and on various length of the published ones (from 300 to 1448 bp for microsporidians, and 1786 bp for the fungus *Basidiobolus ranarum* used as an outgroup). All details, including sequence length, are given in Additional file 4: Table S3. Alignments are given in Additional file 5: Data S2. As some sequences were relatively short, reducing the full dataset to a standard size would, on the one hand, allow defining haplotypes but, on the other hand, would potential induce losing phylogenetic signal. Therefore, we attributed each sequence to haplogroups, defined in such a way that sequences belonged to distinct haplogroups if they differed by one or more variable sites, generating diagnostic features (Additional file 2: Table S2), whatever sequence length. Two sequences were clustered in one haplogroup, despite variable length, based on 100% pairwise identity, therefore sharing the same diagnostic sites. A limited set of newly produced sequences could be assigned to at least two haplogroups due to a combination of reduced length and lack of diagnostic features. Only the longest sequence representing each haplogroup was used for the phylogeny reconstruction (248 to 826 bp, noted in Additional file 4: Table S3; see also alignments in Additional file 5: Data S2).

Bayesian phylogeny reconstructions were performed with MrBayes [58] incorporated in Geneious 10.2.2. The best-fitting model of nucleotide substitution was determined with JModelTest-2.1.10. [59]. This was always the General Time Reversible (GTR) model with gamma-distributed rate heterogeneity (G) and a significant proportion of invariable sites (I). Four heated chains, each 1,100,000 iterations long, sampled every 200 iterations, were run. The runs reached satisfactory effective sampling sizes (ESS > 200), and the potential scale reduction factor values equalled 1 for all parameters. The 50% majority-rule consensus tree was constructed after the removal of 10% ‛burn-inʼ trees. Four phylogenetic trees were constructed. The first tree contained all haplogroups (i.e. sequences from this study and published sequences) using *Basidiobolus ranarum* (GenBank: AY635841) as the outgroup [55]. In this tree, we described novel parasites by conservatively using provisional names, e.g. *Microsporidia* sp. (hereafter abbreviated *Msp*) followed by the clade number (from I to V) *sensu* Vossbrinck et al. [55] and a superscript roman letter. The three other phylogenies represent detailed analyses for the already identified parasites of the microsporidian species of the genera infecting amphipods: *Nosema* [12], *Cucumispora* [9] and *Dictyocoela* [6]. *Nosema antherae* (GenBank: DQ073396), *Vavraia culicis* (GenBank: AJ252961), *Dictyocoela cavimanum* (GenBank: AJ438960) were used as outgroups for the *Nosema*, *Cucumispora* and *Dictyocoela* phylogenies, respectively. Following Grabner et al. [5], if a newly obtained sequence was > *c*.98% similar to a sequence for which a full taxonomic description was available, providing genus and species name, such name was ascribed to the new sequence. Alignments used for building these trees are provided in Additional file 5: Data S2).

### Geographical distribution of parasites and potential host specificity

In addition to Bayesian trees, we provided a map constructed in Qgis 2.18.4 to show the geographical distribution of the three genera *Nosema*, *Cucumispora*, *Dictyocoela* in *G. roeselii* (including GenBank data). We also added to these maps geographical positions of gammarids’ parasite sequences found in the literature (see also Additional file 4: Table S3). We did not analyse geographical distribution of other microsporidian clades, as most of them were present only in single locations.

### Host phylogeny and distribution *versus* microsporidia prevalence and phylogeny

The *cox*1 (cytochrome *c* oxidase subunit 1) results of Grabowski et al. [41, 47] were used as the backbone for *G. roeselii* phylogeography used in the present study. In addition, any individual infected by microsporidians for which the host *cox*1 sequence was not already available (e.g. additional sampling sites) was tentatively newly sequenced for *cox*1 following all the molecular procedures described by Grabowski et al. [41]. These new *cox*1 sequences were attributed, using phylogenetic reconstruction and pairwise identity, to the host MOTUs defined by Grabowski et al. [41] (Additional file 1: Table S1).

Host phylogeny and geographical distribution *versus* those of the microsporidians were assessed in two ways. First, we compared the proportion of infected populations between the three biogeographical regions: 1, 2a and 2b, using Likelihood-Ratio *χ*^2^ or Fisherʼs exact test. Secondly, we tried to confront parasite phylogeny to the phylogeny of the host. The first challenge would be to find an appropriate taxonomic level for microsporidians relative to the age of diversification, as the *G. roeselii* diversification started *c.*18 Mya [41] while the phylum Microsporidia is likely to date back hundreds of Mya [60]. Microsporidian genus level (e.g. *Nosema*, *Cucumispora* and *Dictyocoela*) might be a better choice than the phylum Microsporidia to run such an analysis. However, the number of microsporidian clades at this taxonomic level was limited relative to the high number of MOTUs observed in *G. roeselii*. For these reasons, we were not able to use co-phylogenetic methods (e.g. [61]); the comparisons were therefore made by eye, by investigating how the parasite haplogroups were distributed across the host phylogeny.

## Results

### Overall prevalence and broad geographical distribution of microsporidian infections in *G. roeselii*

The overall prevalence of microsporidian infections in *G. roeselii* was 16.6% with 316 infected individuals out of the 1904 tested. In 51 sites (54.2%), at least one *G. roeselii* individual was found to be infected with a microsporidian parasite (Fig. 1, Additional file 1: Table S1). There was nevertheless a high variation among sites, ranging from nil to even 100% in one French population (# 97, all individuals being female, Additional file 1: Table S1). This crude pattern of microsporidian infections showed a strong contrast depending on the geographical region. Infections were detected in 4/26 sites (15.4%) in Region 1, in 12/22 sites (54.5%) in Region 2a, and in 35/46 sites (76.1 %) in Region 2b (Likelihood ratio *χ*^2^ = 26.38, *P* < 0.0001) (Fig. 1, Additional file 1: Table S1). In fact, the proportion of infected populations was lower in Region 1 compared to Region 2a or 2b (Fisherʼs exact test: *P* = 0.006 and *P* < 0.0001, respectively), while the Regions 2a and 2b showed a comparable proportion of infected populations (Fisherʼs exact test: *P* = 0.095).

### Microsporidian diversity and phylogenetic position

**Fig. 2:**
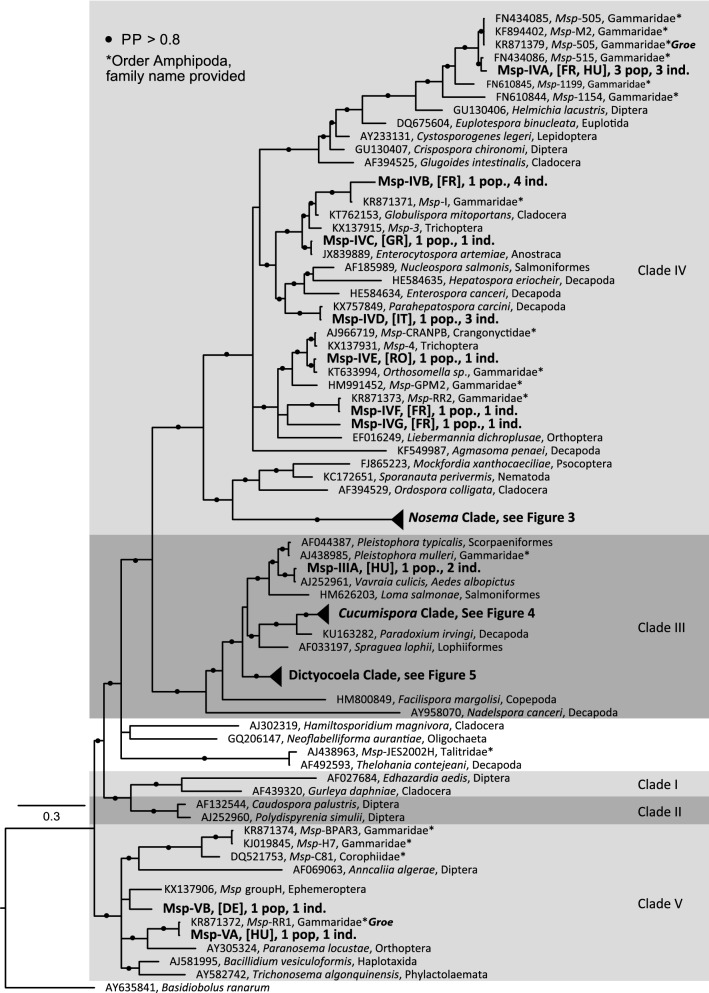
Bayesian phylogenetic reconstruction based on partial small ribosomal subunit rDNA alignment. Labels in bold are parasites of *G. roeselii* found in the present study. These labels show the name of the parasite, the country where it was found (two letter ISO code, see Additional file 1: Table S1), the number of infected populations (=pop.), and the total number of infected individuals (=ind.). Labels with accession numbers are parasite sequences taken from GenBank. These labels show the accession number, the parasite name given in the associated publication, the order of the host (except for amphipod hosts where the family is provided; if *G. roeselii* was found infected by a haplogroup, this was indicated by ‘*Groe*’). Microsporidian clade numbers are as in Vossbrinck and Debrunner-Vossbrinck [49]. Branches are collapsed according to the three genera (triangle sizes not reflecting actual size): *Nosema*, *Cucumispora* and *Dictyocoela*, as further details are given in Figs. 3, 4 and 5, respectively. *Abbreviation*: PP, Bayesian posterior probability

Depending on sequencing success, the final sequence length of microsporidian *SSU* from the 316 infected hosts ranged from 180 bp (1 case) to 840 bp, and 39.4% of the sequences were ≥ 687 bp (Additional file 4: Table S3). These sequences could be ascribed to 24 microsporidian haplogroups, which themselves could be clustered into 18 species-level taxa, based on the divergence threshold of *c*.2% (Additional file 1: Table S1 and Additional file 4: Table S3). The newly generated sequences were only associated with three (III, IV and V) of the five microsporidian clades defined by Vossbrinck and Debrunner-Vossbrinck [55]. Most of these sequences (298/316, 94.3%) could be ascribed to species of three genera already known to infect gammarid hosts: *Nosema* (96/316, 30.38%), *Cucumispora* (37/316, 11.71%) and *Dictyocoela* (165/316, 52.22%) (Fig. 2, Additional file 1: Table S1 and Additional file 4: Table S3). The remaining 18 sequences (5.7%), although being rare overall, were recorded from 11 different geographical sites and accounted for 10 out of the 24 observed haplogroups (Fig. 2, Additional file 1: Table S1).

### Rare infections (i.e. infections not ascribed to the genera *Nosema*, *Cucumispora* and *Dictyocoela*)

Most of the microsporidians representing rare infections in *G. roeselii* were either phylogenetically close to various microsporidians infecting gammarids (*Msp*-IVA, *Msp*-IVB, *Msp*-IVE, *Msp*-IVF, *Msp*-IVG, Msp-VA) or close to microsporidians infecting other crustaceans (*Msp*-IVC, *Msp*-IVD) (Fig. 2). Among the parasites linked to other gammarid infections, *Msp-*IVA was found in France and Hungary (populations 37, 39 and 91, Fig. 1, Additional file 1: Table S1). It was very close to *Msp-*515 infecting Irish and French populations of *G. duebeni* (GenBank: FN434086) (97.7% identity; 311 bp coverage). The *Msp-*IVB haplogroup was found in four individuals in one French population (# 89, Additional file 1: Table S1). This sequence showed between 99.4–99.7% identity with *c.*750 bp coverage to a set of sequences including *Msp-*JES2002I (GenBank: AJ438964), previously detected in *G. pulex* from Scotland [6] and *Msp-*I (GenBank: KR871371), from *G. roeselii* in Germany [5, 26, 31]. *Msp-*IVE parasite was found in one individual in population 31 from Romania and was 99.4% similar (with 327 bp coverage) to *Orthosomella* sp. (GenBank: KT633994) infecting *G. fossarum* and the subterranean amphipod *Niphargus schellenbergi* in Luxembourg [16]. *Msp-*IVF and *Msp-*IVG haplogroups were found in one host individual each, in French populations 84 and 98 (Fig. 1, Additional file 1: Table S1), respectively. The highest match for these haplogroups in GenBank was *Msp-*RR2 (GenBank: KR871373) and microsporidians found in the Ruhr region of Germany, infecting *G. pulex*, *G. fossarum* and *G. roeselii* [26], with 99.7% and 78.6% identity, respectively (coverage 334 bp). Finally, the sequence of *Msp*-VA showed 100% identity (246 bp coverage) with *M*sp-RR1 (GenBank: KR871372) previously found by Grabner et al. [5] to infect *G. pulex* and *G. roeselii* in Germany. In the microsporidian Clade III, *Msp*-IIIA was found in two individuals from the Hungarian population 46 (Additional file 1: Table S1). This haplogroup is relatively closely related to *Pleistophora* parasites (Fig. 2), notably *Pleistophora mulleri* (GenBank: AJ438985) infecting *G. duebeni celticus* (88.6% identity, coverage 388 bp) [62], but was even more closely related to *Vavraia culicis* infecting mosquitoes (99.0% identity, coverage 384 bp) (GenBank: AJ252961) (Fig. 2). Finally, within the Clade V, *Msp*-VB was found in one G*. roeselii* from Germany, population 77 (Fig. 1, Additional file 1: Table S1). This parasite had only 75.7% identity with *Msp*-RR1 infecting gammarids and 80.3% identity with *Msp-*Group H infecting Ephemeroptera (Fig. 2). Most of identified parasites have been reported in the literature to infect gammarid hosts; however, they seem to be relatively rare and geographically widespread.

Two parasites of *G. roeselii* were related to other parasites infecting crustaceans. *Msp*-IVC was found in one *G. roeselii* individual from the Greek population 12 (Fig. 1, Additional file 1: Table S1). This sequence is identical (coverage 330 bp) to the sequence of *Enterocytospora artemiae* found in *Artemia franciscana* in France, USA and Israel [63] (GenBank: JX839889). Similarly, *Msp*-IVD, infecting three individuals from northern Italy, population 60, was 99.5% similar, with a coverage of 762 bp, to *Parahepatospora carcini* infecting, the European shore crab (*Carcinus maenas*) (GenBank: KX757849) [11].

The geographical distribution of these rare infections was similar between the geographical regions (Fig. 1, Additional file 1: Table S1), with 1/26 populations infected in Region 1 (3.8%; *Msp*-IVC only), 4/22 (18.2%) in Region 2a and 6/46 populations infected in Region 2 (13.0%; all other haplogroups) (Likelihood Ratio *χ*^2^ = 2.89, *P* = 0.23).

### Infections ascribed to the genus *Nosema*

**Fig. 3:**
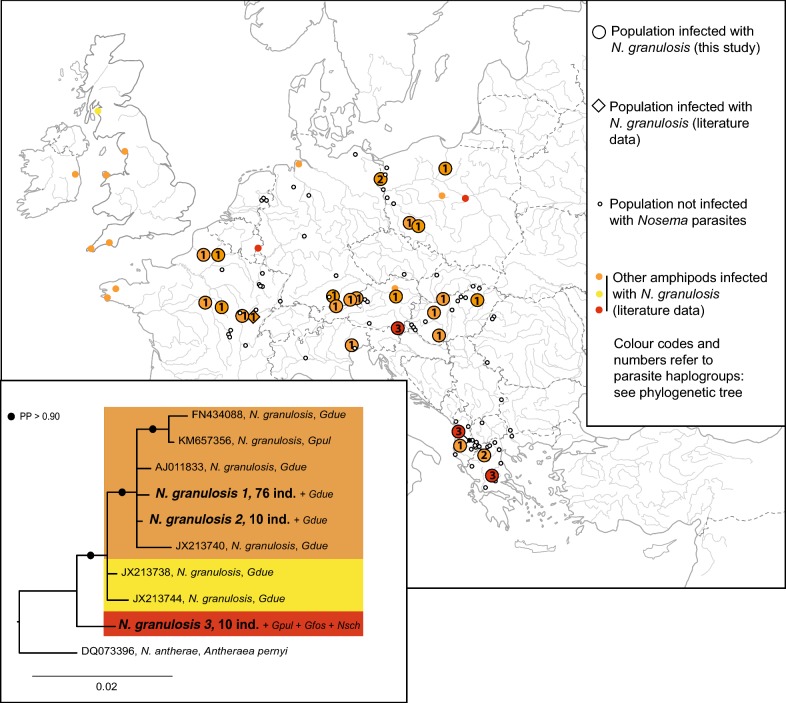
Geographical distribution and phylogenetic tree for infections by microsporidia species of the genus *Nosema* in *Gammarus roeselii.* The map shows the infections found in *G. roeselii* (large colored dots for those detected in the present study; large colored diamonds for those detected in previous studies) and in other amphipods (small colored dots, see Additional file 4: Table S3 for further details). Dot colors match clade colors on the phylogenetic tree. The Bayesian phylogenetic reconstruction is based on a small ribosomal subunit rDNA alignment of *Nosema granulosis*. *Nosema antherae* was used as the outgroup and divergent lineages were ascribed a color code. Sequences from the present study are in bold and labels include haplogroup names, the total number of *G. roeselii* infected individuals (=ind.), plus other hosts found infected by the same haplogroup. Sequences from GenBank are all other *Nosema granulosis* haplogroups (Additional file 4: Table S3). Labels include the accession number, the species name given in the associated publication and the host species abbreviated names. For abbreviations of host species names see Additional file 4: Table S3. *Abbreviation*: PP, Bayesian posterior probability

A total of 96 *G. roeselii* individuals were infected by microsporidia for which partial *SSU* rDNA sequences were ascribed to the genus *Nosema* (Additional file 1: Table S1). Three haplogroups of *Nosema* were identified in our study (Fig. 3), all belonging to *Nosema granulosis* (Fig. 3). Infections with *N. granulosis* represented 30.4% of all the microsporidian infections in *G. roeselii*. *Nosema granulosis 1* was the most frequent haplogroup, infecting 76 individuals from 19 populations across 8 countries (Fig. 3). The haplogroups *N. granulosis 1* and *2* showed, respectively, 100% and 99.8% identity with *N. granulosis* already found in *G. roeselii* from France (GenBank: AY584251) (Additional file 4: Table S3) [19]. The haplogroup *N. granulosis 3* was 100% identical with *N. granulosis* infecting the subterranean amphipod *Niphargus schellenbergi* but also *G. fossarum* individuals from Luxembourg and *G. pulex* from Poland (Additional file 4: Table S3) (GenBank: KP633991 and KM657357, respectively) [16]. This haplogroup had *c.* 98% identity with *N. granulosis* haplogroups *1* and *2* and many sequences, e.g. with *N. granulosis* found in *G. duebeni*, the type-material used to describe this microsporidian species [8] (Additional file 4: Table S3). This parasite was found mostly in the *G. roeselii* diversification hotspot (Region 1), i.e. in Albania and Greece, but also in Slovenia (Region 2a), altogether in 10 infected individuals (Additional file 1: Table S1, Fig. 3).

These parasites were found in both the host diversification areas (Region 1 and Region 2a) and the recent expansion area (Region 2b). It is worth noting that the repartition of infections with *N. granulosis 1* was uneven among the host geographical regions. Only 1/26 populations (3.85%) were infected in Region 1, 5/22 (22.73%) in Region 2a and 13/46 (28.26%) in Region 2b (Likelihood ratio *χ*^2^ = 7.79, *P* = 0.020).

### Infections ascribed to the genus *Cucumispora*

**Fig. 4:**
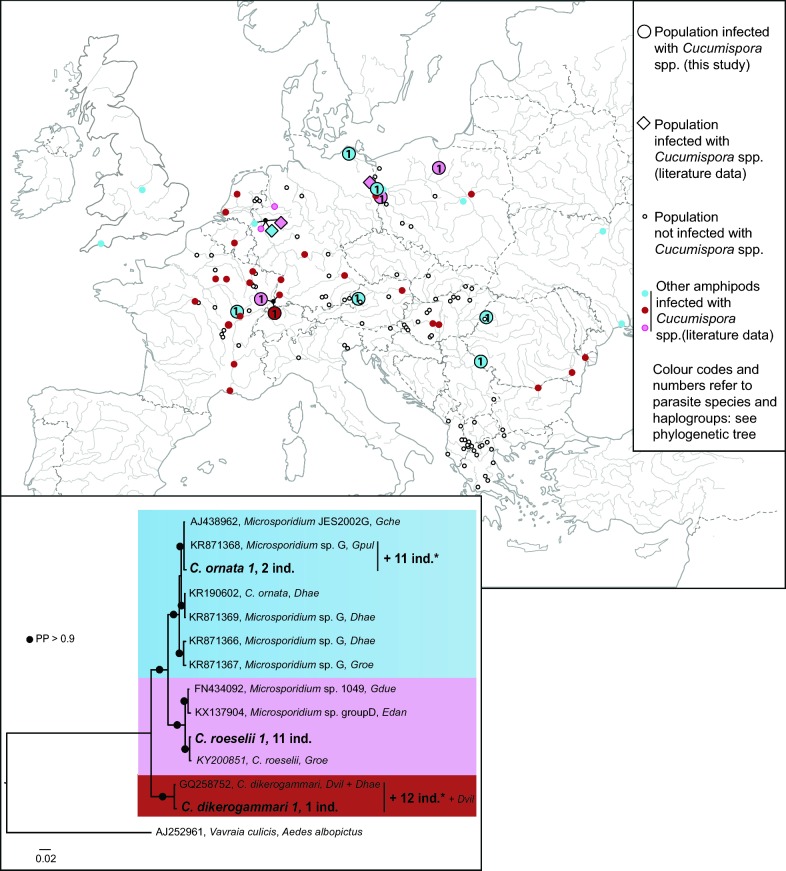
Geographical distribution and phylogenetic tree for infections by microsporidia species of the genus *Cucumispora* in *Gammarus roeselii.* The map shows the infections found in *G. roeselii* (large colored dots for those detected in the present study; large colored diamonds for those detected in previous studies), and in other amphipods (small colored dots, see Additional file 4: Table S3 for further details). Dot colors match clade colors on the phylogenetic tree. The Bayesian phylogenetic reconstruction is based on a small ribosomal subunit rDNA alignment of *Cucumispora* spp. *Vavraia culicis* was used as the outgroup and divergent lineages were ascribed a color code. Sequences from the present study are in bold and labels include haplogroup names, the total number of *G. roeselii* infected individuals (=ind.), plus other hosts found infected by the same haplogroup. Sequences from GenBank represent all other *Cucumispora* haplogroups, following Bojko et al. [20] (Additional file 4: Table S3). Labels include, in this order, the accession number, the microsporidia species name given in the associated publication and the host species abbreviated name(s). For abbreviations of host species names see Additional file 4: Table S3. *Parasites for which the sequence did not allow to distinguish their assignment between the haplogroups indicated by the vertical bar. *Abbreviation*: PP: Bayesian posterior probability

Three haplogroups close to the already described *Cucumispora* parasites, i.e. *C. ornata* [10], *C. dikerogammari* [9] and *C. roeselii* [20], were found in our study (Fig. 4). Indeed, one of the parasites, present in one host individual was 99.9% identical to the sequences of *C. dikerogammari* identified initially in another gammarid host, *Dikerogammarus villosus* [9, 17]. We named this haplogroup *C. dikerogammari 1* (Fig. 4, Additional file 4: Table S3). The second sequence was found in 11 individuals from 3 populations, showing 99.8% identity with *C. roeselii*, already found to infect *G. roeselii* in Poland [20]. We named this haplogroup *C. roeselii 1* (Fig. 4, Additional file 4: Table S3). Finally, one host individual was infected by a parasite showing 97.3% identity with *C. ornata* identified initially in *Dikerogammarus haemobaphes* [10]. We named this haplogroup *C. ornata 1* (Fig. 4). Although all newly generated sequences were informative enough to ascribe them to a species, 23 were too short (180–249 bp) to ascribe them to a specific haplogroup. This was the case for 11 sequences of *C. ornata*, that could belong both to *C. ornata 1* or to the KR871368 haplogroup (see Additional file 6: Data S3), and for 12 sequences of *C. dikerogammari*, that could belong both to *C. dikerogammari* 1 or to the GQ258752 haplogroup (Fig. 4, Additional file 4: Table S3). Altogether, the individuals infected with *Cucumispora* represented 11.7% of microsporidian infections found in *G. roeselii*. The three *Cucumispora* species were represented in almost equal proportions in our *G. roeselii* collection, with between 11–13 individuals infected by each species; however, *C. dikerogammari* was found only in one site in France (population 89).

Even if the overall low number of populations with individuals infected with *Cucumispora* prevented any statistical analyses, it is worth noting that species of the genus *Cucumispora* were found mostly in the recent expansion areas of *G. roeselii* (Regions 2a and 2b) (Fig. 4).

### Infections ascribed to the genus *Dictyocoela*

**Fig. 5:**
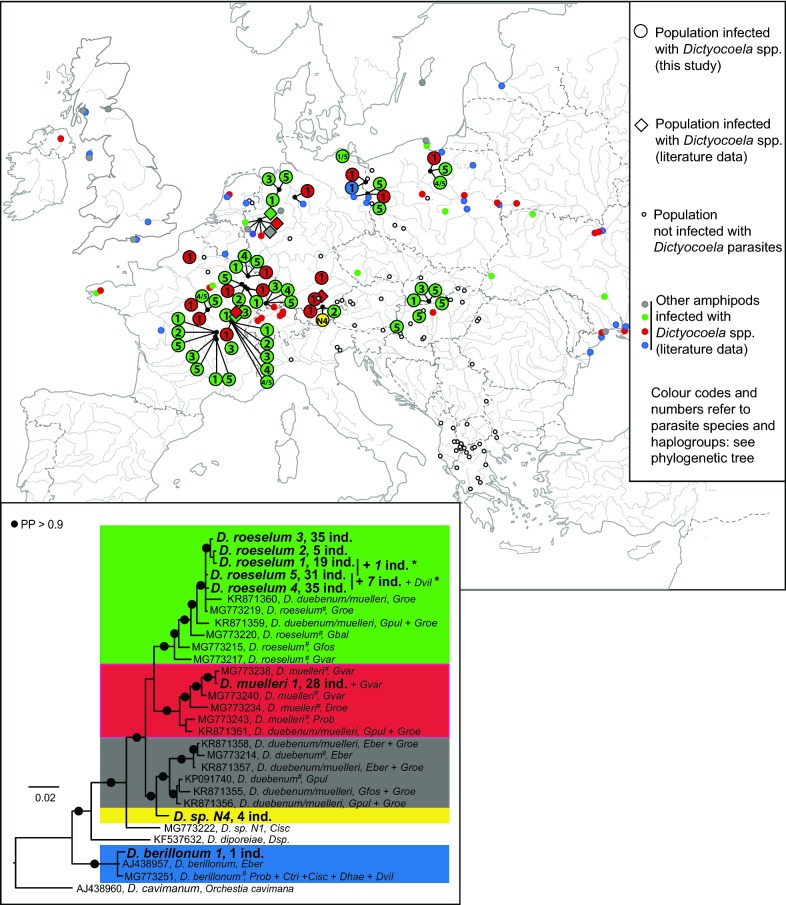
Geographical distribution and phylogenetic tree for infections by microsporidia species of the genus *Dictyocoela* in *Gammarus roeselii.* The map shows the infections found in *G. roeselii* (large colored dots for those detected in the present study; large colored diamonds for those detected in previous studies) and in other amphipods (small colored dots, see Additional file 4: Table S3 for further details). Dot colors match clade colors on the phylogenetic tree. The Bayesian phylogenetic reconstruction is based on a small ribosomal subunit rDNA alignment of *Dictyocoela spp*. *Dictyocoela cavimanum* was used as the outgroup and divergent lineages were ascribed a color code. Sequences from the present study are in bold and labels include haplogroup names, the total number of *G. roeselii* infected individuals (=ind.), plus other hosts found infected by the same haplogroup. Sequences from GenBank are only a representative panel of *Dictyocoela* diversity, divergence and host range (see Additional file 4: Table S3 and Additional file 3: Figure S1 for complete data). Labels include, in this order, the accession number, the microsporidia species name given in the associated publication and the host species abbreviated name(s). For abbreviations of host species names see Additional file 4: Table S3. *Parasites for which the sequence did not allow to distinguish their assignment between the haplogroups indicated by the vertical bar. ^#^Parasites used by Bacela-Spychalska et al. [24] to reassess species level phylogeny of *Dictyocoela* genus. *Abbreviation*: PP, Bayesian posterior probability

Seven haplogroups of *G. roeselii* parasites were phylogenetically closely related to the following three species of *Dictyocoela* parasites: *D. roeselum* [19], *D. muelleri* [6] and *D. berillonum* [6] (Fig. 5, Additional file 7: Figure S1). We found also another *Dictyocoela* haplogroup that could not be assigned to any of the already described species (Fig. 5, Additional file 7: Figure S1). Overall, *Dictyocoela* spp. were the most common microsporidian parasites infecting *G. roeselii*, with 165 individuals infected in 27 populations, corresponding to 52.2 % of all microsporidian infections found.

*Dictyocoela roeselum* parasites were the most common and the most diverse: five haplogroups were found in 133 host individuals from 23 populations. Haplogroups *D. roeselum* 1 to 5 were 99.7% similar to the closest *D. roeselum* sequence (GenBank: MG773219, [24]). *Dictyocoela roeselum* 1, 3, 5 were found in western and northern Europe (Regions 2a and 2b), while haplogroups 2 and 4 were found in Region 2b only (Fig. 5, Additional file 1: Table S1). *Dictyocoela roeselum* infections in eight *G. roeselii* individuals were associated with short sequences (see “Methods”): we were unable to assert if they belong to *D. roeselum* 4 or 5 (7 individuals), or *D. roeselum* 1 or 5 (one individual) (Fig. 5, Additional file 1: Table S1 and Additional file 4: Table S3).

A single haplogroup (called *D. muelleri* 1) infecting *G. roeselii* showed 100% identity with the sequence used originally to identify *D. muelleri* (Terry et al. [6]; parasite found in *G. duebeni* from northern Europe). This unique haplogroup was relatively widespread in the recent expansion area of *G. roeselii* (Region 2b), infecting 28 individuals from 14 different populations in Germany, France and Poland. No *G. roeselii* infected with this parasite were found in the two diversification areas (Region 1 or 2a).

We found one population in Germany with four individuals infected with an undescribed *Dictyocoela* species. We called it *Dictyocoela* sp. *N4* in the absence of morphological data enabling full species description. This haplogroup was 97.7% similar to *Dictyocoela* sp. *N1* infecting *Echinogammarus ischnus* from Poland [24] (Fig. 5, Additional file 1: Table S1, Additional file 7: Figure S1).

Finally, in one individual from Poland, we found one haplogroup of the *D. berillonum* clade (Fig. 5). It was 99.8% similar to the type-sequence used in the first discrimination of *D. berillonum* (GenBank: AJ438957, [6]). However, this sequence was too short to be distinguished from many other closely related *D. berillonum* haplogroups (Additional file 4: Table S3, Additional file 7: Figure S1) and was individualised for the sake of clarity.

### Parasite infections across *G. roeselii* phylogeny

**Fig. 6:**
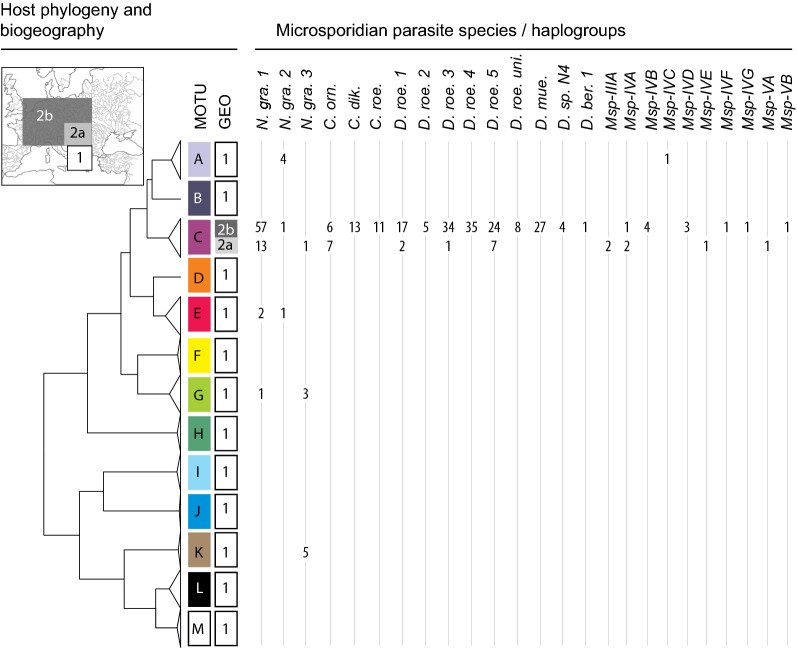
Overview of microsporidian infections in *Gammarus roeselii* according to host phylogeny and biogeography. The number of infected individuals is provided for each microsporidian haplogroup or species. The host phylogeny, the names for Molecular Operational Taxonomic Units (MOTUs) represented by letters and the geographical distribution of these MOTUs (GEO, and small map) refers to Grabowski et al. [41, 47]

The massive majority of microsporidian haplogroups and number of infected individuals were found in association with the *Gammarus roeselii* MOTU C (Fig. 6). Accordingly, because only a few genotypes of *G. roeselii* from this MOTU colonised north-western Europe ([41, 47]; see “Methods”), most infections were found in the host’s recent expansion area, i.e. Region 2b (see above, Figs. 3, 4, 5, summarised in Fig. 6).

However, it is worth noting that *Nosema granulosis* parasites were more scattered across the *G. roeselii* phylogeny than the other parasites. For example, *N. granulosis 2* and *3* were more frequently found in Region 1 than in Regions 2a and 2b. In Region 1, *N. granulosis 2* was associated with host MOTUs A and E, while *N. granulosis 3* was associated with MOTUs G and K. These host MOTUs, endemic to the Balkans, were deeply divergent from the widespread MOTU C. On the other hand, *N. granulosis 1* was found mostly in Regions 2a and 2b (infecting only the host MOTU C), while it was rare in Region 1, where it infected host MOTUs E and G (Fig. 6).

Most of the *Cucumispora* parasites were found in Region 2b, except for *C. ornata*, also found in two populations of Region 2a, all being therefore associated only with host MOTU C.

*Dictyocoela roeselum 1*, *3* and *5* were found mostly in Region 2b, but few host individuals belonging to four populations (#39, 42, 44, 49) were found infected by these haplogroups in Region 2a. Contrastingly, *D. roeselum 2* and *4* were found in Region 2b only. All were associated with host MOTU C. The other *Dictyocoela* spp., i.e. the frequent *D. muelleri* and the rare *Dictyocoela* sp*. N4* and *D. berillonum* were found only in Region 2b, also associated with the MOTU C.

The rare microsporidians were associated with host MOTU C in Regions 2a and 2b, except for *Msp-*IVC, associated with MOTU A in Region 1 (Fig. 6).

## Discussion

### Diversity of microsporidian infections in *Gammarus roeselii* across its entire range

Our study revealed that *Gammarus roeselii* is infected by several microsporidian taxa, represented by at least 24 haplogroups. Part of the newly generated sequences are relatively short, leading to possible underestimation of very closely related haplogroups that could be distinguished only with longer sequences. However, even if *SSU* rDNA is sometimes limited at resolving phylogenies in the Microsporidia at higher taxonomic levels, it has been recently shown that phylogenetic reconstruction based on this marker reflect accurately multimarker phylogenies at the generic level [24]. Thereby, even short sequences (V1f-UNIr) convey enough phylogenetic information for species-level divergence assessment. The 24 haplogroups could be clustered into 18 species-level taxa and, in part, ascribed to already described species (see below). Most of them were associated with Clades III and IV defined by Vossbrinck and Debrunner-Vossbrinck [55]: only two were associated with Clade V and none with Clade I and II.

Infections were found in around half of the investigated populations, ranging from low (prevalence of *c.*5%) to frequent (prevalence of 100%). However, low sampling at some sites may challenge prevalence estimates. More interestingly, the 18 species-level taxa could be classified into two broad categories related to infection patterns: species-level taxa with rare occurrence of infection (present in ≤ 3 populations) and species-level taxa with frequent occurrence (parasites present in more than 10 populations). The three clades with frequent occurrence belong to three microsporidian genera well identified to comprise amphipod-infecting parasites: *Nosema*, *Cucumispora* and *Dictyocoela* [6, 8–10, 24].

The rare infections in *G. roeselii* consisted of 10 parasite haplogroups. Although they represent a high diversity, their detection is challenging, because they represent a neglected parasitic fauna if sampling effort is limited [5, 18, 26, 31]. Nevertheless, most of them were phylogenetically closely related to parasites from other amphipods (sometimes sequences were identical), even sampled from different regions of Europe. This is the case for *Msp*-IVA, *Msp*-IVB, *Msp*-IVE, *Msp*-IVF and *Msp*-VA (Fig. 2). They were all infecting a single host (MOTU C), in the region recently colonised by *G. roeselii*. These parasites may represent generalists, infecting a variety of gammarids. These generalist parasites could have been acquired by *G. roeselii* by horizontal transfers while living in sympatry with local gammarid species (not investigated in the present study). Grabner et al. [5] proposed such a hypothesis for some of these parasites. However, we cannot dismiss a hypothesis that these parasites come from recent, transient, host shifts that will not sustain transmission within the new *G. roeselii* host. The rare infections *Msp*-IVG, *Msp*-IIIA and *Msp*-VB, were less closely related to other gammarid parasites (Fig. 2). They might represent rare cases of horizontal transfers from other, completely unstudied, aquatic taxa. The closest relatives of *Msp*-VB, for example, are parasites infecting aquatic insects (Fig. 2). The case of *Msp*-IIIA haplogroup seems similar. It is very closely related to *Vavraia*, a genus recognised as *Pleistophora*-like microsporidia [64] associated with insects. This pattern is advocating for a transient association with *G. roeselii*, perhaps through the trophic chain, gammarids being both scavengers and predators of other members of the macroinvertebrate community [65].

The remaining microsporidian parasites detected as rare in *G. roeselii* were phylogenetically close to other crustacean microsporidians. *Msp*-IVC found at a site in continental Greece had the same sequence as *Enterocytospora artemiae* from *Artemia franciscana* present in the USA, France and Israel [63], and *Msp*-IVD found in *G. roeselii* form mainland Italy was very close to *Parahepatospora carcini* infecting *Carcinus maenas*, the European shore crab [11, 63]. How is it possible that these parasites from salt water can be found in freshwater animals? A direct horizontal transfer can be excluded, the two sites being too far from the seashore, preventing direct contact between *G. roeselii* and crabs or *Artemia*. However, these sites were located at around 100 km from Aegean or Ligurian seas, respectively (Fig. 1). We can first hypothesise a long-distance transport of microsporidian spores, e.g. by migratory shorebirds or birds associated with both marine and freshwater environments such as seagulls. These birds may have consumed crustaceans, released parasite spores with their faeces, which could have been consumed by *G. roeselii*. We cannot discriminate if these parasites are real infections or are food-borne microsporidia just passing through the gut. A second hypothesis could be that these microsporidia are generalists, infrequently found in various types of host over large geographical areas, both in fresh and saline waters.

### Infections ascribed to the genus *Nosema*

Since the early work of Terry et al. [8], it is known that *Nosema granulosis* infects at least seven amphipod species. *Gammarus roeselii* is one of its hosts, since Haine et al. [19] found one haplogroup of the parasite in three French populations. This haplogroup was 100% similar to *N. granulosis 1* found in the present study. Our study extended this observation geographically, and we additionally detected two other haplogroups (*N. granulosis 2* and *3*) in Albania, Austria, Germany, Greece, Hungary, Italy and Poland. *Nosema granulosis 1* is the most frequent haplogroup in our data. It is widely distributed in *G. roeselii*, but predominantly in Region 2b of the host range (Fig. 4). The main mode of transmission of *N. granulosis 1* in *G. roeselii* and *N. granulosis* in *G. duebeni* is the vertical one (from mother to offspring, *via* eggs). It induces sex ratio distortion in the host populations by reversing males into functional females [8, 19, 48, 66]. We may, therefore, suggest that this peculiar mode of transmission associated to overproduction of female hosts may have helped *N. granulosis 1* to colonise a large area, and even helped the host *G. roeselii* to rapidly spread in north-western Europe. This hypothesis was previously proposed to explain the invasive success of *Crangonyx pseudogracilis* colonising Europe [67]. Indeed, an excess of females generated by the infection may have increased the population dynamics of *G. roeselii*. Neither *N. granulosis 2* nor *N. granulosis 3* showed such a high frequency or large geographical distribution. Their vertical transmission and feminizing effect are therefore questionable, and more data are needed to reveal the effects they may have on the host phenotype.

All of the *N. granulosis* haplogroups infecting *G. roeselii* are scattered throughout the host phylogeny (Fig. 6), and this was the only parasite with such a pattern in our study. Notably, parasites of these haplogroups infect hosts present in the ancient diversification area (Region 1), as well as hosts present in the secondary diversification area (Region 2a). It is therefore tempting to propose that the infection by *N. granulosis* is ancient in *G. roeselii*, and that host–parasite co-diversification occurred after an initial ancestral infection. This hypothesis will be interesting to test in the future, by addressing the following issues. First, even if *SSU* rDNA is a useful marker for microsporidian phylogeny, it might not appear to be variable enough in this case for a detailed exploration of *N. granulosis* diversification history. Thus, additional markers such as RPB1 [68] could be useful to the dataset to step forward in addressing specificity. Secondly, the precise host diversification process in the secondary diversification area (Region 2a) remains to be explored in details [47]. It will notably be useful to understand if variation within the host MOTU C fits variation within *N. granulosis*. Thirdly, all *N. granulosis* haplogroups infecting *G. roeselii* are shared between this host and other amphipod hosts (*G. duebeni* for *N. granulosis 1* and *2*; *Niphargus schellenbergi*, *G. fossarum* and *G. pulex* for *N. granulosis 3*; see Fig. 5, Additional file 4: Table S3). It has been proposed that interspecific horizontal transmission may occasionally occur, which could be a survival strategy of *N. granulosis* in ephemeral habitats [69]. Horizontal transmission was also evoked to explain the presence of the same parasite haplogroup in subterranean and surface amphipods [16]. Our results are in agreement with such hypotheses. However, another hypothesis would be worth exploring, namely that infection with *N. granulosis* is very ancient in amphipods, and that these parasites co-diverged with the host species. Such a hypothesis assumes that *SSU* rDNA is not an appropriate marker for revealing such a pattern because the same haplogroups are shared by very divergent host species. Therefore, again, it requires employment of more variable markers. The multiplication of studies over an entire range of other gammarid species or studies testing vertical transmission [8, 19, 48, 66] would provide more opportunities to test these hypotheses.

### Infections ascribed to the genus *Cucumispora*

We found three haplogroups within the genus *Cucumispora* [9]. Two of the sequences in our samples were almost identical to *C. dikerogammari* and *C. roeselii*. The third one was more distantly related to *C. ornata* (97.7% identity), but, following Bojko et al. [20], the similarity level suggests that it could be considered as belonging to *C. ornata*. *Cucumispora* are horizontally-transmitted parasites, virulent for their amphipod hosts. *Cucumispora dikerogammari* and *C. ornata* followed the hosts during their invasion of western Europe [10, 17]. They also have the potential to shift hosts and threaten local gammarid species [21, 70]. We hypothesise that the scattered infection pattern in *G. roeselii* with individuals infected by one clade of microsporidia may be due to interspecific horizontal transfers in sites where the original infected hosts live in sympatry with other gammarid species. This is strengthened by the fact that *Cucumispora* parasites are absent in Region 1 of *D. roeselii*, where the Ponto-Caspian hosts of *C. dikerogammari* and *C. ornata* are seldom present. The only infection with *C. dikerogammari* was identified where *G. roeselii* co-occurs with *D. villosus* (population 89) so it can be treated as the first evidence of a host shift observed in this parasite as was suggested by Bojko et al. and  Bacela-Spychalska et al. [20, 21].

### Infections ascribed to the genus *Dictyocoela*

With 27 populations and 165 individuals infected, *Dictyocoela* were the most abundant parasites found in our survey, confirming their status of dominant microsporidian infections in gammarids [24]. However they were found to infect only the host MOTU C in Regions 2a and 2b (Figs. 5, 6). We found eight haplogroups of *Dictyocoela*, corresponding to three previously identified species, i.e. *D. roeselum*, *D. muelleri*, *D. berillonum*, plus an unidentified one: *Dictyocoela* sp*. N4* (Fig. 5). Recently, Bacela-Spychalska et al. [24] re-assessed *Dictyocoela* diversity based on the integrative approach combining phylogenetic, large geographical survey and ultrastructural data, and this paper provides an ideal backbone of our discussion.

*Dictyocoela roeselum* is the most abundant *Dictyocoela* in *G. roeselii* regarding both the total number of infected hosts and the number of populations with high prevalence. This result is analogous to previous studies, with prevalence reaching 60% in some local populations [19]. In our study, five haplogroups have been identified thus revealing this microsporidian to be the most diversified one in *G. roeselii*. This species has been described as infecting sporadically some other gammarid species such as *G. fossarum*, *G. varsoviensis*, *G. balcanicus* and *D. villosus* ([17, 24]; Additional file 4: Table S3, Additional file 7: Figure S1, Fig. 5). However, each host species is infected with a different, divergent, *D. roeselum* haplogroup [24]. Therefore, it seems that the particular *D. roeselum* haplogroups show some host specificity. *Dictycoela roeselum* was shown to be vertically transmitted in *G. roeselii* [19], strengthening this possibility, although the precise haplogroup could not be determined at that time. The exception could be the microsporidian found in single *D. villosus* individuals in two populations [17], which could be explained by the acquisition of the parasite by this predatory species, upon feeding on infected *G. roeselii*, as both the host species co-occurred in these sites. *Dictycoela roeselum* was absent in Region 1 of *G. roeselii*, but three haplogroups were present in Region 2a. This suggests that the infection by this parasite was as ancient as the secondary diversification of the host within the Pannonian basin (but younger than the primary diversification in the Balkans) and that these co-diversified parasites were carried during colonisation of the Region 2b. The presence of two supplementary haplogroups in this region suggests that parasite diversification is still ongoing in the region of recent invasion. This would assume a high nucleotide substitution rate in the parasite species (the colonisation of Region 2b was recent, probably post-glacial, see [47]), for which we have no information. Alternatively, underestimation of *D. roeselum* diversity in Region 2a, due to smaller sample size, cannot be dismissed.

Contrasting with *D. roeselum*, only a single haplogroup of *D. muelleri* was observed in our samples. It was nevertheless widely distributed, being present in 14 different populations from western and northern Europe (Region 2b). *Dictycoela muelleri* has been observed to infect numerous other gammarids (*D. haemobaphes*, *D. villosus*, *G. aequicauda*, *G. duebeni*, *G. varsoviensis* and *Pontogammarus robustoides*), sometimes at high prevalence [6, 17, 18, 24, 71]. In *G. roeselii*, this parasite was shown to use vertical transmission, and its role in sex ratio distortion was proposed [19]. Therefore, two parasites (*D. roeselum* and *D. muelleri*) with a similar life-cycle in a single host (vertical transmission) display different diversity patterns in *G. roeselii*. It is possible that *D. muelleri* only recently infected *G. roeselii* from local host species after it colonised the Region 2b, and, thanks to vertical transmission and sex ratio distortion in its host, rapidly spread throughout Europe. Alternatively, *D. roeselum* would represent a rather ancient infection for *G. roeselii*, which has co-diversified during the secondary diversification of its host.

We found one haplogroup of *D. berillonum*, highly similar to the haplogroup described from *Echinogammarus berilloni* [6]. This microsporidian species was previously found mainly in Ponto-Caspian hosts or other closely-related gammarids [5, 24, 25, 72]. Numerous *D. berillonum* infections result from successful co-invasion of the parasites alongside their host invasion [5, 72]. Moreover, the same *D. berillonum* haplogroups can infect several host species (Fig. 5, [24]). *Dictycoela berillonum*, therefore, does not show host-specificity, suggesting a high rate of horizontal transmission. The infected *G. roeselii* individual we found in Poland may, therefore, have acquired this parasite that way.

Finally, we encountered one haplogroup of uncertain phylogenetic proximity (*Dictyocoela* sp. *N4*). It was 97.7% similar to a set of sequences called *Dictyocoela* sp. *N1*, *2*, *3* [24] but also 97.4% similar to *D. duebenum* [24]. *Dictyocoela* sp. N4 is therefore a temporary name for the species, awaiting ultrastructural data for full species description. The three other *Dictycoela* sp. *N* previously described were only infecting the Ponto-Caspian *Echinogammarus ischnus* in Poland [73]. The single population where we found *Dictyocoela* sp. *N4* is situated in Germany, within the area where *E. ischnus* occurs in sympatry with *G. roeselii*. We may, therefore, infer that the presence of this parasite in the latter species originates from a recent (perhaps transient) host shift.

### Comparison of microsporidian diversity in *G. roeselii* with other gammarid hosts

Our results can be compared with the only other published study concerning the whole Microsporidia community over the European geographical range of a single host species, i.e. *Gammarus duebeni* from the north-western Europe [15]. We found a lower proportion of infected populations in *G. roeselii* (51/94 in our study *vs* 32/35 in *G. duebeni*; Fisherʼs exact test, *P* < 0.0001) but a higher parasite haplogroup diversity (24 *vs* 11 haplogroups). This was found despite a higher numbers of populations sampled (94 *vs* 35) but a comparable sampling effort per population (20.2 ± 1.90 individuals/population in our study *vs* 25.5 ± 4.25 in [18]; *F*_(1.132)_ = 1.72; *P* = 0.19). *Gammarus roeselii* and *G. duebeni* shared the same prevailing microsporidian genera: *Dictyocoela*, *Nosema* and *Cucumispora* (the latter group was not described yet at the time the results for *G. duebeni* were published, but the haplogroup *Msp*-1049 (GenBank: FN434092) falls within this clade. Some major differences can nevertheless be noted between the infection patterns in *G. roeselii* and *G. duebeni*. First, some infections that we qualified as “rare” in *G. roeselii* are more common in *G. duebeni*. This is the case of *Pleistophora*-*Vavraia*-like parasites, being present in seven populations of *G. duebeni* but only in one of *G. roeselii*. Similarly, but in larger proportions, *Msp*-505-515 were found in only three populations of *G. roeselii* (*Msp*-IVA in our study), but in 10 populations of *G. duebeni* [18]. Since parasites of this group have also been identified in other species of *Gammarus* [5, 31], it is tempting to suggest, following [5], that they are ubiquitous in *Gammarus* and may show a horizontal transmission pattern as well as low host specificity. Conversely, while relatively abundant in *G. roeselii*, *D. muelleri* was much rarer in *G. duebeni* [18]. Finally, the sharp disproportion in prevalence of *Dictyocoela* species between the two hosts is particularly interesting for understanding the evolution of microsporidians within the family Gammaridae. While *D. duebenum* was predominant in *G. duebeni*, we did not find it in *G. roeselii* in our study (however, in other studies, it was found in Germany [5], see Fig. 5); the reverse is true for *D. roeselum.* It, therefore, seems that, even if some haplogroups of these parasites were sporadically found in other gammarid species [24, 25]; these two parasites might show a certain amount of host specialisation. All these differences may be due to different co-evolutionary histories of the two host–pathogen associations (e.g. *G. roeselii* presents a higher cryptic species diversity compared to *G. duebeni* in Europe). The lack of overlap in the geographical distribution of the two host species may also prevent microsporidian host shifts, thereby leading to these contrasting patterns. Only an increasing number of studies similar to the present one and the one conducted by Krebes et al. [18], involving hosts with overlapping distributions, would allow to discriminate between these competing hypotheses.

Another study, although conducted on only a smaller part of the host geographical range, showed that the invasive Ponto-Caspian species *Dikerogammarus villosus* also harbours a substantial microsporidian parasite diversity [17]. In this species, the most abundant parasite was *Cucumispora dikerogammari* [9], the parasite being quite rare in *G. roeselii* (Fig. 4). Infections with *Nosema* and *Dictyocoela* parasites represented less than 1% in *D. villosus*, sharply contrasting with the prevalence observed in the present study (Figs. 3 and 5). It is worth noting that the geographical area studied for *D. villosus* partially overlaps with the geographical range of *G. roeselii* in central-western Europe and in the northern Balkans. This overlap is nevertheless recent, after the invasion of *D. villosus* in these areas in the last 30 years [74]. Therefore, it is probable that the differences in infection patterns reflect the different host–parasite evolutionary histories before the overlap of the geographical ranges of different hosts. The slight similarities could be due to high probabilities for interspecific transfers of parasites after this overlap [17, 22].

As noted by Pilosof et al. [75] and Wells et al. [76], the parasite assemblage of a given spreading host species often highly depends on the host–parasite network met by this species in a newly colonised area. Since *G. roeselii* is a species expanding its range, the comparison with the study of Grabner et al. [5] may help understand if *G. roeselii* shares parasitic fauna with local hosts. In Grabner et al. [5], *G. roeselii* was found to be infected with 11 microsporidian haplogroups representing four parasite species-level taxa: *D. duebenum/muelleri* complex, *Msp*-G, *Msp*-RR1, *Msp*-505. They shared these haplogroups with the local gammarid assemblages at the scale of the study (tributaries to the Rhine River, an area representing *c.*1000 km^2^). It is nevertheless worth noting that some of the dominant species detected in our study were absent in *G. roeselii* in [5] (e.g. *Nosema*), indicating that it would be useful to investigate other local assemblages to compare precisely local and global parasite fauna of *G. roeselii*.

## Conclusions

Microsporidian infections are common, diverse and widespread in *Gammarus roeselii* over its entire European geographical range. Two microsporidian species share infections between regions of host differentiation (Region 1 and/or 2a) and the recently colonised area (Region 2b): *Nosema granulosis* and *Dictyocoela roeselum.* For these two species, an evolutionary scenario of co-diversification with the host is a reasonable hypothesis. These patterns sharply contrasted with those of *Dictyocoela muelleri* and of the three species of *Cucumispora* parasites. In the latter, a single haplogroup per parasite species was found associated to many populations of Region 2b and therefore only to the host MOTU C. It seems parsimonious to explain these patterns as secondary acquisitions by host shifts from local gammarid species, after recent colonisation of this area by *G. roeselii*, rather than invoking an ancient infection and a secondary loss of parasites in the diversification area. Similar patterns of interspecific parasite transfers would also explain most of the rare infections, because they were close to parasites from other gammarid species. Indeed, it is known that host shifts are more probable between phylogenetically related hosts than between unrelated hosts [75, 77]. Our data cannot distinguish between recent spillover events (i.e. transient infections) and sustained transmission within the new *G. roeselii* host, but we know from [23] that *D. muelleri* is vertically transmitted in this host and, therefore, is well established in *G. roeselii* populations.

## Additional files

**Additional file 1: Table S1.** Microsporidian infections in the 94 populations investigated over the geographical range of *Gammarus roeselii*.

**Additional file 2: Table S2.** Variable sites in sequences of the three major microsporidian genera used to construct phylogenetic trees (Figs. 3, 4, 5).

**Additional file 3: Data S1.** Alignments based on *SSU* rDNA sequences, used for Additional file 2: Table S2.

**Additional file 4: Table S3.** Individual data for microsporidian infections from this study and found in GenBank (NCBI), mainly for freshwater and brackish waters amphipod species occurring in Europe.

**Additional file 5: Data S2.** Alignments based on *SSU* rDNA sequences, used for trees in Figs. 2, 3, 4, 5 and Additional file 1: Figure S1.

**Additional file 6: Data S3.** Sequences under 200 bp for which no GenBank number can be attributed.

**Additional file 7: Figure S1.** Phylogenetic tree for infections by microsporidians of the genus *Dictyocoela* in European freshwater amphipods, including all haplogroups. Bayesian phylogenetic reconstruction based on small ribosomal subunit rDNA alignment of *Dictyocoela spp*. *Dictyocoela cavimanum* was used as outgroup. Divergent lineages were ascribed to the same color code as Fig. 5, and follow recent reassessment of the genus taxonomy by Bacela-Spychalska et al. [24]. Sequences from the present study are in bold and labels include haplogroup names, and the hosts found infected by the haplogroup. Sequences from GenBank are all other *Dictyocoela* haplogroups (Additional file 4: Table S3). Labels include, in this order, the accession number, the microsporidia species name given in the associated publication and the species abbreviated name(s). For abbreviations of host species names: see Additional file 4: Table S3. Numbers on the branches indicates Bayesian posterior probability.

## Data Availability

All the data are available from the laboratory of Biogeosciences, University Bourgogne Franche-Comté, Dijon, France and material is available at the Department of Invertebrate Zoology and Hydrobiology, University of Lodz, Poland, upon request. GenBank accession numbers of partial *SSU* rDNA sequences generated in this study are MK719236-MK719541.

## Abbreviations

*SSU*: small subunit ribosomal RNA gene
*Msp*: *Microsporidia* sp.
SNP: single nucleotide polymorphism
MOTU: molecular operational taxonomic unit
Mya: million years ago
