# Supplementary material for: Microsporidian infections in the species complex Gammarus roeselii (Amphipoda) over its geographical range: evidence for both host–parasite co-diversification and recent host shifts
Source: Parasit Vectors. 2019 Jun 28;12:327. doi: 10.1186/s13071-019-3571-z (PMC6599290; doi:10.1186/s13071-019-3571-z)
Supplement: Supplementary file 3 — Additional file 3: Data S1. Alignments based on SSU rDNA sequences, used for Additional file 2: Table S2. [file 13071_2019_3571_MOESM3_ESM.docx]

Microsporidian infections in the Amphipoda species complex *Gammarus roeselii* over its geographic range: evidence for both host-parasite co-diversification and recent host-shifts.

Adrien Quiles, Karolina Bacela-Spychalska, Maria Teixeira, Nicolas Lambin, Michal Grabowski, Thierry Rigaud & Rémi A. Wattier

Additional file 3 - Supplementary data S1. Alignments based on SSU rDNA fragment sequences.

Figure 3 : *Nosema*

>GR11-06_GR_Groe_Ngra02_ADR-18_714pb_GR11-06-Ngra02

TTGAATATAAAGAAAAGACGAACAGCTCAGTAACTCTTATTTTATTTGATGTATTAGGATTATAACTATGTTAAATTATAGATAACAATAATACAATAAGAATAAGATCTATCAGTTAGTTGTTAAGGTAATGGCTTAACAAGACTATGACGGATAACGGTATTACTTTGTAATATTCCGGAGAAGGAGCCTGAGAGATTGCTACTAAGTCTAAGGATTGCAGCAGGGGCGAAACTTGACCTATGATATGATATTGAGGCAGTTATGAGTAGTATTTTTAATTATTGTAGTATTGTAAGTATATACTACAAGATAAATCGGAGGGCAAATCGAGTGCCAGCAGCCGCGGTAATACTTGTTCCGATAGTGTGTATGATGATTGATGCAGTTAAAAAGTCTGTAGTTTATGAT-TAATAAGCATTGTGAGGTATATTGTATGGTTAGGAGAGAGATGAAATGTGATAACCCTAACTGGATGAACAGAAGCGAAAGCTATATACTTAAATGTATTATTAGAACAAGGACGTAAGCTAGAGGATCGAAGATGATTAGATACCATTGTAGTTCTAGCAGTAAACTATGTTAAATCATAGATACA-TTTTAATATGTATTTATGTAAAGAAATTAAGATTATATTGACTCTGGGGATAGTATGATCGCAAGATTGAAAATTAAAGAAATTGACGGAAGAATACCACAAGGAGTGGATTGTGC-----

>PA-02_AT_Groe_Ngra01_ADR-18_687pb_PA-02-Ngra01

ACTCTTATTTTATTTGATGTATTAGGACTATAACTATGTTAAATTATAGATAACAATAATACAATAAGAATAAGATCTATCAGTTAGTTGTTAAGGTAATGGCTTAACAAGACTATGACGGATAACGGTATTACTTTGTAATATTCCGGAGAAGGAGCCTGAGAGATTGCTACTAAGTCTAAGGATTGCAGCAGGGGCGAAACTTGACCTATGATATGATATTGAGGCAGTTATGAGTAGTATTTTTAATTATTGTAGTATTGTAAGTATATACTACAAGATAAATCGGAGGGCAAATCGAGTGCCAGCAGCCGCGGTAATACTTGTTCCGATAGTGTGTATGATGATTGATGCAGTTAAAAAGTCTGTAGTTTATGAT-TAATAAGCATTGTGAGGTATATTGTATGGTTAGGAGAGAGATGAAATGTGATAACCCTAACTGGATGAACAGAAGCGAAAGCTATATACTTAAATGTATTATTAGAACAAGGACGTAAGCTAGAGGATCGAAGATGATTAGATACCATTGTAGTTCTAGCAGTAAACTATGTTAAATCATAGATACA-TTTTAATATGTATTTATGTAAAGAAATTAAGATTATATTGACTCTGGGGATAGTATGATCGCAAGATTGAAAATTAAAGAAATTGACGGAAGAATACCACAAGGAGTGGATTGTGCGGCTT

>GR25-02_GR_Groe_Ngra03_ADR-18_716pb_GR25-02-Ngra03

TTGAATATAAAGAAAAGACGAACAGCTCAGTAACTCTTATTTTATTTGATGTATTAGGATAATAACTATGTTAAATTATAGATAATAATAATACAATAAGAATAAGATCTATCAGTTAGTTGTTAAGGTAATGGCTTAACAAGACTATGACGGATAACGGTATTACTTTGTAATATTCCGGAGAAGGAGCCTGAGAGATTGCTACTAAGTCTAAGGATTGCAGCAGGGGCGAAACTTGACCTATGATATTATATTGAGGCAGTTATGAGTAGTATTTTTAATTATTGTAATATTGTAAGTATATATTACAAGATAAATCGGAGGGCAAATCGAGTGCCAGCAGCCGCGGTAATACTTGTTCCGATAGTGTGTATGATGATTGATGCAGTTAAAAAGTCTGTAGTTTATTTTATAATAAGCATTGTGAGGTATATTGTATGGTTAGGAGAGAGATGAAATGTGATAACCCTAACTGGATGAACAGAAGCGAAAGCTATATACTTAAATGTATTATTAGAACAAGGACGTAAGCTAGAGGATCGAAGATGATTAGATACCATTGTAGTTCTAGCAGTAAACTATGTTAAATCATAGATATACTTTTAATGTATATTTATGTAGAGAAATTAAGATTATATTGACTCTGGGGATAGTATGATCGCAAGATTGAAAATTAAAGAAATTGACGGAAGAATACCACAAGGAGTGGATTGTGC-----

Figure 4 : *Cucumispora ornata*

>>ALP53-04_AT_Groe_Corn_ADR-18_407pb_ALP53-04-Corn

GCATGTGTAAGCGAACAATTAGGGAGCTGCGGACTGCTCAGTAACAGGCGATTAATTTAATCTTTACAAA-------CGGACAAACTCAGGAAACGGAGTGTAATACGTAAAAGATGATTTTTTATTT-AAAAAAGAAAACATTTTTAGCTTGAATAAAGCGGTAAAGAATAAGACGCCAACCCATCAGTTAGTAAGTAGGGTAAGGGCCTATTTAGACGAATACGGGTACGGGGAATTAGGGTTTGATTCCGGAGAGGGAGCCTGAGAGACGGCTACCAGGTCCAAGGACGGCAGCAGGCGCGAAAATTACCGAAGCTCAAATAGAGGCGGTAGTAATGAGACGTATAAATAATAATATAAGGGTAAAAAACTTATTAATAACTGGAGGTCAAGTCTGGTGCCAGCAGCCGCGG

>>KR871368_DE_Gpul_Msp-G_GRA-15_786pb_KR871368

CGAACAATTAGGGAGCTGCGGACTGCTCAGTAACAGGCGATTAATTTAATCTTTACAAA-------CGGACAAACTCAGGAAACGGAGTGTAATACGTAAAAGATGATTTTTTATTT-AAAAAAGAAAACATTTTTAGCTTGAATAAAGCGGTAAAGAATAAGACGCCAACCCATCAGTTAGTAAGTAGGGTAAGGGCCTATTTAGACGAATACGGGTACGGGGAATTAGGGTTTGATTCCGGAGAGGGAGCCTGAGAGACGGCTACCAGGTCCAAGGACGGCAGCAGGCGCGAAAATTACCGAAGCTCAAATAGAGGCGGTAGTAATGAGACGTATAAATAATAATATAAGGGTAAAAAACTTATTAATAACTGGAGGTCAAGTCTGGTGCCAGCATCCGCGGTAATACCAGCTCCAGGGGTGTCTATGATGATTGCTGCGATTAAAAGGTCCGTAGTCGAATTTAAATAATTGTTTGTAATATGTTAGATAAAATAATAAAAAGAACAATTACTTAAATGAAAGGAATAACAAGAGGTTGATTAATTGAGTAACGAGAGGTGAAATTTGATGACTTACTTAGGAGAAACAGAGGCGAAAGCGTCAATCAAGTGTAAATCCGATGATCAAGGACGTAGGCTGGAGTATCGAACACGATTAGATACCGTAGTAGTTCCAGCAGTAAACTATGCCTACGCCAATAAATAAAAGTTTAATGGACGAGAAATCTAGAGTAGGGCTTTGGGGAGAGTACACGCGCAAGCGATAAATTTAAAGGAAATTGACGGAAGAA

>>AJ438962_GB_Gche_Msp-JES2002G_TER-04_1327pb_AJ438962

ATTTAGCCATGCATGTGTAAGCGAACAATTAGGGAGCTGCGGACTGCTCAGTAACAGGCGATTAATTTAATCTTTACAAA-------CGGACAAACTCAGGAAACGGAGTGTAATACGTAAAAGATGATTTTTTATTTAAAAAAAGAAAACATTTTTAGCTTGAATAAAGCGGTAAAGAATAAGACGCCAACCCATCAGTTAGTAAGTAGGGTAAGGGCCTATTTAGACGAATACGGGTACGGGGAATTAGGGTTTGATTCCGGAGAGGGAGCCTGAGAGACGGCTACCAGGTCCAAGGACGGCAGCAGGCGCGAAAATTACCGAAGCTCAAATAGAGGCGGTAGTAATGAGACGTATAAATAATAATATAAGGGTAAAAAACTTATTAATAACTGGAGGTCAAGTCTGGTGCCAGCATCCGCGGTAATACCAGCTCCAGGGGTGTCTATGATGATTGCTGCGATTAAAAGGTCCGTAGTCGAATTTAAATAATTGTTTGTAATATGTTAGATAAAATAATAAAAAGAACAATTACTTAAATGAAAGGAATAACAAGAGGTTGATTAATTGAGTAACGAGAGGTGAAATTTGATGACTTACTTAGGAGAAACAGAGGCGAAAGCGTCAATCAAGTGTAAATCCGATGATCAAGGACGTAGGCTGGAGTATCGAACACGATTAGATACCGTAGTAGTTCCAGCAGTAAACTATGCCTACGCCAATAAATAAAAGTTTAATGGACGAGAAATCTAGAGTAGGGCTTTGGGGAGAGTACACGCGCAAGCGATAAATTTAAAGGAAATTGACGGAAGAACACCACAAGGAGTGGAGTGTGCGGCTTAATTTGACTCAACGCGGGACAGCTTACCAAACCCGAAAACTAAAAGAGTGAATACACGATAAGTTTAAGAGTGGTGCATGGCCGTTATCGACGAGTGAAGTGATTTTATGGTTAAATCCGACAAGTCGTGAGACCCTATAAATAATTAATAAAACAGGTATGAAAATACAGGAAGGATAGGACAAGAACAGGTCAGTGATGCCCTTAAATGGTTTGGGCTGCACGCGCACTACAGTGGTTATAATAAAATATTATAGAAAATTAAATAAAGAAATATAATCAAGAGGGATTGAGTATTGAAAAATACCCATGAACGAGGAATTGCTAGTAATCGTAGGCTCAGTAAGATACGATGAATATGTCCCTGTTCTTTGTACACACCGCCCGTCGTTATCGAAGATGGAGTTTTGCCCGAACGAGCTTTAGCGAGTAAGTGTATGATTCTAGATCTGATACAAGTCGTAACAAGGCAGCTGTAGGAGAACCTGTAGC

>>KR190602_GB_Dhae_Corn_BOJ-15_1186pb_KR190602

GTCTCATAGATTTAGCCATGCATGTGTAAGCGAACAATTAGGGAGCTGCGGACTGCTCAGTAACAGGCGATTAATTTAATCTTTACAAA-------TGGACAAACTCAGGAAACGGAGTGTAATACATAAAAAATGATTTTTTAAAT--AAAAAGAAAACATTTTTAGCTTGAAAAAAGCGGTAAAGAATAAGACGCCAACCCATCAGTTAGTAAGTAGGGTAAGGGCCTATTTAGACGAATACGGGTACGGGGAATTAGGGTTTGATTCCGGAGAGGGAGCCTGAGAGACGGCTACCAGGTCCAAGGACGGCAGCAGGCGCG-AAATTACCGAAGCTCAAATAGAGGCGGTAGTAATGAGACGTATAAAAAATAATATAAGGGTAAAAAACTTATTAATAACTAGAGGTCAAGTCTGGTGCCAGCATCCGCGGTAATACCAGCTCTAGGGGTGTCTATGATGATTGCTGCGATTAAAAGGTCCGTAGTCGAATTTAAATAATTGTTTGTAATATGTTAGATAAAATAATAAAAAGAACAATTACTTAAATGAAAGGAATAACAAGAGGTTGATTAATTGAGTAACGAGAGGTGAAATTTGATGACTTACTTAGGAGAAACAGAGGCGAAAGCGTCAATCAAGTGTAAATCCGATGATCAAGGACGTAGGCTGGAGTATCGAACACGATTAGATACCGTAGTAGTTCCAGCAGTAAACTATGCCTACGCCAATGAATGAAAGTTTGATGGACGAGAAATCTAGAGTAGGGCTTTGGGGAGAGTACACGCGCAAGCGATAAATTTAAAGGAAATTGACGGAAGAACACCACAAGGAGTGGAGTGTGCGGCTTAATTTGACTCAACGCGGGACAGCTTACCAAACCCGAAAACTAAAAGAGTGAATACACGATAAGTTTAAGAGTGGTGCATGGCCGTTATCGACGAGTGAAGTGATTTTATGGTTAAATCCGACAAGTCGTGAGACCCTATAA------AATAAAACAGGTATGAAAATACAGGAAGGATAGGACAAGAACAGGTCAGTGATGCCCTTAGATGGTTTGGGCTGCACGCGCACTACAGTGGTTATAATAAAATA-TATAGAAAACTAAATAAAGAAATATAATCAAGAGGGATTGAGTATTGAAAAATACCCATGAACGAGGAATTGCTAGTAATCGTAGGCTCAGTAAGATACG

>>KR871369_DE_Dhae_Msp-G_GRA-15_960pb_KR871369

TTAGGGAGCTGCGGACTGCTCAGTAACAGGCGATTAATTTAATCTTTACAAA-------TGGACAAACTCAGGAAACGGAGTGTAATACATAAAAAATGATTTTTTAAAT--AAAAAGAAAACATTTTTAGCTTGAAAAAAGCGGTAAAGAATAAGACGCCAACCCATCAGTTAGTAAGTAGGGTAAGGGCCTATTTAGACGAATACGGGTACGGGGAATTAGGGTTTGATTCCGGAGAGGGAGCCTGAGAGACGGCTACCAGGTCCAAGGACGGCAGCAGGCGCGAAAATTACCGAAGCTCAAATAGAGGCGGTAGTAATGAGACGTATAAAAAATAATATAAGGGTAAAAAACTTATTAATAACTAGAGGTCAAGTCTGGTGCCAGCATCCGCGGTAATACCAGCTCTAGGGGTGTCTATGATGATTGCTGCGATTAAAAGGTCCGTAGTCGAATTTAAATAATTGTTTGTAATATGTTAGATAAAATAATAAAAAGAACAATTACTTAAATGAAAGGAATAACAAGAGGTTGATTAATTGAGTAACGAGAGGTGAAATTTGATGACTTACTTAGGAGAAACAGAGGCGAAAGCGTCAATCAAGTGTAAATCCGATGATCAAGGACGTAGGCTGGAGTATCGAACACGATTAGATACCGTAGTAGTTCCAGCAGTAAACTATGCCTACGCCAATGAATGAAAGTTTGATGGACGAGAAATCTAGAGTAGGGCTTTGGGGAGAGTACACGCGCAAGCGATAAATTTAAAGGAAATTGACGGAAGAACACCACAAGGAGTGGAGTGTGCGGCTTAATTTGACTCAACGCGGGACAGCTTACCAAACCCGAAAACTAAAAGAGTGAATACACGATAAGTTTAAGAGTGGTGCATGGCCGTTATCGACGAGTGAAGTGATTTTATGGTTAAATCCGACAAGTCGTGAGACCCTATAA------AATAAAACAGGTAT

>>KR871366_DE_Dhae_Msp-G_GRA-15_940pb_KR871366

TTAGGGAGCTGCGGACTGCTCAGTAACAGGCGATTAATTTAATCTTTACAGAAACGAGCGGAATAAACTCAGGAAACGGAGTGTAATACGTAAAAGATGAATTTTTTATTTTAAGAAGAAAACATTTTTAGCTTGAAAAAAGCGGTAAAGAATAAGACGCCAACCCATCAGTTAGTAAGTAGGGTAAGGGCCTATTTAGACGAATACGGGTACGGGGAATTAGGGTTTGATTCCGGAGAGGGAGCCTGAGAGACGGCTACCAGGTCCAAGGACGGCAGCAGGCGCGAAAATTACCGAAGCTCAAATAGAGGCGGTAGTAATGAGACGTATAAAAAATAATATAAGGGTAAAAAACTTATTAATAACTAGAGGTCAAGTCTGGTGCCAGCATCCGCGGTAATACCAGCTCTAGGGGTGTCTATGATGATTGCTGCGATTAAAAGGTCCGTAGTCGAATTTAAATAATTGTTTGTAATATGTTAGATAAAATAATAAAAAGAACAATTACTTAAATGAAAGGAATAACAAGAGGTTGATTAATTGAGTAACGAGAGGTGAAATTTGATGACTTACTTAGGAGAAACAGAGGCGAAAGCGTCAATCAAGTGTAAATCCGATGATCAAGGACGTAGGCTGGAGTATCGAACACGATTAGATACCGTAGTAGTTCCAGCAGTAAACTATGCCTACGCCAATGAATGAAAGTTTGATGGACGAGAAATCTAGAGTAGGGCTTTGGGGAGAGTACACGCGCAAGCGATAAATTTAAAGGAAATTGACGGAAGAACACCACAAGGAGTGGAGTGTGCGGCTTAATTTGACTCAACGCGGGACAGCTTACCAAACCCGAAAACTAAAAGAGTGAATACACGATAAGTTTAAGAGTGGTGCATGGCCGTTATCGACGAGTGAAGTGATTTTATGGTTAAATCCGACAAGT

>>KR871367_DE_Groe_Msp-G_GRA-15_952pb_KR871367

TTAGGGAGCTGCGGACTGCTCAGTAACAGGCGATTAATTTAATCTTTACAGAAACGAGCGGAATAAACTCAGGAAACGGAGTGTAATACGTAAAAGATGAATTTTTTATTTTAAGAAGAAAACATTTTTAGCTTGAATAAAGCGGTAAAGAATAAGACGCCAACCCATCAGTTAGTAAGTAGGGTAAGGGCCTATTTAGACGAATACGGGTACGGGGAATTAGGGTTTGATTCCGGAGAGGGAGCCTGAGAGACGGCTACCAGGTCCAAGGACGGCAGCAGGCGCGAAAATTACCGAAGCTCAAATAGAGGCGGTAGTAATGAGACGTATAAATAATAATATAAGGGTAAAAAACTTATTAATAACTGGAGGTCAAGTCTGGTGCCAGCATCCGCGGTAATACCAGCTCCAGGGGTGTCTATGATGATTGCTGCGATTAAAAGGTCCGTAGTCGAATTTAAATAATTGTTTGTAATATGTTAGATAAAATAATAAAAAGAACAATTACTTAAATGAAAGGAATAACAAGAGGTTGATTAATTGAGTAACGAGAGGTGAAATTTGATGACTTACTTAGGAGAAACAGAGGCGAAAGCGTCAATCAAGTGTAAATCCGATGATCAAGGACGTAGGCTGGAGTATCGAACACGATTAGATACCGTAGTAGTTCCAGCAGTAAACTATGCCTACGCCAATAAATAAAAGTTTAATGGACGAGAAATCTAGAGTAGGGCTTTGGGGAGAGTACACGCGCAAGCGATAAATTTAAAGGAAATTGACGGAAGAACACCACAAGGAGTGGAGTGTGCGGCTTAATTTGACTCAACGCGGGACAGCTTACCAAACCCGAAAACTAAAAGAGTGAATACACGATAAGTTTAAGAGTGGTGCATGGCCGTTATCGACGAGTGAAGTGATTTTATGGTTAAATCCGACAAGTCGTGAGACCCTA

Figure 4 : *Cucumispora roeselii*

>PL5-10_PL_Groe_Croe_ADR-18_818pb_PL5-10-Croe

TGTAAGCGAACGAGAGGGAGCTGCGGACTGCTCAGTAACAGGCGAATAATTTAATCTTTACAAACGGACAAACTCAGGAAACGGAGTGAAATACGAAAAAGTTGATATTTTTATAAAAGAAAATCGAAACTAGCTTGAAAAAGCGGTAAAGAATAAGACGCCAGCCCATCAGTTAGTAAGTAGGGTAAGGGCCTATTTAGACGAATACGGGTACGGGGAATTAGGGTTTGATTCCGGAGAGGGAGCCTGAGAGACGGCTACCAGGTCCAAGGACGGCAGCAGGCGCGAAAATTACCGAAGCTCGAAATGAGGCGGTAGTAATGAGACGTAAATAAA-TAATACAAGGGTAAAAAACTTGTTAATAACTGGAGGTCAAGTCTGGTGCCAGCATCCGCGGTAATACCAGCTCCAGGGGTGTCTATGATGATTGCTGCGATTAAAAGGTCCGTAGTCGAAAATAGATAATTGTTTGTAAGATGTTATCTAAAATAATAGAAAGAACAATTACCTAATTGAAAGGAATAGCAAGAGGCTGGTTAATCGAGTAACGAGAGGTGAAATTTGATGACTTACTTGGGAGAAACAGAGGCGAAAGCGCCAGTCAAGTGTAAATCCGATGATCAAGGACGTAGGCTGGAGTATCGAACACGATTAGATACCGTAGTAGTTCCAGCAGTAAACTATGCCTACGCCGTTGAATGAAAGTTTGGCGGACGAGAAATTTAGAGTAGGGCTTTGGGGAGAGTACACGCGCAAGCGATAAATTTAAAGGAAATTGACGGAAGAACACCACAAGGAGTGGAGTGTGCGGCTTAATT

>KY200851_PL_Groe_Croe_BOJ-17_823pb_KY200851

GACGGCTACCAGGTCCAAGGACGGCAGCAGGCGCGAAAATTACCGAAGCTCGAAATGAGGCGGTAGTAATGAGACGTAAATAAA-TAATACAAGGGTAAAAAACTTGTTAATAACTGGAGGTCAAGTCTGGTGCCAGCATCCGCGGTAATACCAGCTCCAGGGGTGTCTATGATGATTGCTGCGATTAAAAGGTCCGTAGTCGAAAATAGATAATTGTTTGTAAGATGTTATTTAAAATAATAGAAAGAACAATTACCTAATTGAAAGGAATAGCAAGAGGCTGGTTAATCGAGTAACGAGAGGTGAAATTTGATGACTTACTTGGGAGAAACAGAGGCGAAAGCGCCAGTCAAGTGTAAATCCGATGATCAAGGACGTAGGCTGGAGTATCGAACACGATTAGATACCGTAGTAGTTCCAGCAGTAAACTATGCCTACGCCGTTGAATGAAAGTTTGGCGGACGAGAAATTTAGAGTAGGGCTTTGGGGAGAGTACACGCGCAAGCGATAAATTTAAAGGAAATTGACGGAAGAACACCACAAGGAGTGGAGTGTGCGGCTTAATTTGACTCAACGCGGGACAGCTTACCAAACCCGAGGACCAAAAGAGTGAATACACGATAGGTTTAAGAGTGGTGCATGGCCGTTATCGACGAGTGAAGTGATTTTATGGTTAAATCCGACAAGTCGTGAGACCCT------AAAATAAACAGGTATGAAAATACAGGAAGGATAGGACAAGAACAGGTCAGTGATGCCCTTAGATGGTTTGGGCTGCACGCGCACTACAGTGGTTATAAAAAAAAAGAAAAAAGAAAATAAATAT

>FN434092_GB_Gdub-du_Croe_KRE-10_1249pb_FN434092

GTGTAAGCGAACGAGAGGGAGCTGCGGACTGCTCAGTAACAGGCGAATAATTTAATCTTTACAAACGGACAAACTCAGGAAACGGAGTGAAATACGAAAAAGTTGATATTTTTATAAAAAGAAATCGAACTTAGCTTGAAAAAGCGGTAAAGAATAAGACGCCAGCCCATCAGTTAGTAAGTAAGGTAAGGGCTTATTTAGACGAATACGGGTACGGGGAATTAGGGTTTGATTCCGGAGAGGGAGCCTGAGAGACGGCTACCAGGTCCAAGGACGGCAGCAGGCGCGAAAATTACCGAAGCTCAAAAAGAGGCGGTAGTAATGAGACGTAAAAAAATTAATACAAGGGTAAAAAACTTGTTAATAACTGGAGGTAAAGTCTGGTGCCAGCATCCGCGGTAATACCAGCTCCAGGGGTGTCTATGATGATTGCTGCGATTAAAAGGTCCGTAGTCGAAAATAGATAATTGTTTGTAAGATGTTATCTAAAATAATAGAAAGAACAATTACCTAATTGAAAGGAATAGCAAGAGGCTGATTAATCGAGTAACGAGAGGTGAAATTTGATGACTTACTTGGGAGAAACAGAGGCGAAAGCGTCAGTCAAGTGTAAATCCGATGATCAAGGACGTAGGCTGGAGTATCGAACACGATTAGATACCGTAGTAGTTCCAGCAGTAAACTATGCCTACGCCGTTGAATGAAAGTTTGGCGGACGAGAAATTTAGAGTAGGGCTTTGGGGAGAGTACACGCGCAAGCGATAAATTTAAAGGAAATTGACGGAAGAACACCACAAGGAGTGGAGTGTGCGGCTTAATTTGACTCAACGCGGGACAGCTTACCAAACCCGAGGACCAAAAGAGTGAATACACGATAGGTTTAAAAGTGGTGCATGGCCGTTATCGACGAGTGAAGTGATTTTATGGTTAAATCCGACAAGTCGTGAGACCCTAAAAAAAAAATTAACAGGTATGAAAATACAGGAAGGATAGGACAAGAACAGGTCAGTGATGCCCTTAGATGGTTTGGGCTGCACGCGCACTACAGTGGTTATAAAAAAAAAGAAAAAAGAAAATAAATATAATCAAGAGGAATTGAGCACTGAAAAGTGCCCATGAACGAGGAATTGCTAGTAATCGTAGGCTCAGTAAGATACGATGAATATGTCCCTGTTCTTTGTACACACCGCCCGTCGTTATCGAAGATGGAGTTTTGCCCGAACGAGCTTTAGCGAGTAAGTGTATGATT

>KX137904_DE_Ephemera_danica_Msp-groupD_GRA-17_393pb_KX137904

GTGTAAGCGAACGAGAGGGAGCTGCGGACTGCTCAGTAACAGGCGAATAATTTAATCTTTACAAACGGACAAACTCAGGAAACGGAGTGAAATACGAAAAAGTTGATATTTTTATAAAAAGAAATCGAACTTAGCTTGAAAAAGCGGTAAAGAATAAGACGCCAGCCCATCAGTTAGTAAGTAAGGTAAGGGCTTATTTAGACGAATACGGGTACGGGGAATTAGGGTTTGATTCCGGAGAGGGAGCCTGAGAGACGGCTACCAGGTCCAAGGACGGCAGCAGGCGCGAAAATTACCGAAGCTCAAAAAGAGGCGGTAGTAATGAGACGTAAAAAAATTAATACAAGGGTAAAAAACTTGTTAATAACTGGAGGTAAAGTCTGGTGCCAGCAC

Figure 4 : *Cucumispora dikerogammari*

>GRR-15_FR_Groe_Cdik_ADR-18_385pb_GRR-15-Cdik

CTGCGGACTGCTCAGTAACAGACATATAATTTAATCTTTACAGAAACGAGCGGAATAAACTCAGGAAACAGAGTGCAATACGTAAAAGACGAATTTTTTATTATAAGAAATACGTTTTTAGCTTGAACAAAGCGGTAAAGAATAAGTTGTCAGCCTATCAGTTAGTAAGTAGGGTAAGGGCCTATTTAGACGAAGACGGGTACGGGGAATTAGAGTTTGATTCCGGAGAGGGAGCCTGAGAAATAGCTACCAGGTCCAAGGACGGCAGCAGGCGCGAAAATTACCGAAGCTCGAATAGAGGCGGTAGTAATGAGACGTATTAATATAAAACAAGGGTAAAAAACTTGTTAGTAACTGGAGGTCAAGTCTGGTGCCAGCAGCCGCG

>GQ258752_PL_Dvil_Cdik_OVC-10_1291pb_GQ258752

CATGTGTAAGCGAACAAGAGGAAGCTGCGGACTGCTCAGTAACAGACATATAATTTAATCTTTACAGAAACGAGCGGAATAAACTCAGGAAACAGAGTGCAATACGTAAAAGACGAATTTTTTATTATAAGAAATACGTTTTTAGCTTGAACAAAGCGGTAAAGAATAAGTTGTCAGCCTATCAGTTAGTAAGTAGGGTAAGGGCCTATTTAGACGAAGACGGGTACGGGGAATTAGAGTTTGATTCCGGAGAGGGAGCCTGAGAAATAGCTACCAGGTCCAAGGACGGCAGCAGGCGCGAAAATTACCGAAGCTCGAATAGAGGCGGTAGTAATGAGACGTATTAATATAAAACAAGGGTAAAAAACTTGTTAGTAACTGGAGGTCAAGTCTGGTGCCAGCATCCGCGGTAATACCAGCTCCAGGGGTGTCTATGATGATTGCTGCGATTAAAAGGTCCGTAGTCGAATTTATATAATTGTTTGTAATATGCTAGATAAAATAACAGAAAGAACAATTACTTTAAATGAAAGGAATAGTAAGGGGCTGATTAATTGAGCAACGAGAGGTGAAATTTGATGACTTGCTTAGGAGAAACAGAGGCGAAAGCGTCAGTCAAGTATAAATCCTATGATCAAGGACGTAGGCTAGAGTATCGAACACGATTAGATACCGTAGTAGTTCTAGCAGTAAACTATGCCTACACTATCGAATAAAAGTTTGGTAGAAGAGAAATCTTAAGTAGGGCTTTGGGGAGAGTACACGCGCAAGCGATAAATTTAAAGGAAATTGACGGAAGAACACCACAAGGAGTGGAGTGTGCGGCTTAATTTGACTCAACGCGGGACAGCTTACCATACCCGAGGACTATAAGAGTGAATACACGATAAGTCTAAAAGTGGTGCATGGCCGTTATCGACGAGTGAAGTGATTTTATGGTTAAATCCGACAAGTTGTGAGACCCTTATTTAAATACAGGTATTGTTAAAATACAGGAAGGAAAGGACAAGAACAGGTCAGTGATGCCCTTAGATGGTATGGGCTGCACGCGCACTACAATGGTTATAATAATAAAGATAATTAAAGTATAAATATAATCAAGAGGAATTGAGAACTGAAAAGTTCCCATGAACGAGGAATTGCTAGTAATCGTAGGCTCAGTAAGATACGATGAATATGTCCCTGTTCTTTGTACACACCGCCCGTCGTTATCGAAGATGGAGTTTTACCCGAACAAGCTTAAGCGAGTGAGTGTATGATTCTAGATCTGATACAAGTCGTAACAAGGCAG

Figure 5 *: Dictyocoela roeselii*

>LOQ-07_FR_Groe_Droe-1_ADR-18_818pb_LOQ-07

CATGTGTAAGCGAAGCGATAAGTGGAGCGGTGAAAGGCTCAGTAACGGGCGATTTATTTGATCTCCTGGGACGGACAACATCGGGAAACTGATGGGAAAACGTCTAAGTTGCAGTTTTTTTTTGATTGCGACGTAAACCTTTTGGTGCAGGAGAGTAAGATGCCCTCCTATCAGTTAGTAAGTAGGGTAGGGGCCTACTTAGACGAAGACGGGTACGGGGAATGAGGGTTTGATTCCGGAGAGGGAGCCTGAGAGATGGCTACCAGGACCAAGGTCAGCAGCAGGCGCGAAAATTATCGAACCCCGCCTAGGGGCGATAGTGAGGAGACGTGTATAACGAAGTACGTGTAAAGACCGTACTAATAACTGGAGGTCAAGTCTGGTGCCAGCATCCGCGGTAATTCCAGCTCCAGGGGTGTCTATGATGATTGCTGCGATTAAAAAGTCCGTAGTCGAGCTGACTGACTGACCTGCAATGTGGTTGATTAAAAGACGAGCAGGGTTAGGAAAGCAGAGAATTAGGAGCGACCGAGGGCTAGAGTATTGAATGGCGAGAGGTGAAATTTGATGACCCATTCAGGAGTGACAAAGGCGAAGGCACTAGTCAAGGGCGAATCCGATGATCAAGGACGTAGGCTAGAGTTTCGAAAACGATTAGAGACCGGAGTAGTTCTAGCAGTAAACTATGCCGACACCGTGGTATTAATTTTTTAGTATTGCGGAAGAGAAATCAAGTAAGGCTTTGGGGAGAGTACGCGCGCAAGCGATAAATTTAAAGGAAATTGACGGAGGAACACCACAAGGAGTGGAGTGTGCGG

>VOL5.4-07_FR_Groe_Droe-2_ADR-18_818pb_VOL5.4-07

CATGTGTAAGCGAAGCGATAAGTGGAGCGGTGAAAGGCTCAGTAACGGGCGATTTATTTGATCTCCTGGGACGGACAACATCGGGAAACTGATGGGAAAACGTCTAAGTTGCAGTTTTTTTTTGATTGCGACGTAAACCTTTTGGTGCAGGAGAGTAAGATGCCCTCCTATCAGTTAGTAAGTAGGGTAAGGGCCTACTTAGACGAAGACGGGTACGGGGAATGAGGGTTTGATTCCGGAGAGGGAGCCTGAGAGATGGCTACCAGGACCAAGGTCAGCAGCAGGCGCGAAAATTATCGAACCCCGCCTAGGGGCGATAGTGAGGAGACGTGTATAACGAAGTACGTGTAAAGACCGTACTAATAACTGGAGGTCAAGTCTGGTGCCAGCATCCGCGGTAATTCCAGCTCCAGGGGTGTCTATGATGATTGCTGCGATTAAAAAGTCCGTAGTCGAGCTGACTGACTGACCTGCAATGTGGTTGATTAAAAGACGAGCAGGGTTAGGAAAGCAGAGAATTAGGAGCGACCGAGGGCTAGAGTATTGAATGGCGAGAGGTGAAATTTGATGACCCATTCAGGAGTGACAAAGGCGAAGGCACTAGTCAAGGGCGAATCCGATGATCAAGGACGTAGGCTAGAGTTTCGAAAACGATTAGAGACCGGAGTAGTTCTAGCAGTAAACTATGCCGACACCGTGGTATTAATTTTTTAGTATTGCGGAAGAGAAATCAAGTAAGGCTTTGGGGAGAGTACGCGCGCAAGCGATAAATTTAAAGGAAATTGACGGAGGAACACCACAAGGAGTGGAGTGTGCGG

>MAR6-4-01_FR_Groe_Droe-3_ADR-18_823pb_MAR6-4-01

CATGTGTAAGCGAAGCGATAAGTGGAGCGGTGAAAGGCTCAGTAACGGGCGATTTATTTGATCTCCTGGGACGGACAACATCGGGAAACTGATGGGAAAACGTCTAAGTTGCAGTTTTTTTTTGATTGCGACGTAAACCTTTTGGTGCAGGAGAGTAAGATGCCATCCTATCAGTTAGTAAGTAGGGTAGGGGCCTACTTAGACGAAGACGGGTACGGGGAATGAGGGTTTGATTCCGGAGAGGGAGCCTGAGAGATGGCTACCAGGACCAAGGTCAGCAGCAGGCGCGAAAATTATCGAACCCCGCCTAGGGGCGATAGTGAGGAGACGTGTATAACGAAGTACGTGTAAAGACCGTACTAATAACTGGAGGTCAAGTCTGGTGCCAGCATCCGCGGTAATTCCAGCTCCAGGGGTGTCTATGATGATTGCTGCGATTAAAAAGTCCGTAGTCGAGCTGACTGACTGACCTGCAATGTGGTTGATTAAAAGACGAGCAGGGTTAGGAAAGCAGAGAATTAGGAGCGACCGAGGGCTAGAGTATTGAATGGCGAGAGGTGAAATTTGATGACCCATTCAGGAGTGACAAAGGCGAAGGCACTAGTCAAGGGCGAATCCGATGATCAAGGACGTAGGCTAGAGTTTCGAAAACGATTAGAGACCGGAGTAGTTCTAGCAGTAAACTATGCCGACACCGTGGTATTAATTTTTTAGTATTGCGGAAGAGAAATCAAGTAAGGCTTTGGGGAGAGTACGCGCGCAAGCGATAAATTTAAAGGAAATTGACGGAGGAACACCACAAGGAGTGGAGTGTGCGGCTTAA

>LOQ-08_FR_Groe_Droe-4_ADR-18_801pb_LOQ-08

CATGTGTAAGCGAAGCGATAAGTGGAGCGGTGAAAGGCTCAGTAACGGGCGATTTATTTGATCTCCTGGGACGGACAACATCGGGAAACTGATGGGAAAACGTCTAAGTTGCAGTTTTTTTTTGATTGCGACGTAAACCTTTTGGTGCAGGAGAGTAAGATGCCATCCTATCAGTTAGTAAGTAGGGTAAGGGCCTACTTAGACGAAGACGGGTACGGGGAATGAGGGTTTGATTCCGGAGAGGGAGCCTGAGAGATGGCTACCAGGACCAAGGTCAGCAGCAGGCGCGAAAATTATCGAAGCCCGCCTAGGGGCGATAGTGAGGAGACGTGTATAACGAAGTACGTGTAAAGACCGTACTAATAACTGGAGGTCAAGTCTGGTGCCAGCAGCCGCGGTAATTCCAGCTCCAGGGGTGTCTATGATGATTGCTGCGATTAAAAAGTCCGTAGTCGAGCTGACTGACTGACCTGCAATGTGGTTGATTAAAAGACGAGCAGGGTTAGGAAAGCAGAGAATTAGGAGCGACCGAGGGCTAGAGTATTGAATGGCGAGAGGTGAAATTTGATGACCCATTCAGGAGTGACAAAGGCGAAGGCACTAGTCAAGGGCGAATCCGATGATCAAGGACGTAGGCTAGAGTTTCGAAAACGATTAGAGACCGGAGTAGTTCTAGCAGTAAACTATGCCGACACCGTGGTATTAATTTTTTAGTATTGCGGAAGAGAAATCAAGTAAGGCTTTGGGGAGAGTACGCGCGCAAGCGATAAATTTAAAGGAAATTGACGGAGGAACACCACA

>LOQ-05_FR_Groe_Droe-5_ADR-18_826pb_LOQ-05

CATGTGTAAGCGAAGCGATAAGTGGAGCGGTGAAAGGCTCAGTAACGGGCGATTTATTTGATCTCCTGGGACGGACAACATCGGGAAACTGATGGGAAAACGTCTAAGTTGCAGTTTTTTTTTGATTGCGACGTAAACCTTTTGGTGCAGGAGAGTAAGATGCCATCCTATCAGTTAGTAAGTAGGGTAAGGGCCTACTTAGACGAAGACGGGTACGGGGAATGAGGGTTTGATTCCGGAGAGGGAGCCTGAGAGATGGCTACCAGGACCAAGGTCAGCAGCAGGCGCGAAAATTATCGAAGCCCGCCTAGGGGCGATAGTGAGGAGACGTGTATAACGAAGTACGTGTAAAGACCGTACTAATAACTGGAGGTCAAGTCTGGTGCCAGCATCCGCGGTAATTCCAGCTCCAGGGGTGTCTATGATGATTGCTGCGATTAAAAAGTCCGTAGTCGAGCTGACTGACTGACCTGCAATGTGGTTGATTAAAAGACGAGCAGGGTTAGGAAAGCAGAGAATTAGGAGCGACCGAGGGCTAGAGTATTGAATGGCGAGAGGTGAAATTTGATGACCCATTCAGGAGTGACAAAGGCGAAGGCACTAGTCAAGGGCGAATCCGATGATCAAGGACGTAGGCTAGAGTTTCGAAAACGATTAGAGACCGGAGTAGTTCTAGCAGTAAACTATGCCGACACCGTGGTATTAATTTTTTAGTATTGCGGAAGAGAAATCAAGTAAGGCTTTGGGGAGAGTACGCGCGCAAGCGATAAATTTAAAGGAAATTGACGGAGGAACACCACAAGGAGTGGAGTGTGCGGCTTAATTT

>KR871360_DE_Groe_Ddue_GRA-15_548pb_KR871360

GTAAGATGCCATCCTATCAGTTAGTAAGTAGGGTAAGGGCCTACTTAGACGAAGACGGGTACGGGGAATGAGGGTTTGATTCCGGAGAGGGAGCCTGAGAGATGGCTACCAGGACCAAGGTCAGCAGCAGGCGCGAAAATTATCGAAGCCCGCCTAGGGGCGATAGTGAGGAGACGTGTATAACGAAGTACGTGTAAAGACCGTACTAATAACTGGAGGTCAAGTCTGGTGCCAGCATCCGCGGTAATTCCAGCTCCAGGGGTGTCTATGATGATTGCTGCGATTAAAAAGTCCGTAGTCGAGCTGACTGACTGACCTGCAGTGTGGTTGATTAAAAGACGAGCAGGGTTAGGAAAGCAGAGAATTAAGAGCGACCGAGGGCTAGAGTATTGAATGGCGAGAGGTGAAATTTGATGACCCATTCAAGAGTGACAAAGGCGAAGGCACTAGTCAAGGGCGAATCCGATGATCAAGGACGTACGCTAGAGTTTCGAAAACGATTAGAGACCGGAGTAGTTCTAGCAGTAAACTATGCCGACACCGTGGTA

>R5_GRR24_FR_Groe_Droe_KAR-18_1759pb_MG773219*

AGCGATAAGTGGAGCGGTGAAAGGCTCAGTAACGGGCGATTTATTTGATCTCCTGGGACGGACAACATCGGGAAACTGATGGGAAAACGTCTAAGTTGCAGTTTTTTTTTGATTGCGACGTAAACCTTTTGGTGCAGGAGAGTAAGATGCCATCCTATCAGTTAGTAAGTAGGGTAAGGGCCTACTTAGACGAAGACGGGTACGGGGAATGAGGGTTTGATTCCGGAGAGGGAGCCTGAGAGATGGCTACCAGGACCAAGGTCAGCAGCAGGCGCGAAAATTATCGAAGCCCGCCTAGGGGCGATAGTGAGGAGACGTGTAT-ACGAAGTACGTGTAAAGACCGTACTAATAACTGGAGGTCAAGTCTGGTGCCAGCATCCGCGGTAATTCCAGCTCCAGGGGTGTCTATGATGATTGCTGCGATTAAAAAGTCCGTAGTCGAGCTGACTGACTGACCTGCAATGTGGTTGATTAAAAGACGAGCAGGGTTAGGAAAGCAGAGAATTAGGAGCGACCGAGGGCTAGAGTATTGAATGGCGAGAGGTGAAATTTGATGACCCATTCAGGAGTGACAAAGGCGAAGGCACTAGTCAAGGGCGAATCCGATGATCAAGGACGTAGGCTAGAGTTTCGAAAACGATTAGAGACCGGAGTAGTTCTAGCAGTAAACTATGCCGACACCGTGGTATTAATTTTTTAGTATTGCGGAAGAGAAATCAAGTAAGGCTTTGGGGAGAGTACGCGCGCAAGCGATAAATTTAAAGGAAATTGACGGAGGAACACCACAAGGAGTGGAGTGTGCGGCTTAATTTGACTCAACGCGGGACAGCTTACCAGGCCCGATAATCGAGCGAGCGTAGTACGCGATAGATTAAAAAGTGGTGCATGGCTGCTATCGACAGTTGGGGTGACCTTAGGGTTAATTCCGGCAAGTAGTGAGACCCCTGCAGATAGTGGACAGGTATTTTTAAGATACAGGAAGGAAGGGACAAGAGCAGGTCAGTGATGCCCTTAGATGGCCTGGGCTGCACGCGCACTACAGTGGTCATTATAAGTAGAAATTAGATATAAAGATGATCGAGAGGGACTGAGCTTTGTAAGAGGCTCACGAACGAGGAATTGCTAGTAATCGTAGGCTCATTAAGATACGATGAATATGTCCCTGTACCTTGTACACACCGCCCGTCGTTATCGAAGATGGAATTGTGTGCGAACGAGCATTAAGCGAGTGAGCGCATAGTTCTAGATGTGATAAAAGTCGTAACAAGGCAACTGTAGGAGAACCTGTAGTTGGATCATACAGATTGTTTAAT-AGAAAGTT--TTTAATTTAATTCCTTACGCAAGGGATCGTTTGGTTCTATGTACGATGAAGGTCGACACTGTATTCGATAAAATAAGATGAAAGTAAAAATTGTCTTATATTACTGAATAGAGTAGCGATGCTCGAATACTCCTTTGAATTAAGCATATGAGTAAAGGAAGGAGAAGAAACTAACAAGGATTCTCTTATTAGTGGCGAATGAACAGAGAATAGCCCTAAGTGTAATCAAATTCTAGCAATTTGAGATGTCAGGCATTGAATAGTGTGAATATACTGGAAAGTATAGCCAAAGAGAGTTATAGCCTCGTAACACTATATGATAGGGATGAGTAGTTGTGCTCGGTAATGCACAATGAAATAGGTGGTAGTGTCCATCTAAGGCTAAATATGACATAGAGACCGATAGTGAATAAGTAAAGTGATCGAAAATGGAATAGA

>KR871359_DE_Gpul_Ddue_GRA-15_1054pb_KR871359

GTGGAGCGGTGAAAGGCTCAGTAACGGGCGATTTATTTGATCTCCTGGGACGGACAACATCGGGAAACTGATGGGAAAACGTCTAAGTTGCAG---TTTATTGATTGTGACGTAAACCTATGTGTGCAGGAGAGTAAGATGCCATCCTATCAGTTAGTAAGTAGGGTAAGGGCCTACTTAGACGAAGACGGGTACGGGGAATGAGGGTTTGATTCCGGAGAGGGAGCCTGAGAGATGGCTACCAGGACCAAGGTCAGCAGCAGGCGCGAAAATTATCGAAGCCCGCCCAGGGGCGATAGTGAGGAGACGTGTATAACGAAGTACGTGTAAAGACCGTACTAATAACTGGAGGTCAAGTCTGGTGCCAGCATCCGCGGTAATTCCAGCTCCAGGGGTGTCTATGATGATTGCTGCGATTAAAAAGTCCGTAGTCGAGCTGACTGACTGACCTGCAATGTGATTGATTAAAAGACGAGCAGGGTTAGGAAAGCAGAGAATTAGGAGCGACCGAGGGCTAGAGTATTGAATGGCGAGAGGTGAAATTTGATGACCCATTCAGGAGTGACAAAGGCGAAGGCACTAGTCAAGGGCGAATCCGATGATCAAGGACGTAGGCTAGAGTTTCGAAAACGATTAGAGACCGGAGTAGTTCTAGCAGTAAACTATGCCGACACCGTGGTATTAA-ATTTTAGTATTGCGGAAGAGAAATCAAGTAAGGCTTTGGGGAGAGTACGCGCGCAAGCGATAAATTTAAAGGAAATTGACGGAGGAACACCACAAGGAGTGGAGTGTGCGGCTTAATTTGACTCAACGCGGGACAGCTTACCAGGCCCGATAATCGAGCGAGCGTAGTACGCGATAGGTTAAAGAGTGGTGCATGGCTGCTATCGACAGTTGGGGTGACCTTAGGGTTAATTCCGGCAAGTAGTGAGACCCCTGCAGATAGTGGACAGGTATTTTTAAGATACAGGAAGGAAGGGACAAGAGCAGGTCAGTGATGCCCTTAGATGGCCTGGGCTGCACGCGCACTACAGTGGTCATTATAAGTAGAAAGTAG

>R6_GbOv2_PL_Gbal_Droe_KAR-18_1755pb_MG773220

AGCGATAAGTGGAGCGGTGAAAGGCTCAGTAACGGGCGATTTATTTGATCTCCTGGGACGGACAACATCGGGAAACTGATGGGAAAACGTCTAAGTTGCAG---TTGTTTTATTGTGACGTAAACCTTTGTGTGCAGGAGAGTAAGATGCCATCCTATCAGTTAGTAAGTAGGGTAAGGGCCTACTTAGACGAAGACGGGTACGGGGAATGAGGGTTTGATTCCGGAGAGGGAGCCTGAGAGATGGCTACCAGGACCAAGGTCAGCAGCAGGCGCGAAAATTATCGAAGCCCGCCTAGGGGCGATAGTGAGGAGACGTGTAT-ACGAAGTACGTGTAAAGACCGTACTAATAACTGGAGGTCAAGTCTGGTGCCAGCATCCGCGGTAATTCCAGCTCCAGGGGTGTCTATGATGATTGCTGCGATTAAAAAGTCCGTAGTCGAGCTGACTGACTGACCTGCAATGTGATTGATTAAAGAACGAGCAGGGTTAGGAAAGCAGAGGATTAGGAGCGACCGAGGGCTAGAGTATTGAATGGCGAGAGGTGAAATTTGATGACCCATTCAGGAGTGACAAAGGCGAAGGCACTAGTCAAGGGCGAATCCGATGATCAAGGACGTAGGCTAGAGTTTCGAAAACGATTAGAGACCGGAGTAGTTCTAGCAGTAAACTATGCCGACACCGTGGTATTAA--TTTTAGTATTGCGGAAGAGAAATCAAGTAAGGCTTTGGGGAGAGTACGCGCGCAAGCGATAAATTTAAAGGAAATTGACGGAGGAACACCACAAGGAGTGGAGTGTGCGGCTTAATTTGACTCAACGCGGGACAGCTTACCAGGCCCGATAATCGAGCGAGCGTAGTACGCGATAGGTTAAAAAGTGGTGCATGGCTGCTATCGACAGTTGGGGTGACCTTAGGGTTAATTCCGGCAAGTAGTGAGACCCCTGCAGATAGTGGACAGGTATTTTTAAGATACAGGAAGGAAGGGACAAGAGCAGGTCAGTGATGCCCTTAGATGGCCTGGGCTGCACGCGCACTACAGTGGTCATTATAAGTAGAAGTTAGATATAAAGATGATCGAGAGGGACTGAGCTTTGTAAGAGGCTCACGAACGAGGAATTGCTAGTAATCGTAGGCTCATTAAGATACGATGAATATGTCCCTGTACCTTGTACACACCGCCCGTCGTTATCGAAGATGGAATTGTGTGCGAACGAGCATTAAGCGAGTGAGCGCATAGTTCTAGATGTGATAAAAGTCGTAACAAGGCAACTGTAGGAGAACCTGTAGTTGGATCATACAGATTGTTTATTTAAAAAGTT--TTAATTCTAATTCCTTACGCAAGGGATCGTTTGGTTCTATGTACGATGAAGGTCGAAACTGTATTCGATAAAATAGGATGAACGTAAAAAATGTCCTATATTACTGAATAGAGTAGCGATGCTCGAATACTCCTTTGAATTAAGCATATGAGTAAAGGAAGGAGAAGAAACTAACAAGGATTCTCTTATTAGTGGCGAATGAACAGAGAACAGCCCTAAGTGTAATCAAATTCATGTAATTTGAGATGTCAGGCATTGAATAGTGTGAATATACTGGAAAGTATAGCCCAAGAGAGTTATAGCCTCGTAGCACTATATGATAGGGATGAGTAGTTGTGCTCGGTAATGCACAATGAAATAGGTGGTAGTGTCCATCTAAGGCTAAATATGACATAGAAACCGATAGTGGAAAAGTAAAGTGATCGAAAATGGAATAGA

>R1_GFOS_GfOv_PL_Gfos_Droe_KAR-18_1751pb_MG773215

AGCGATATGTGGAGCGGTGAAAGGCTCAGTAACGGGCGATTTATTTGATCTCCTGGGACGGACAACATCGGGAAACTGATGGGAAAACGTCTAAGTTGCAG---TTTTCTGATTGCGACGTAAACCATAGTGTGCAGGAGAGTAAGATGCCATCCTATCAGTTAGTAAGTAGGGTAAGGGCCTACTTAGACGAAGACGGGTACGGGGAATGAGGGTTTGATTCCGGAGAGGGAGCCTGAGAGATGGCTACCAGGACCAAGGTCAGCAGCAGGCGCGAAAATTATCGAAGCCCGCCTAGGGGCGATAGTGAGGAGACGTGTAT-ACGAAGTACGTGTAAAGACCGTACTAATAACTGGAGGTCAAGTCTGGTGCCAGCATCCGCGGTAATTCCAGCTCCAGGGGTGTCTATGATGATTGCTGCGATTAAAAAGTCCGTAGTCGAGCTGACTGACTGACCTGCAATGTGATTGATTAAAGAACGAGCAGGGTTAGGAAAGCAGAGAATTAGGAGCGACCGAGGGCTAGAGTATTGAATGGCGAGAGGTGAAATTTGATGACCCATTCAGGAGTGACAAAGGCGAAGGCACTAGTCAAGGGCGAATCCGATGATCAAGGACGTAGGCTAGAGTTTCGAAAACGATTAGAGACCGGAGTAGTTCTAGCAGTAAACTATGCCGACGCCGTGGTATT----TTTTAGTATTGCGGAAGAGAAATCAAGTAAGGCTTTGGGGAGAGTACGCGCGCAAGCGATAAATTTAAAGGAAATTGACGGAGGAACACCACAAGGAGTGGAGTGTGCGGCTTAATTTGACTCAACGCGGGACAGCTTACCAGGCCCGATAATCGAGCGAGCGTAGTACGCGATAGGTTAAAAAGTGGTGCATGGCTGCTATCGACAGTTGGGGTGACCTTAGGGTTAATTCCGGCAAGTAGTGAGACCCCTGCAGATAGTGGACAGGTATTTTTAAGATACAGGAAGGAAGGGACAAGAGCAGGTCAGTGATGCCCTTAGATGGCCTGGGCTGCACGCGCACTACAGTGGTCATTATAAGTAGAAGTTAGATATAAAGATGATCGAGAGGGACTGAGCTTTGTAAGAGGCTCACGAACGAGGAATTGCTAGTAATCGTAGGCTCATTAAGATACGATGAATATGTCCCTGTACCTTGTACACACCGCCCGTCGTTATCGAAGATGGAATTGTGTGCGAACGAGCATTAAGCGAGTGAGCGCATAGTTCTAGATGTGATAAAAGTCGTAACAAGGCAACTGTAGGAGAACCTGTAGTTGGATCATACAGATTGTTTATC-ATAGTTTT--TTTAGTAT-ATTCCTTACGCAAGGGATCGTTTGGTTCTATGTACGATGAAGGTCGAAACTGTATTCGATAAAATAGGATGAAAGTAAAAATTGTCCTATATTACTGAATAGAGTAGCGATGCTCGAGTACTCCTTTGAATTAAGCATATGAGTAAAGGAAGGAGAAGAAACTAACAAGGATTCTCTTATTAGTGGCGAATGAACAGAGAAGAGCCCTAAGTGTAATCAAATTCTTGTAATTTGAGATGTCAGGCATTAGATAGTGTGAATATACTGGAAAGTATAGTCGTAGAGAGTTATAGCCTCGTAGCACTATATGATAGGGATGAGTAGTTGTGCTCGGTAATGCACAATGAAATAGGTGGTAGTGTCCATCTAAGGCTAAATATGACATAGAAACCGATAGTGAATAAGTAAAGTGATCGAAAATGGAATAGA

>R3_GvarM41_HU_Gvar_Droe_KAR-18_1756pb_MG773217

AGCGATATGTGGAGCGGTGAAAGGCTCAGTAACGGGCGATTTATTTGATCTCCTGGGACGGACAACATCGGGAAACTGATGGGAAAACGTCTAAGTTGCAG---TTTTTTGACTGTGACGTAAACCGTAGTGTGCAGGAGAGTAAGATGCCATCCTATCAGTTAGTAAGTAGGGTAAGGGCCTACTTAGACGAAGACGGGTACGGGGAATGAGGGTTTGATTCCGGAGAGGGAGCCTGAGAGATGGCTACCAGGACCAAGGTCAGCAGCAGGCGCGAAAATTATCGAAGCCCGCCTAGGGGCGATAGTGAGGAGACGTGTTA-ACGAAGTACGTGTAAAGACCGTACTAATAACTGGAGGTCAAGTCTGGTGCCAGCATCCGCGGTAATTCCAGCTCCAGGGGTGTCTATGATGATTGCTGCGATTAAAAAGTCCGTAGTCGAGCTGACTGACTGACCTGCAATGTGATTGATTAAAGAACGAGCAGGGTTAGGAAAGCAGAGGATTAGGAGCGACCGAGGGCTAGAGTATTGAATGGCGAGAGGTGAAATTTGATGACCCATTCAGGAGTGACAAAGGCGAAGGCACTAGTCAAGGGCGAATCCGATGATCAAGGACGTAGGCTAGAGTTTCGAAAACGATTAGAGACCGGAGTAGTTCTAGCAGTAAACTATGCCGACGCCGTGGTATTAG-GTATTAGTATTGCGGAAGAGAAATCAAGTAAGGCTTTGGGGAGAGTACGCGCGCAAGCGATAAATTTAAAGGAAATTGACGGAGGAACACCACAAGGAGTGGAGTGTGCGGCTTAATTTGACTCAACGCGGGACAGCTTACCAGGCCCGAT-ATCGAGCGAGCGTAGTACGCGATAGGTTAAGAAGTGGTGCATGGCTGCTATCGACAGTTGGGGTGACCTTAGGGTTAATTCCGGCAAGTAGTGAGACCCCTGCAGATAGTGGACAGGTATTTTCAAGATACAGGAAGGAAGGGACAAGAGCAGGTCAGTGATGCCCTTAGATGGCCTGGGCTGCACGCGCACTACAGTGGTCATTATAAGTAGAAGTTAGATATAAAGATGATCGAGAGGGACTGAGCTTTGTAAGAGGCTCACGAACGAGGAATTGCTAGTAATCGTAGGCTCATTAAGATACGATGAATATGTCCCTGTACCTTGTACACACCGCCCGTCGTTATCGAAGATGGAATTGTGTGCGAACAAGCATTAAGCGAGTGAGCGCATAGTTCTAGATGTGATAAAAGTCGTAACAAGGCAACTGTAGGAGAACCTGTAGTTGGATCATACAGATTGTTTTTT-AACAGTTTTATTTATAAATATTCCTTACGCAAGGGATCGTTTGGTTCTATGTACGATGAAGGTCGAAACTGTATTCGATAAAATAGGATGAAAGTAAAAATTGTCCTATGTTACTGAATAGAGTAGCGATGCTCGAGTACTCCTTTGAATTAAGCATATGAGTAAAGGAAGGAGAAGAAACTAACAAGGATTCTCTTATTAGTGGCGAATGAACAGAGAAGAGCCCTAAGTGTAATCAAATTCTTGCAATTTGAGATGTCAGGCATTAGATAGTGTGAATATACTGGAAAGTATTGCCGTAGAGAGTTATAGCCTCGTAGCACTATATGATAGGGATGAGTAGTTGTGCTCGGTAATGCACAATGAAATAGGTGGTAGTGTCCATCTAAGGCTAAATATGACATAGAAACCGATAGTGGATAAGTAAAGTGATCGAAAATGGAATAGA

Figure 5 *: Dictyocoela muelleri*

>ALP40-04_DE_Groe_Dmue_ADR-18_822pb_ALP40-04

CATGTGTAAGCGAAGCTATATGTGGAGCGGTGAAAGGCTCAGTAACGGGCGATTTATTTAATCTCCTGGGGCGGACAACATCGGGAAACTGATGGGAAAACGTCTAAGTTGCATTAAT----TTTAGTGTGACGTAAACGATTATCGTGCAGGAGAGTAAGATGCCATCCTATCAGTTAGTAAGTAGGGTAAGGGCCTACTTAGACGAAGACGGGTACGGGGAATGAGGGTTTGATTCCGGAGAGGGAGCCTGAGAGATGGCTACCAGGACCAAGGTCAGCAGCAGGCGCGAAAATTATCGAAGCCCGCCTAGGGGCGATAGTGAGGAGACGTGTATAACGAAGTACGTGTAAAGAACGTACTAATAACTGGAGGTCAAGTCTGGTGCCAGCATCCGCGGTAATTCCAGCTCCAGGGGTGTCTATGATGATTGCTGCGATTAAAAAGTCCGTAGTCAAGCTGCCTGACTGACCTGCAATGTGATTGATTAAGGAACGAGCAGGGTTAGGAAAGCAGAGAATTAGGAGCGACCGAGGGCTAGAGTATTGAATGGCGAGAGGTGAAATTTGATGACCCATTCAGGAGTGACAAAGGCGAAGGCACTAGTCAAGGGCGAATCCGATGATCAAGGACGTAGGCTAGAGTTTCGAAAACGATTAGAGACCGGAGTAGTTCTAGCAGTAAACTATGCCGACGCCGTGGTATGGTTTTTTGTGGCTGTATTGCGGAAGAGAAATCAAGTAAGGCTTTGGGGAGAGTACGCGCGCAAGCGATAAATTTAAAGGAAATTGACGGAGGAACACCACAAGGAGTGGAGTGTGCGGCT

>M16_GvarMB5_BY_Gvar_Dmue_KAR-18_1760pb_MG773238

AGCTATATGTGGAGCGGTGAAAGGCTCAGTAACGGGCGATTTATTTAATCTCCTGGGGCGGACAACATCGGGAAACTGATGGGAAAACGTCTAAGTTGCATTAAT----TTTAGTGTGACGTAAACGATTATCGTGCAGGAGAGTAAGATGCCATCCTATCAGTTAGTAAGTAGGGTAAGGGCCTACTTAGACGAAGACGGGTACGGGGAATGAGGGTTTGATTCCGGAGAGGGAGCCTGAGAGATGGCTACCAGGACCAAGGTCAGCAGCAGGCGCGAAAATTATCGAAGCCCGCCTAGGGGCGATAGTGAGGAGACGTGTATAACGAAGTACGTGTAAAGAACGTACTAATAACTGGAGGTCAAGTCTGGTGCCAGCATCCGCGGTAATTCCAGCTCCAGGGGTGTCTATGATGATTGCTGCGATTAAAAAGTCCGTAGTCAAGCTGCCTGACTGACCTGCAATGTGATTGATTAAGGAACGAGCAGGGTTAGGAAAGCAGAGAATTAGGAGCGACCGAGGGCTAGAGTATTGAATGGCGAGAGGTGAAATTTGATGACCCATTCAGGAGTGACAAAGGCGAAGGCACTAGTCAAGGGCGAATCCGATGATCAAGGACGTAGGCTAGAGTTTCGAAAACGATTAGAGACCGGAGTAGTTCTAGCAGTAAAATATGCCGACGCCGTGGTATGGTTTTTTGTGGCTGTATTGCGGAAGAGAAATCAAGTAAGGCTTTGGGGAGAGTACGCGCGCAAGCGATAAATTTAAAGGAAATTGACGGAGGAACACCACAAGGAGTGGAGTGTGCGGCTTAATTTGACTCAACGCGGGACAGCTTACCAGGCCCGATAATCGAGCGAGCGTTGTACGCGATAGATTAAAGAGTGGTGCATGGCTGCTATCGACAGTTGGGGTGACC-TTAGGGTTAATTCCGGCAAGTAGTGAGACCCCTGCAGATAGTGGACAGGTATTTTT-AAAATACAGGAAGGAAGGGACAAGAGCAGGTCAGTGATGCCCTTAGATGGCCTGGGCTGCACGCGCACTACAGTGGTCATTATAAGTAGAAGTTAGAAGTAAAGATGATCGAGAGGGACTGAGCTTTGTAAGAGGCTCACGAACGAGGAATTGCTAGTAATCGTAGGCTCATTAAGATACGATGAATATGTCCCTGTACCTTGTACACACCGCCCGTCGTTATCGAAGATGGAATTGTGTGCGAACGAGCAACAAGCGAGTGAGCGCATAGTTCTAGATGTGATAAAAGTCGTAACAAGGCAACTGTAGGAGAACCTGCAGTTGGATCATACAGATTTA-TTTTAGGTAGTTTATAA---AAAATTCCTTACGCAAGGGATCGTTTGGCTCTATGTGCGATGAAGGTCGGAGCAGTATTCGATAATATAAGATGATTGTATAAATTGTCTTATGTTACTGAATAGAGTAGTGATGCTCGATTACTCCTTTGAATTAAGCATATGAGTAAAGGAAGGAAAAGAAACCAACGAGGATTCTCTTATTAGTGGCGAATGAACAGAGAACAGCCCTAAGTGTAATCAAATTCGTTGAATTTGAGATGTCAGGTATCATGTGGTGTGAATGTACTGGAAAGTACTGCCGAAGAGAGTTATAGCCTCGTAGCACCATTTTTTAGGTATGAGTAGTTGTGCTCGGTAATGCACAATGAAATAGGTGGTAGTGTCCATCTAAGGCTAAATATGACATAGAGACCGATAGTAAATAAGTAAAGTGATCGAAAATGGAATAGA

>M18_GvarM48_HU_Gvar_Dmue_KAR-18_1759pb_MG773240

AGCTATATGTGGAGCGGTGAAAGGCTCAGTAACGGGCGATTTATTTAATCTCCTGGGACGGACAACATCGGGAAACTGATGGGAAAACGTCTAAGTTGCATTAAT----GTTAGTGTGACGTAAACGAT-ATCGTGCAGGAGAGTAAGATGCCATCCTATCAGTTAGTAAGTAGGGTAAGGGCCTACTTAGACGAAGACGGGTACGGGGAATGAGGGTTTGATTCCGGAGAGGGAGCCTGAGAGATGGCTACCAGGACCAAGGTCAGCAGCAGGCGCGAAAATTATCGAAGCCCGCCTAGGGGCGATAGTGAGGAGACGTGTATAACGAAGTACGTGTAAAGAACGTACTAATAACTGGAGGTCAAGTCTGGTGCCAGCATCCGCGGTAATTCCAGCTCCAGGGGTGTCTATGATGATTGCTGCGATTAAAAAGTCCGTAGTCAAGCTGACTGACTGACCTGCAATGTGATTGATTAAGGAACGAGCAGGGTTAGGAAAGCAGAGAATTAGGAGCGACCGAGGGCTAGAGTATTGAATGGCGAGAGGTGAAATTTGATGACCCATTCAGGAGTGACAAAGGCGAAGGCACTAGTCAAGGGCGAATCCGATGATCAAGGACGTAGGCTAGAGTTTCGAAAACGATTAGAGACCGGAGTAGTTCTAGCAGTAAACTATGCCGACGCCGTGATATAGTTTTTTGTGGCTGTATTGCGGAAGAGAAATCAAGTAAGGCTTTGGGGAGAGTACGCGCGCAAGCGATAAATTTAAAGGAAATTGACGGAGGAACACCACAAGGAGTGGAGTGTGCGGCTTAATTTGACTCAACGCGGGACAGCTTACCAGGCCCGATAATCGAGCGAGCGTTGTACGCGATAGATTAAAGAGTGGTGCATGGCTGCTATCGACAGTTGGGGTGACCTTTAGGGTTAATTCCGGCAAGTAGTGAGACCCCTGCAGATAGTGGACAGGTATTTTT-AAAATACAGGAAGGAAGGGACAAGAGCAGGTCAGTGATGCCCTTAGATGGCCTGGGCTGCACGCGCACTACAGTGGTCATTATAAGTAGAAGTTAGAAGTAAAGATGATCGAGAGGGACTGAGCTTTGTAAGAGGCTCACGAACGAGGAATTGCTAGTAATCGTAGGCTCATTAAGATACGATGAATATGTCCCTGTACCTTGTACACACCGCCCGTCGTTATCGAAGATGGAATTGTGTGCGAACGAGCAACAAGCGAGTGAGCGCATAGTTCTAGATGTGATAAAAGTCGTAACAAGGCAACTGTAGGAGAACCTGTAGTTGGATCATACAGATTTA-ATTTAAGTAGTTTTTAA---TAAATTCCTTACGCAAGGGATCGTTTGGCTCTATGTGCGATGAAGGTCGGAGCAGTATTCGATAATATAAGATGATTGTATAAATTGTCTTATGTTACTGAATAGAGTAGTGATGCTCGATTACTCCTTTGAATTAAGCATATGAGTAAAGG-AGGAGAAGAAACTAACAAGGATTCTCTTATTAGTGGCGAATGAACAGAGAACAGCCCTAAGTGTAATCAAATTCTATGAATTTGAGATGTCAGGTATCATGTGGTGTGAATGTACTGGAAAGTACTGCCGAAGAGAGTTATAGCCTCGTAACACCATTTTGTAGGTATGAGTAGTTGTGCTCGGTAATGCACAATGAAATAGGTGGTAGTGTCCATCTAAGGCTAAATATGACATAGAGACCGATAGTGAATAAGTAAAGTGATCGAAAATGGAATAGA

>M9_ProbM33_HU_Prob_Dmue_KAR-18_1767pb_MG773232

AGCTATATGTGGAGCGGTGAAAGGCTCAGTAACGGGCGATTTATTTAATCTCCTGGGACGGACAACATCGGGAAACTGATGGGAAAACGTCTAAGTTGCATTAATATTAGTTAGTGTGACGTAAACGGTAATCGTGCAGGAGAGTAAGATGCCATCCTATCAGTTAGTAAGTAGGGTAAGGGCCTACTTAGACGAAGACGGGTACGGGGAATGAGGGTTTGATTCCGGAGAGGGAGCCTGAGAGATGGCTACCAGGACCAAGGTCAGCAGCAGGCGCGAAAATTATCGAAGCCCGCCTAGGGGCGATAGTGAGGAGACGTGTATAACGAAGTACGTGTAAAGAACGTACTAATAACTGGAGGTCAAGTCTGGTGCCAGCATCCGCGGTAATTCCAGCTCCAGGGGTGTCTATGATGATTGCTGCGATTAAAAAGTCCGTAGTCAAACTGACTGACTGACCTGCAATGTGATTGATTAAAGAACGAGCAGGGTTAGGAAAGCAGAGAATTAGGAGCGACCGAGGGCTAGAGTATTGAATGGCGAGAGGTGAAATTTGATGACCCATTCAGGAGTGACAAAGGCGAAGGCACTAGTCAAGGGCGAATCCGATGATCAAGGACGTAGGCTAGAGTTTCGAAAACGATTAGAGACCGGAGTAGTTCTAGCAGTAAACTATGCCGACGCCGTGATATTGTTTTTTGTGGCAGTATTGCGGAAGAGAAATCAAGTAAAGCTTTGGGGAGAGTACGCGCGCAAGCGATAAATTTAAAGGAAATTGACGGAGGAACACCACAAGGAGTGGAGTGTGCGGCTTAATTTGACTCAACGCGGGACAGCTTACCAGGCCCGATAATCGAGCGAGCGTAGTACGCGATAGATTAAAGAGTGGTGCATGGCTGCTATCGACAGTTGGGGTGACC-TTAGGGTTAATTCCGGCAAGTAGTGAGACCCCTGCAGATAGTGGACAGGTATTTTT-AAAATACAGGAAGGAAGGGACAAGAGCAGGTCAGTGATGCCCTTAGATGGCCTGGGCTGCACGCGCACTACAGTGGTCATTATAAGTAGAAATTAGAAATAAAAATGATCGAGAGGGACTGAGCTTTGTAAGAGGCTCACGAACGAGGAATTGCTAGTAATCGTAGGCTCATTAAGATACGATGAATATGTCCCTGTACCTTGTACACACCGCCCGTCGTTATCGAAGATGGAATTGTGTGCGAACGAGCAACAAGCGAGTGAGCGCATAGTTCTAGATGTGATAAAAGTCGTAACAAGGCAACTGTAGGAGAACCTGTAGTTGGATCATACAGATTTA-TATTAAGTAGTTTTTTATATTAAATTCCTTACGCAAGGGATCGTTTGGTTCTATGTACGATGAAGGTCGGAGCAGTATTCGATAATATAAAATGATTGTTTAAATTGTTTTATGTTACTGAATAGAGTAGTGATGCTCGATTACTCCTTTGAATTAAGCATATGAGTAAAGGAAGGAAAAGAAACTAACAAGGATTCTCTTATTAGTGGCGAATGAACAGAGAAGAGCCCTAAGTGTAATCAAATTCTATGAATTTGAGATGTCAGGTATAAAGTGGTGTGAATGTATTGGAAAATACAGCCTAAGAGAGTTATAGCCTCGTAACACCATTTTGTAGGAATGAGTAGTTGTGCTCGGTAATGCACAATGAAATAGGTGGTAGTGTCCATCTAAGGCTAAATATGACATAGAGACCGATAGTGAATAAGTAAAGTGATCGAAAATGGAATAGA

>M21_PrOv1_PL_Prob_Dmue_KAR-18_1759pb_MG773243

AGCTATATGTGGAGCGGTGAAAGGCTCAGTAACGGGCGATTTATTTAATCTCCTGGGACGGACAACATCGGGAAACTGATGGGAAAACGTCTAAGTTGCAGTTTT----GTTATTGCGACGTAAACGGTAATCGTGCAGGAGAGTAAGATGCCATCCTATCAGTTAGTAAGTAGGGTAAGGGCCTACTTAGACGAAGACGGGTACGGGGAATGAGGGTTTGATTCCGGAGAGGGAGCCTGAGAGATGGCTACCAGGACCAAGGTCAGCAGCAGGCGCGAAAATTATCGAAGCCCGCCTAGGGGCGATAGTGAGGAGACGTGTAT-TCGAAGTACGTGTAAAGAACGTACTAATAACTGGAGGTCAAGTCTGGTGCCAGCATCCGCGGTAATTCCAGCTCCAGGGGTGTCTATGATGATTGCTGCGATTAAAAAGTCCGTAGTCAAGCTGACTGACTGACCTGCAATGTGATTGATTAAAGAACGAGCAGGGTTAGGAAAGCAGAGAATTAGGAGCGACCGAGGGCTAGAGTATTGAATGGCGAGAGGTGAAATTTGATGACCCATTCAGGAGTGACAAAGGCGAAGGCACTAGTCAAGGGCGAATCCGATGATCAAGGACGTAGGCTAGAGTTTCGAAAACGATTAGAGACCGGAGTAGTTCTAGCAGTAAACTATGCCGACGCCGTGATATTGTTTTTTGTGGCGGTATTGCGGAAGAGAAATCAAGTAAAGCTTTGGGGAGAGTACGCGCGCAAGCGATAAATTTAAAGGAAATTGACGGAGG-ACACCACAAGGAGTGGAGTGTGCGGCTTAATTTGACTCAACGCGGGACAGCTTACCA-GCCCGATAATCGAGCGAGCGTAGTACGCGATAGATTAAAGAGTGGTGCATGGCTGCTATCGACAGTTGGGGTGACC-TTAGGGTTAATTCCGGCAAGTAGTGAGACCCCTGCAGATAGTGGACAGGTATTTTTCAAAATACAGGAAGGAAGGGACAAGAGCAGGTCAGTGATGCCCTTAGATGGCCTGGGCTGCACGCGCACTACAGTGGTCATTATAAGTAGAAGTTAGATATAAAGATGATCGAGAGGGACTGAGCTTTGTAAGAGGCTCACGAACGAGGAATTGCTAGTAATCGTAGGCTCATTAAGATACGATGAATATGTCCCTGTACCTTGTACACACCGCCCGTCGTTATCGAAGATGGAATTGTGTGCGAACGAGCAACAAGCGAGTGAGCGCATAGTTCTAGATGTGATAAAAGTCGTAACAAGGCAACTGTAGGAGAACCTGTAGTTGGATCATACAGATTTATTTTTAAGTAGTTTTTTA---TAATTTCCTTACGCAAGGGATCGTTTGGTTCTATGTACGATGAAGGTCGAAGCAGTATTCGATAATGTAAGATGGTTGTATAAATTGTCTTATGTTACTGAATAGAGTAGTGATGCTCGAATACTCCTTTGAATTAAGCATATGAGTAAAGGAAGGAGAAGAAACTAACAAGGATTCTCTTATTAGTGGCGAATGAACAGAGAATAGCCCTAAGTGTAATCAAATTCATTGAATTTGAGATGTCAGGTATCATGTAGTGTGAATATACTGGAAAGTATTGCCAGAGAGAGTTATAGCCTCGTAGCACTATTTTGTAGGGATGAGTAGTTGTGCTCGGTAATGCACAATGAAATAGGTGGTAGTGTCCATCTAAGGCTAAATATGACATAGAGACCGATAGTGGATAAGTAAAGTGATCGAAAATGGAATAGA

>KR871361_DE_Gpul_Ddue_GRA-15_1075pb_KR871361

GTGGAGCGGTGAAAGGCTCAGTAACGGGCGATTTATTTAATCTCCTGGGACGGACAACATCGGGAAACTGATGGGAAAACGTCTAAGTTGCAGTTTT----GTGAGTGCGACGTAAACGATAATCGTGCAGGAGAGTAAGATGCCATCCTATCAGTTAGTAAGTAGGGTAAGGGCCTACTTAGACGAAGACGGGTACGGGGAATGAGGGTTTGATTCCGGAGAGGGAGCCTGAGAGATGGCTACCAGGACCAAGGTCAGCAGCAGGCGCGAAAATTATCGAAGCCCGCCTAGGGGCGATAGTGAGGAGACGTGTATATCGAAGTACGTGTAAAGAACGTACTAATAACTGGAGGTCAAGTCTGGTGCCAGCATCCGCGGTAATTCCAGCTCCAGGGGTGTCTATGATGATTGCTGCGATTAAAAAGTCCGTAGTCAAGCTGACTGACTGACCTGCAATGTGATTGATTAAAGAACGAGCAGGGTTAGGAAAGCAGAGAATTAGGAGCGACCGAGGGCTAGAGTATTGAATGGCGAGAGGTGAAATTTGATGACCCATTCAGGAGTGACAAAGGCGAAGGCACTAGTCAAGGGCGAATCCGATGATCAAGGACGTAGGCTAGAGTTTCGAAAACGATTAGAAACCGGAGTAGTTCTAGCAGTAAACTATGCCGACGCCGTGATATTGTTTTTTGTGGCGGTATTGCGGAAGAGAAATCAAGTAGGGCTTTGGGGAGAGTACGCGCGCAAGCGATAAATTTAAAGGAAATTGACGGAGGAACACCACAAGGAGTGGAGTGTGCGGCTTAATTTGACTCAACGCGGGACAGCTTACCAGGCCCGATAATCGAGCGAGCGTAGTACGCGATAGATTAAAGAGTGGTGCATGGCTGCTATCGACAGTTGGGGTGACC-TTAGGGTTAATTCCGGCAAGTAGTGAGACCCCTGCAGATAGTGGACAGGTATTTTTTAAAATACAGGAAGGAAGGGACAAGAGCAGGTCAGTGATGCCCTTAGATGGCCTGGGCTGCACGCGCACTACAGTGGTCATTATAAGTAGAAGTTAGATATAAAGATGATC

Figure 5 *: Dictyocoela berillonum*

>PL1-08_PL_Groe_Dber1_ADR-18_249pb_PL1-08

CATGTGTAAGCGAAGCGTAACGTGGAGCGGTGAAAGGCTCAGTAACGGGCGAGTTATTTGTTCTCCTGGGACGGACAACACCGGGAAACTGGTGGGAAAACGTCTAAGTTGCGGTTTTTTAATCGTGGCGTAAACCATGTGGTGCAGGAGAGTAAGCTGCCATCCTATCAGTTAGTAAGTAGGGTAAGGGCCTACTTAGACGAAGACGGGTACGGGGAATGAGGGTTTGATTCCGGAGAGGGAGCCTGA

>AJ438957_GB_Echinogammarus_berilloni_Dber_TER-04_912pb_AJ438957

CATGTGTAAGCGAAGCGTAACGTGGAGCGGTGAAAGGCTCAGTAACGGGCGAGTTATTTGTTCTCCTGGGACGGACAACACCGGGAAACTGGTGGGAAAACGTCTAAGTTGCGGTTTTTTAATCGTGGCGTAAACCATTTGGTGCAGGAGAGTAAGCTGCCATCCTATCAGTTAGTAAGTAGGGTAAGGGCCTACTTAGACGAAGACGGGTACGGGGAATGAGGGTTTGATTCCGGAGAGGGAGCCTGAGAGACGGCTACCAGGACCAAGGTCAGCAGCAGGCGCGAAAATTATCGAAGCCCGCATAGGGGCGATAGTGAGGAGACGTGTATTACGAAGTGTGTGTAAAGAACGCACTAATAACTGGAGGTCAAGTCTGGTGCCAGCATCCGCGGTAATTCCAGCTCCAGGGGTGTCTATGATGATTGCTGCGATTAAAAAGTCCGTAGTCAAGCTGACTGACTTGCCTGCAATGTGACTGATTAAGAGACGAGCAGGGCTAGGAAAGCAGAGAATTAGGAGCGACCGAGGGCTAGAGTATTGAATGGCGAGAGGTGAAATTTGATGACCCATTCAGGAGTGACAAAGGCGAAGGCACTAGTCAAGGGCGAATCCGATGATCAAGGACGTAGGCTAGAGTTTCGAAAACGATTAGAGACCGGAGTAGTTCTAGCAGTAAACTATGCCGACGCCGTGGTATGGTATTCTGTATTGCGGAAGAGAAATCAAGTAAGGCTTTGGGGAGAGTACGCGCGCAAGCGATAAATTTAAAGGAAATTGACGGAGGAACACCACAAGGAGTGGAGTGTGCGGCTTAATTTGACTCAACGCGGGACAGCTTACCAGGCCCGATAATCATACGAGCGTAGTACGCGATAGGTTAAAGAGTGGTGCATGGCTGCTATCGACAGT

>B7_ProbM32_HU_Prob_Dber_KAR-18_1756pb_MG773251*

AGCGTAACGTGGAGCGGTGAAAGGCTCAGTAACGGGCGAGTTATTTGTTCTCCTGGGACGGACAACACCGGGAAACTGGTGGGAAAACGTCTAAGTTGCGGTTTTTTAATCGTGGCGTAAACCATTTGGTGCAGGAGAGTAAGCTGCCATCCTATCAGTTAGTAAGTAGGGTAAGGGCCTACTTAGACGAAGACGGGTACGGGGAATGAGGGTTTGATTCCGGAGAGGGAGCCTGAGAGACGGCTACCAGGACCAAGGTCAGCAGCAGGCGCGAAAATTATCGAAGCCCGCGTAGGGGCGATAGTGAGGAGACGTGTATTACGAAGTGTGTGTAAAGAACGCACTAATAACTGGAGGTCAAGTCTGGTGCCAGCATCCGCGGTAATTCCAGCTCCAGGGGTGTCTATGATGATTGCTGCGATTAAAAAGTCCGTAGTCAAGCTGACTGACTTGCCTGCAATGTGACTGATTAAGAGACGAGCAGGGCTAGGAAAGCAGAGAATTAGGAGCGACCGAGGGCTAGAGTATTGAATGGCGAGAGGTGAAATTTGATGACCCATTCAGGAGTGACAAAGGCGAAGGCACTAGTCAAGGGCGAATCCGATGATCAAGGACGTAGGCTAGAGTTTCGAAAACGATTAGAGACCGGAGTAGTTCTAGCAGTAAACTATGCCGACGCCGTGGTATGGTATTCTGTATTGCGGAAGAGAAATCAAGTAAGGCTTTGGGGAGAGTACGCGCGCAAGCGATAAATTTAAAGGAAATTGACGGAGGAACACCACAAGGAGTGGAGTGTGCGGCTTAATTTGACTCAACGCGGGACAGCTTACCAGGCCCGATAATCATACGAGCGTAGTACGCGATAGGTTAGAGAGTGGTGCATGGCTGCTATCGACAGTTGGGGTGACCTTAGGGTTAATTCCGGCAAGTAGTGAGACCTCTGCAGTTATGGACAGGTATTTTTAAGATACAGGAAGGAAGAGACAAGAGCAGGTCAGTGATGCCCTTAGATGGCCTGGGCTGCACGCGCACTACAGTGGTCATTATAAGTAGAAGTTAGATTTTAAAGATGATCGAGAGGGACTGGGCTTTGTAAGAGGCCCAAGAACGAGGAATTGCTAGTAATCGTAGGCTCATTAAGATACGATGAATATGTCCCTGTACCTTGTACACACCGCCCGTCGTTATCGAAGATGGAATTGTGTGCGAACGAGCAACAAGCGAGTGAGCGCATAGTTCTAGATGTGATAAAAGTCGTAACAAGGCAACTGTAGGAGAACCTGTAGTTGGATCATACAGATATAATAAAAAGGTAGGTTTGTTTTTAATTTCCCTGCGCAAGGGATCGTTTGGTTCTATGTGCGATGAAGGTCGGAGCAGTATCTGATAATGTAGAATGATTGTAATAATTGTTCTATGTGACTGAATTGAACAGTGATGTTCGGGTACTCCTTTGAATTAAGCATATGAGTAAAGGAAGGAAAAGAAACTAACTAGGATTCTCTTATTAGTGGCGAATGAACAGAGAATAGCCCAAGTGTAATCAATAATAAATAATTATTGAGATGTCTAGTATATTGACGTGAAAACAATGGAAAGTGTTGCCGTAGAGAGTTATAGCCTCGTAGCGTCTAAATTTAAAAAGGGAGTAGTTGTGCTCGGTAATGCACAATGAATAGGTGGTAGTGTCCATCTAAGGCTAAATATGACATAGAGACCGATAGTGAATAAGTAGAGTGATCGAATATGGAATAGA
