# Supplementary material for: Microsporidian infections in the species complex Gammarus roeselii (Amphipoda) over its geographical range: evidence for both host–parasite co-diversification and recent host shifts
Source: Parasit Vectors. 2019 Jun 28;12:327. doi: 10.1186/s13071-019-3571-z (PMC6599290; doi:10.1186/s13071-019-3571-z)
Supplement: Supplementary file 5 — Additional file 5: Data S2. Alignments based on SSU rDNA sequences, used for trees in Figs. 2, 3, 4, 5 and Additional file 1: Figure S1. [file 13071_2019_3571_MOESM5_ESM.docx]

Microsporidian infections in the Amphipoda species complex *Gammarus roeselii* over its geographic range: evidence for both host-parasite co-diversification and recent host-shifts.

Adrien Quiles, Karolina Bacela-Spychalska, Maria Teixeira, Nicolas Lambin, Michal Grabowski, Thierry Rigaud & Rémi A. Wattier

Additional file 5 - Data S2. Alignments based on SSU rDNA fragment sequences, used for trees in Fig. 2 to Fig 5 and Fig S1.

Alignment Figure 2 :

>>AF027684_ND_Aedes_aegypti_Edhazardia_aedis_BAK-98_1448pb_

-------CACCAGGTTGATTCTGCCTGACGTGGACGCTTGCCTCGAAGATTAAGCCATGCAAGTCT--------GTGAA----------TATATGAAA-CAGTGTACGGCTCAG-TATAACACCTAT-ATCTACCCAC------TTTT-TA-------AAT-ATAACCATGGTAA-ACTATGGCTAATATA---------------------------------------------------------------------------------------------------------------------------------GTGGATGAGTGGGTGACCTATCAGC--TT-GACGGTACGGTAAGTGCGTACCGAGGCTATAACGGGTAACGGGGAATATGGGTTTTATTCCGGAGAGGGAGC-CTGAGAGATGGCTGCCACGTCCAAGGACGGCAGCAGGCGCGAAACTTACCCAATGA-------ACGTTGAGGTAGTTACGAGGCGTA--GT-A---------------------------T--TATGAA---------GTGTGTGTAAAGAGCATA---CTGAAGTACT---GGAGGGCAAG-TCTGGTGCCAGCAGCCGCGGTAATACCAGCTCCAGTAGCGTCTGTTTTTATTGCTGCGGTTAAAACGTGCGTAGT--CGGCTAGTTGTGTGAATAGTGA-------TTCGGGATGGTACGCGGAGTAATCTGTGGATTGTGCTATAAT--------------------TGCTATTTGTGAATATGCAA-----------CAGCCTGGGTTAAAT------ATGCACTTTCATGGT--------------GCT-----TTTAATTTCAGGA-ACTT------------------------------------------ATAGCTTGAGACAGGGACGGGGTAATTTTATTTGGTAGCGAGAGGTGACAAT-CGATGACCTA-CCAAGGAGGACCGGAGGCGAAAGCGATTACCAAGAACTGATCTGACGATCAAGCACGTGAGCAGGA-GTATCGAAGAGGATT--AGAGACCCACGTAGTTCCTT-GCAGTCAACGATGCCAACGTAG-TGGTGAT-T--------------------------------------------------------------TTGGTC-ATTGCTAGAGCGAAAG--CTAGTGTATGGGCTCCGGGGATAGTACGGACGCAAGTTTGAAACTTGAA--GAAATTGACGGAAGGACACCACAAGGAGTGGAGTGTGCGGGTTAATTTGACTCAACGCGGG-AAAACTTACCCGGGCAG-GCGATTGTTGTGAGAAGGA-----AT-ACCTGATGATGA--------------TCGCGTGTGGTGCATGGCCGTTCTTAACACGTGGAGTGATCTG------------------------------------------TCTGGTCAAATCTGAT-AACGCGTGAGAGGTGAGTGG------TTTT----------------------------------TAACCAG-ACGATGTGTG-TAAGCACAAGGAAGAGACACCCAATAACA-GGTCTGTGATGCCCGTAGATGTTCGGGGCTCCACGCGCACTACAATGAGTG-G-----------TGGT---GCT--ATA---------------------------AGTAGTAGCCAGTCGTAGTTGGGATTGACATATGTAA--TTATGTCATGAACGTGGAATTCCTAGTAGT--------TGGTAG-TCACTAACGACTAACGAATGAGT-CCCTGTTCTTTGTACACACCGCCCGTCGTTATCTAAGATGGAGGTGCGGGTGAAGATGTG---------------------------------------------AGTGTGTAGAGTTGGTGCGATGCATGGAAGTTAATGACGAGTGCGAGTAGT-TA--GCTATGAGTATGAGTTGTTGATGGAAGTGGTTGTAACTAATGGCATGAGCATAAG----AACCCGTG----------------------------------------------------------------CAGCTAGATTGGATGTAAGTCGTAACA

>>AF132544_US_Cnephia_ornithophilia_Caudospora_palustris_ADL-00_1341pb_

----------------------------------------TTTTAAGGATTTAGCCATGCATGTCT-------AGCGAAGC-------TTCGGTGGAG-CGGCGAACGGCTCAG-TAATACAGTTATGATCTACTCACA-----CAGCCAA-------TGG-ATAACCACGGTAA-CCTGTGGCTAACACAA---------------------------CGGAAATATGTCATTGGTGAAACAGG------------------------------------------------------------------ACATTGATGGTGATTGAGTTTCTGGCCTATCAGCTAGT-GAAGGTAGGGTAAGGGCCTACCTTGGCGATGACGGGTAACGGGGGATCAGGGTTTGATTCCGGAGAAGGAGC-CTGAGAGATGGCTGCTACGTCCAAGGATGGCAGCAGGCGCGAAAATTACCCAATCTCC--ATTTGGAGGAGGTAGTGACGAAACGTA--TG------------------------------CGAGAGAG---------GGCAGAGCAAATATCTGCCC-GAGAGCGACT---GGAGGGCAAG-TCTGGTGCCAGCAGCCGCGGTAATTCCAGCTCCAGTAGCGTCCATGTGTGTTGCTGCAGTTAAAACGTCCGTAGT--CGTGTGAGGCGCTTATGAACATTCATAAGTGTC----------------------------------------------------------ACTGTCCTGTGGGT----------------------------AAAT------GTGACGTATCAAAC---------------GTC----ACCTGTCCACAGCA-GCAT------------------------------------------ACACAGCAGGAGCAGTTGAGGGCGATGGTATTCCTGTGCGAGAGGTGAAATT-CAATGACCAC-TGGAGGACCGACAGAAGCGAAAGCGTTCGTCATGGATGTTTCCGATGATCAAGGACGTAAGCCGGA-GTATCGAAGGCGATT--AGAAACCGTCGTAGTTC-CG-GCCGTAAACGATGCCGACCCAC-AGGGATT-C---------------------------------------------------------------AAGTC-TTTGTGTGGAGAAATT--AGAGTTTATGGGCTCTGGGGATAGTATAGGCGCAAGCCCGAAACTTTAA--GAAATTGACGGAAGGACACCACAAGGAGTGGAGTATGCGGGTTAATTTGACTCAACGCGGG-ACAACTTACCAGGGCCT-GATGGAG-GTGC-GAAGTA--------AACATT-ATCGTGTTTGCTGATTCTCCAGCGAGTGGTGCATGGCCGTTCCCAACACGTGGGGTGACCTG------------------------------------------TCTGGCTAATTCCGAT-AACGCGTGAGACCCTCGTGC------CGTGCA-------------------------------AATGGCAG-ACGATCACCGACGAGGTGAAGGAAGCAGGGGGCGATAACA-GGTCCGTGATGCCCTTAGATGTTCTGGGCTTCACGCGTACTACATTGCGCA------------------GGGTTTACTT--------------------------AAGCGAAACCTGCGCGAGGTCGGGATAGACAGTTGTAA--ATCTGTCTTGAACGAGGAATTCCTAGTAAC--------AGCGAG-TCACTAACTTGTTGTGAATGCGT-CCCTGTCCTTTGTACACACCGCCCGTCGTTATCTAAGATGGATGTGGGGATGAAGAGAAC------------------------------------------TTTGTTTTTTGAATCTCTGCAACTAGATTGGATACAA-----GTCGTAACAAGG-TA--ACC-----------------------------------------------------------------------------------------------------------------------------------------------------------

>>AJ252960_GB_Simulium_sp._Polydispyrenia_simulii_CHE-00_1291pb_

-------CACCAGGTTGATTCTGCCTGACGTGGACGCTTGTTTTAAGGATTTAGCCATGCATGTCT-------AGCGAAGC-------GTAAGTGGAG-CGGCGAACGGCTCAG-TAATACAGTTATGATCTATTCATG-----CAGCAAG-------GGG-ATAACCACGGTAA-CCTGTGGCTAAGACGA---------------------------CGGAGATATGCGGGCACTGAGGTGTA------------------------------------------------------------------GCATTGCTTGTGACTGAGTTTCTGGCCTATCAGCTAGT-GAAGGTAGGGTAAGGGCCTACCTTGGCGATGACGGGTAACGGGGGATCAGGGTTTGATTCCGGAGAAGGAGC-CTGAGAGATGGCTGCTACGTCCAAGGATGGCAGCAGGCGCGAAAATTACCCAATCTCC--CACGGGAGGAGGTAGTGACGAAACGTA--TG------------------------------CGAGAGAG---------AGCAGTGTAAACGCCTGCTC-GAGAGCGACT---GGAGGGCAAG-TCTGGTGCCAGCAGCCGCGGTAATTCCAGCTCCAGTAGCGTCCATGTGTGTTGCTGCAGTTAAAACGTCCGTAGT--CGTGTGAGAGTTTTACGAATG-TCGTAGGGCGC----------------------------------------------------------ACTGTCCTGTGGGT----------------------------AACT------GTGTCACATCAAAC---------------GTC----ACGGAGCCACAGCA-GCGA------------------------------------------GCACAGCAGGAGCAGTTGAGGGCGATGGTATTCCTGTGCGAGAGGTGAAATT-CGATGACCAC-TGGAGGACCGACAGAAGCGAAAGCGTTCGTCATGGATGTTTCCGATGATCAAGGACGTAAGCCGGA-GTATCGAAGGCGATT--AGAAACCGTCGTAGTTC-CG-GCCGTAAACGATGCCGACCCAC-AGGGATG-A---------------------------------------------------------------CAGTC-CTTGTGTGGAGAAATT--AGAGTTTATGGGCTCTGGGGATAGTACAGGCGCAAGCCCGAAACTTTAA--GAAATTGACGGAAGGACACCACAAGGAGTGGAGTATGCGGGTTAATTTGACTCAACGCGGG-ACAACTTACCAGGGCCG-GATGGAG-GCGC-GAAGCA--------AGCATG-ACCGTGCTTGCTGATTCTCCAGCGAGTGGTGCATGGCCGTTCCCAACACGTGGGGTGACCTG------------------------------------------TCTGGCTAATTCCGAT-AACGCGTGAGACCCTCGTGC------CGTGCG-------------------------------AATGGCAG-ACGATCACCGACAAGGTGAGGGAAGCAGGGGGCGATAACA-GGTCCGTGATGCCCTTAGATGTTCTGGGCTTCGCGCGTACTACATTGTGCA------------------GGGCTTACTG--------------------------AAGCGAAACCTGCACGAGGTCGGGACAGACGGTTGTAA--ATCCGTCTCGAACGAGGAATTCCTAGTAAC--------AGCGAG-TCACTAACTTGTTGTGAATGCGT-CCCTGTCCTTTGTACACACCGCCCGTCGCTAT-----------------------------------------------------------------------------------------------------------------------------------------------------------------------------------------------------------------------------------------------------------------------------------------------

>>AJ581995_UK_Nais_simplex_Bacillidium_vesiculoformis_MOR-05_1422pb_

--------------------------------------------------------ATGCAAGCTC--------GCGAAGC------CTTGGGCGGAG-CAGCAGACAGCTCAG-TAATACAGTTATAATTTACTCGTGAT---CTGA-TA-------CGG-ATAACCTTGGTAA-ACTAAGGCTAATACGTAGGATAGGACGACTGA------C----T-TGAGGAAGCAGCAGTAGTCCTGAG------------------------------------------------------------------GATTCTATCGCGATTAAGTTTCTGGCCTATCAGC--TT-GTTGGTAAGGTAATGGCTTACAAAGGCTTTGACGGGTAACGGGGAATCAGTGTTCGATTCCGGAGAGGGAGC-CTGAGAAATAGCTCCCACGTCCAAGGATGGCAGCAGGCGCGAAAATTTCCCAATTCCT--TCACGGGAGAGGTAGTTAAGAGACATG--AA-GTTCT----------------------ACTTAGAGAC---------TGTACAATAAATACGTAT---AGGATCGACT---GGAGGGCAAG-TCTGGTGCCAGCAGCCGCGGTAATTCCAGCTCCAGTAGCGCATATAATAGTTGCTGCAGTTAAAACGTCCGTAGT--TGATCACAAGGTATATT-------------TCAACATAGCATTTG--------------TTGGGCTCTGCC--------------------TCGGTACTGTGAGAA---------------------------AAAT------ATAG-TTCTCAAAGCAGGTT---ACGTGAGTA----ATTCAGCATGGGAC-ACAG------------------------------------------TGAATATAGGAACAGATGATGGCAATAGTATTCAGTAGTTAGAGGTGAAAAT-TGATGACCTG-CTGAGGACTGTCGGAAGCGAAGGCGATTGTCAAGTATGTCTCCGTTAATAAAGGACTTAAGCTAGA-GGATCGAAGATGATT--AGATACCGTCGTAGTTC-TA-GCTGTAAACGATGCCGACTAGG-TGAGGCC-A----------------------------------------------------------CAC--GGCTG-CGCCTTAGGAGAAATC--AAAGTCTATGGGCTCTGGGGATAGTATTTGCGCAAGCTTGAAACTTAAA--GAAATTGACGGAAGGACACCACAAGGAGTGGATTGTGCGGCTTAATTTGACTCAACGCGGG-ACAACTTACCAGGGCCG-AACTTGT-TTGA-GATTGG-----CG-GTTTGA-GAGAACCCCCGAGATTATAAGAAAGGTGGTGCATGGCCGTTTTCAACACGTGGGGTGACCTG------------------------------------------TCTGGTTAATTCCGTT-AACGCGTGAGGCCCTGTAGG------CAAC-GAT-----------------------------GTTGCCTG-ACGGATGGCGGCAAGCCATAGGAAGT--GGGGCCATAACA-GGTCAGTGATGCCCCTAGATGTTCTGGGCTGCACGCGCAATACAATG-GCACG-----------GTGA-GACTA-TGCACT-------GAGAAGTGT--------GCTCTAATCCGTGCCGAAGCTGGGATAAGCTCTTGTAA--ATGAGCTTTGAACGAGGAATTCCTAGTAAT--------CGCAAG-TCATTATGTTGCGATGAATGCGT-CCCTGTTCTTTGTACACACCGCCCGTCACTATCTCAGATGGATGTATTGATGAAATGGCTGGACG---------------------------------CAAGTAAGGCAGTTGAATCAGTGCAACTAGATAAGATATAA-----GTCGTAACAAGG-CT--GCTATAAGTG------------------------AACTTGTGGCAGGATCATT---------------------------------------------------------------------------------------------------------

>>AY582742_ND_Pectinatella_magnifica_Trichonosema_algonquinensis_DES-04_1027pb_

------------------------------------------------------------------------------------------------------------------------------------------------------------------------------------------------------------------------------------------------------------------------------------------------------------------------------------------------------------------------------------------------------------CCCTTGTTATGTTCCGGAGAAGGAGC-CTGAGAAATAGCTCCCACGTCCAAGGATGGCAGCAGGCGCGAAACTTACCCAATTCCT---ACAGGGAGAGGTAGTGAAGAGACGTG--TT---------------------------TATAATGAGGC---------TACATTGCAAAAAGATGA---GTGCCCGATT---GGAGGGCAAG-TCTGGTGCCAGCAGCCGCGGTAATTCCAGCTCCAATAGCGCATATGATAGTTGCTGCAGTTAAAACGTCCGTAGT--TGGCCACAC-----------------------------------------------------------------------------------TATTACTGTGAGGA---------------------------AATT------ATGT-TTCTCAAAG-----------ACTAGTA----TTATAGCATGGAAT-AATG------------------------------------------TGGAGAAAGGAACAGATGATGGCAATAGTATTGAATAGCGAGGGGTGAAAAC-CGATGACCTA-TTGAGGACTACCGGAGGCGAAGGCGATTGTCAAGTATGTATCCGTTAATCAAGGACTTAAGCTGGA-GGATCGAAGATGATT--AGATACCGTCGTAGTTC-CA-GCTGTAAACGATGCCGACTATA-TGGTAGA------------------------------------------------------------GAC--TCTAC-CGTATAAGGAGAAATC--AAAGTGTATAGGCTCTGGGGATAGTATTAATGCAAATTAGAAACTTAAA--GAAATTGACGGAAGGACACCACAAGGAGTGGATTGTGCGGCTTAATTTGACTCAACGCGGG-ACAACTTACCAGGGCCG-AATGTGT-AGGA-GAATGG-----CG-ACCCGA-GAGGATCCCCATGATTATGCAGGAGGTGGTGCATGGCCGTTTTCAACACGTGGGGTGACCTG------------------------------------------TCAGGTTAAATCCTTT-AACGCGTGAGGCTGTGAAGT------TTATAT-------------------------------GTAAATTG-ACGGATGGCGGCAAGCCATAGGAAGC--ACGGCGATAACA-GGTCAGTGATGCCCCTAGACGTTCTGGGCTGCACGCGCAATACAATGGGATG------------GAGA-ATTTAGTATGCT-------GAGAAGTATA-------GAAGTAATTCATTCCGAAGCAGGGATAAGCTTTTGTAA--ATAAGTTTTGAACGAGGAATTCCTAGTAAT--------CGCAAG-TCACTAAGTTGCGATGAATGAGTCCCCTGTTCTTTGTACACACCGCCCGTCATTATA----------------------------------------------------------------------------------------------------------------------------------------------------------------------------------------------------------------------------------------------------------------------------------------------

>>KX137906_DE_Ephemera_danica_Msp-groupH_GRA-17_445pb_

-------------------TCTGCCTGACGTGTGTGCTTGTCTTGAAGATTTAGCCATGCAAGTTT-------AGCGAAGC------CTTTGGTGGAG-CGGCGTACAGCTCAG-TAATACAGTTTTAATTTAATCGTAGT---GCTA-TA-------TGG-ATAACCATGGTAA-GCTATGGCTAATACAA--GCAAAGGCTATCAG------TG-----TGGCGAGGCATTGGTAGTTGACAGTCT---------------------------------------------------------------TTGACGACTACGACTAAGAATCTGGCCTATTAGC-AAA-GATGGTAGGGTAACGGCCTACCATGGCTATGATGGGTAACGGAGAATCAGTGTTCGATTCCGGAGAGGGAGC-TTGAGAAACAGCTCCCACGCCTAAGGGTGGCAGCAGGCGCGAAACTTACCCAATTCCG---AATAGGAGAGGTAGTGAAGAGACATG--AG-GCTCG----------------------GCCTAGAGAT---------TGCACAATAAATTAGTGT---AAGATCGACT---GGAGGGCAAG-TCTGGTGCC----------------------------------------------------------------------------------------------------------------------------------------------------------------------------------------------------------------------------------------------------------------------------------------------------------------------------------------------------------------------------------------------------------------------------------------------------------------------------------------------------------------------------------------------------------------------------------------------------------------------------------------------------------------------------------------------------------------------------------------------------------------------------------------------------------------------------------------------------------------------------------------------------------------------------------------------------------------------------------------------------------------------------------------------------------------------------------------------------------------------------------------------------------------------------------------------------------------------------------------------------------------------------------------------------------------------------------------------------------------------------------------------------------------------------------------------------------------------------------------------------------------------------------------------------------------------------------------

>>AWG-03_DE_Groe_Msp-VB_ADR-18_348pb_AWG-03-VB

-------------------------------------------------------------------------------------------------------------------------------------------------ATG-TA-------TGG-ATAACCATGGTAA-ACTATGGCTAATACAT--GAACAGGTTATTTA------T----A-TGGTGAGACATTGATAACAGACAG------------------------------------------------------------------GAAGCGACAACGATTAAGTTTCTGGCCTATCAGC-TTT-GAAGGTAGGGTAATGGCCTACCTTGGCTATGACGGGTAACGGGGGATCAGCGTCTGATACCGGAGAGGGAGC-CTGAGAAATAGCTCCCACGTCTAAGGATGGCAGCAGGCGCGAAACTTACCCAATCTCT--TTATGGAGGAGGTAGTGAAGAGACATG--AGAGATAT----------------------ATCTACAGAC---------TATACAATAAATTAGTAT---AGGATCGACT---GGAGGGCAAG-TCTGGTGCCAGCAGCCGCGGA----------------------------------------------------------------------------------------------------------------------------------------------------------------------------------------------------------------------------------------------------------------------------------------------------------------------------------------------------------------------------------------------------------------------------------------------------------------------------------------------------------------------------------------------------------------------------------------------------------------------------------------------------------------------------------------------------------------------------------------------------------------------------------------------------------------------------------------------------------------------------------------------------------------------------------------------------------------------------------------------------------------------------------------------------------------------------------------------------------------------------------------------------------------------------------------------------------------------------------------------------------------------------------------------------------------------------------------------------------------------------------------------------------------------------------------------------------------------------------------------------------------------------------------------------------------------------

>>AY305324_ND_Gryllus_bimaculatus_Paranosema_locustae_SOK-03_1337pb_

-------------------------TGACGTGTGCGCTAGTCTTGGGGACTTAGCCATGCAAGCCA--------GCGAAGC--------GGCGCGGAG-CGGCGGACGGCTCAG-TAGGACGGCGATAATCCACCCGCAGCA--GAGA-CA-------AGG-ATAACCGCGGCAA-GCTGCGGCTAAGACAT--GGACAGGCTGCGGC------G------CGGGGAGGCGCGGGCAGCGGACAG------------------------------------------------------------------GGAGCGGCTGCGGCGGAGTTGCTGGCCCATCAGC--TG-GTAGGTAGGGTAAGGGCCTACCTAGGCGACGACGGGTGACGGGGGGTGAGAGCCCGGAACCGGAGAGGGAGC-CTGAGAAACAGCTCCCACGTCCAAGGACGGCAGCAGGCGCGGAACTTGCCCAATCCC----GGCGGGGGAGGCAGCCACAAGACGTG--GG-----------------------------GGCCCGGAA---------CGCACGGCAAAGGAGTG----CGGGGCGACT---GGAGGGCAAG-TCTGGTGCCAGCAGCCGCGGTAATTCCAGCTCCAGGAGCGCACATGAGTGTTGCTGCAGTTAAAACGTCCGTAGT--CGGCCCGGG------------------------------------------------------------------------------------CGCGCTGTGAGGA---------------------------AACC------GCGG-GGCCCAAGG---------------CGG----CGGGCAGCACGGGA-GCGG------------------------------------------GGGCGACAGGAACGGACAACGGCCGCGGGATCCGGGGGCGAGAGGTGAAAAT-CGGGGACCCC-GCGGGGACGGGCGGAAGCGAAGGCGGCGGCCGGGGACGTGTCCGTTGATCAAGGACGAAGGCCGGA-GGATCAAAGATGATT--AGATACCGTCGTAGTTC-CG-GCAGTAAACGATGCCGACGGGG-CGAGGCC------------------------------------------------------------AGG--GGGCT-GTGTCCGGGAGAAATT--GGAGTGTTTGGGCTCTGGGGATAGTACGGTCGCAAGACAGAAACTTAAA--GAAATTGACGGAAGGACACCACAAGGAGTGGAGTGTGCGGCTTAATTTGACTCAACGCGGG-GCAGCTTACCAGGGCCG-GATGCGC-GGGA-GATTGG--------CGGCGG-AGCGCCCCCGAGATTGCGCAGGGAGTGGGTGCATGGCCGTTTGCAACACGTGGGGTGACCTG------------------------------------------TCTGGTTGATTCCGAC-AACGCGTGAGGCCTGGGGTG------CAGCAGGG-----------------------------GCTGTGCG-ACGGGCGGCGGCAAGCTGCAGGAGGG--CAGGCGAAAACA-GGTCAGTGATGCCCTCAGATGCTCTGGGCTGCACGCGCACTACAATGGCGCG------------GCGAGCGGGAATGCC---------GGGAGGCGC--------GGGCCGAGCCGTGGCGCAGCAGGGATGGGGGCCTGGAA--GGGTGCCCTGAACGAGGAATTCCTAGTAGC--------CGCGGG-TCACCAAGCCGCGGCGAATGAGT-CCCTGTTCTTTGTACACACCGCCCGTCACTACCTAAGATGGATGTGCAGGCGAGGAGGCT----------------------------------------GGGCAGGCACCCGAGCCTGTGCAACTAGATAAGGTAT--------------------------------------------------------------------------------------------------------------------------------------------------------------------------------------

>>KR871372_DE_Gpul_Msp-RR1_GRA-15_1092pb_

----------------------------------------------------CAGCGCGCGAGCTT------------------------ACGCGGAG-CGGCGGACAGCTCAG-TAATACAGTTACGATTTACCCGGGGC---AGCA-AA-------CGG-ATAACCGTGGTAA-GCTACGGCTAAGACGT--GGACGCGCTGCGCG------G------GGGTGAGGCCGGCGCAGCGGACAG------------------------------------------------------------------GACACGGCCGCGGCTAAGTTTCTGGCCTATCAGCTATTGGAAGGTAGGGTAAGGGCCTACCTTGGCGATGACGGGTGACGGGGGATGAGAGTCCGATTCCGGAGAGGGAGC-CTGAGAAACAGCTCCCACGTCTAAGGATGGCAGCAGGCGCGAAACTTACCCAATC--------TGCGGGAGGTAGTGAAGAGACGTG--GG------------------------------CACGAGGC---------CGCACACCAAAGACGTGC---GGGCCCGACT---GGAGGGCAAG-TCTGGTGCCAGCAGCCGCGGTAATTCCAGCTCCAGGAGCGCACATACACGTTGCTGCAGTTAAAACGTCCGTAGT--TGGCGCACG-------------------------------------------------------------------------------------GCACTGTGAGAC---------------------------ACGC------ATAG---CTCAAGA--------------TATG----GAGCAGGCATGGGACGCCG------------------------------------------GCGACACAGGAACAGATGATGGCAATAGGATTGCGCAGTTAGAGGTGAAAAT-CGAGGACCTG-CGCAGGACTATCGGAAGCGAAGGCGATTGCCAAGGATGTCTCCGTTGATCAAGGACTTAAGCCGGA-GGATCAAAGATGATT--AGATACCGTCGTAGTTC-CG-GCTGTAAACGATGCCGACTAGG-TGAGGCC------------------------------------------------------------GCGGGGCTGT-GCCTTAGGAGAAATCA--AGAGTGTTTGGGCTCTGGGGATAGTACGGTCGCAAGACAGAAACTTAAA--GAAATTGACGGAAGGACACCACAAGGAGTGGAGTGTGCGGCTTAATTTGACTCAACGCGGG-GCAACTTACCAGGGCAC-GTGGACG-GGCG-ATTGGG--------TGCCTG-GGCGGCGACCAAGATGGTCCACGGAGTGGTGCATGGCCGTTTGCAACACGTGGGGTGACCTG------------------------------------------TCTGGTTAATTCCGAC-AACGCGTGAGGCCGCAGACA--------------------------------------CAAGCGTTGTGTG-ACGGGCTGTGGCAAGCAGCAGGAGGC--GCGGCGATAACA-GGTCAGTGATGCCCTCGGATGCTCTGGGCTGCACGCGCACTACACTGCCCG---------------------------------------------------------------------------------------------------ACGCAGACGCGGA----------------------------------------------------------------------------------------------------------------------------------------------------------------------------------------------------------------------------------------------------------------------------------------------------------------------------------------------------------------------------------

>>TAP-4R_HU_Groe_Msp-VA_ADR-18_248pb_TAP-4R-VA

------------------------------------------------------------AAGCTT------------------------ACGCGGAG-CGGCGGACAGCTCAG-TAATACAGTTACGATTTACCCGGGGC---AGCA-AA-------CGG-ATAACCGTGGTAA-GCTACGGCTAAGACGT--GGACGCGCTGCGCG------G------GGGTGAGGCCGGCGCAGCGGACAG------------------------------------------------------------------GACACGGCCGCGGCTAAGTTTCTGGCCTATCAGCTATTGGAAGGTAGGGTAAGGGCCTACCTTGGCGATGACGGGTGACGGGGGATGAGAGTCCGATTCCGGAGAGGGAGC-CTGAA-----------------------------------------------------------------------------------------------------------------------------------------------------------------------------------------------------------------------------------------------------------------------------------------------------------------------------------------------------------------------------------------------------------------------------------------------------------------------------------------------------------------------------------------------------------------------------------------------------------------------------------------------------------------------------------------------------------------------------------------------------------------------------------------------------------------------------------------------------------------------------------------------------------------------------------------------------------------------------------------------------------------------------------------------------------------------------------------------------------------------------------------------------------------------------------------------------------------------------------------------------------------------------------------------------------------------------------------------------------------------------------------------------------------------------------------------------------------------------------------------------------------------------------------------------------------------------------------------------------------------------------------------------------------------------------------------------------------------------------------------------------------------------------

>>DQ521753_CA_Corophium_volutator_Msp-C81_MAU-07_894pb_

-------GCCCTTGTTGATTCTGCCTGACGTGTGTGCTAGCCTTGAAGATTAAGCCATGCAAGCTA--------GGGAAGC------AATATGTGGAC-CGGCGAACAGCTCAG-TAAGACAGTTATAATTTACTCGTA-----GATC-CA-------AGG-ATAACCATGGTAA-GCTATGGCTAAAACAG--GAATAG---------------------AGCCGAAACAACAACGGCTTGAAG------------------------------------------------------------------AAAAACTCTACGACTAAGTTTCTGGCCTATTAGC--TA-GTAGGTAGTGTAAGGGACTACGTAGGCAGTGATAGGTAACGGGAAATGATCGTTTGATACCGGAGAGGGAGC-CTGAGAAATAGCTCCCACGTCTAAGGATGGCAGCAGGCGCGAAAATTACCCAATGC-----AAGAAGCGAGGTAGTCAAGAGGCGTA--AA-----------------------------AAATTAAAA---------TTAACAGTAAAGATGTT----AATTGTAACT---GGAGGGCAAG-TCTGGTGCCAGCAGCCGCGGTAATTCCAGCTCCAGGAGCACATATGATAGTTGCTGCAGTTAAAAAGTTCGTAGT--TTGAGAAGA-------------------------------------------------------------------------------------AGCCTGTGAGTA---------------------------AAAT------ACAC-AACTCAAGA---------------GTG----TAATTAGCGCAGCT-AATC------------------------------------------TCTAGAGAGGAACAGAAGAAGGTGGTTGTATTGGGCAGCGAAAGGTGAAAAT-TGGCGACCTG-CTCAGGACAAACGGAGGCGAAAGCGACCACCTAGTATGTATCCGTTAATCAAGAACGTAAGCTGGA-GGATCGAAAAAGATT--AGACACCTTTGTAGTTC-CA-GCTGTAAACTATGCCGACTGGG-CGTAGGC------------------------------------------------------------GTAATTGCCT-GCGTCCTCGAGAAATCA-AGAGTTTGTGGGCTCTGGGGATAGTACGATCGCAAGATTGAAACTTAAA--GAAATTGACGGAAGGACACCACAAGGAGTGGAGTGTGCGGCTTAATTTGACTCAACGCGAA---------------------------------------------------------------------------------------------------------GGGCGAATTC---------------------------------------------------------------------------------------------------------------------------------------------------------------------------------------------------------------------------------------------------------------------------------------------------------------------------------------------------------------------------------------------------------------------------------------------------------------------------------------------------------------------------------------------------------------------------------------------------------------------------------------------------------------------------------------------------------------------------------

>>KJ019845_NL_Echinogammarus_trichiatus_Msp-H7_ARU-14_560pb_

-----------------------------------------------------------------------------------------------------------------------------------------------------------------------------------------------------------------------------------------------------------------------------------------------------------------------------------------------------------------------------------------------------------------GTTTGATACCGGAGAGGGAGC-CTGAGAAATAGCTCCCACGTCTAAGGATGGCAGCAGGCGCGAAAATTACCCAATGC-----AAGTAGTGAGGTAGTTAAGAGGCGTG--CA---------------------------ATTTGCACAAG---------TTAACAGTAAAGAAGTT----AATGGAAACT---GGAGGGCAAG-TCTGGTGCCAGCAGCCGCGGTAATTCCAGCTCCAGAAGCGCATATGACCGTTGCTGCAGTTAAAAAGTTCGTAGT--TTGAGCACA-------------------------------------------------------------------------------------TGCTTGCGAGTA---------------------------AAAT------ACAT-TACTTAAAG---------------ATG----TAATTAGCGCAACT-AATG------------------------------------------TCTAGATAGGAACAGGAGAAGGTGGTTGTATTGGGTAGCGAAAGGTGAAAAT-TGCAGACCTA-CTCAGGACAAACAGAAGCGAAAGCGGCCATCTAGAATGTATCCGTTAATCAAGAACGTAAGCTGGA-GTATCGAAAAAGATT--AGACACCTTTGTAGTTC-CA-GCTGTAAACTATGCCGACTAGG-TGAGAGA------------------------------------------------------------AATATTCTTG-TGCCTTAGGAGAAATCA-AGAGTTTGTGGGCTCTGGGGATAGTACGATCGCAAGATTGAAACTTAAA--GAAATT-----------------------------------------------------------------------------------------------------------------------------------------------------------------------------------------------------------------------------------------------------------------------------------------------------------------------------------------------------------------------------------------------------------------------------------------------------------------------------------------------------------------------------------------------------------------------------------------------------------------------------------------------------------------------------------------------------------------------------------------------------------------------------------------------------------------------------------------------------------------------------------------------------------

>>KR871374_DE_Gtig_Msp-BPAR3_GRA-15_1132pb_

--------------------------------------------AAGCATTAA-------------------------------------TTGTGGAC-CAACGAACAGCTCAG-TAACACAGTTATAATTTACTCGTA-----GATC-AA-------AGG-ATAACCATGGTAA-GCTATGGTTAAAACAA--GAATTAGTG------------------GTTTTCTAATAGGAAACAACAAAG------------------------------------------------------------------AAAAACTCTACGACTAAGTTTCTGGCCTATTAGC--TA-GTAGGTAGTGTAAGGGACTACCTAGGCTGTGATGGGTAACGGGAAATGATCGTTTGATACCGGAGAGGGAGC-CTGAGAAATAGCTCCCACGTCTAAGGATGGCAGCAGGCGCGAAAATTACCCAATGC-----AAGTAGTGAGGTAGTTAAGAGGCGTG--CA---------------------------ATTTGCACAAG---------TTAACAGTAAAGAAGTT----AACGGAAACT---GGAGGGCAAG-TCTGGTGCCAGCAGCCGCGGTAATTCCAGCTCCAGAAGCGCATATGACCGTTGCTGCAGTTAAAAAGTTCGTAGT--TTGAGCACA-------------------------------------------------------------------------------------TGCTTGCGAGTA---------------------------AAAT------ACAT-TACTTAAAG---------------ATG----TAATTAGCGCAACT-AGTG------------------------------------------TCTAGATAGGAACAGGAGAAGGTGGTTGTATTGGGTAGCGAAAGGTGAAAAT-TGCAGACCTA-CTCAGGACAAACAGAAGCGAAAGCGGCCATCTAGAATGTATCCGTTAATCAAGAACGTAAGCTGGA-GTATCGAAAAAGATT--AGACACCTTTGTAGTTC-CA-GCTGTAAACTATGCCGACTAGG-TGAGAGA------------------------------------------------------------AATATTCTTG-TGCCTTAGGAGAAATCA-AGAGTTTGTGGGCTCTGGGGATAGTACGATCGCAAGATTGAAACTTAAA--GAAATTGACGGAAGGACACCACAAGGAGTGGAGTGTGCGGCTTAATTTGACTCAACACGCG-AAAACTTACCAAGACCA-AATCGGA-AGAG-AAAGTC--------ATAACA-GAGTTATTACTTGATACCGACAAAAGTAGTGCATGGCCGTTTTCAACACGTGGTGTGAACT-------------------------------------------TATGGTTAATTCCATC-AACGCGTGAGGCCTCTTAA-----------------------------------------------------ACGGATAGCGTCAAGCTATAGGAAGT--GAGGCTATAACA-GGTCAGTGATGCTCTTAGATGTTTTGGGCTGCACGCGCACTACAATTGCGA-------------CACGAAGGGAACCGA------------------AAGGTTTTATATAAAGGTCGATATTAGCAGGGATAAACCGTTGTAA--ATCGGTTTTGAACAAGG-----------------------------------------------------------------------------------------------------------------------------------------------------------------------------------------------------------------------------------------------------------------------------------------------------------------------------------------------------------------------------------

>>AF439320_AU_Daphnia_sp._Gurleya_daphniae_REF-02_1294pb_

-----------------------------------GCTTGCCTTAAAGATTAAGCCATGCAAGCCA--------GCGAAGG---------TAACGGAG-CGGCGAACGGCTCAG-TAGGACAG-GGTTATCTGACCA-------CCGA-AC-------AGG-ATAACCGCGGTAA-TCTGTGGCTAATACGT------------------------------------------------------------------------------------------------------------------------------GTGTGGGTGAGACTCTGCCCTATCAGC--TT-GGGGGTACGGTAAGTGCGTACCCTTGCGACGACGGGTAACGGGAAATCGGGGTTTGATTCCGGAGAGGGAGC-CTGAGAGATGGCTACCACGTCCAAGGATGGCAGCAGGCGCGGAAATTACCCACTTGG----AGGACCAGAGGTAGTGACGAGGCGTT--TT-------------------------------TAATGAG---------AATAGTGTAAAAAGCTGTTC-GAAAGCAACT---GGAGGGCAAG-TCTGGTGCCAGCAGCCGCGGTAATTCCAGCTCCAGGAGCGTCTGCGTGTGTTGCTGCGGTTAAAGAGTGCGTAGT--CGGGTTATT-------------------------------------------------------------------------------------TGCCAGCAATAA---------------------------AGGT------GCAG-TCTCTAACT---------------GTA------TATTGTGCTGGT-ACTT------------------------------------------TGATCTAGGAACTGGATAAAGGTTATGGTATTCGACAGCGAGGGGTGAAAAT-TAATGACCTG-TTGAGGAGCGACTGATGCGAAAGCGGTAATCTATGACGGGTTTGACGATAAAGCGCGTAGGCAGGA-GTATCGAAGGCGATT--AGAGACCGTCGTAGTTCTTT-GCAGTAAACGATGCCAACAGGA-ATGCAAG-----------------------------------------------------------------TGCTG-TGTTCCTAGGGAAACT---GAGTGTATGGGCTCTGGGGATAGTACAGGCGCAAGCTAGAAACTTGAA--GAAATTGACGGAAGGACACCACAAGGAGTGGAGTGTGCGGGTTAATTTGACTCAACGCGGG-ACAACTTACCAGGGTAT-GTGACAT-TGAGCGAAGAG-----CATGGATGC-GATCTGTGTTCTGATGATGTTACGTGTGGTGCATGGCCGTTCTTAACACGTGGGGTGACCTG------------------------------------------TCTGGTTAAATCCGAT-AACGCGTGAGACCCTGGATA------------------------------------------------ATG-ACGATACTTGACAAGAGTAGGGAAGCTCGGGTCGATAACA-GGTCTGTGATGCCCATAGATATTCTGGGCGCCACGCGCACTACATTGGGGA------------------TGGTTACGTG--------------------------AAACGGAACATCCTTTCGGTTGGGATTGACGTTTGTAA--ATACGTCATGAACTCGGAATTCCTAGTAAT--------TTGTTG-TCATTAACGACATATGAATATGT-CCCTGTTCTTTGTACACACCGCCCGTCGTTATCTAAGATGAATGTGCGGATGAAGTTGGT------------------------------------------TAGGCCAAAAGAATCCGTGCGATTAGATTGGATACAA-----GTCGTAACAAGG-TT--ACTGTAGGAG------------------------AACCTGCAGTAGGATCAATAACA-----------------------------------------------------------------------------------------------------

>>AJ302319_UK_Daphnia_magna_Hamiltosporidium_magnivora_CAN-01_1304pb_

-------CACCAGGTTGATTCTGCCTGACGAGGATGCTTGTCTCTGGGATTAAGCCATGCAAGTCT-------GGTGAAGC-------GAAAGTGGAA-CTGCGAACGGCTCAG-TAGAACGGTGATTATTTAATCGT------GTAG-GA-------AGG-ATAACCGCGGGAA-ACTGTGGCTAATAA---------------------------------------------------------------------------------------------------------------------------------CACGAGTAAGACGCCGACCCATCAGT--TT-TATCGTACGGTAAGGGCGTACGATGGCTTTAACGGGT-ACGGGGGATCAGGGTTTGATTCCGGAGAGGGAGC-CTGAGAAACGGCTACCAGGTCTAAGGACAGCAGCAGGCGCGAAACTTGCCCAATT--------GGAGAGAGGCAGTTATGAGACGTA--TA-------------------------------TTTTGTA---------ACCGGGGTAGAATACCG----GTGATTGACT---GGAGGGCAAG-TCTGGTGCCAGCAGCCGCGGTAATTCCAGCTCCAGGAGTGTCATAGTGCATTGCTGCATTTAAAAGGTCCGTAGT--CAGTGTTGA-----------------------------------------------------------------------------------CAGGGATGCTAGA----------------------------AGAC------ACTT-TCTTCATGG--------------AGTG-----TATTGGCATTCTA-GGTT------------------------------------------AGCATGTGAGAGCGGAAGAGGGCGACTGTATTGCGTAGCGAGAGGTGAAAGT-TGACGACCTA-CGTAGGACAAACCGAAGCGAAAGCTGTCGTCCAGTACGTAGCCGGTGATCAAGGACGTAAGCCGGA-SGAGCAAAGGTGATT--AAAGACCCCTGTAGTTC-CG-GCCGTAAACTATGCCAACTGGG-TGTTACG-----------------------------------------------------------------TAGTG-GTACCCACGAGAAATC---AAGTATATGGGCTATGGGGATAGTNCGATCGCAAGATTGAAACTTGAA--GAAGTTGACGGAAGGACACCACCAGGAGTGGAATGTGCGGCTTAATTTGACTCAACGCGGGCACAACTTATCAGGGCCA-GTTGTAT-GACG-AATCTA--------TGATGA--ATAC--------------GACTAAGTGGTGCATGGCCGTTCACAACATATGAGGCGACTT-------------------------------------------TTAGGTTTATTGCTGT-AATGTGTGAGACCCTCAGCT---------------------------------------------ACGGCG-ACTGGGGACTATAAGTTTCAGGAAGGAGGGGGCTATAACA-GGTCTGTGATGCCCTTAGATGTTCTGGGCTGCACGCGCACTACAATGTTAT-T-----------TCTAGGTATATTGTA--------------------------AAGATAAAGGATAACTCGGTTGGGATTGCGTTCTGTAA--TGGACGCATGAACCAGGAATTCCTAGTAGT--------CGCGTG-TCACTAACGGGCGACGACTGCGT-CCCTGTTCTTTGTACACACCGCCCGTCGTTATCTAAGATGGTGCTATGTTCGAAGAGGGT-------------------------------------------TTTCCTTCTGAGGACGTAGGATTAGATTGGATACAA-----GTCGTAACAAGG-TT--GCTGTAGGAG------------------------AACCAGCAGCAGGATCATAA--------------------------------------------------------------------------------------------------------

>>AF185989_US_Rainbow_trout_Nucleospora_salmonis_GRE-00_1250pb_

--------------------------GACGTGGGTGCTAGCCTCTAAGATTTAGCCATGCATGCTT-------TGTGAACC-----CAGACGGGGGAA-CGGCGAACGGCTCAG-TAATGTTGCGGTGATCTGCTCTGG-----TTGA-GA-------AAG-ATAACCACGGTAA-ACTGTGGCTAAGAGA--------------------------------------------------------------------------------------------------------------------------------CCAGAATGAGCTGCAACCCTATCAGT--TT-GTTGGAAGTGTAAAAGACTCCCAAGACGTCGACGGGTAACGGGAAATCAGTGTTTGATTCCGGAGAGGGAGC-CTGAGAGATGGCTCCCACGTCCAAGGATGGCAGCAGGCGCGAAAATTGTCCACTCTT----TGGAGAGGAGACAGTTATGAGGCGTG--AT-----------------------------T-AAAAGAG---------CGAAGTGTAAACACCTTCGTGTAACGCAATT---GGAGGGCAAGTTTTGGTGCCAGCAGCCGCGGTAATTCCAACTCCAAGAGTGCCCATGGTGGATGCTGCAGTTAAAAAGTCCGTAGT--CGTAG--------------------------------------------------------------------------------------------ATGCAATTA---------------------------AAGG------GTGC-GGATCAAGC---------------GCA-------CTGAGTTTGT---TGA------------------------------------------GTTGTAGCGGAGCGGATAGGGAGCATGGTATAGGTTGGCGAAGAATGAAATC-TCAAGACCCA-ACCTGGACCAACTGAGGCGAAAGCGGTGCTCTTATACGCATCCGAGGATCAAGGACGAAGGCTGGA-GGATCGAAAGTGATT--AGATACCGCTGTAGTTC-CA-GCAGTAAACTATGCCGACATTC-TCTGTCC-------------------------------------------------------------AG--CGGAC-GGGGAAGGGAGAAATC--TTAGTTTTCGGGCTCTGGGGATAGTACGCTTGCAAGAGTGAAACTTAAAGCGAAATTGACGGAAGGACACTACCAGGAGTGGATTGTACTGCTTAATTTGACTCAACGCGGG-AAAACTTACCAGGGTCA-AGTTCTC-CGTA-GATCGA------GTACGTGA-GGGGA--------------ACGAGAGTGGTGCATGGCCGTTGGAAATTGATGGGATGACTT-------------------------------------------TTAGCTTAAATGCTGA-AACCAGTGAGATCTTC----------------------------------------------------TAG-ACAGGTGCCA-TCCGGCACAGGAGGGCGAAGGCGATAACA-GGTCCGTGATGCCCTTAGATATCCTGGGCTGCAAGTGCAATACAATATCCA-------------TGTC---GATCAAGA----------------------------AAGGTGATTATGGATGAGCAGGATTAGCTCTTGTAA--ATGAGCTATGAATGAGGAATTCCTAGTAAC--------AGTGTC-TCACTAAGGCATTGTGAATGCGT-CCCTGTTCTTTGTACACACCGCCCGTCACTATTTCAGATGGCCATGAAGATGAAGAGCTT---------------------------------------------CGGTTCTGAATATTCATGTCTAGATAAAGTACAA-----GTCGTAACAAGGTTGC---------------------------------------------------------------------------------------------------------------------------------------------------------------

>>HE584634_UK_Cancer_pagarus_Enterospora_canceri_STE-11_816pb_

------------------TTTNNNNNNACGTAGATGCTAGTCTTTAAGATTAAGCCATGCATGCTA-------AGTGAACC-----TGTACGGGGGAA-CGGCGAACGGCTCAG-TAATGTTGCGGTGATTTGCTCTAT-----ATGA-TA-------AGA-ATAACCACGGTAA-CCTGTGGCTAAAAGT--------------------------------------------------------------------------------------------------------------------------------GTAGAATAAGGCGCAATCCTATCAGT--TT-GTTGGTAGTGTAAAGGACTACCAAGACTACGACGGGTGACGGGAAATCAGGGTTTGATTCCGGAGAGGGAGC-CTGAGAGATGGCTCCCACGTCCAAGGACGGCAGCAGGCGCGAAACTTATCCACTCTT----CTGAGAGGAGATAGTTATGAGACGTG--AT-----------------------------T--TAAGGG---------TTGAGTGTAAAGAACTCGACGTGAAGCAATT---GGAGGGCAAGTTTTGGTGCCAGCAGCCGCGGTAATACCAACTCCAAGAGTGTCTATGGTGGGTGCTGCAGTTAAAGGGTCCGTAGT--CGTAG--------------------------------------------------------------------------------------------ATGCAATTA---------------------------AAGG------GTGG-TGCTCAAGA---------------CCA-------TTAAGTTTGT----TG------------------------------------------TTTTTAGCGGAACGGATAGGGAGCGTGGTATAGGCGGGCCAAGAATGAAATC-TCAAGACCCC-GCCTGGACCAACTGAGGCGAAAGCGACGCTCTTAGACGTATCTAAGGATCAAGGACGAAGGCTAGA-TTATCGAAAGTGATT--AGACACCGCTGTAGTTC-TA-GCAGTAAACGATGCCGACAGCT-CCTGCTG----------------------------------------------------------CGAAA--GTGGT-GGGAGCAAGAGAAATC--ATAGTTTTCGGGCTCTGGGGATAGTACACTCGCAAGAGTGAAACTTAAAGCGAGATTGACGGAAGAACACTACCAGGAGTGGATTGTGCGGCTTAATTTG----------------------------------------------------------------------------------------------------------------------------------------------------------------------------------------------------------------------------------------------------------------------------------------------------------------------------------------------------------------------------------------------------------------------------------------------------------------------------------------------------------------------------------------------------------------------------------------------------------------------------------------------------------------------------------------------------------------------------------------------------------------------------------------------------------------------------------------------------------------

>>AJ438964_GB_Gpul_Msp-JES2002I_TER-04_1250pb_

------------------------------TAGATGCTAGTCTCTAAGATTTAGCCATGCATGCTT-------AGTAT----------------ATAA-CGGCGAACGGCTCAG-TAATGTTGCGATGATTTGGTCTTT-----TTGG-GA-------TAGAATAACTACGGTAA-GCTGTAGCTAAAAAT--------------------------------------------------------------------------------------------------------------------------------AAAGAATAAGTCGCAACCCTATCAGT--TA-GTTGGTAGTGTAAAGGACTACCAAGACAATGACGGGTGACGGGAAATTAGGGTTTTATTCCGGAGAGGGAGC-CTGAGAGATGGCTCCCACGTCCAAGGACGGCAGCAGGCGCGAAAATTGCCCACTCTT----TTAAGAGGAGGCAGTTATGAGACGTA--TA-----------------------------A--ATAGGA---------AATATTATAAAAAGATA----TAAAAGAATT---GGAGGGCAAG-TTTGGTGCCAGCAGCCGCGGTAATACCGACTCCAAGAGTGTCTATGGTGGATGCTGCAGTTAAAAAGTCCGTAGTTTTATAA--------------------------------------------------------------------------------------------ATGCAATTA---------------------------AAAA------ACGT-AGATAAAAT---------------ACG-------TTTAGTTTGT----TA------------------------------------------AGTATAGCGAAACGGGTAGAGTGCATAGTATAGATAGGCGAGAGATGAAATTACCAAGACCCT-ATCTGGACTAACGGAGGCGAAAGCGATGTACTTAAACGAATTTAAGGATCAAGGACGAAGGCTGGA-GGATCGAAAGTGATT--AGATACCGCTGTAGTTC-CA-GCAGTAAACGATGCCGACAAGT-AAGATA-----------------------------------------------------------CTGTG--GAGTA-TTTACTGGGAGAAATT--TTAGTTTTCGGGCTCTGGGGATAGTACGCTCGCAAGGGTGAAACTTAAAGTGAAATTGACGGAACTACACTACCAGGAGTGGATTGTGCGGCTTAANNNNNNNNNNCGCGAG-GAAACTTACCAGGCCCT-TTTGCAC-CTAA-GAT-------------ACGA-GGTGC--------------GAAGGAGTGGTGCATGGTCGTTGGAAATTCATGGGATGACTT-------------------------------------------TGAGCTTAAATGCTTG-AATGAGTGAGA-CTCC---------------------------------------------------GTAG-ACTGGCTGCG-TTAAGCAGCAGGAGGGTGGAGCTATAACA-GATCAGTGATGCCCTTAGATGGCCTGGGCTGCACGCGCAATACAGTGGGAC-G-----------TAGGTATAGA-GACG----------------------------TACAGGTCCCCA-----GATGGATTGCCGATTGTAA--GTTCGGCATGAAAGCGGAATTCCTAGTAAG--------CACGTA-TTATCAATGGGTGCTGAATGCGT-CCCTGTAGCTTGTACACACCGTCCGTCAGTATCTCAGATGTTTTTTAGGATGAAGAGTTT---------------------------------------------CGATTCTGAATAATGAAAAGTAGATAAGATGTAA-----GTCGTAGCAAGGTTTCGGTGGG---AG------------------------AACCAGCCGAAGGATCA-----------------------------------------------------------------------------------------------------------

>>GRR-05_FR_Groe_Msp-IVB_ADR-18_758pb_GGR-05-IVB

-------------------------------------------------------------TGCTT-------AGTAT----------------ATAA-CGGCGAACGGCTCAG-TAATGTTGCGATGATTTGGTCTTT-----TTGG-GA-------TAGAATAACTACGGTAA-GCTGTAGCTAAAAAT--------------------------------------------------------------------------------------------------------------------------------AAAGAATAAGTCGCAACCCTATCAGT--TA-GTTGGTAGTGTAAAGGACTACCAAGACAATGACGGGTGACGGGAAATTAGGGTTTTATTCCGGAGAGGGAGC-CTGAGAGATGGCTCCCACGTCCAAGGACGGCAGCAGGCGCGAAAATTGCCCACTCTT----TTAAGAGGAGGCAGTTATGAGACGTA--TA-----------------------------A--CTAGGA---------AATATTATAAAAAGATA----TAAAAGAATT---GGAGGGCAAG-TTTGGTGCCAGCAGCCGCGGTAATACCGACTCCAAGAGTGTCTATGGTGGATGCTGCAGTTAAAAAGTCCGTAGTTTTATAA--------------------------------------------------------------------------------------------ATGCAATTA---------------------------AAAA------ACGT-AGATAAAAT---------------ACG-------TTTAGTTTGT----TA------------------------------------------AGTATAGCGAAACGGGTAGAGTGCATAGTATAGATAGGCGAGAGATGAAATTACCAAGACCCT-ATCTGGACTAACGGAGGCGAAAGCGATGTACTTAAACGAATTTAAGGATCAAGGACGAAGGCTGGA-GGATCGAAAGTGATT--AGATACCGCTGTAGTTC-CA-GCAGTAAACGATGCCGACAAGT-AAGATA-----------------------------------------------------------CTGTG--GAGTA-TTTACTGGGAGAAATT--TTAGTTTTCGGGCTCTGGGGATAGTACGCTCGCAAGGGTGAAACTTAAAGTGAAATTGACGGAACTACACTACCAGGAGTGGATTGTGCGGCTTAATT------------------------------------------------------------------------------------------------------------------------------------------------------------------------------------------------------------------------------------------------------------------------------------------------------------------------------------------------------------------------------------------------------------------------------------------------------------------------------------------------------------------------------------------------------------------------------------------------------------------------------------------------------------------------------------------------------------------------------------------------------------------------------------------------------------------------------------------------------------------

>>KR871371_DE_Gpul_Msp-I_GRA-15_1069pb_

----------------------------------------------------------------------------------------------------GGCGAACGGCTCAG-TAATGTTGCGATGATTTGGTCTTT-----TTGG-GA-------TAGAATAACTACGGTAA-GCTGTAGCTAAAAAT--------------------------------------------------------------------------------------------------------------------------------AAAGAATAAGTCGCAACCCTATCAGT--TA-GTTGGTAGTGTAAAGGACTACCAAGACAATGACGGGTGACGGGAAATTAGGGTTTTATTCCGGAGAGGGAGC-CTGAGAGATGGCTCCCACGTCCAAGGACGGCAGCAGGCGCGAAAATTGCCCACTCTT----TTAAGAGGAGGCAGTTATGAGACGTA--TA-----------------------------A--ATAGGA---------AATATTATAAAAAGATA----TAAAAGAATT---GGAGGGCAAG-TTTGGTGCCAGCAGCCGCGGTAATACCGACTCCAAGAGTGTCTATGGTGGATGCTGCAGTTAAAAAGTCCGTAGTTTTATAA--------------------------------------------------------------------------------------------ATGCAATTA---------------------------AAAA------ACGT-AGATAAAAT---------------ACG-------TTTAGTTTGT----TA------------------------------------------AGTATAGCGAAACGGGTAGAGTGCATAGTATAGATAGGCGAGAGATGAAATTACCAAGACCCT-ATCTGGACTAACGGAGGCGAAAGCGATGTACTTAAACGAATTTAAGGATCAAGGACGAAGGCTGGA-GGATCGAAAGTGATT--AGATACCGCTGTAGTTC-CA-GCAGTAAACGATGCCGACAAGT-AAGATA-----------------------------------------------------------CTGTG--GAGTA-TTTACTGGGAGAAATT--TTAGTTTTCGGGCTCTGGGGATAGTACGCTCGCAAGGGTGAAACTTAAAGTGAAATTGACGGAACTACACTACCAGGAGTGGATTGTGCGGCTTAATTTGACTCAACGCGGG-AAAACTTACCAGAACCA-AATTTAG-TAAA-GATTGA------AAACATGA-ACTAA--------------GTAAAAGTGGTGCATGGTCGTTGGAAATTGATGGGATGACTT-------------------------------------------TCAGCTTAAATGCTAT-AATTAGTGAGATCTTT---------------------------------------------------ATAG-ACAGGTATTT-AATAATACAGGAAGGAGAAGGCTATAACA-GATCAGTGATGCCCTTAGATGATCTGGGCTGCACGCGCAATACAATAATTA-A-----------TAGA---AAG-GAAA----------------------------GATAAATTTTTGATTACGAGGGATTAGTTTTTGTAA--ATGAACTATGAACGAGGAATTCCTAGTAAC--------GATAAT-TCATTAAGTTATG------------------------------------------------------------------------------------------------------------------------------------------------------------------------------------------------------------------------------------------------------------------------------------------------------------------------------------------

>>KT762153_CZ_Daphnia_pulex_Globulispora_mitoportans_VAV-16_1257pb_

-------CACCAGGTTGATTCTGCCTGACGTAGATGCTAGTCTCTGAGATTTAGCCATGCATGCTT-------AATAT----------------ATAA-TGGCGAACGGCTCAG-TAATGTTGCGATGATTTGCTCTTG-----TTGA-TG-------TAG-ATAACCACGGTAA-GCTGTGGCTAATAGT--------------------------------------------------------------------------------------------------------------------------------GAAGAATAAGACGCAACCCTATCAGT--TA-GTTGGTAGTGTAAAGGACTACCAAGACGATGACGGGTGACGGGAAATCAGGGTTTGATTCCGGAGAGGGAGC-CTGAGAGATGGCTCCCACGTCCAAGGACGGCAGCAGGCGCGAAAATTGCCCACTCTT----TTATGAGGAGGCAGTTATGAGACGTA--AG-----------------------------A--TTAAGC--------GGGCACAGTAAACAAGTGC---TTAGTTCATT---GGAGGGCAAG-TTTGGTGCCAGCAGCCGCGGTAATACCGACTCCAAGAGTGTCTATGGTGGATGCTGCAGTTAAAAAGTCCGTAGTTGTATGT--------------------------------------------------------------------------------------------ATGCAATTA---------------------------AAAA------GCAT-GGTTAAAGC---------------GTG-------TTTAGTTTGC----TA------------------------------------------AAGGTAGCGGAACGGGTAGAGTGCATAGTATAGATAGGCGAGAGATGAAATGGCCAAGACCCT-ATCTGGACTGACGGAGGCGAAAGCGATGTACTTAAACGAATCTGGGGATCAAGGACGAAGGCTGGA-GGATCGAAAGTGATT--AGATACCGCTGTAGTTC-CA-GCAGTAAACGATGCCGACAAGC-AGGACA-----------------------------------------------------------C--GG--GAGTG-TTTGCTGGGAGAAATC--TTAGTTTTCGGGCTCTGGGGATAGTACGCTCGCAAGGGTGAAACTTAAAGTGAAATTGACGGAACTACACTACCAGGAGTGGATTGTGCGGCTTAATTTGACTCAACGCGGG-AAAACTTACCAGGACCA-AGCTTAG-TGGA-GATCGA-----CT-ACGTGA-ACTAA--------------GCAGAAGTGGTGCATGGTCGTTGGAAATTGATGGGATGACTT-------------------------------------------TCAGCTTAAATGCTAT-AATTAGTGAGATCTTT---------------------------------------------------GTAG-ACAGGTGCAT-AATGGTACAGGAAGGAGAAGGCGATAACA-GATCAGTGATGCCCTTAGATGCCCTGGGCGGCACGCGCAATACAATACTTG-A-----------T-GA---TTA-TGCA----------------------------GATAGATTTTGAAGTGCGAGGGATTAGTTTTTGTAA--ATGAACTATGAACGAGGAATTCCTAGTAAC--------GATAAT-TCATTAAGTTATGGTGAATGAGT-CCCTGTAGTTTGTACACACCGCCCGTCACTATCTCAGATGGTTATGAAGATGAAGAGCCT---------------------------------------------CGGTTCTGAATAGTCATAGCTAGATAAAGTACAA-----GTCGTAACAAGG-TT--GCG-----------------------------------------------------------------------------------------------------------------------------------------------------------

>>KX137915_DE_Sericostoma_sp._Msp-3_GRA-17_397pb_

---------CCAGGTTGATTCTGCCTGACGTAGATGCTAGTCTCTAAGATTTAGCCATGCATGCTT-------AATAT----------------AGAA-TGGCGAACGGCTCAG-TAATGTTGCGATGATTTGCTCTTG-----ATGG-CG-------TAG-ATAACCACGGTAA-GCTGTGGCTAATAGG--------------------------------------------------------------------------------------------------------------------------------CAAGAATAAGACGCAACCCTATCAGT--TA-GTTGGTAGTGTAAAGGACTACCAAGACGATGACGGGTGACGGGAAATGAGGGTTTGATTCCGGAGAGGGAGC-CTGAGAGATGGCTCCCACGTCCAAGGACGGCAGCAGGCGCGAAAATTGCCCACTCTT----TGGTGAGGAGGCAGTTATGAGACGTA--AG-----------------------------T--TTAAGC---------AGCACAGTAAACAAGTGC---GTATTTGATT---GGAGGGCAAG-TTTGGTGCCAGCATCCGCGGCAATA------------------------------------------------------------------------------------------------------------------------------------------------------------------------------------------------------------------------------------------------------------------------------------------------------------------------------------------------------------------------------------------------------------------------------------------------------------------------------------------------------------------------------------------------------------------------------------------------------------------------------------------------------------------------------------------------------------------------------------------------------------------------------------------------------------------------------------------------------------------------------------------------------------------------------------------------------------------------------------------------------------------------------------------------------------------------------------------------------------------------------------------------------------------------------------------------------------------------------------------------------------------------------------------------------------------------------------------------------------------------------------------------------------------------------------------------------------------------------------------------------------------------------------------------------------------------

>>GR38-13_GR_Groe_Msp-IVC_ADR-18_330pb_GR38-13-IVC

----------------------------------------------------------GCATGCTT-------AATA-----------------GAAA-TGGCGAACGGCTCAG-TAATGTTGCGATGATTTGCTCTTG-----ATGG-TA-------CAG-ATAACCACGGCAA-GCTGTGGCTAAGAGG--------------------------------------------------------------------------------------------------------------------------------CAAGAATAAGACGCAACCCTATCAGT--TA-GTTGGTAGTGTAAAGGACTACCAAGACGACGACGGGTGACGGGAAATGAGGGTTTGATTCCGGAGAGGGAGC-CTGAGAGATGGCTCCCACGTCCAAGGACGGCAGCAGGCGCGAAAATTGCCCACTCTT----TGGTGAGGAGGCAGTTATGAGACGTA--GA--------------------------------TTAGAG---------GGCACAGTAAACAAGTGC---TTTGAGTATT---GGAGGGCAAG-TTTGGTGCC----------------------------------------------------------------------------------------------------------------------------------------------------------------------------------------------------------------------------------------------------------------------------------------------------------------------------------------------------------------------------------------------------------------------------------------------------------------------------------------------------------------------------------------------------------------------------------------------------------------------------------------------------------------------------------------------------------------------------------------------------------------------------------------------------------------------------------------------------------------------------------------------------------------------------------------------------------------------------------------------------------------------------------------------------------------------------------------------------------------------------------------------------------------------------------------------------------------------------------------------------------------------------------------------------------------------------------------------------------------------------------------------------------------------------------------------------------------------------------------------------------------------------------------------------------------------------------------

>>KX757849_CA_Carcinus_maenas_Parahepatospora_carcini_BOJ-16_936pb_

---------------------------------------------------------------------------------------------------------------------------------------------------------------------------------------------------------------------------------------------------------------------------------------------------------------------------------------------------------------------------------------------------------------------------------------CTGAGAGATTGCTCCTACGTCCAAGGACGGCAGCAGGCGCGAAAATTGCCCACTCTTC--TTTTGGAGGAGGCAGTTATGAGACGTG--AA-----------------------------T--AAAGGA---------AGTACAGTAAATAAGTGCTTGTAAAGCAATT---GGAGGGCAAG-TTTGGTGCCAGCAGCCGCGGTAATACCGACTCCAAGAGTGTCTATTGTGGATGCTGCAGTTAAAAAGTCCGTAGTT-TTTTT--------------------------------------------------------------------------------------------GTGCAATTA---------------------------AAAG------GTAT-TGATCAAAG---------------ATA-------CCAAGTTTGCA-ACAA------------------------------------------TATTTTTCGGAACGGATAGGGAACATAGTATAGATGGGCGAGAGATGAAATGACCAAGACCCC-ATCTGGACTAACAGAGGCGAAAGCGATGTTCTTAGACGAATCTGTGGATCAAGGACGAAGGCCGGA-GGATCGAAAGTGATT--AGATACCGCTGTAGTTC-CG-GCAGTAAACTATGCCGACATCT-TTTCCAT------------------------------------------------------GAATTTAAT--AGTGG-GAGAGAGGGAGAAATC--TTAGTGTTCGGGCTCTGGGGATAGTATGCTCGCAAGAGTGAAACTTAAAACGAAATTGACGGAATAACACCACCAGGAGTGGATTATGCGGCTTAATTTGACTCAACGCGGG-ACAACTCACCAGGGTCA-AGTCTAT-CAAA-GATTGA------ATACATGA-GATAG--------------GCAGGAGTGGTGCATGGTCGTTGGAAATTGATGGGATGACTT-------------------------------------------TTAGCTTAAATGCTGA-AATGAGTGAGATCTTC----------------------------------------------------TAG-ACAGGTGTTT-TATAACACAGGAGGGAGAAGGCTATAACA-GATCAGTGATGCCCTTAGATATCCTGGGCTGCACGCGTAATACAATTCTGATTATTTGT-----TAGATAAATCTAATA----------------------------ATAATATTTTTCAGAGAGAGGGATTGATTCTTGTAA--ATGAATCATGAACGAGGAATTCCTAGTAAC--------AGTGTC-TCATTAAGAGATTGTGAATGTGT-CCCTGTTGTT---------------------------------------------------------------------------------------------------------------------------------------------------------------------------------------------------------------------------------------------------------------------------------------------------------------------

>>NIT26-04_IT_Groe_Msp-IVD_ADR-18_762pb_NIT26-04-IVD

----------------------------------------------------------------------------------------TTTGCGGGAA-CGACGAACGGCTCAG-TAATATTGCGATGATTTGCTCTGT-----TGGG-AG-------AAG-ATAACCATGGTAA-CCTATGGCTAAGAG---------------------------------------------------------------------------------------------------------------------------------ACAGAATAAGACGCAATCCTATCAGT--TT-GTTGGTTGTGTAATGGACAACCAAGACTATGACGGGTGACGGGAAATCAGGGTTTTATTCCGGAGAGGGAGC-CTGAGAGATTGCTCCTACGTCCAAGGACGGCAGCAGGCGCGAAAATTGCCCACTCTTC--TTTTGGAGGAGGCAGTTATGAGACGTG--AA-----------------------------T--AAAGGA---------AGTACAGTAAATAAGTGCTTGTAAAGCAATT---GGAGGGCAAG-TTTGGTGCCAGCAGCCGCGGTAATACCGACTCCAAGAGTGTCTATTGTGGATGCTGCAGTTAAAAAGTCCGTAGTT-TTTTT--------------------------------------------------------------------------------------------GTGCAATTA---------------------------AAAG------GTAT-TGATCAAAG---------------ATA-------CCAAGTTTGCA-ACAA------------------------------------------TATTTTTCGGAACGGATAGGGAACATAGTATAGATGGGCGAGAGATGAAATGACCAAGACCCC-ATCTGGACTAACAGAGGCGAAAGCGATGTTCTTAGACGAATCTGTGGATCAAGGACGAAGGCCGGA-GGATCGAAAGTGATT--AGATACCGCTGTAGTTC-CG-GCAGTAAACTATGCCGACATCT-TTTCCAT-------------------------------------------------------GTATTATT--AGTGG-GAGAGAGGGAGAAATC--TTAGTGTTCGGGCTCTGGGGATAGTATGCTCGCAAGAGTGAAACTTAAAACGAAATTGACGGAATAACACCACCAGGAGTGGATTGTGCGGCTTAA--------------------------------------------------------------------------------------------------------------------------------------------------------------------------------------------------------------------------------------------------------------------------------------------------------------------------------------------------------------------------------------------------------------------------------------------------------------------------------------------------------------------------------------------------------------------------------------------------------------------------------------------------------------------------------------------------------------------------------------------------------------------------------------------------------------------------------------------------------------------

>>AJ966719_GB_Crangonyx_pseudogracilis_Msp-CRANPB_SLO-10_737pb_

---------------------------------------------------------TGCATGCCC--------GTGA-----------CGCAAGGAA-CGGCGAACGGCTCAG-TAATACTGCGATGATCTGCTCCGC-------GC-GA-------ACG-ATAACCACGGGAA-ACTGTGGCTAAGAGC--------------------------------------------------------------------------------------------------------------------------------GCGGATTGAGACGCAGCCCTATCAGC--CT-GTTGGTAGTGTAACGGACTACCAAAGCAGCGACGGGTGACGGGAAATTAGGGTTTGATTCCGGAGAGGGAGC-CTGAGAGATGGCTCCCACGTCCAAGGACGGCAGCAGGCGCGAAAATTGCCCACTCTC----TCGCGAGGAGGCGGTTACGAGGCGTG--AC-------------------------------GGAAGGG---------CGCACTGTAAAGATGTGCGC-GAACGCGATT---GGAGGGCAAG-TTCGGTGCCAGCAGCCGCGGTAATACCGACTCCAAGAGTGTCTATGGTGGATGCTGCAGTTAAAAAGTCCGTAGT--CGGCA--------------------------------------------------------------------------------------------CTGCGACGA---------------------------AAGC------GCGC-CGTTCGATG---------------GCG-------CAGGCGTTGC----AG------------------------------------------GAGCTTGCGGAGCGGGCGGGGGGCACGGTATAGGCAGGCGAGAGATGAAATGGCCAAGACCCT-GCCTGGACCGACGGCGGCGAAGGCGGTGCCCTCGCACGCATCTGTGGATCAAGGACGAAGGCCGGA-GGATCGAAAGTGATT--AGAGACCGCTGTAGTTC-CG-GCAGTAAACGATGCCGACACCG-CGCGGCG-----------------------------------------------------------------GCGCT-GCGCGGGGGGGAAACC--TTAGTGTTCGGGCTCTGGGGATAGTATGCTCGCAAGTGTGAAAATTAAA-CGAAATTGACGGAGCTACACCACAAG----------------------------------------------------------------------------------------------------------------------------------------------------------------------------------------------------------------------------------------------------------------------------------------------------------------------------------------------------------------------------------------------------------------------------------------------------------------------------------------------------------------------------------------------------------------------------------------------------------------------------------------------------------------------------------------------------------------------------------------------------------------------------------------------------------------------------------------------------------------------------------------

>>KX137931_DE_Agap_Msp-4_GRA-17_341pb_

---------------------------------------------------------TGCATGCCC--------GTGA-----------CGC-AGGAA-CGGCGAACGGCTCAG-TAATACTGCGATGATCTGCTCCGC-------GC-GA-------ACG-ATAACCACGGGAA-ACTGTGGCTAAGAGC--------------------------------------------------------------------------------------------------------------------------------GCGGATTGAGACGCAGCCCTATCAGC--CT-GTTGGTAGTGTAACGGACTACCAAAGCAGCGACGGGTGACGGGAAATTAGGGTTTGATTCCGGAGAGGGAGC-CTGAGAGATGGCTCCCACGTCCAAGGACGGCAGCAGGCGCGAAAATTGCCCACTCTC----TCGCGAGGAGGCGGTTACGAGGCGTG--AC-------------------------------GGAAGGG---------CGCACTGTAAAGATGTGCGC-GAACGCGATT---GGAGGGCAAG-TTCGGTGCCAGCAC-----------------------------------------------------------------------------------------------------------------------------------------------------------------------------------------------------------------------------------------------------------------------------------------------------------------------------------------------------------------------------------------------------------------------------------------------------------------------------------------------------------------------------------------------------------------------------------------------------------------------------------------------------------------------------------------------------------------------------------------------------------------------------------------------------------------------------------------------------------------------------------------------------------------------------------------------------------------------------------------------------------------------------------------------------------------------------------------------------------------------------------------------------------------------------------------------------------------------------------------------------------------------------------------------------------------------------------------------------------------------------------------------------------------------------------------------------------------------------------------------------------------------------------------------------------------------------------

>>KT633994_LU_Gfos_Orthosomella-DG-2015_WEI-16_327pb_

--------------------------------------------------------------GCCC--------GTGA-----------CGCAAGGAA-CGGCGAACGGCTCAG-TAATACTGCGATGATCTGCTCCGC-------GT-GC-------ACG-ATAACCACGGGAA-ACTGTGGCTAAGAGC--------------------------------------------------------------------------------------------------------------------------------GCGGATTGAGACGCAGCCCTATCAGC--AA-GTTGGTAGTGTAACGGACTACCAAGGCGGCGACGGGTGACGGGAAATCGGGGTTTGATTCCGGAGAGGGAGC-CTGAGAGATGGCTCCCACGTCCAAGGACGGCGGCAGGCGCGAAAATTGCCCACTCTC----TCGCGAGGAGGCAGTTACGAGGCGTG--AC-------------------------------GGAAGGG---------CGCACCGCAAAGACGTGCGC-GAACGCGATT---GGAGGGCAAG-TTCG---------------------------------------------------------------------------------------------------------------------------------------------------------------------------------------------------------------------------------------------------------------------------------------------------------------------------------------------------------------------------------------------------------------------------------------------------------------------------------------------------------------------------------------------------------------------------------------------------------------------------------------------------------------------------------------------------------------------------------------------------------------------------------------------------------------------------------------------------------------------------------------------------------------------------------------------------------------------------------------------------------------------------------------------------------------------------------------------------------------------------------------------------------------------------------------------------------------------------------------------------------------------------------------------------------------------------------------------------------------------------------------------------------------------------------------------------------------------------------------------------------------------------------------------------------------------------------------------

>>BG01-01_RO_Groe_Msp-IVE_ADR-18_362pb_BG01-01IVE

-------------------------------------------CTCGAGGCTAGCCATGCATGCCC--------GTGA-----------CGCAAGGAA-CGGCGAACGGCTCAG-TAATACTGCGATGATCTGCTCCGC-------GC-GC-------ACG-ATAACCACGGGAA-ACTGTGGCTAAGAGC--------------------------------------------------------------------------------------------------------------------------------GCGGATTGAGACGCAGCCCTATCAGC--AA-GTTGGTAGTGTAACGGACTACCAAGGCGGCGACGGGTGACGGGAAATCGGGGTTTGATTCCGGAGAGGGAGC-CTGAGAGATGGCTCCCACGTCCAAGGACGGCAGCAGGCGCGAAAATTGCCCACTCTC----TCGCGAGGAGGCAGTTACGAGGCGTG--AC-------------------------------GGAAGGG---------CGCACCGCAAAGACGTGCGC-GAACGCGATT---GGAGGGCAAG-TTCGGTGCCGCCCGCCGCGG-----------------------------------------------------------------------------------------------------------------------------------------------------------------------------------------------------------------------------------------------------------------------------------------------------------------------------------------------------------------------------------------------------------------------------------------------------------------------------------------------------------------------------------------------------------------------------------------------------------------------------------------------------------------------------------------------------------------------------------------------------------------------------------------------------------------------------------------------------------------------------------------------------------------------------------------------------------------------------------------------------------------------------------------------------------------------------------------------------------------------------------------------------------------------------------------------------------------------------------------------------------------------------------------------------------------------------------------------------------------------------------------------------------------------------------------------------------------------------------------------------------------------------------------------------------------------------

>>HM991452_US_Gpse_Msp-GPM2_RYA-10_852pb_

-------ACCAGGTTTGATTCTGCCTGACGTAGATGCTCGCCTCGGAGATTAAGCCATGCATGCCC--------GTGA-----------AGATGGGAA-CGGCGAACGGCTCAG-TAATACCGCGCTCATCTGCTCTCC-------GC-GC-------AAC-ATAACCACGGGAA-ACTGTGGCTAACAGC--------------------------------------------------------------------------------------------------------------------------------GGAGATTGAGGCGCGGCCCTATCAGC--TT-GTTGGTAGTGTAACGGACTACCAAGGCGGCGACGGGTGACGGGGGATCGGGGTCCGACTCCGGAGAGGGAGC-CTGAGAGATGGCTCCCACGTCCAAGGACGGCAGCAGGCGCGGAAATTGCCCACTCTC----CCGCGAGGAGGCAGTTACGAGGCGTG--AC--------------------------------GAAGGG---------CGCACGGCAAACGCGTGCGC-GAAGGCGATT---GGAGGGCAAG-TTCGGTGCCAGCAGCCGCGGTAATACCGACTCCAAGAGTGTCTATGGTGGATGCTGCAGTTAAAAGGTCCGTAGT--CGTGG--------------------------------------------------------------------------------------------CTGCAATCG---------------------------AACG------GTGC-CGCTCAAGC---------------GCG-------CACGTGTTGC----AA------------------------------------------TGGCTGGCGGAGCGGACGGGGGGCGCGGTATAGGCGGGCGAGAGATGAAATCGCCAAGACCCC-GCCTGGACCGGCGGAGGCGCAGGCGGCGCCCCCGGACGCGTCTGGGGATCAAGGACGAAGGCTGGA-GGATCGAAAGTGATT--AGAGACCGCTGTAGTTC-CA-GCAGTAAACGATGCCGACAGCG-CGTGGCG----------------------------------------------------------------CGCGCC-GCGCGCGGGCGAAAGC--TTAGTGTTCGGGCTCTGGGGATAGTATGCCCGCAAGTGTGAAAATTAAA--GAAATTGACGGAGCTACACCACAAGGAGTGGATTGTGCGGCTTAATTTGACTCAACCGCGG-GACACCTACCAGGCCAG-CGCGCCN----------------------------------------------------------------------------------------------------------------------------------------------------AAGGTG----------------------------------------------------------------------------------------------------------------------------------------------------------------------------------------------------------------------------------------------------------------------------------------------------------------------------------------------------------------------------------------------------------------------------------------------------------------------------------------------------------------------------------------------------------------------------------------------------------------------------------------------------------------

>>KR871373_DE_Gfos_Msp-RR2_GRA-15_1089pb_

--------------------------------------------------------------------------------------------GGGAAA-CGGCGTACAGCTCAG-TAATACTGCGATGATCTGGTCAGG-------CG-CT-------ACG-ATAACCACGGGAA-ACTGTGGCTAACAGG--------------------------------------------------------------------------------------------------------------------------------CCTGGATGAGACGCGGGTCTATCAGC--TA-GTTGGTAGTGTAATGGACTACCAAGGCGACGACGGATGACGGGAGATGGGGGTCTGATTCCGGAGAGGGAGC-CTGAGAGATAGCTCCCACGTCCATGGACGGCAGCAGGCGCGGAAATTGCCCACTCTCT--TCGCGGAGGAGGCGGTTAAGAGGCGTG--AG-----------------------------C-ACAAGGA---------CCGACGGCAAAGAAGTCGGT-GAACGCGATT---GGAGGGCAAG-TTCGGTGCCAGCAGCCGCGGTAATACCGACTCCAAGAGTGTCTGTGATTGATGCTGCAGTTAAAAAGTCCGTAGT--CGTG---------------------------------------------------------------------------------------------ATGCAGGGG---------------------------AAGG------CCGT-GGGTCGAGC---------------ACG-------GCAGGCCTGCATGCGATGTGTCCAGCAATGGAGAC-----------------------ATCATGGCGGAGCGGGTGGGGGGCGCAGTATAGGTGGGCGAGAGATGAAATGACCAAGACCCC-ATCTGGACTGACGGAGGCGAAGGCGGCGCCCCAGCACGCGTCTGAGGATCAAGGACGAAGGCCGGA-GGATCGAAAGTGATT--AGAGACCGCTGTAGTTC-CG-GCAGTAAACGATGTCGACGCCG-CGTGGCC----------------------------------------------------------TTCCG--GGCCG-CGTGGGTGGGAAACCT--CAAGTGTTCGGACTCTGGGGATAGTATGCTCGCAAGGGTGAAAATTGAA--GAAATTGACGGAGCTACACCACAAGGAGTGGATTGTGCGGCTTAATTTGACTCAACGCGGG-GAAACTTACCATCACCA-AGCGCCG-CAGA-GACTGG------CCACACGA-GCGGC--------------GCGCGAGTAATGCATGGTCGTTGGAAACCTGTGGGGTGACCT-------------------------------------------TTGGCTTAAGTGCCTG-AATGGGTGAGATCTCC----------------------------------------------------TGG-ACGGGTGCCTGAGCGGCGCAGGAGGGCGGAGGCGATAACA-GATCAGTGATGTTCTCAGATGCGGTGGGCTGCACGCGCGATACAGTGTGTGA------------CGCG---GACACAGA----------------------------TGGACTGTCACACCAGGTCGGGACTGGGCTCTGGTA--CGGGCCCACGAACGAGGAATTCCTAGTAAC--------TGCGGC-TCA----------------------------------------------------------------------------------------------------------------------------------------------------------------------------------------------------------------------------------------------------------------------------------------------------------------------------------------------------

>>LOQ-06_FR_Groe_Msp-IVF_ADR-18_356pb_LOQ-06-IVF

----------------------------------------------------------GCATGCTC--------GCGACTC-----GGCAACGGGGAA-CGGCGTACAGCTCAG-TAATACTGCGATGATCTGGTCAGG-------CG-CT-------ACG-ATAACCACGGGAA-ACTGTGGCTAACAGG--------------------------------------------------------------------------------------------------------------------------------CCTGGATGAGACGCGGGTCTATCAGC--TA-GTTGGTAGTGTAATGGACTACCAAGGCGACGACGGATGACGGGAGATGGGGGTCTGATTCCGGAGAGGGAGC-CTGAGAGATAGCTCCCACGTCCATGGACGGCAGCAGGCGCGGAAATTGCCCACTCTCT--TCGCGGAGGAGGCGGTTAAGAGGCGTG--AG-----------------------------C-ACAAGGA---------CCGACGGCAAAGAAGTCGGT-GAACGCGATT---GGAGGGCAAG-TTCGGTGCCAGCAGCCGCGG-----------------------------------------------------------------------------------------------------------------------------------------------------------------------------------------------------------------------------------------------------------------------------------------------------------------------------------------------------------------------------------------------------------------------------------------------------------------------------------------------------------------------------------------------------------------------------------------------------------------------------------------------------------------------------------------------------------------------------------------------------------------------------------------------------------------------------------------------------------------------------------------------------------------------------------------------------------------------------------------------------------------------------------------------------------------------------------------------------------------------------------------------------------------------------------------------------------------------------------------------------------------------------------------------------------------------------------------------------------------------------------------------------------------------------------------------------------------------------------------------------------------------------------------------------------------------------

>>EF016249_AR_Dichroplus_elongatus_Giglio-Tos_1894_Liebermannia_dichroplusae_SOK-07_1242pb_

--------ACCAGGTTGATTCTGCCTGACGCAGATGCTAGCTTCGGAGATTAAGCCATGCATGCGC--------GCGA-----------AGAAGGGAG-CGGCGAACGGCTCAG-TAATACTGCGATGATCTGCTCTTT-------GC-GG-------AAG-ATAACCACTGGAC-ACGGTGGCTAAGGC---------------------------------------------------------------------------------------------------------------------------------GGAGATTGAGACGCAGTCCTATCAGC--TTGGATGGTAGTGTAAAGGACTACCATGGCGACGACGGGTGACGGGAGATGAGGGTCTTGTACCGGAGAGGGAGC-CTGAGAGATGGCTCCCACGTCCATGGATGGCAGCAGGCGCGAGAATTGCCCACTCTC----AGATGAGGAGGCAGTAACGAAGCGTG--GG----------------------------------CGGC---------GCGGGTGTAAAAAGCCTG---CGGCCCGACT---GGAGGGCAAG-TTTGGTGCCAGCAGCCGCGGTAATACCGACTCCAGGAGTGTCTGTGGTGGATGCTGCAGTTAAAAGGTCCGTAGT--CGAG---------------------------------------------------------------------------------------------CGGCAGTGA---------------------------CAGG------GCGTGAGGTCAGAG---------------ACG-------CAGTTGCTGT----CG------------------------------------------AGAGTAGCGGAGCGGGCAGGGTGCGCGGAATGGTTGGGCGAGCGATGAAATG-CCAGGACCCC-AGGCGGACGAACGGAGGCGAAGGCGGCGCACTGGGACGCATCTGAGGATCAAGGACGAAGGCTGGA-GGATCGAAGGCGATT--AGAGACCGCTGTAGTTC-CA-GCAGTAAACGATGCCGACAACG-CGGCGCG-----------------------------------------------------------------AGGCG-TGGCGAGGGAGAAATC--GTAGTGTACGGGCTCTGGGGATAGTATGCTCGCAAGTGTGAAAATTAAA--GAAATTGACGGAGCTACACCACAAGGAGTGGATTGTGCGGCTTAATTTGACTCAACGCGGG-ACAGCTTACCAGGGCCA-AGCGGTG-CAGA-GAGCGG------AGACGCGA-GTGCC--------------GTGGGAGTGGTGCATGGTCGCTGGAAACCGGTGGGGCGACCC-------------------------------------------GATGCTCAAGTGCAGG-AACCGGTGAGACCCA-----------------------------------------------------TAG-ACAGGTACCG-GAGCGGTACAGGAAGGACGGGCGATAGCA-GATCAGTGATGCCCTCAGATGCCCTGGGCGGCACGCGCAATACAATTCCAG-------------TGTG---CAG---------------------------------AAAAGCGAAGTATCTGGAGGGGACTGGGGCCTGGGA--GGGCGCCACGAACGCGGAATTCCTAGTAAC--------GGCGGC-TCATGAGGCCGTGGTGAATGTGT-CCCTGTAGCTTGTACACACCGCCCGTCACTATCTCAGATGGTCGTTGCGATGAGGGACGG---------------------------------------------GAGTCCCGAATCGCTAGGACTAGATAAGATGTAA-----GTCGTAACAAGGTAACCA-------------------------------------------------------------------------------------------------------------------------------------------------------------

>>Ou-85_FR_Groe_Msp-IVG_MAR-UN_334pb_Ou-85-IVG

-------------------------------------------------------------TGTCC-------GGTGAA---------CGTAGGGGAA-CGACGAACGGCTCAG-CAGTTCTGCGATGATTTGCTCTCT-------GC-GG-------ACG-ATAAGCACGGGAA-AGTGGGGCTAAGAGC--------------------------------------------------------------------------------------------------------------------------------GGAGAATAAGACGCGGACCTATCAGG--TT-GTTGGCAGTGTAATGGACTACCAAGGC-------GGTGACGGGAGATAGGGGTCTGATTCCGGAGAGGGAGC-CTGAGAGATGGCTCCCACGTCCATGGACGGCAGCAGGCGCGGAAATTGCGCACTCCC-----------GAGGCAGTTATGAGACGTG--GC-----------------------------T--CAGGAC---------CCGACAGCAGAGGTGTTGGGGTGAGGCGACC---GGAGGGCAAG-TTTGGTGCCAGCAGCCGCGG-----------------------------------------------------------------------------------------------------------------------------------------------------------------------------------------------------------------------------------------------------------------------------------------------------------------------------------------------------------------------------------------------------------------------------------------------------------------------------------------------------------------------------------------------------------------------------------------------------------------------------------------------------------------------------------------------------------------------------------------------------------------------------------------------------------------------------------------------------------------------------------------------------------------------------------------------------------------------------------------------------------------------------------------------------------------------------------------------------------------------------------------------------------------------------------------------------------------------------------------------------------------------------------------------------------------------------------------------------------------------------------------------------------------------------------------------------------------------------------------------------------------------------------------------------------------------------

>>AF394525_ND_Daphnia_sp._Glugoides_intestinalis_REF-02_1209pb_

--------------------------------------------------------ATGCATGTTK--------AGG---------------GAATAA-NAACGGACAGCTCAG-TAAAACTGTGATGATTTAGTCTAA-----TAGA-GA-------AAG-AGAACTTCGTGAA-AATGAAGCTAGAAGG-------------------------------------------------------------------------------------------------------------------------------ATTAGAATAAGACGCAGACCTATCAGT--TA-GTTGGTAGTGTAATGGACTACCAAGACGATGACGGG-GACGGGGAATGAGGGTTTGATACCGGAGAGGGAGC-CTGAGAGATAGCTCCCACGTCCAAGGACGGCAGCAGGCGCGAAAATTGCCCACTCTT------AGTAGGAGGCAGTTAAGAGACGTG--AA--------------------------------TAAGAA---------GAGCCAGTAAAGAGGGGTA--AGTAAGAATT---GGAGGGCAAG-TTTGGTGCCAGCAGCCGCGGTAATACCGACTCCAAGAGTGTGTGAGAGAGATGCTGCAGTTAAAAAGTCCGTAGT--CGTAG--------------------------------------------------------------------------------------------AGGTAGAAA---------------------------GAAT------AAGG---ATATTGG--------------------------------------ATA------------------------------------------GGAATAGAGGAGCCGATGGGGATGACAGTATAGCAGGGCGAGAGATGAAATG-CCAAGACCCC-TGGTGGACTAAGCGAGGCGAAAGCGGTGATCTAGTAGGAGTTTGATGATCAAGGACGAAGGCCGGAGGGATCGAAAGTGATT--AGATACCGCAGTAGTTC-CS-GCAGTAAAAGATGCCGACAATT-TGACGGG---------------------------------------------------------GGGGAG--AGCCG-TTGAAAGGGAGAAATT--GTAGAGTTCGGGCTCTGGGGATAGTATGCTCGCAAGAGTGAAAATTAAA--GAAATTGACGGAGCTACACCACAAGGAGTGGATTGTGCGGCTTAATTTGACTCAACGCGAG-GAAACTTACCAAAGGCA-AGCAGTG-TGTA-GA-------------GAAGA-GCATT--------------ATGGAAGTGGTGCATGGTCGTTGGAAATTGATGGGATGACTT-------------------------------------------TGGCCTTAAATGGTTG-AATGAGTGAGATCTT-----------------------------------------------------TAG-ACAAGTCAGA-TATAGGACAGGAGGAAAGAGGCGATAACA-GATCAGAGATGCCCTNAGATGCGTTGGGCAGCACGCGCAATACAATAGCAA-G-----------CAAG---GAAATAGA---------------------------GGAGGGGAGAAGAA-TAAGATGGATTGTAGTCTGTAA--GGACGACATGAAAGAGGAATTCCTAGTAAG--------TATGTA-TCAACAATGGGTATTGAATGAGT-CCCTGTAGCTTGTACACACCGCCCGTCACTATCTCAGATGTTTTTCAGGATGAAGAGTCT---------------------------------------------AGGCTCTGAATAATGAAAAGTAGATAAGATGTAA-----GTCGTAACAAGGTTGCGGTCGGTG---------------------------AACCAGCCGCAGGATCAT----------------------------------------------------------------------------------------------------------

>>AY233131.2_ND_Lobesia_botrana_Cystosporogenes_legeri_KLE-03_1231pb_

TGAATTCCACCAGGTTGATTCTGCCTGACGTAGATGCTAGTCTCTGAGATTAAGCCATGCATGTTT--------ATG---------------TAATAA-GGACGAACAGCTCAG-TAGAACTGCGATTATTTAGTCTGT-----CTGT-GT-------GAG-ATAACTACGTGAA-AATGTAGCTAAGAGA------------------------------------------------------------------------------------------------------------------------------GGACAGAATAAGACGTAGGACTATCAGT--TA-GTTGGTAGTGTAATGGACTACCAAGACGACGACGGTTAACGGGGAATCAGGGTTTTATACCGGAGAGGGAGC-CTGAGAGATTGCTCCCACGTCCATGGACGGCAGCAGGCGCGAAAATTGCCCACTCTT------TGCAGGAGGCAGTTATGAGACGTG--AA--------------------------------GACGAG---------TGCTTGGTAAAGAGAAG----CAGGAGAATT---GGAGGGCAAG-TTTGGTGCCAGCAGCCGCGGTAATACCGACTCCAAGAGTGTGTATGAGAGATGCTGCAGTTAAAAAGTCCGTAGT--CGTGG--------------------------------------------------------------------------------------------AGGTTGAAAT--------------------------TGGG------GCTT--GGCCCCAG-----------------------------------ACAGAC------------------------------------------ATTGTGGAGGAGCCAATGGGGAACATAGTATACCAGGGCGAGAGATGAAATG-CCTAGACCCC-TGGTGGACTGAGCGAGGCGAAAGCGATGTTCTTGTGGGTATTCGGTGATCAAGGACGAAGGCTGGA-GGATCGAAAGTGATT--AGATACCGCAGTAGTTC-CA-GCAGTAAAAGATGCCGACATGT-CGGCTGG--------------------------------------------------------------G--CACAG-CTGACAGGGAGAAATC--TTAGAGTTCGGGCTCTGGGGATAGTATGCTCGCAAGAGTGAAAATTAAA--GAAATTGACGGAGCTACACCACAAGGAGTGGATTGTGCGGCTTAATTTGACTCAACGCGAG-GAAACTTACCAGGGGCA-AGTATTG-TGTA-GA-------------GAGGA-GCAAT--------------ACAGAAGTGGTGCATGGTCGTTGGAAATTGATGGGATGACTT-------------------------------------------TGAGCTTAAATGGTTG-AATGAGTGAGATCTT-----------------------------------------------------CAG-ACATGTTCCT-CACGGGACAGGAAGGAGGAGGCTATAACA-GATCAGAGATGCCCTTAGATGCCCTGGGCTGCACGCGCAATACAATAGCAC-T-----------GAGC---CGG--AGA----------------------------GAGAGGAAAGTGT-TCTGATGGATTATGTGTTGTAA--GACAGATATGAAAGAGGAATTCCTAGTAAG--------TATGTA-TCAACAATGGATATTGAATGAGT-CCCTGTAGCTTGTACACACCGTCCGTCGCTATCTCAGATGTTTTTCAGGATGAAGAGTCC---------------------------------------------AGGCTCTGAATAATGAAAAGTAGATAAGATGTAA-----GTCGTAACAAGG-------------------------------------------------------------------------------------------------------------------------------------------------------------------

>>GU130407_RU_Chironomus_plumosus_Crispospora_chironomi_TOK-12_1230pb_

-------CACCAGGTTGATTCTGCCTGACGTAGATGCTAGTCTCTAAGATTAAGCCATGCATGTTT--------ATG---------------TAATAA-GGACGAACAGCTCGG-TAGAACTACGATGATTTAGTCTGT-----CTGA-AC-------AGG-ATAACTACGTGAA-AATGTAGCTAAGAGG------------------------------------------------------------------------------------------------------------------------------GGACAGAATAAGACGTAGGACTATCAGT--TA-GTTGGCAGTGTAATGGACTACCAAGACGATGACGGGTAACGGGGAATCAGGGTTTGATACCGGAGAGGGAGC-CTGAGAGACAGCTCCCACGTCCATGGACGGCAGCAGGCGCGAAAATTGCCCACTCTT------TGTAGGAGGCAGTTATGAGACGTG--AA--------------------------------GATGAT---------TATTCAGCAAAGATGAA----TAGGAGAATT---GGAGGGCAAG-TTTGGTGCCAGCCGCCGCGGTAATACCGACCCCAAGAGTGTGTATGAGAGATGCTGCAGTTAAAAAGTCCGTAGT--CATAG--------------------------------------------------------------------------------------------AAGCGAAAAT--------------------------GGGG------ATGCGAAGTCTCAG-----------------------------------TATTTA------------------------------------------GTGATGGAAAAGCCGATGGGGAACATAGTGTACCAGGGCGAGAGATGAAATG-CCAAGACCCC-TGGTGGACTGAGCGAGGCGAAACGGATGTTCTTGTAGGCATTTGGTGATCAAGGACGAAGGCTGGA-GTATCGAAAGTGATT--AGATACCGCAGTAGTTC-CA-GCAGTAAAAGATGCCGACATGT-TCATTGG--------------------------------------------------------------A--GACAG-TGGACAGGGAGAAATC--TTAGAGTTCGGGCTCTGGGGATAGTATGCTCGCAAGAGTGAAAATTAAA--GAAATTGACGGAGCTACACCACAAGGAGTGGATTGTGCGGCTTAATTTGACTCAACGCGAG-GAAACTTACCAGGGCCA-AGTGTTG-TGCA-GA-------------AACGA-GCAAC--------------ACAGAAGTGGTGCATGGTCGTTGGAAATTGATGGGATGACTT-------------------------------------------TGAGCTTAAATGCTTG-AATGAGTGAGATCTT-----------------------------------------------------TAG-ACATGTTCCT--GCAGAACAGGAAGGAAGAGGCTATAACA-GATCAGAGATGCCCTTAGATGCCCTGGGCTGCACGCGCAATACAATAGCAC-A-----------GAAG---CGA--ATA----------------------------GACTGGAATGTGT-TTTGATGGATTATGTGCTGTAA--GGCAGATATGAAAGAGGAATTCCTAGTAAG--------TATGTA-TCAACAATGGGTATTGAATAAGC-CCCTGTAGCTTGTACACACCGCCCGTCACTATCTCAGATGTTTTTCAGGATGAAGAGTTT---------------------------------------------AGGCTCTGAATAATGAAAAGTAGATAAGATGTAA-----GTCGTAACAAGGTAACC--------------------------------------------------------------------------------------------------------------------------------------------------------------

>>DQ675604_ND_Euplotes_woodruffi_Euplotespora_binucleata_FOK-08_1146pb_

-----------------------------GTAGATGCTAGTCTCTAAGATTAAGCCATGCATGTTA--------ATGT--------------AATTAA-GGACGAATAGCTCAG-TAAAACTACGTTAATTTAATCTGT-----TTG--GA-------AGA-ATAACTACGTGAA-AATGTAGCTAAGAGA--------------------------------------------------------------------------------------------------------------------------------ACAGAATAAGACGTAGGACTATCAGT--TA-GTTGGTAGTGTAATGGACTACCAAGACTATAACGGTTAACGGGGAATCAGGGTTTGATACCGGAGAGGGAGC-CTGAGAAATTGCTCCCACGTCCAAGGACGGCAGCAGGCGCGAAAATTGCCCACTCTT------TGTAGGAGGCAGTTATGAGACGTT--TT-----------------------------------TAT---------TGTTTTGTAAAAAGAAA----CATAGTAATT---GGAGGGCAAG-TTTGGTGCCAGCAGCCGCGGTAATACCGACTCCAAGAGTGTGTATGAGAGATGCTGCAGTTAAAAAGTCCGTAGT--------------------------------------------------------------------------------------------------------------------------------------------------------------------------------------------------------------------------------------------------TTTTTAGAGGAACCAATGGGGATTATAGTATACAAAGGCGAGAGATGAAATG-TCAGGACCCT-TTGTGGACTAAGCGAGGCGAAGGCGATAATCTTGTGGGTATTCGTTGATCAAGGACGAAGGGTGGA-GGATCGAAAGTGATT--AGATACCGCTGTAGTTC-CA-CCAGTAAAATATGCCGACATGT-TTTTTGG--------------------------------------------------------------A--AACAA-GAAGCAGGGAGAAATC--TTAGAGTTCGGGCTCTGGGGATAGTATGCTCGCAAGGGTGAAAATTAAA--GAAATTGACGGAGCTACACCACAAGGAGTGGATTGTGCGGCTTAATTTGACTCAACGCGAG-AAATTTTACCAGGCCCA-AGTTTAA-TGTA-GA-------------AATGA-GTTAA--------------ACAGAAGTGGTGCATGGTCGTTGGAAATTCATGGGTTGACTT-------------------------------------------TGAGCTTAAATGCTTG-AATGAGTGAGATCTA-----------------------------------------------------AAG-ACTTGTTCCT-TTTAGGGATAGGAAGATTAGGCTATAACA-GATCAGTGATGCCCTTAGATGGCCTGGGCAGCACGCGCAATACAATATTTC-G-----------TAAA---TAA--ATA----------------------------GATTAGAACGTTA-TTCTATGGATTAACGGTTGTAA--TTCCGTTATGAAACAGGAATTCCTAGTAAA--------TGTGTA-TCATCAATGCATATTGAATAAGT-CCCTGTAGCTTGTACACACCGTCCGTCACTATCTCAGATGTTTTTCACAATGAAGAGTTT---------------------------------------------AGGCTCTGAATAATGAAAAGTAGATAAGATGT--------------------------------------------------------------------------------------------------------------------------------------------------------------------------------------

>>FN434085_IE_Gdub-ce_Msp-505_KRE-10_1087pb_

--------------------------------------------------TAAGCCATGCAATGTT-------TCTG---------------CTATAA-GGACAAACAGCTCAG-TAAAACTAAG-CGCTCTAACCT-------CGGC-AT-------CAA-CTAACTTTGGGAA-ACTAAGGCTAAAAGC--------------------------------------------------------------------------------------------------------------------------------TGAGGATGAGGCTTAGCACTATCAGC--TA-GTAGGTAGTGTAATGGACTACCTAGGCGGTGACGGTTAACGGGATACGAGAGTGTGATACCGGAGAGGGAGC-CTGAGAAACAGCTCCTACATCCAAGGATGGCAGCAGGCGCGAAAATTGTCCACTCTT------TGCAGGAGACAGTTATGAAAAGTA--AA-----------------------------A--AAGGTA---------GGTCTTGTAAAGAAAGA----TTCTAAAATT---GGAGGGCAAG-TTTGGTGCCAGCAGCCGCGGTAATACCAACTCCAAGAGTGTGTATGAGAGTTGTTGCAGTTAAAAGGTCCGTAGT--CGTTC--------------------------------------------------------------------------------------------ATGGAAATA---------------------------AGGC------TTTG---GCCTGCA--------------------------------------TTT------------------------------------------AATGTGGAGGGGCTGATGGGGACCATAGTATACAGGGGCGAGAGATGAAATG-TCTAAACCCT-CTGTGGACTCAGCGAGGCGAAAGCGGTGGTCTAGTAAGTACTCGGTGATCAAGGACGAAGGGTAGA-GGAGCGAAAGTGATT--AGATACCGCTGTAGTTT-TA-CCAGTAAAAGATGCCGACTATCCTGGCGTG------------------------------------------TGCGGGCGCAAGCAATGCGCA--GGCTG-GGAATAGAGAAATCGA--TAAGTGTTCGGGCTCTGGGGATAGTATGCTCGCAAGAGTGAAAATTAAA--GAAATTGACGGAGCTACACCACAAGGAGTGGATTGTGCGGCTTAATTTGACTCAACGCGAG-GAAACTTACCAGGCCCT-TTTGCAC-CTAA-GA-------------TACGA-GGTGC--------------GGAGGAGTGGTGCATGGTCGTTGGAAATTCATGAGATGACTT-------------------------------------------TGAGCTTAAATGCTTG-AATGAGTGAGACTCCG----------------------------------------------------TAG-ACTGGCTGCG-TTAAGCAGCAGGAGGGTGGGGCTATAACA-GATCAGTGATGCCCTTAGATGGCCTGGGCTGCACGCGCAATACAGTGGGAC-G-----------TAGG---TAT--AGA----------------------------GACGTACATGTCC-CCAGATGGATTGCCGATTGTAA--GTTCGGCATGAAAGCGGAATTCCTAGTAAG--------CACGTA-TTATCAATGGGTGCTGAATGC----------------------------------------------------------------------------------------------------------------------------------------------------------------------------------------------------------------------------------------------------------------------------------------------------------------------------------

>>KF894402_DE_Gpul_Msp-M2_GRA-14_1110pb_

-----------------------------GTAGATGCTATTTTCTGAGATTAAGCCATGCAATGTT-------TCTG---------------CTATAA-GGACAAACAGCTCAG-TAAAACTAAG-CGCTCTAACCT-------CGGC-AT-------CAA-CTAACTTTGGGAA-ACTAAGGCTAAAAGC--------------------------------------------------------------------------------------------------------------------------------TGAGGATGAGGCTTAGCACTATCAGC--TA-GTAGGTAGTGTAATGGACTACCTAGGCGGTGACGGTTAACGGGATACGAGAGTGTGATACCGGAGAGGGAGC-CTGAGAAACAGCTCCTACATCCAAGGATGGCAGCAGGCGCGAAAATTGTCCACTCTT------TGCAGGAGACAGTTATGAAAAGTA--AA-----------------------------A--AAGGTA---------GGTCTTGTAAAGAAAGA----TTCTAAAATT---GGAGGGCAAG-TTTGGTGCCAGCAGCCGCGGTAATACCAACTCCAAGAGTGTGTATGAGAGTTGTTGCAGTTAAAAGGTCCGTAGT--CGTTC--------------------------------------------------------------------------------------------ATGGAAATA---------------------------AGGC------TTTG---GCCTGCA--------------------------------------TTT------------------------------------------AATGTGGAGGGGCTGATGGGGACCATAGTATACAGGGGCGAGAGATGAAATG-TCTAAACCCT-CTGTGGACTCAGCGAGGCGAAAGCGGTGGTCTAGTAAGTACTCGGTGATCAAGGACGAAGGGTAGA-GGAGCGAAAGTGATT--AGATACCGCTGTAGTTT-TA-CCAGTAAAAGATGCCGACTATCCTGGCGTG------------------------------------------TGCGGGCGCAAGCAATGCGCA--GGCTG-GGAATAGAGAAATCGA--TAAGTGTTCGGGCTCTGGGGATAGTATGCTCGCAAGAGTGAAAATTAAA--GAAATTGACGGAGCTACACCACAAGGAGTGGATTGTGCGGCTTAATTTGACTCAACGCGAG-GAAACTTACCAGGCCCT-TTTGCAC-CTAA-GA-------------TACGA-GGTGC--------------GAAGGAGTGGTGCATGGTCGTTGGAAATTCATGAGATGACTT-------------------------------------------TGAGCTTAAATGCTTG-AATGAGTGAGACTCCG----------------------------------------------------TAG-ACTGGCTGCG-TTAAGCAGCAGGAGGGTGGGGCTATAACA-GATCAGTGATGCCCTTAGATGGCCTGGGCTGCACGCGCAATACAGTGGGAC-G-----------TAGG---TAT--AGA----------------------------GACGTACATGTCC-CCAGATGGATTGCCGATTGTAA--GTTCGGCATGAAAGCGGAATTCCTAGTAAG--------CACGTA-TTATCAATGGGTGCTGAATGCGT--------------------------------------------------------------------------------------------------------------------------------------------------------------------------------------------------------------------------------------------------------------------------------------------------------------------------------

>>KR871381_DE_Gfos_Msp-505_GRA-14_732pb_

--------------------------------------------------------------------------------------------------------------TCAGCTAAAACTAAG-CGCTCTAACCT-------CGGC-AT-------CAA-CTAACTTTGGGAA-ACTAAGGCTAAAAGC--------------------------------------------------------------------------------------------------------------------------------TGAGGATGAGGCTTAGCACTATCAGC--TA-GTAGGTAGTGTAATGGACTACCTAGGCGGTGACGGTTAACGGGATACGAGAGTGTGATACCGGAGAGGGAGC-CTGAGAAACAGCTCCTACATCCAAGGATGGCAGCAGGCGCGAAAATTGTCCACTCTT------TGCAGGAGACAGTTATGAAAAGTA--AA-----------------------------A--AAGGTA---------GGTCTTGTAAAGAAAGA----TTCTAAAATT---GGAGGGCAAG-TTTGGTGCCAGCAGCCGCGGTAATACCAACTCCAAGAGTGTGTATGAGAGTTGTTGCAGTTAAAAGGTCCGTAGT--CGTTC--------------------------------------------------------------------------------------------ATGGAAATA---------------------------AGGC------TTTG---GCCTGCA--------------------------------------TTT------------------------------------------AATGTGGAGGGGCTGATGGGGACCATAGTATACAGGGGCGAGAGATGAAATG-TCTAAACCCT-CTGTGGACTCAGCGAGGCGAAAGCGGTGGTCTAGTAAGTACTCGGTGATCAAGGACGAAGGGTAGA-GGAGCGAAAGTGATT--AGATACCGCTGTAGTTT-TA-CCAGTAAAAGATGCCGACTATCCTGGCGTG------------------------------------------TGCGGGCGCAAGCAATGCGCA--GGCTG-GGAATAGAGAAATCGA--TAAGTGTTCGGGCTCTGGGGATAGTATGCTCGCAAGAGTGAAAATTAAA--GAAATTGACGGAGCTACACCACAAGGAGTGGATTGTGCGGCTTAATTTGACT-------------------------------------------------------------------------------------------------------------------------------------------------------------------------------------------------------------------------------------------------------------------------------------------------------------------------------------------------------------------------------------------------------------------------------------------------------------------------------------------------------------------------------------------------------------------------------------------------------------------------------------------------------------------------------------------------------------------------------------------------------------------------------------------------------------------------------------------------------------

>>FN434086_IE_Gdub-ce_Msp-515_KRE-10_1107pb_

-----------------------------GTAGATGCTATTTTCTGAGATTAAGCCATGCAATGTT-------TCT----------------TAACAA-GGACAAATAGCTCAG-TAAAACTAGG-CGATCTAACCT-------CGGC-AT-------CGG-ATAACCTTGGGAA-ACTAAGGCTAAAGGC--------------------------------------------------------------------------------------------------------------------------------TGAGGGTGACGCCTAGCACTATCAGC--TA-GTAGGTAGTGTAACGGACTACCTAGGCGGTGACGGTTAACGGGATACGAGAGTGTGATACCGGAGAGGGAGC-CTGAGAAATGGCTCCCACATCCAAGGATGGCAGCAGGCGCGAAAATTGTCCACTCTT------TATAGGAGACAGTTATGAAAAGTA--AA-----------------------------A--ACGGTG---------GGTCTTGTAAAGAAAGA----TTCTAAAATT---GGAGGGCAAG-TTTGGTGCCAGCAGCCGCGGTAATACCAACTCCAAGAGTGTGTATGAGAGTTGTTGCAGTTAAAAGGTCCGTAGT--CGTTC--------------------------------------------------------------------------------------------ATGGAAATA---------------------------AGGC------TTCG---GCCTGTG--------------------------------------CTC------------------------------------------AATGTGGAGGGGCTGATGGGGACCATAGTATACAGTGGCGAGAGATGAAATG-TCTAAACCCT-CTGTGGACTCAGCGAGGCGAAAGCGGTGGTCTAGTAAGTACTCGGTGATCAAGGACGAAGGGTAGA-GGAGCGAAAGTGATT--AGATACCGCTGTAGTTT-TA-CCAGTAAAAGATGCCGACTATCCTGGCGTG------------------------------------------CGCGGGCGAAAGCGATGCGCA--GGCTG-GGAATAGAGAAATCGA--TAAGTGTTCGGGCTCTGGGGATAGTATGCTCGCAAGAGTGAAAATTAAA--GAAATTGACGGAGCTACACCACAAGGAGTGGATTGTGCGGCTTAATTTGACTCAACGCGAG-GAAACTTACCAGGCCCT-TTTGCAC-CTAA-GA-------------TACGA-GGTGC--------------GAAGGAGTGGTGCATGGTCGTTGGAAATTCATGGGATGACTT-------------------------------------------TGAGCTTAAATGCTTG-AATGAGTGAGACTCCG----------------------------------------------------TAG-ACTGGCTGCG-TTAAGCAGCAGGAGGGTGGAGCTATAACA-GATCAGTGATGCCCTTAGATGGCCTGGGCTGCACGCGCAATACAGTGGGAC-G-----------TAGG---TAT--AGA----------------------------GACGTACAGGTCC-CCAGATGGATTGCCGATTGTAA--GTTCGGCATGAAAGCGGAATTCCTAGTAAG--------CACGTA-TTATCAATGGGTGCTGAATGC----------------------------------------------------------------------------------------------------------------------------------------------------------------------------------------------------------------------------------------------------------------------------------------------------------------------------------

>>H09-01_HU_Groe_Msp-IVA_ADR-18_311pb_COU-03-IVA

------------------------------------------------------------------------------------------------AA-GGACAAATAGCTCAG-TAAAACTAGG-TGATCTAACCT-------CGGC-AG-------CAA-ATAACTTTGGGAA-ACTAAGGCTAAAGGC--------------------------------------------------------------------------------------------------------------------------------TGAGGGTGACGCCTAGCACTATCAGC--TA-GTAGGTAGTGTAACGGACTACCTAGGCGGTGACGGTTAACGGGATACGAGAGTGTGATACCGGAGAGGGAGC-CTGAGAAATGGCTCCCACATCCAAGGATGGCAGCAGGCGCGAAAATTGTCCACTCTT------TATAGGAGACAGTTATGAAAAGTA--AA-----------------------------A--ACGGTA---------GGTCTTGCAAAGAAAGA----TTCTAAAATT---GGAGGGCAAG-TTTGGTGCC----------------------------------------------------------------------------------------------------------------------------------------------------------------------------------------------------------------------------------------------------------------------------------------------------------------------------------------------------------------------------------------------------------------------------------------------------------------------------------------------------------------------------------------------------------------------------------------------------------------------------------------------------------------------------------------------------------------------------------------------------------------------------------------------------------------------------------------------------------------------------------------------------------------------------------------------------------------------------------------------------------------------------------------------------------------------------------------------------------------------------------------------------------------------------------------------------------------------------------------------------------------------------------------------------------------------------------------------------------------------------------------------------------------------------------------------------------------------------------------------------------------------------------------------------------------------------------------

>>FN610845_GB_Gdub-du_Msp-1199_KRE-10_1095pb_

------------------------------------------------------CCATGCAATGTT-------TCTGC--------------TACAAA-GGACAAATAGCTCAG-TAAAACTAGGCTCATTTGACCT-------CGCG-AT-------GCG-ATAACCTGGAGAA-ATTCAGGCTAAGGGC---------------------------------------------------------------------------------------------------------------------------------GAGGATAAGGCCTAGGACTATCAGC--TA-GTTGGTAGTGTAACGGACTACCAAGGCGACGACGGTTAACGGGACACGGGAGTGTGATACCGGAGAGGGAGC-CTGAGAGACTGCTCCCACGTCCAAGGACGGCAGCAGGCGCGAAAATTGTCCACTCTT------TGCAGGAGACAGTTATGAAAAGTA--AA-----------------------------A--AAGATA---------GGTCTTGTAAAGAGAGA----TTCTAAAATT---GGAGGGCAAG-TTTGGTGCCAGCAGCCGCGGTAATACCAACTCCAAGAGTGTGTATGAGAGTTGCTGCAGTTAAAAGGTCCGTAGT--CGTTA--------------------------------------------------------------------------------------------GTCGAAATA---------------------------GGAC------GTAG-G-GTCCAGA---------------T-----------------------TC------------------------------------------AATGTGGAGGGGCTGATGGGGGCCGCAGTATACAAGGGCGAGAGATGAAATG-CCAAGACCCC-TTGTGGACTCAGCGAGGCGAAAGCGGCGGCCCGGTAAGTGCTCGGTGATCAAGGACGAAGGGTAGA-GGATCGAAGGTGATT--AGATACCGCTGTAGTTC-TA-CCAGTAAAAGATGCCGACTATCCTGGCGTG-C---GGCGT----------------------------AGGCGGTTCGCCGCCCGTCGGCGCA--TGCCG-GGAACAGAGAAATCGA--TAAGTGTTCGGGCTCTGGGGATAGTATGCTCGCAAGAGTGAAAATTAAA--GAAATTGACGGAGCTACACCACAAGGAGTGGATTGTGCGGCTTAATTTGACTCAACGCGAG-GAAACTTACCAAGCCCT-TCCCGTC-CTAA-GA-------------CACGA-GGGCG--------------CGAGGAGTGGTGCATGGTCGTTGGAAATTCATGGGATGACCT-------------------------------------------TGAGCTTAAATGCTTG-AATGAGTGAGACCCCG----------------------------------------------------TAC-ACCGGCTGCG-CTGAGCAGCAGGGAGGTGGGGCGATAACA-GATCAGTGATGCCCTTAGATGGCTTGGGCTGCACGCGCAATACAATGGGGC-A-----------CGTC---GCG--ACA----------------------------GATGGACACGCCC-CGAGATGGATTGCCGATTGTAA--GTTCGGCATGAAAGCGGAATTCCTAGTAGA--------CACGTA-TTATCAATGGGTGTCGAATGC----------------------------------------------------------------------------------------------------------------------------------------------------------------------------------------------------------------------------------------------------------------------------------------------------------------------------------

>>GU130406_RU_Chironomus_plumosus_Helmichia_lacustris_TOK-12_1284pb_

-------CACCAGGTTGATTCTGCCTGACGTAGATGCTATTTTCTAAGATTAAGCCATGCATAGTT-------TCTGT--------------TATAAA-GGACAAATAGCTCAG-TAAAACTAAATTCATTTAATCT-------TGAT-AA-------AAA-ATAACCAAGTGAA-AATTTGGCTAAAAAC--------------------------------------------------------------------------------------------------------------------------------AAAGAATAAGATTTAGAACTATCAGC--TA-GTTGGTAGTGTAAAGGACTACCAAGGCGACGACGGCTAACGGGACACGAGAGTGTTATACCGGAGAGGGAGC-CTGAGAGATTGCTCCCACGTCCAAGGACGGCAGCAGGCGCGAAAATTGTCCACTCTT------TGTAGGAGACAGTTATGAAAAGTA--AA-----------------------------T--TTGATA---------GGTTTTTTAAAGAAAAA----TTCTAAAATT---GGAGGGCAAG-TTTGGTGCCAGCAGCCGCGGTAATACCAACTCCAAGAGTGTGTATGAGAGTTGTTGCAGTTAAAAGGTCCGTAGT--TGTTA--------------------------------------------------------------------------------------------AATTAAATA---------------------------AAAT------ATAA-C-ATTTTTA---------------A-----------------------TT------------------------------------------AATTTTGAGGGACTGATGGGGATTATAGTATACAAGGGCGAGAGATGAAATG-CCAAGACCCC-TTGTGGACTAAGTGAGGCGAAACGGATAATCTTGTAAGTACTCGATAATCAAGGACGAAGGGTGGA-GGATCGAAGGTGATT--AGATACCGCTGTAGTTC-CA-CCAGTAAAAGATGCCGACTATC-TAATTTA-A---GATATTTATTTAAATAAGTTTTTATTTATAAAGACAAAATTTAATTTATTATAATTTA--GATTA-GGAATAGAGAAATCAA--TAAGTGTTTGGGCTCTGGGGATAGTACGCTCGCAAGGGTAAAAATTAAA--GAAATTGACGGAGCTACACCACAAGGAGTGGATTGTGCGGCTTAATTTGACTCAACGCGAG-GAAACTTACCAAGCCCT-TTTTTAT-TTAA-GA-------------TATGA-GATAA--------------ATAGGAGTGGTGCATGGTCGTTGGAAATTCATGAGATGACTT-------------------------------------------TGAGCTTAAATGCTTG-AATGAGTGAGACTTTT----------------------------------------------------TAT-ACTGACTGTG-TTAAACAGTAGGAAGGTAAAGCTATAACA-GATCAGTGATGCCCTTAGATGGCTTGGGCTGCACGCGCAATACAATAAGTT-A-----------TATA---TTA-TATA----------------------------AGTAAATAAAACT-TTAGATGGATTGTTGATTGTAA--TTTCAACATGAAAGTGGAATTCCTAGTAAA--------TGTGCA-TTATCAATAGATATTGAATAAGT-CCCTGTAGCTTGTACACACCGTCCGTCAGTATCTCAGATGTTTTTTAGGATGAAGAGTTT---------------------------------------------CGATTCTGAATAATGAAGAATAGATAAGATGTAA-----GTCGTAACAAGG-TA--ACC-----------------------------------------------------------------------------------------------------------------------------------------------------------

>>FN610844_FR_Gdub-du_Msp-1154_KRE-10_1075pb_

----------------------------------------TTTCTAAGATTAAGCCATGCAGAGTT--------CTG---------------CAACAA-GAACCGATAGCTCAG-TAAAACCAGG-TTATTTGCTCT-------CGGC-GG-------ACG-ATAACCACGTGAA-AACGCGGCTAAGGAC--------------------------------------------------------------------------------------------------------------------------------AGAGAATAAGACCTGGCACCATCAGC--TA-GTTGGCAGTGTAAAGGACTACCAAGGCGGCGACGGTTGACGGGATACGAGAGTGTTATGCCGGAGAGGAAGC-CTGAGAGACTGCTTCCACGTTCATGGACGGCAGCAGGCGCGAAAATTGTCCACTCTT------TGCAGGAGACAGTTATGAGGAGTA--CG-----------------------------A--GCGATA---------GGCCCTGCAAAGACGGG----TGCTAGAATT---GGAGGGCAAG-TTTGGTGCCAGCAGCCGCGGTAATACCAACTCCAAGAGTGTGTATGAGAGTTGTTGTGGTTAAAAGGTCCGTAGT--CGTTA--------------------------------------------------------------------------------------------AGCTAAACA---------------------------AGGC------GTG----GCCTGGG--------------------------------------GCG------------------------------------------AGTTTGGAGGGGCTGGTTGGGGCCGCAGTATACAGGGGCGAGAGATGAAATG-CCAAGACCCC-CTGTGGACTCGGCGAGGCGAAGGCGGCGGCCCGGCAAGTGCTCGATGATCAAGGACGAAGGGTGGA-GGATCGAAGGTGATT--AGATACCGCTGTAGTTC-CA-CCAGTAAAAGATGCCGACTATTCCGGCCCG------------------------------------------------------------GCG--GGCCG-GGAACAGGGAAACCGA--TAAGTGTCTGGGCTCCGGGGATAGTACGCTCGCAAGGGTAAAAATTAAA--GAAATTGACGGAGCTACACCACAAGGAGTGGATTGTGCGGCTTAATTTGACTCAACGCGAG-GAAGCTTACCAGGCCCT-TGCGCGC-CGGA-GA-------------GACGA-GGCGC--------------GCAGGAGTGGTGCATGGTCGTTGGAAATTCATGGGATGACTT-------------------------------------------TGAGCTTCAATGCTTG-AATGAGTGAGACCCG-----------------------------------------------------GAG-ACGGCCTGCG--CAAGCAGGAGGACGGCGGGGCAATAACA-GATCAGTGATGCCCTTCGATGGCCTGGGCTGCACGCGCAATACAATAGGCA-G-----------CACG---GCG--ACA----------------------------AGCGGAAAGCGCC-TCAGATGGACTGCCGGCTGTAA--GCCCGGCACGAAAGCGGAATTCCTAGTAGG--------CGCGCG-GCACCAGCGCGTGCCGAATGC----------------------------------------------------------------------------------------------------------------------------------------------------------------------------------------------------------------------------------------------------------------------------------------------------------------------------------

>>HE584635_UK_Eriochier_sinensis_Hepatospora_eriocheir_STE-11_957pb_

---------------------------------------------------------------------------------------------------------------------------------------------------------------------------------------------------------------------------------------------------------------------------------------------------------------------------------------------------------------------------------------------------------------------------------------------------------GCCCAAGGGCAGCAGCAGGCGCGAAACTTGTCCACTCTT----CTGCGAGGAGACAGTCAGGAAACGTG--AA-----------------------------ATGAAAGGA---------GGGAGTGTAAACTACTACCTGTGAGGCTATT---GTTGGGCAAG-CTTGGTGCCAGCAGCCGCGGTAATTCCAACAACAAGAATGTCTATGATTGATGCTGCAGTTAAAAAGTCCGTAGT--TGTAT--------------------------------------------------------------------------------------------ATGCAATGA---------------------------AAAA------ACAC-TGATTAAGG---------------GTG-------TTAGATTTGT----TC------------------------------------------GCATTGGCGGAATGGACAGGGAGCACAGTATAGATGGGCGAAGGATGAAATC-TCAAGACCCT-ATCTGGACTGACAGAAGCGAAGGCGGTGCTCTTGTACATCTCAGAGAATCAAGGACGAAGGCTATA-GTAGTGATGGCGATT--AGAGACCGCCGTAGCTT-TA-GCAGTAAATTATGCCGACATTC-TCTATCG-G---------------------------------------------------------------AAGAT-GGGGAAGGGAGAAATC--TTAGTGTACGGGCTCTGGGGAAAATACGATCGCAAGATTGAAACTTAAAACGAAATTGACGGAAGGACACCACAAAGAGTGGATTGTGCTGCTTAATTTGACTCAACGCGGG-AAAACTTACCAAGCCAG-AGCCTAT-CGTA-GAATG-------C-AAACGA-GATAG--------------GCAAGAGTGGTGCATGGCCGTTGGAAATTGATGGGATGACCT-------------------------------------------TTCGCTTAAATGCTTT-AACCAGTGAGATCTTC----------------------------------------------------TAG-ACAGGTACCA-TGCGGTACAGGAAGGAGAAGGCTATAACA-GGTCCGTAATGCCCTCA-ATGGCTTGGGCAGCAAGCGCAATACAATATCTA-T-----------CGTA---AGA--GAA----------------------------AGCAAATGGTAGA-TAAGTAGGATTGGATCTTGTAA--GAGATCCATGAATGCGGAATTCTCAGTAAC--------AATAGGTTCATTAAGCCATTGTGAATACGT-CCCTGTTCTTTGTACACACCGCCCGTCGCCATTTCAGATGATGTTGGATATGAAGAGCCT---------------------------------------------AGGTTCTGAATATTCAA-----------------------------------------------------------------------------------------------------------------------------------------------------------------------------------------------------

>>KF549987_US_Litopenaeus_setiferus_Agmasoma_penaei_SOK-15_1271pb_

-------CACCAGGTTGATTCTGCCTGACGTAGAGGCTAGCCTCAGGGACTAAGCCATGCATTGTT-------AGTGAAGT------TTTTAATGAAA-CGACGGACGGCTCAG-TAATACTACTTTTAACTAACCTTT-------TG-TA-------CTA-ATAATTAAGGGAA-ACTGTAATTAAAAATC------------------------------------------------------------------------------------------------------------------------------ATGAGGATGTGAGGTAGACCTATTAGC--TA-GTTGGTTGTGTAAAGGACTACCAAGGCTATAATGGGTAACGGAGATTTAGTGATCGAAACCGGAGATGGAAG-CTGAGAAACGGTTCCAATGTCCAAGGATAGCAGCAGGCGCGAAAATTGCACACTCTT----TAATGGGGATGCAGTTATGAGGTATG--AC-----------------------------A--GAAAGG--------GTTATCAATAAATAAGATGACGTAAAGCTATT---AGAGGGAAAG-TTTGGTGCCAGCAGCCGCGGTAATACCAACTCTAAGAGTCTCTATGCGAGTTGCTGCAGTTAAAAAGTCCGTAGT--C-TTT--------------------------------------------------------------------------------------------ACGTAATTA---------------------------AAAA------TGAA-TGATCAAGT---------------TTC-------ATATTTTTAC----GT------------------------------------------TTATATATGAGACGGATTGGGAGCATAGTATAACTGGGTTAAGAATGAAATC-TCACTACCCT-AGTTGGACTATCAGAAGCGAAAGCGATGCTCTAATACGTACTTTTAGATAAAGGACGAAGGCTAGA-GTAGCGAAAGGGATT--AGATACCCCTGTAGTTC-TA-GCAGTAAACTATGCCGACAGAA-TGTTAGA-T--------------------------------------------------------ATATT--TCTAG-TGTTCAAGGGAAACCT--TAAGTGATCGGGCTCTGGGGAGAGTATGCTCGCAAGTGTGAAAATTAAA-CGAAATTGACGGAGTTACACCACAAGGAGTGGATTGTGCGGCTTAATTTGACTCAACGCGAG-GAATTTTACCAGGGCTG-AATATAT-TTGA-GATTGA-----TT-ACATGA-AATAT--------------ATTTGAGTGGTGCATGGTCGTTGTAAACTCATGGATTGATCT-------------------------------------------TAAGTTCAACTGCTAA-AATGGGTGAGACTTTC---------------------------------------------------ATAA-ACAGCTATCTAACAGGTAGAGGAAGGGGAAGGCGATAACA-GATCCGTGATGCCCTCAGATGTCCTGGGCTGCACGCGCAATACATTATGTA-T-----------ATTT---TTT--ATA----------------------------AATAGATACTACATATTGGGGAATTGACTTTTGTAA--ATAAGTCATGAACTTGGAATTCCTAGTAAT--------AATGAT-TCATCAAGTCATTGTGAATGTGT-CCCTGTAGCTTGTACACACCGCCCGTCACTGTCTCAGATGGTTGATGAGATGAAGAGCTT---------------------------------------------CGGTTCTGAATCTTAACAACTAAATAAGACATAA-----GTCGTAACAAGG-TA--ACCA----------------------------------------------------------------------------------------------------------------------------------------------------------

>>AF394529_ND_Daphnia_sp._Ordospora_colligata_REF-02_1349pb_

-------CACCAGGTTGATTCTGCCTGACGTGGATGCTAGTGTCTGAGATTAAGCCATGCATGTGA--------GTGAATG----TAGTAAGCAGGAA-CTGCGGACGGCTCAG-TAAGACTACGATGAAATGATCTGC-----GAAG-AA-------GAA-GTAACGGCGGGAA-ACTGAAGAAGAGAGTT--TTTGAGAATACTTGAAGGGATGCGGAGAAGTGATTTTTGAAGTGATGTTGA------------------------------------------------------------------GTATTTGAGCGGAGTGTGTTGTAGACCTATCAGC--TG-GTAGCTAGGGTAATGGCCTAGGTAGGCGACGACGGGAGACGGGGGATTAGGGTTTGATTCCGGAGAGGGAGC-CTGAGAGATGGCTACTACGTCCAAGGATGGCAGCAGGCGCGAAACTTGCCTAATCCT------GTGGGGAGGCGGTTATGAGACGTA--TG------------------------ATATTGTTATGAAG---------CGGACGATGAAAGAGTC----AGTGGATATT---GGAGGGCAAG-TCGGGTGCCAGCAGCCGCGGTAATACCTGCTCCGATGGTGTCTATGGTGAATGCTGCGGTTAAAAAGTCTGTAGT--CAAGTGAGG------------------------------------------------------------------------------------ATGTGTATGAGTAT--------------------------GAAG------GTGAGTGAACTAGA---------------GTT-----GAATAAGCTTGTA-TGTA------------------------------------------TGAGGAGAGCAACGGAGGGGGTGCACTGGATAGCTGGGCGAGAGGTGAAATG-TGAAGACCCT-GGCTGGACGAACGGAGGCGAAGGCTGTGTGCCATGACGAATGTGATGATCAAGGACGAAGGCCAGA-GGATCGAAATCGATT--AGATACCGTTTTAGTTC-TG-GCAGTAAACGATGCTGACGGGA-TGACATG------------------------------------------------------------TAT--GTGTG-TTATGCAAGAGAAATT---GAGTATGTGAGCTCTGGGGATAGTATGTTCGCAAGGGTGAAACTTAAA--GAGATTGACGGAAGGACACCACAAGGAGTGGAGTGTGCGGCTTAATTTGACTCAACGCGGG-GCAACTTACCAGGGCTG-AAGATGT-GTGGAGAACTG-----AG-AGGGGA-GCACA--------------TTGGCGGTGGTGCATGGCCGTTTGAAACGGATGGCGTGAGCT-------------------------------------------GAGGATTAATTTCCGG-AAGGCGTGAGACCCAC----------------------------------------------------AAG-ACAGGTTGTT-TGGATGACAGGAGGGAGTGGAACAGAACA-GGTCCGTTATGCCCTGAGATGTCCTGGGCGGCACGCGCACTACGATACGTG-T-----------ATGA-------------------------------------------------GATGTGTGAGGGATGAATGTGTGGAA--TGGCATTCTGAACGTGGAATTCCTAGTAAT--------GGTGGC-TCAACAAGCCGACGTGAATGTGT-CCCTGTCCTTTGTACACACCGCCCGTCGCTATCTGAGATGGCACATGGGACGAACACCGA---------------------------------------------GGGGTGTGAGTCTTCTGTGGTAGATGAGATATAA-----GTCGTAACATGG-CT--GCGGTTGGAG------------------------AACCAGCTGCAGGATCA-----------------------------------------------------------------------------------------------------------

>>FJ865223_US_Xanthocaecilius_sommermanae_Mockfordia_xanthocaeciliae_SOK-10_1443pb_

------TCACCAGGTTGATTCTGCCTGACGTGGATGCTAGTTCCTGAGATTAAGSCATGCATGTTT--------ATGAAGCGATAT-CGATTGCGGATTGATAGTACGGCTCAS-TAATACTGCGATGATTTGGTTGAG-----GGGAGTG-------TCT-GTAACTACRGGAA-ACWGTARGTAGTGGGC--TTGTAKGGTATTT--------------GCACGTGGCTTTGTGTTATGTGTG------------------------------------------------------------------GTATGTGTCTTGAGTAAGTTGCAGGCCTATCAAC--TG-GTAGGTAGGGTAATGGCCTACCTAGGTGGTGACGGGATACGGGGGATTAGGGTTTGATTCCGGAGAGGGAGC-CTGAGAGATGGCTWCTACGTCCAAGGATGGCAGCAGGCGCGAAACTTGCCTAATCCA----GTATTGGGAGGCAGTGACGGGACGTG--AT-------------------------------TGGATGG---------CGTGATGTAAAGAAGCA----TGCTTGTATC---RSAGGGCAAG-TCGGGTGCCAGCAGCCGCGGTAATACCTGCTCCGATAGTGTCTATGGTGAATGCTGCAGTTAAAAAGTCTGTAGT--CKGGACCTT-----------------------------------------------------------------------------------TTGGTATGTCTGT----------------------------TTGT------GCTT-ATGTTTGTA---------------GTG-----AGCTGTGGATGTACTTAT------------------------------------------GGTTGTACGCAGAGGATGAGGGGTACTGGATAGTTGGGCGAGAGGTGAAATG-TGATGACCCT-GACTGGACGAACAGAAGCGAAGGCTGTACTCTTGGACTTGTGTGTGGATCAAGGACGAAGGCTGGA-GGATCGAAATCGATT--AGATACCGTTTTAGTTC-CA-GCAGTAAACGATGCCGACTGTG-TGGTGCGGT---------------------------------------------------------------ATGCA-CTGTGCGCGAGAAATCAGTTAGTATGTGGGTTTTGGGGATAGTATGTTCGCAAGGATGAAACTTGAA--GAGATTGACGGAAGGACACCACCAGGAGTGGAGTGTGCGGCTTAATTTGACTCAACGCGGG-ACAACTTACCAATGCAG-TACTGAG-CGTGTGATTGA-----GT-ACAAGA-TGCTT--------------AGGGTGGTGGTGCATGGCCGTTTTAAATGGATGGCGTGAGCT-------------------------------------------GTGGATTAATTTCCGT-AAGATGTGAGACCCATGG------------------------------------------------------ACGGCGTCAA-GTGTGGCGAGGAAGGGTAGGAATAGAACA-GGTCCGTTATGCCCTAAGATGTGTTGGGCTGCACGCKCACTACSATATGCC-------------------------------------------------------------AGTGGGGCATGTGAGGGATTGAGGGATGTGA--TTGCCTCATGAACGTGGAATTCCTAGTAAG--------TRCGGK-TCATTAAGCCGTGTTGAATGAGT-CCCTGTCCTTTGTACACACCGCCCGTCGCTATCTAAGATGATACTCTGGACGAAGACTGT---------------------------------------------GAGGTCTGAGTTTGTTGNNTTAGATAANATATNA-----NNCGNANCNAGG-TN--ACCAGNGCCC------------------------GANCCTGTGCAACTAGATAAGGTATAAGTCGTAACAAGGTAACCANNATCCAATGGGGTAACTCAGCGGGACTCCAATGTTTTGTAAACCGTCATGTTTGAAGAATGAGAAAGTCGTAACAAGG

>>KC172651_CA_Odontophora_rectangula_Sporanauta_perivermis_ARD-12_1295pb_

-------------------------TGACGTGGATGCTAGTTCCTGAGGTTAAGCCATGCATGCCC--------ATGAAGCGACA--CGGTTGCGGAT-TGGCGGACGGCTCAG-TAAGACTGCGATGATTTGATCGA------TTGC-GA-------GCC-GTAACTACGGGAA-ACTGTAGGTAGGGGGG-----------------------------------GTAGGATACTGCGCGGCG------------------------------------------------------------------GTATGTGTGTCGAGTAAGTTGCAGGCCCATCAAC--TG-GTAGGTAGGGTAATGGCCTAACTAGGTTGCGACGGGAGACGGGGGATCGGGGTTTGATTCCGGAGAGGGAGC-CTGAGAGACGGCTACTACGTCCAAGGATGGCAGCAGGCGCGAAACTTGCCCAATCCTT---TGGTTGGGAGGCAGTTATGGGACGTG--AG--------------------------------GAATGG---------CGATTTGCAAAGAGAAT----TGCCGGTATC---GGAGGGCAAG-TCGGGTGCCAGCAGCCGCGGTAATACCTGCTCCGATAGTGTCTATGGTGAATGCTGCAGTTAAAAAGTCCGTAGT--CGAGATGTG------------------------------------------------------------------------------------CAGTATGTCTGGG---------------------------GGGC------CTGT---GTTTGTT---------------GCG-----GGCTGTGGACGTA-TGTG------------------------------------------GGTTTGACGCAGCGGACGGGGCGCACTGGATAGTTGGGCGAGAGGTGAAATG-TGAGGACCCT-GACTGGACGGACGGAGGCGAAGGCGGTGCGCTTGGACGAGTGTGTGGATCAAGGACGAAGGCTGGA-GGATCGAAATCGATT--AGAGACCGTTTTAGTTC-CA-GCAGTAAACGATGCCGACTGGA-CGGCGCG-G---------------------------------------------------------------GAGCG-CTGTGGGAGAGAAATCGGTTAGTGTGTGGGCTCTGGGGATAGTATGTTCGCAAGGATGAAACTTAAA--GAGATTGACGGAAGGACACCACAAGGAGTGGAGTGTGCGGCTTAATTTGACTCAACGCGGG-GCAACTTACCAGTGCCA-AGCGCCT-CTGG-GATTGA-----GT-ACATGA-GGGGC--------------GCGGTGGTGGTGCATGGCCGTTTCAAATGGATGGCGTGAGCT-------------------------------------------GTAGATTAAGTTCTGC-AAGACGTGAGACCCTG----------------------------------------------------TGG-ACGGTGCCC--CTGGGGCAAGGAGGGGCAGGAAAACAACA-GGTCCGTTATGCCCTAAGATGCACTGGGCTGCACGCGCACTACGATATCTG-C-----------CTGC----------------------------------------------GGCACGATGTGGGGGATCAACGGTTGTAA--ATCTGTTGTGAACGTGGAATTCCTAGTAAG--------GGCCGT-TCATCAGGCGGGCTTGNANGTGT-CCCTGTCCTTTGTACACACCGCCCGTCGCTATCTAAGATGATATGTTGGACGAAGATTGG---------------------------------------------GAGATCTGAGTCCTGTGTATTAGATAAGATATAA-----GTCGTAACATGG-CT--GCTGTTGGAG------------------------AACCATTAGCAGGATCATAA--------------------------------------------------------------------------------------------------------

>>GQ206147_UK_Tubifex_tubifex_Neoflabelliforma_aurantiae_MOR-10_1375pb_

-------------------------TGACGTGGACACTTGTCTCGAAGAATAAGCCATGCATGTTC--------GTGAAGC------TTTTAGTGAAA-CTGCGAATGGCTCAG-TAATATGGTYCTAATTTGATCAA------CAAGCAT-------TGG-ATAACCACGGGAA-ACTGTGGCTAATACAT---------------------------GTAGAAGATGTTGTATGGGAAGAAGA------------------------------------------------------------------CATCAGATGTTGAGTAAGGGACCAGCCTATCAGT--TT-GATTGTAGTGTAAAGGACTACAATGACGATGACGGGT-ACGGGGAATCAGGGTTCGATTCCGGAGAGGGAGC-CTGAGAAATGGCTCCCAGTTCTAAGGAACCCAGCAGGCGCGAACCATTTTCAATCTA------CAAAGGAAAGTGTGAAGAGACGTG--AT--------------------------------TCAAGA---------AGTACTTTAAAAGGGTA----CTGATAAACT---AGAAGGCAAG-CGAGGTGCCAGCAGCTGCGGTAATTCCCCCTCTAGGAGTGTCTGTGAGTATTGCTGTGGTTAAAACGTCCGTAGT--TGATATAAAGAAGTAGTTTGATCTACTTT--------------------------------------------------------------GTATGGTCATGGATA---------------------------AAAG------AATG-TGCTTAAAG---------------CGA----TCTAATCCATGATA-CTAG------------------------------------------ATGTGATAAGAGCAGATGAGGAACACAGGATTCTGCAGCGAGAGGTGAAATT-TGATGACCTG-CGGGGGACTAACAGATGCGAAGGCGGTGTTCTAGGATGTTTCTGTTAATCAAGGACTTAAGCCAGA-GGATCGAAGGCGATT--AGAGACCGTCGTAGTTC-TGAGCTGTAAACGATGCCAACGGTA-CGATGTG------------------------------------------------------------AGGATTTGCA-TTGTATAAGAGAAATT---TAGTGTTTGGGCTCCAGGGATAGTACGCCGGCAACGGTGAAATTTGAA--GACATTGACGGAAGGACACCACAAGGAGTGGAGTGTGCGGCTTAATTTGACTCAACGCGGG-ACAACTTACCAGGGCCG-GATGTAC-GTGT-GAAAGA-----AGGGGTTGA-GATACCTGTCTTGATTGTACAGAGAGTGGTGCATGGCCGTTCTCAACACGTGGGGTGACTTG------------------------------------------TCAGGTTAATTCCGGT-AACGTGTGAGACCCTTAA--------------------------------------------------AAG-ACTGGGAACG-TAAGTTTCAGGAAGGAAAGGGCAATAACA-GGTCTGTGATGCCCTCAGATGTTCTGGGCTGCACGCGCACTACAATAGAAG-A-----------TGTA-AATTTCAGTGTAA-----------------------AATAAAAATCTTTTGAATGTTGGGATTGAGTTTTGTAA--ATGACTCATGAACATGGAATTCCTAGTAAT--------TGCTATTTCATTAAGAAGCGATGAATGAGT-CCCTGTCCTTTGTACACACCGCCCGTCGTTATCTAAAATGGATGTGCAGGTGAAGTGAGT---------------------------------------GTGATAGCTAGCTGAACCTGTATAACTAGATCGGATACAA-----GTCGTAACAAGG-TA--TCTGTAGGTG------------------------AACCTGCAGATGGATCAGTGAAAA----------------------------------------------------------------------------------------------------

>>AF069063_ND_Anopheles_stephensi_Anncaliia_algerae_MUL-00_1389pb_

-------CACCAGGTTGATTCTGCCTGATATGTGTGCTAGCGTCAAAGATTTAGCCATGCATGCTT-------TTCGAACC------CTCGTGGGGAG-AGGCGGATAGCTCAG-TAATACAGTTACAACATAAGCTG------CGTG-TG-------TGG-ATAACCTTGTTAA-GATAAGGCTAAGACTT--------------------------AGCGTGTCGCACTTTTGTGAAGAAAGG------------------------------------------------------------------CGACTTGAGCAGCATTGGTTTCTGACCTATCAGT--TA-GTATGTTCTGTAAGGGAGAACATAGACTATGACGGGTAACGGGGGATGCACGTCTGATACCGGAGAGGAAGC-CTTAGAGACAGCTTTCACGTCCAAGGATGGCAGCAGGCGCGAAACTTACCCAATTGTTTTTTGTGACAGAGGTAGTTATGACGTATT--CT----------------------------TTAGAGAGAG---------ACCTTGTTAGACATGGTC---CATAGCGACT---GGAGGGCAAG-TCTGGTGCCAGCAGCCGCGGTAATTCCAGCTCCAGTAGTGCATATAC---ATGCTGTAGTTAGAAAGTTTGTAGC--CTATTTATG------------------------------------------------------------------------------------GATTGTTTTAGAC---------------------------AAAA------GGAC-GACTCAAAT-------------TGACC----TTTCATTTGACTAA-TGCA------------------------------------------TGAATGTAGAAGCGATTGAAGGCGATTGTATTCACCAGCCAGAGGTAAAATT-TGATGACCTG-GTGAGGACACACGGAGGCGAAAGCGATTGCCTAGAGCGTATTCAGTGGTCAAGAACGTAAGCCGGA-GGATCAAAGATGATT--AGATACCGTTGTAGTTC-CG-GCCGTAAATTATGCCAACTTGT-GCTTCTG------------------------------------------------------------CTTCTGCGGA-GGCGCATAGAGAAATCA-AGAGTTTATGGGCTCTAGGGATAGTAATCCGGCAACGGACAAACTTAAA--GAAATTGGCGGAAGGACACCACAAGGAGTGGATTATGCGGCTTAATTTGACTCAACGCGGG-ACAACTCACCAGAGCCT-ATGTGCA-GGAG-ACAGTG--------AGCTTT-GAGAGCGGACTGGATAGTACTTTGAGTGGTGCATGGCCGTTTGCAACACGTGAGGTGACTTG------------------------------------------TCAGGTTTACTCCGGT-AACGTGTGATGTGCTGTATG------CAAGTATT-----------------------------TTTGTGAG-ACTGCAGGCGGTAAGCCTGATGAAGC--GGCGCTATAACA-GGTCAGTGATGCCCTTGGATGTTCTGGGCTGCACGCGTAATACAGTGGGTG-C-----------TGTA-GATATATATA--------------------------GGTGGAAAAGGGCCCGAGACTGGGATCATGCTTTGTAA--GAAGGATGTGAACGTGGAATTCCTAGTAAT--------CGCTGC-TCACTAAGTAGCGATGAATGAGT-CCCTGTTCTTTGCACACACCGCCCGTCGCTATCTGAGATGGATGTTTTTATGAAGATGCT------------------------------------GCTGTTAGAGGCATTTGAGTAAGGACGACTAGATTAGATATAA-----GTCGTAACAAGG-CA--GCGGTAGAAG------------------------AATCTGCCGCTAGATCATA---------------------------------------------------------------------------------------------------------

>>AF033197_ND_Lophius_sp._Spraguea_lophii_HIN-UN_1364pb_

-------CACCAGGTTGATTCTGCCTGACGTGGATGCTAGTCTCATAGATTTAGCCATGCATGTGTA------AGCGAAGC------GATTAGTGAAG-CGGCGGAAGGCTCAG-TAACGGGCGAGTATTTTAATCTC------TAGA-AC-------CGG-ACAACCTCAGGAA-ACAGAGGGGAAAACGG--AGTAAGAGCATGT---------------GTTGTTAGCATGTGATGAGAACA------------------------------------------------------------------ACAGTGCAGGAGAGTAAGAAGCCATCCCATCAGT--TA-GTAAGTAGGGTAAGGGCCTATTTAGACGTAGACGGGT-ACGGGGGATTAGGGTTTGATTCCGGAGAGGGAGC-CTGAGAGACGGCTACCAGGTCCAAGGACAGCAGCAGGCGCGAAAATTACCGAAGCCTC---CAAGAGGGCGGTAGTGAAGAGACGTG--AA--------------------------------AACAA----------GTGTGTGTAAAAACCAC----ATGAGTAACTGTGGAGGGTCAAG-GCTGGTGCCAGCATCCGCGGTAATACCAGCTCCAGGAGTGTCTATGATGATTGCTGCGATTAAAAGGTCCGTAGT--CGAAGAACA------------------------------------------------------------------------------------GTACTTGTTTGTA---------------------------ATGT------GGC--TTATCGAGA--------------GCCG----AATAGAACGAGAAG-AGTT------------------------------------------CAGAGTAAGGAGYGAAGGAGGGCTAGATTATTGAGCAGCGAGAGGTGAAATT-TGATGACCTGCTTTAGGAGTAACAGAGGCGAAAGCGCTAGTCAAGAACGAATCCGATGATCAAGGACGTAGGCTGGA-GGATCGAAGACGATTAGAGAGACCGTAGTAGTTC-CA-GCAGTAAACGATGCCTACGCTG-TTGTGGT------------------------------------------------------------TAT--GAGCA-CGGCAGAAGAGAAATC---TAGTA---GGGCTTTGGGGAGAGTACGCGCGCAAGCGATAAATTTAAAG-GAAATTGACGGAAGAACACCACAAGGAGTGGAGTGTGCGGCTTAATTTGACTCAACGCGGG-ACAGCTTACCATACCCG-ATGGCCG-TGTG-AGCGTA-----GT-ACGCGA-TAGGT--------------CAGAGAGTGGTGCATGGCCGTTAACGACGAGTGGGGTGACCT-------------------------------------------TTGGGTTAAGTCCGCCGAAGTAGTGAGATCCCTAGGA------TACGAAA-------------------------------GTTATCG-ACAGGTGT--TAAAAACACAGGAAGGAAGGGGCAAGAACAGGGTCAGTGATGCCCTTAGATGGTATGGGCTGCACGCGCACTACAGTGGTCA-C-----------AGCA---GAAACGGAT--------------------------AGAAGTAAATGTGATCGAGAGGGAATGAGCACTGTAA--TGTGCACAGGAACGAGGAATTGCTAGTAAT--------CGTTATCTCATTAAGAAGCGATGAATGTGT-CCCTGTTCTTTGTACACACCGCCCGTCGTTATCGAAGATGGAACCAGGTGCGAACAAGTG-------------------------------------------AAAGCGAGTGAGTGCAGGGTTCYAGATCTGATACAA-----GTCGTAACAAGG-CA--GCTGTAGGAG------------------------AACCATTAGCAGGA--------------------------------------------------------------------------------------------------------------

>>AJ438957_GB_Echinogammarus_berilloni_Dber_TER-04_912pb_AJ438957

-----------------------------------------------------------CATGTGTA------AGCGAAGC------GTAACGTGGAG-CGGTGAAAGGCTCAG-TAACGGGCGAGTTATTTGTTCTC------CTGG-GA-------CGG-ACAACACCGGGAA-ACTGGTGGGAAAACGT--CTAAGTTGCG---------------GTTTTTTAATCGTGGCGTAAACCATT-------------------------------------------------------------------TGGTGCAGGAGAGTAAGCTGCCATCCTATCAGT--TA-GTAAGTAGGGTAAGGGCCTACTTAGACGAAGACGGGT-ACGGGGAATGAGGGTTTGATTCCGGAGAGGGAGC-CTGAGAGACGGCTACCAGGACCAAGGTCAGCAGCAGGCGCGAAAATTATCGAAGCCCG--C-ATAGGGGCGATAGTGAGGAGACGTG--TA-----------------------------T--TACGAA---------GTGTGTGTAAAGAACGC----ACTAATAACT---GGAGGTCAAG-TCTGGTGCCAGCATCCGCGGTAATTCCAGCTCCAGGGGTGTCTATGATGATTGCTGCGATTAAAAAGTCCGTAGT--CAAGCTGAC------------------------------------------------------------------------------------TGACTTGCCTGCA---------------------------ATGT------GAC--TGATTAAGA--------------GACG----AGCAGGGCTAGGAA-AGCA------------------------------------------GAGAATTAGGAGCGACCGAGGGCTAGAGTATTGAATGGCGAGAGGTGAAATT-TGATGACCCA-TTCAGGAGTGACAAAGGCGAAGGCACTAGTCAAGGGCGAATCCGATGATCAAGGACGTAGGCTAGA-GTTTCGAAAACGATT--AGAGACCGGAGTAGTTC-TA-GCAGTAAACTATGCCGACGCCG-TGGTATG-G---TATT--------------------------------------------------------CTGTA-TTGCGGAAGAGAAATC---AAGTA---AGGCTTTGGGGAGAGTACGCGCGCAAGCGATAAATTTAAAG-GAAATTGACGGAGGAACACCACAAGGAGTGGAGTGTGCGGCTTAATTTGACTCAACGCGGG-ACAGCTTACCAGGCCCG-ATAATCA-TACG-AGCGTA-----GT-ACGCGA-TAGGT--------------TAAAGAGTGGTGCATGGCTGCTATCGACAGT---------------------------------------------------------------------------------------------------------------------------------------------------------------------------------------------------------------------------------------------------------------------------------------------------------------------------------------------------------------------------------------------------------------------------------------------------------------------------------------------------------------------------------------------------------------------------------------------------------------------------------------------------------------------------------------------------------------------------------------------

>>AJ438956_FR_Groe_Dmue_TER-04_919pb_ALP40-04

-----------------------------------------------------------CATGTGTA------AGCGAAGC------TATATGTGGAG-CGGTGAAAGGCTCAG-TAACGGGCGATTTATTTAATCTC------CTGG-GG-------CGG-ACAACATCGGGAA-ACTGATGGGAAAACGT--CTAAGTTGCA---------------TTAATTTTAGTGTGACGTAAACGATT------------------------------------------------------------------ATCGTGCAGGAGAGTAAGATGCCATCCTATCAGT--TA-GTAAGTAGGGTAAGGGCCTACTTAGACGAAGACGGGT-ACGGGGAATGAGGGTTTGATTCCGGAGAGGGAGC-CTGAGAGATGGCTACCAGGACCAAGGTCAGCAGCAGGCGCGAAAATTATCGAAGCCCG--C-CTAGGGGCGATAGTGAGGAGACGTG--TA-----------------------------T--AACGAA---------GTACGTGTAAAGAACGT----ACTAATAACT---GGAGGTCAAG-TCTGGTGCCAGCATCCGCGGTAATTCCAGCTCCAGGGGTGTCTATGATGATTGCTGCGATTAAAAAGTCCGTAGT--CAAGCTGCC------------------------------------------------------------------------------------TGACTGACCTGCA---------------------------ATGT------GAT--TGATTAAGG--------------AACG----AGCAGGGTTAGGAA-AGCA------------------------------------------GAGAATTAGGAGCGACCGAGGGCTAGAGTATTGAATGGCGAGAGGTGAAATT-TGATGACCCA-TTCAGGAGTGACAAAGGCGAAGGCACTAGTCAAGGGCGAATCCGATGATCAAGGACGTAGGCTAGA-GTTTCGAAAACGATT--AGAGACCGGAGTAGTTC-TA-GCAGTAAACTATGCCGACGCCG-TGGTATG-G---TTTTTT------------------------------------------------GTGG--CTGTA-TTGCGGAAGAGAAATC---AAGTA---AGGCTTTGGGGAGAGTACGCGCGCAAGCGATAAATTTAAAG-GAAATTGACGGAGGAACACCACAAGGAGTGGAGTGTGCGGCTTAATTTGACTCAACGCGGG-ACAGCTTACCAGGCCCG-ATAATCG-AGCG-AGCGTT-----GT-ACGCGA-TAGAT--------------TAAAGAGTGGTGCATGGCTGCTATCGACAGT---------------------------------------------------------------------------------------------------------------------------------------------------------------------------------------------------------------------------------------------------------------------------------------------------------------------------------------------------------------------------------------------------------------------------------------------------------------------------------------------------------------------------------------------------------------------------------------------------------------------------------------------------------------------------------------------------------------------------------------------

>>AJ438960_GB_Orchestia_cavimana_Dcav_TER-04_1326pb_Out_Group

-----------------------------------------------GATTTAGCCATGCATGTGTA------AGCGAAGC-------TATTGTGGAG-CGGTGAAAGGCTCAG-TAACGGGCGATTTATTTAGTCTT------CTGG-GA-------CGG-ACAACACCGGGAA-ACTGGTGGGAAAACGT--CTAAGCTGCGAATC------A---CGCTATGTGGTTGTGGCAGAAACTGT--------------------------------------------------------------------TAGTGCAAAAAAGTAAGATGCCATCCTATCAGT--TA-GTAAGTAGGGTAAGGGCCTACTTAGACGAAGACGGGT-ACGGGGAATGAGGGTTTGATTCCGGAGAGGGAGC-CTGAGAGACGGCTACCAGGACCAAGGTCAGCAGCAGGCGCGAAAATTATCGAAGCCCG--C-CTAGGGGCGATAGTGAGGAGACGTG--AA-----------------------------T--T-TTAG---------GTGCGTGTAAAGAACGC----ACTAGCAACT---GGAGGTCAAG-TCTGGTGCCAGCATCCGCGGTAATTCCAGCTCCAGGGGTGTCTATGATGATTGCTGCGATTAAAAAGTCCGTAGT--CAAGCCGCC------------------------------------------------------------------------------------AGACCAGTCTGGA---------------------------ATGT------TTCT-TGATCAAGA--------------GACG----AGCAGGGCTGGGAA-AGCG------------------------------------------GAGAATTAGGAGCGACCGAGGGCTAGAGTATTGGGTGGCGAGAGGTGAAATT-TGATGACCCA-TCCAGGAGTGACAAAGGCGAAGGCACTAGTCAAGGGCGAATCCGATGATCAAGGACGTAGGCTAGA-GTTTCGAAAACGATT--AGAGACCGGAGTAGTTC-TA-GCAGTAAACTATGCCGACGCCG-TGATATG-A---TTTT-----------------------------------------------------G--TTGTA-TTGCGGAAGAGAAATC---AAGTA---AGGCTTTGGGGAGAGTACGCGCGCAAGCGATAAATTTAAAG-GAAATTGACGGAGGAACACCACAAGGAGTGGAGTGTGCGGCTTAATTTGACTCAACGCGGG-ACAGCTTACCAGGCCCG-ATAATCG-TACG-AGCGTA-----GT-ACGCGA-TAGAT--------------TAGAGAGTGGTGCATGGCTGCTATCGACAGTTGGGGTGACCT-------------------------------------------TAGGGTTAATTCCGGC-AAGTAGTGAGACCCCCGCAG------TAT---------------------------------------TGG-ACAGGCGTCGTGAAGATGCAGGAAGGAGGGGACAAGAGCA-GGTCAGTGATGCCCTTAGATGGCCTGGGCTGCACGCGCACTACAGTGGTCA-T-----------TATA-AGGAG-AAGTT--------------------------AGAAATAAAGATGATCGAGAGGGACTGGGCTTTGTAA--GAGGCCCACGAACGAGGAATTGCTAGTAAT--------CGCAGGCTCATTAGGATGCGATGAATATGT-CCCTGTACCTTGTACACACCGCCCGTCGTTATCGAAGATGGAATTGTATGCGAACGAGCA------------------------------------------GCAAGCGAGTGAGCGTATAGTTCTAGATGTGATAAAA-----GTCGTAACAAGG-CA--ACTGTAGGAG------------------------AACCTGTAGTTGGATCACAC--------------------------------------------------------------------------------------------------------

>>AF044387_ND_Myoxocephalus_scorpius_Pleistophora_typicalis_NIL-98_1369pb_

-------CACCAGGTTGATTC----TGCCGTGGATGCTAGTTTCATAGGTTAAGCCATGCATGTGTA------AGCGACTG------TTTCAGTGGAGCCGGCGCAAGGCTCAG-TAACGGGCGTCTGATTTGATCTC------TCGG-AG-------TGG-ACAACCTCTGTAA-CCGGAGGCCAAAACAC--GGGAGGGGCAT--------------GCAGTGGAGATGCCCCGAGAAC---A------------------------------------------------------------------TTAGTGAGAGAGAGTAAGGAGCCATCCCATCAGT--TA-GTAAGTAGGGTAAGGGCCTACTTAGACGAGGACGGGT-ACGGGGAATTAGAGTTTGATTCCGGAGAGGGAGC-CTGAGAGACGGCTACCAGGTCCAAGGACAGCAGCAGGCGCGAAAATTACCGAAGCCTG--CATTCAGGGCGGTAGTAAGGAAACGTG--AA--------------------------------AACGAT---------GTGCAGGTAAAGAATGC----ACTAGTAACA---GGAGGTCAAG-ACTGGTGCCAGCATCCGCGGTAATACCAGCTCCTGGAGTGTCTATGATGATTGCTGCAGTTAAAGAGTTCGTAGT--CGGACTGCG------------------------------------------------------------------------------------TGACTGGCGTGAA---------------------------AGACCCTTTTATT--CATTCAAGG--------------GGGG----CGCA-CGCCGGAAA-AGCA------------------------------------------GGGAATAAGGAGCGGCCGGGGCACAGGTTATTAAGCGACGAGAGGTGAAATT-TGATGACTCG-CTTAGGAGCAACAGAGGCGAAACGGCTGTGCAGGGGCGATTCCGATGATAAAGGACGTAGGCTAGA-GGATCGAAGATGATT--AGAGACCGTTGTAGTTC-TA-GCAGTAAACGATGCCGATGCCG-TGGGGCA-G---TTCTT--------------------------------------------------AGA--CTGCG-CCGCGGAAGAGAAATT---GAGTA---GGGCCCTGGGGAGAGTACACGCGCAAG----AAATTTAAAG-GAAATTGACGGAAGAACACCACAAGGAGTGGAGTGTGCGGCTTAATTTGACTCAACGCGGG-ACAGCTTACCAGGCCCG-ACGACCG-CACG-AGTGTG-----GT-ACACGA-TAGGT--------------CGGAGAGTGGTGCATGGCCGTTAACGACAAGTGGGGTGACCT-------------------------------------------TTGGGTTAAGTCCGGG-AAGTTGTGAGACCCCTGCTG------GCG-----------------------------------AGCCAGGCACAGGTGC--TCAAAGCACAGGAAGGAAGGGTCAAGAACA-GGTCAGTGATGCCCTTAGATGGTCTGGGCTGCACGCGCACTACAGTGGTCA-C-----------ACGA-GTTTA-TAGAT--------------------------AGAAGAAATGGTGATCGAGAGGAAATGAGCTTTGCAA--GAGGCTCAGGAACGAGGAATTGCTAGTAAT--------CGCGGGCTCATTAAGACGCGATGAATACGT-CCCTGTTCTTTGTACACACCGCCCGTCGTTATCGAAGATGGAGTCAGGCGCGAACAAGCG------------------------------------------A-GAGCGAGTGAGTGCAGGGTTCTAGATGTGATACAA-----GTCGTAACAAGG-TA--GCTGTAGGAG------------------------AACCTTTAGCTGGATCAGACCGATA---------------------------------------------------------------------------------------------------

>>AJ438985_IE_Gdue.cel_Pmul_MAC-03_1328pb_

-----------------------------------------------GGGTTAGCCATGCATGTGTA------AGCGAAGC------TTTTAGTGGAG-CGGCGCAAGGCTCAG-TAACGGGCGTCTGATTTGATCTC------TCGG-AG-------TGG-ACAACCTCTGTAA-CCGGAGGCCAAAACAC--GGGAGGGGCAT--------------GCAGTGGAGATGCCCCGAGAAC---A------------------------------------------------------------------TTAGTGAGAGAGAGTAAGGAGCCATCCCATCAGT--TA-GTAAGTAGGGTAAGGGCCTACTTAGACGAGGACGGGT-ACGGGGAATTAGAGTTTGATTCCGGAGAGGGAGC-CTGAGAGACGGCTACCAGGTCCAAGGACAGCAGCAGGCGCGAAAATTACCGAAGCCTG--CATTCAGGGCGGTAGTAAGGAAACGTG--AA--------------------------------AACGAT---------GTGCAGGTAAAGAATGC----ACTAGTAACA---GGAGGTCAAG-ACTGGTGCCAGCATCCGCGGTAATACCAGCTCCTGGAGTGTCTATGATGATTGCTGCAGTTAAAGAGTTCGTAGT--CGGACTGCG------------------------------------------------------------------------------------TGACTGGCGTGAA---------------------------AGACCCTTTTATT--CATTCAAGG--------------GGGG----CGCAGCGCCGGAAA-AGCA------------------------------------------GGGAATAAGGAGCGGCCGGGGCACAGGTTATTAAGCGACGAGAGGTGAAATT-TGATGACTCG-CTTAGGAGCAACAGAGGCGAAAGCGCTGTGCAGGGGCGATTCCGATGATAAAGGACGTAGGCTAGA-GGATCGAAGATGATT--AGAGACCGTTGTAGTTC-TA-GCAGTAAACGATGCCGATGCCG-TGGGGCA-G---TTCTT--------------------------------------------------AGA--CTGCG-CCGCGGAAGAGAAATT---GAGTA---GGGCCCTGGGGAGAGTACACGCGCAAGCGAGAAATTTAAAG-GAAATTGACGGAAGAACACCACAAGGAGTGGAGTGTGCGGCTTAATTTGACTCAACGCGGG-ACAGCTTACCAGGCCCG-ACGACCG-CACG-AGTGTG-----GT-ACACGA-TAGGT--------------CGGAGAGTGGTGCATGGCCGTTAACGACAAGTGGGGTGACCT-------------------------------------------TTGGGTTAAGTCCGGG-AAGTTGTGAGACCCCTGCTG------GCA-----------------------------------TGCCAGG-ACAGGTGC--TCAAAGCACAGGAAGGAAGGGTCAAGAACA-GGTCAGTGATGCCCTTAGATGGTCTGGGCTGCACGCGCACTACAGTGGTCA-C-----------ACGA-GTTTA-TAGAT--------------------------AGAAGAAATGGTGATCGAGAGGAAATGAGCTTTGCAA--GAGGCTCAGGAACGAGGAATTGCTAGTAAT--------CGCGGGCTCATTAAGACGCGATGAATACGT-CCCTGTTCTTTGTACACACCGCCCGTCGTTATCGAAGATGGAGTCAGGCGCGAACAAGCG------------------------------------------A-GAGCGAGTGAGTGCAGGGTTCTAGATGTGATACAA-----GTCGTAACAAGG-TA--GCTGTAGGAG------------------------AACCTTTAGCTGGATCA-----------------------------------------------------------------------------------------------------------

>>AJ252961_US_Aedes_albopictus_Vavraia_culicis_CHE-00_1312pb_Outgroup

-----------------------------------------------------------CATGTGCA------AGCGAAGC------CGGTGGTGGAG-CGGCGCAAGGCTCAG-TAACGGGCGACTGATTTGATCTC------CCGG-TG-------TGG-ACAACCTCTATAA-CCGGAGGCCAAAACAC--AGGAGGCGCGT--------------GACACGGGCACGGGCGTGCAACC-GG------------------------------------------------------------------CAGGTGCGGGAGAGTAAGGAGCCATCCCATCAGT--TA-GTAAGTAGGGTAAGGGCCTACTTAGACGAAGACGGGT-ACGGGGAATTAGAGTTCGATTCCGGAGAGGGAGC-CTGAGAGACGGCTACCAGGTCCAAGGACAGCAGCAGGCGCGGAAATTACCGAAGCCCG--CGTTCGGGGCGGTAGTAAGGAGACGTG--AA--------------------------------TACGAT---------GTGCAGGTAAAATATGC----ACTGGTAACA---GGAGGTCAAG-ACTGGTGCCAGCATCCGCGGTAATACCAGCTCCTGGAGTGTCTATGACGATTGTTGCAGTTAAAGAGTTCGTAGT--CGGGCTGCA------------------------------------------------------------------------------------TGACCGGCGCTGA---------------------------AGGCTCCCT--------ATCGAGG--------------GGGG----CAGGGCGCCGGAAA-AGCA------------------------------------------GAGGATGAGGAGCGGCCGGGGGCCAGGTTATTAAGCGACGAGAGGTGAAATT-TGATGACTCG-CTTAGGAGCAGCAGAGGCGAAAGCGCTGGCCAGGGGCGAATCCGATGATAAAGGACGTAGGCTAGA-GGATCGAAGACGATT--AGAGACCGTTGTAGTTC-TA-GCAGTAAACGATGCCGATGCCG-TGGGGCG-G---TGC-----------------------------------------------------AG--CCGCC-ACGCGGAGGAGAAATT---GAGTA---GGGCCCTGGGGAGAGTACACGCGCAAGCGGGAAATTTAAAG-GAAATTGACGGAAGAACACCACAAGGAGTGGAGTGTGCGGCTTAATTTGACTCAACGC-GG-ACAGCTTACCAGGCCCG-ACGGTCG-CACG-AGTGTG-----GT-ACACGA-TAGGC--------------CGGAGAGTGGTGCATGGCCGTTAACGACAAGTGGGGTGACCT-------------------------------------------TTGGGTTAAGTCCGGG-AAGTAGTGAGACCCCTGCCG------GCG-----------------------------------CGCCGGG-ACAGGTGC--TCAAAGCACAGGAAGGAAGGGTCAAGAACA-GGTCAGTGATGCCCTTAGATGGTCTGGGCTGCACGCGCACTACAGTGGTCG-C-----------AGAA-ATCAG-TGCCC-------------------------GAGCGGCAATGGCGATCGAGAGGGAACGAGCTCTGGAA--GGGGCTCGGGAACGAGGAATTGCTAGTAAT--------CGTGGGCTCATTAAGACACGGTGAATACGT-CCCTGTTCTTTGTACACACCGCCCGTCGTTATCGAAGATGGAGTCAGGCGCGAACAAGCG------------------------------------------A-GAGCGAGTGAGTGCAGGGTTCTAGATGTGATACAA-----GTCGTAACAAGG-TA--GCTGTAGGAG------------------------AACCAGTAGCAGGATCATAA--------------------------------------------------------------------------------------------------------

>>TAP-1R_HU_Groe_Msp-IIIA_ADR-18_388pb_TAP-1R-IIIA

--------------------------------------------------------------------------GCGAAGC------CGGTGGTGGAG-CGGCGCAAGGCTCAG-TAACGGGCGACTGATTTGATCTC------CCGG-TG-------TGG-ACAACCTCTGTAA-CCGGAGGCCAAAACAC--AGGAGGCGCGT--------------GACACGGGCACGGGCGTGCAACC-GG------------------------------------------------------------------CAGGTGCGGGAGAGTAAGGAGCCATCCCATCAGT--TA-GTAAGTAGGGTAAGGGCCTACTTAGACGAAGACGGGT-ACGGGGAATTAGAGTTCGATTCCGGAGAGGGAGC-CTGAGAGACGGCTACCAGGTCCAAGGACAGCAGCAGGCGCGGAAATTACCGAAGCCCG--CGTCCGGGGCGGTAGTAAGGAGACGTG--AA--------------------------------TACGAT---------GTGCAGGTAAAATATGC----ACTGGTAACA---GGAGGTCAAG-ACTGGTGCCAGCAGCCGCGGA----------------------------------------------------------------------------------------------------------------------------------------------------------------------------------------------------------------------------------------------------------------------------------------------------------------------------------------------------------------------------------------------------------------------------------------------------------------------------------------------------------------------------------------------------------------------------------------------------------------------------------------------------------------------------------------------------------------------------------------------------------------------------------------------------------------------------------------------------------------------------------------------------------------------------------------------------------------------------------------------------------------------------------------------------------------------------------------------------------------------------------------------------------------------------------------------------------------------------------------------------------------------------------------------------------------------------------------------------------------------------------------------------------------------------------------------------------------------------------------------------------------------------------------------------------------------------

>>HM626203_CA_Oncorhynchus_sp._Loma_salmonae_BRO-10_1354pb_

-------------------------TGACGTGGATGCTAGTCTCATAGGTTAAGCCATGCATGTGGA------AGCGAAGC---CTTTTATGGTGGAG-CGGCGTACGGCTCAG-TAACGGGCGATCGATTTGATCTG------CCTA-TA-------CGG-ACATCCTCTGTAA-ACGGAGGGTAAAACGT--AAGGGGGGCTTCC--------------TTGGGGAAGTTCGCCAAACCTGTA------------------------------------------------------------------AAGGTGAGGCAGAGTAAGAAGCCATCCCATCAGT--TA-TTAAGTATGGTAAGGGCCTACTTAGACGAAGACGGGT-ACGGGGAATTATCGTTTGATTCCGGAGAGGGAGC-CTGAGAGACGGCTACCGGGTCCAAGGACAACAGCAGGCGCGAAAATTACCGCAGCCTG--CGATCAGGGCGGTAGTAAGGAGACGTG--AT--------------------------------GACGAA---------GTGCTGATAAAAGCTGC----ACTAGTGACA---GGAGGAAAAG-ACTGGTGCCAGCACCCGCGGTAATACCAGCTCCTGGAGTGTCTATGATGATTGCTGCAGTTAAAGAGTTCGTAGC--CGAGGCATT----------------------------------------------------------------------------------ATTTAACTGCGTTAA---------------------------AGGG------GAT--ATCTCAAAT--------------ATCC----CTGTGCGCAGTGAT-TTTG------------------------------------------CAGAATAAGGAGTGCTTAGGGACCAGAGTATCATACGGCGACTGGTGAAATG-GGATGACCCG-TGTGGGAGTAACAGAGGCGAAAGCGCTGGCCAGGGGCGAGTCCGATGGTAAAGGACGTAGGCTAGA-GGATCGAAGACGATT--AGAGACCGTTGTAGTTC-TA-GCAGTAAACGATGCCGATGTCG-TGGTGCC-G---AATAA-------------------------------------------------------TGGCG-ACGCGAAAGAGAAATC---GAGTA---GGGCCCTGGGGAGAGTACACGCGCAAGCGAGAAATTTAAAG-GAAATTGACGGAAGAACACCACAAGGAGTGGAGTGTGCGGCTTAATTTGACTCAACGCGGG-ACAACTTACCGGGCCCG-ACGACCG-TAAG-AGTGTG-----AC-ACACGA-TAGGT--------------CGAAGAGTGGTGCATGGCCGTTAACGACGAGTGGAGTGATCT-------------------------------------------TTGGGTTAAGTCCGTA-AATTAGTGAGACCTCAGCCG------TAA---------------------------------------GGG-ACAGGTGC--GCAAAGCACAGGAAGGATGGGTCAAGGACA-GGTCAGTGATGCCCTTAGATGGTCCGGGCTGCACGCGCACTACAGTGGTCG-C-----------CGAA-ATTAC-CTGAT--------------------------AATTATAAAGGCGATCGAGAGGGAATGAGCTTTGTAA--GAGGCTCAGGAACGAGGAATTGCTAGTAAT--------CGCGGACTCATTAAGACGCGATGAATACGT-CCCTGTTCTTTGTACACACCGCCCGTCGTTATCGAAGATGAAGATAGGCGCGAACGATCT------------------------------------------ACCAGAAAGTGAGCGCAGGTTTTTAGATCTGATACAA-----GTCGTAACAAGG-TA--GCTGTAGGAG------------------------AACCTGTAGCTGGATCAGACCGATT---------------------------------------------------------------------------------------------------

>>KU163282_ND_Pandalus_montagui_Paradoxium_irvingi_STE-15_937pb_

---------------------------------------------------------------------------------------------------------------------------------------------------------------------------------------------------------------------------------------------------------------------------------------------------------------------------------------------------------------------------------------------------------------------------------------------------------GGTCCAGGACGGCAGCAGGCGCGAAAATTACCGAAGCTCA--A-AAAGAGGCGGTAGTAAAGAGACGTGT-AA-----------------------------A--AACGAA---------ACAAGGGTAAAAAACTT----GTTAAAAACT---GGAGGTCAAG-TCTGGTGCCAGCATCCGCGGTAATACCAGCTCCAGGGGTGTCTATGATGATTGCTGCGATTAAAAGGTCCGTAGT--CAAAATTAC------------------------------------------------------------------------------------AAACTAGTCTGAA---------------------------AAAT------GTT--AGATAAAAA--------------AGCG----AATAGGACAAGTACGTAAG------------------------------------------AAAAATTAGGAACGACCGGGGGCTAGTTAATCGAGCAACGAGAGGTGAAATT-TGATGACTTG-CTTGGGAGAAACAGAGGCGAAAGCGCTAGTCAAGGGCGAATCCGATGATCAAGGACGTAGGCTGGA-GTATCGAACACGATT--AGATACCGTAGTAGTTC-CA-GCAGTAAACAATGCCGACACCG-TATAATG-C---------------------------------------------------------------AAGTT-GAGCGGAAGAGAAATT---AAGTA---GGGCTTTGGGGAGAGTACACGCGCAAGCGATAAATTTAAAG-GAAATTGACGGAAGAACACCACAAGGAGTGGAGTGTGCGGCTTAATTTGACTCAACGCGGG-ACAGCTTACCAGACCCG-AGGATTA-AAGG-AGCG-A-----AT-ACGCGA-TAAAT--------------CTGAAAGTGGTGCATGGCCGTTATCGACGAGTGAAGTGATTT-------------------------------------------CAAGGTTAAATCCGAC-AAGTCGTGAGACCCTTAAAA------TAATAAATAATTAGTTTTAGT----------------TATTATAT-ACAGGTA---TGAAAATACAGGAAGGAAAGGACAAGAACA-GGTCAGTGATGCCCTTAGATGGTCTGGGCTGCACGCGCACTACAGTGGTTA-TTTAAAAAGAAGAATA-ATTTA-TAAAAA-------AAGTTAT----------TAGAGAGAAATATAATCGAGAGGGAATGAGCGCTGCAA--GGCGCACAGGAACGAGGAATTGCTAGTAAT--------CGCAAGCTCAGTAAGATGCGAAGAA-------------------------------------------------------------------------------------------------------------------------------------------------------------------------------------------------------------------------------------------------------------------------------------------------------------------------------------

>>GQ246188_FR_Dvil_Cdik_OVC-10_1292pb_GQ258752

----------------------------------------------------------GCATGTGTA------AGCGAACA------A---GAGGAAG-CTGCGGACTGCTCAG-TAACAGACATATAATTTAATCTT------TACA-GAAACGAGCGGA-ATAAACTCAGGAA-ACAGAGTGCAATACGT--AAAAGACGAATTTT------TTATTATAAGAAATACGTTTTTAGCTTGAAC------------------------------------------------------------------AAAGCGGTAAAGAATAAGTTGTCAGCCTATCAGT--TA-GTAAGTAGGGTAAGGGCCTATTTAGACGAAGACGGGT-ACGGGGAATTAGAGTTTGATTCCGGAGAGGGAGC-CTGAGAAATAGCTACCAGGTCCAAGGACGGCAGCAGGCGCGAAAATTACCGAAGCTCG--A-ATAGAGGCGGTAGTAATGAGACGTAT-TA-----------------------------A--TATAAA---------ACAAGGGTAAAAAACTT----GTTAGTAACT---GGAGGTCAAG-TCTGGTGCCAGCATCCGCGGTAATACCAGCTCCAGGGGTGTCTATGATGATTGCTGCGATTAAAAGGTCCGTAGT--CGAATTTAT------------------------------------------------------------------------------------ATAATTGTTTGTA---------------------------ATAT------GCT--AGATAAAAT--------------AACA----GAAAGAACAATTACTTTAA------------------------------------------ATGAA--AGGAATAGTAAGGGGCTGATTAATTGAGCAACGAGAGGTGAAATT-TGATGACTTG-CTTAGGAGAAACAGAGGCGAAAGCGTCAGTCAAGTATAAATCCTATGATCAAGGACGTAGGCTAGA-GTATCGAACACGATT--AGATACCGTAGTAGTTC-TA-GCAGTAAACTATGCCTACACTA-TCGAATA-A---------------------------------------------------------------AAGTT-TGGTAGAAGAGAAATCT-TAAGTA---GGGCTTTGGGGAGAGTACACGCGCAAGCGATAAATTTAAAG-GAAATTGACGGAAGAACACCACAAGGAGTGGAGTGTGCGGCTTAATTTGACTCAACGCGGG-ACAGCTTACCATACCCG-AGGACTA-TAAG-AGTG-A-----AT-ACACGA-TAAGT--------------CTAAAAGTGGTGCATGGCCGTTATCGACGAGTGAAGTGATTT-------------------------------------------TATGGTTAAATCCGAC-AAGTTGTGAGACCCTTATTT------AAATACA---------------------------------------GGTATTG---TTAAAATACAGGAAGGAAAGGACAAGAACA-GGTCAGTGATGCCCTTAGATGGTATGGGCTGCACGCGCACTACAATGGTTA-T-----------AATA-ATAAA-GATAA------------TT-----------AAAGTATAAATATAATCAAGAGGAATTGAGAACTGAAA--AGTTCCCATGAACGAGGAATTGCTAGTAAT--------CGTAGGCTCAGTAAGATACGATGAATATGT-CCCTGTTCTTTGTACACACCGCCCGTCGTTATCGAAGATGGAGTTTTACCCGAACAAGCT------------------------------------------T-AAGCGAGTGAGTGTATGATTCTAGATCTGATACAA-----GTCGTAACAAGG-CA--G-------------------------------------------------------------------------------------------------------------------------------------------------------------

>>KR190602_GB_Dhae_Corn_BOJ-15_1186pb_KR871369

---------------------------------------GTCTCATAGATTTAGCCATGCATGTGTA------AGCGAACA------A--TTAGGGAG-CTGCGGACTGCTCAG-TAACAGGCGATTAATTTAATCTT------TACA-AA-------TGG-ACAAACTCAGGAA-ACGGAGTGTAATACAT--AAAAAATGATTTTT------TAAATAAAAAGAAAACATTTTTAGCTTGAAA------------------------------------------------------------------AAAGCGGTAAAGAATAAGACGCCAACCCATCAGT--TA-GTAAGTAGGGTAAGGGCCTATTTAGACGAATACGGGT-ACGGGGAATTAGGGTTTGATTCCGGAGAGGGAGC-CTGAGAGACGGCTACCAGGTCCAAGGACGGCAGCAGGCGCG-AAATTACCGAAGCTCA--A-ATAGAGGCGGTAGTAATGAGACGTATAAA-----------------------------A--AATAAT---------ATAAGGGTAAAAAACTT----ATTAATAACT---AGAGGTCAAG-TCTGGTGCCAGCATCCGCGGTAATACCAGCTCTAGGGGTGTCTATGATGATTGCTGCGATTAAAAGGTCCGTAGT--CGAATTTAA------------------------------------------------------------------------------------ATAATTGTTTGTA---------------------------ATAT------GTT--AGATAAAAT--------------AATA----AAAAGAACAATTAC-TTAA------------------------------------------ATGAA--AGGAATAACAAGAGGTTGATTAATTGAGTAACGAGAGGTGAAATT-TGATGACTTA-CTTAGGAGAAACAGAGGCGAAAGCGTCAATCAAGTGTAAATCCGATGATCAAGGACGTAGGCTGGA-GTATCGAACACGATT--AGATACCGTAGTAGTTC-CA-GCAGTAAACTATGCCTACGCCA-ATGAATG-A---------------------------------------------------------------AAGTT-TGATGGACGAGAAATCT-AGAGTA---GGGCTTTGGGGAGAGTACACGCGCAAGCGATAAATTTAAAG-GAAATTGACGGAAGAACACCACAAGGAGTGGAGTGTGCGGCTTAATTTGACTCAACGCGGG-ACAGCTTACCAAACCCG-AAAACTA-AAAG-AGTG-A-----AT-ACACGA-TAAGT--------------TTAAGAGTGGTGCATGGCCGTTATCGACGAGTGAAGTGATTT-------------------------------------------TATGGTTAAATCCGAC-AAGTCGTGAGACCCT--ATA------AAATAAA---------------------------------------ACAGGTA---TGAAAATACAGGAAGGATAGGACAAGAACA-GGTCAGTGATGCCCTTAGATGGTTTGGGCTGCACGCGCACTACAGTGGTTA-T-----------AATA-AAATA-TATAGA-------AAACTA-----------AATAAAGAAATATAATCAAGAGGGATTGAGTATTGAAA--AATACCCATGAACGAGGAATTGCTAGTAAT--------CGTAGGCTCAGTAAGATACG------------------------------------------------------------------------------------------------------------------------------------------------------------------------------------------------------------------------------------------------------------------------------------------------------------------------------------------

>>HM800849.2_CA_Lepeophtheirus_salmonis_Facilispora_margolisi_JON-12_1403pb_

------------------------------------------------GTTGAGTGATTCTTTTTT--------TTTA---------GTTGAGATGAG-CGACGAACGGCTCAG-TAACG-GGCGATGATCTGTTCAG------CGGA-AG-------CGG-TCGACCTCGGGAA-ACGGAGGGGAGAACGT--GGAGCTTGCGC-----------------------GTTTGGTAGATTAGCGT------------------------------------------------------------------GTGAGTGTGTTGAGTGAGTTGCCATCCCATCAGC--TA-GTAAGTAGGGTAAGGGCCTACTTAGGCGTAGACGGGT-ACGGGGAATAGGGGTTTGATTCCGGAGAGGGAGC-CTGAGAGATGGCTACCATGTCCAAGGACGGCAGCAGGCGCGAAAATTACCGAAGGCT----TGAGAGTGCGGTAGTGATGAGACGTA--TG-------------------------------AGTTGAG---------AGGAGGGTAAAGATCTC----CGATGTAACT---GGAGGGAAAG-TCTGGTGCCAGCAGCCGCGGTAATACCAGCTCCAGGAGTGTCTATGAATATTGCTGCGATTAAAAAGTCCGTAGT--CATGTATGTTGTTTTTTAATTC---------------------------------------------------------------------CTCGCTGGGCGTGTA---------------------------AAGC------ACT--AGCTTAAAG--------------GGTG----TGAGTGTCCAAACCGGGGA------------------------------------------TGACATTGGGATAGTGTGGGGGGTAATGAATCGGGGCACGAGAGGTGAAATT-TGATGACTGT-TCCGGGTCGTCCGGAGGCGAAGGCGTTACCCAAGCACGAATCTGCCGATCAAGGACGTAGGCTGGA-GGATCGAACACGATT--AGATACCGTAGTAGTTC-CA-GCAGTAAACGATGTAGACGAGC-CGGTTGTGGTGCGGATTGCTTT-------------------TCCTGCGAGTTGGGGGGAGTGTGATTCTGCATTGCG-GTGGTGAATAGAAATAG-AGAGTA---GTACTTCGGGGAGAGTACACGCGCAAGCGATAAATTTAAAG-GAAATTGACGGAAGAACACCACAAGGAGTGGAGTGTGCGGCTTAATTTGACTCAACGCGGG-ACAGCTTACCAGTGCAG-AAGGTCG-TAAG-AGCGTG------A-ACGCGA-TAGAC--------------TGGAGAGTGGTGCATGGCCGTTATAGATGGGTGTATTGATAT-------------------------------------------TGCTATTAAGTTACGT-AATCCATAAAACGTGCGTTTTCTGTCTAGTAGATTTATTTTTGGGTTGGTTAGG---------CGGGAGAG-ACGGTTACCC-CTCGGTAAAGGAAGGAGCACGCAAGAACA-GGTCAGTGATGCCCTCAGACGTACTGGGCTGCACGCGCACTACATTTGTTG-G-----------TGTAATATTATTGTATTATA---------------------TAGAGAGAAAGCCAATATGGAGGAACTGTGTACTGAAA--GGTACTCACGAACGAGGAATTGCTAGTAAT--------CGCGAGTTCAGGAGAGCGCGAAGAATGTGT-CCCTGTTCTTTGTACACACCGCCCGTCGTTATCGAAGATGGGGCTGCGTATGAACGAGTT------------------------------------------TTTAGTGAGTGAATGCGCGGTTCTAGATCTGATACAA-----GTCGTAACAAGG-CA--GCTGTAGGAG------------------------AACCTGCAGCTGGATCA-----------------------------------------------------------------------------------------------------------

>>NAU97150_ND_Apis_mellifera_Nosema_apis_GAT-98_1243pb_

------ACACCAGGTTGATTCTGCCTGACGTAGACGCTATTCCCTAAGATTGACCCATGCATGTCT--------TTGACGTA----CTATGTACTGAA-AGATGGACTGCTCAG-TAATACTCACTTTATTTGATGTA------CA-T-TA-------TAC-ATAACTACGTTAA-AGTGTAGCTAACATAT-------------------------------------------------------------------------------------------------------------------------------GTACAGTAAGAGTGAGACCTATCAGC--TA-GTTGTTAAGGTAATGGCTTAACAAGGCAATAACGGGTAACGGTATTACTTTGTAATATTCCGGAGAAGGAGC-CTGAGAGACGGCTACTAAGTCTAAGGATTGCAGCAGGGGCGAAACTTGACCTATGGAT---ATTATCTGAGGCAGTTATGGGAAGTA--AC-AT----------------------------AGTTGTT--------TCACATTTTAAACGTATGTGAGCAGATTAATT---GGAGGGCAAA-TCGAGTGCCAGCAGCCGCGGTAATACTTGTTCCAAGAGTGTGTATGATGATTGATGCAGTTAAAAAGTCCGTAGT--TTA---------------------------------------------------------------------------------------------------------------------------------------------------------------------------------------------------------------------------------------------TTGTTAAGAAGCAATATGAGGTGTACTGTATAGTTGGGAGAGAGATGAAATG-TGACGACCCT-GACTGGACGAACTGAAGCGAAAGCTGTACACTTGTATGTATTTTTTGAACAAGGACGTAAGCTGGA-GGATCGAAGATGATT--AGATACCATTGTAGTTC-CA-GCAGTAAACTATGCCGACGATG-TGATATG-A---G------------------------------------------------------ATG--TTGTA-TTACATTATAGAAATT--AGAGTTTTTTGGCTCTGGGGATAGTATGATCGCAAGATTGAAAATTAAA--GAAATTGACGGAAGAATACCACAAGGAGTGGATTGTGCGGCTTAATTTGACTCAACGCGAG-GTAACTTACCAATATTTTATTGTTC-TGCG-AGGATA-----TG-ATCTGA-GGATG--------------ATAATAGTGGTGCATGGCCGTTTTCAATGGATGCTGTGAAGT-------------------------------------------TTTGATTAATTTCAAC-AAGACGTGAGACCCTTTATT-------------------------------------------------AG-ACTGACACTA--TTAGTGTAGGAAGGAAAGGACTAAAACA-GGTCAGTTATGCCCTCTGACATTTTGGGCAGCACGCGCAATACAATAGAC--------------TTTA--------------------------------------------------ATCTTTATGGGATAATATTTTGTAA--GAGATATTTGAACTTGGAATTGCTAGTAAA--------TTTTAT-TAAATAAGTAGAATTGAATGTGT-CCCTGTTCTTTGTACACACCGCCCGTCGCTATCTAAGATGATATGTGTTGTGAAATTAGTG-----------------------------------------CAAGCTACTTGAACAATATGTATTAGATCTGATATAA-----GTCGTAACATGG-TT--GCTGTTGGAG------------------------AACCATTAGCAGGATCATAA--------------------------------------------------------------------------------------------------------

>>XR_002966746_ND_Apis_mellifera_Nosema_ceranae_PEL-15_1259pb_

-------CACCAGGUUGAUUCUGCCUGACGUAGACGCUAUUCCCUAAGAUUAACCCAUGCAUGUUU--------UUGACAU------------UUGAA-AAAUGGACUGCUCAG-UAAUACUCACUUUAUUUUAUGUA------AAUU-UU-------UAA-UUAACUACGUUAA-AGUGUAGAUAAGAUGU-------------------------------------------------------------------------------------------------------------------------------UUACAGUAAGAGUGAGACCUAUCAGC--UA-GUUGUUAAGGUAAUGGCUUAACAAGGCUGUGACGGGUAACGGUAUUACUUUGUAAUAUUCCGGAGAAGGAGC-CUGAGAGACGGCUACUAAGUCUAAGGAUUGCAGCAGGGGCGAAACUUGACCUAUGGAU---UUUAUCUGAGGCAGUUAUGGGAAGUA--AU-AU--------------------------UAUAUUGUU--------UCAUAUUUUAAAAGUAUAUGAGGUGAUUAAUU---GGAGGGCAAA-UCAAGUGCCAGCAGCCGCGGUAAUACUUGUUCCAAGAGUGUGUAUGAUGAUUGAUGCAGUUAAAAAGUCCGUAGU--UUAUU-------------------------------------------------------------------------------------------------------------------------------------------------------------------------------------------------------------------------------------------UUUUUAAGAAGCAAUAUGAGGUGUACUGUAUAGUUGGGAGAAAGAUGAAAUG-UGACGACCCU-GACUGGACGAACAGAAGCGAAAGCUGUACACUUGUAUGUAUUUUUUGAACAAGGACGUAAGCUGGA-GGAGCGAAGAUGAUU--AGAUACCAUUGUAGUUC-CA-GCAGUAAACUAUGCCGACGAUG-UGAUAUG-A---AAAU------------------------------------------------AUUAAU--UUGUA-UUACAUAAUAGAAAUU--UGAGUUUUUUGGCUCUGGGGAUAGUAUGAUCGCAAGAUUGAAAAUUAAA--GAAAUUGACGGAAGAAUACCACAAGGAGUGGAUUGUGCGGCUUAAUUUGACUCAACGCGAG-GUAACUUACCAAUAUUUUAUUAUUU-UGAG-AGAACGGUUUUUU-GUUUGA-GAAUG--------------AUAAUAGUGGUGCAUGGCCGUUUUCAAUGGAUGCUGUGAAGU-------------------------------------------UUUGAUUAAUUUCAAC-AAGACGUGAGACCCUUAUUU------UUU-----------------------------------AUUAAAG-ACAGACACAA--UCAGUGUAGGAAGGAAAGGAUUAAAACA-GGUCCGUUAUGCCCUCUGACAUUUUGGGCUGCACGCGCAAUACAAUAGAUA-------------UAUA--------------------------------------------------AUCUUUAUGGGAUAAUAUUUUGUAA--GAGAUAUUUGAACUUGGAAUUGCUAGUAAA--------UUUUAU-UAAAUAAGUAGAAUUGAAUGUGU-CCCUGUUCUUUGUACACACCGCCCGUCGCUAUCUAAGAUGAUAUAUGUUGUGAAAUUAGUG-----------------------------------------AAAACUACUUAAACAAUAUGUAUUAGAUCUGAUAUAA-----GUCGUAACAUGG-UU--GCUGUUGGAG------------------------AACCAUUAGCAGGAUCAUAA--------------------------------------------------------------------------------------------------------

>>AJ011833_ND_Gdub-ce_Ngra_TER-99_787pb_AJ011833

---------------------------------------------------------------------------------------------------------------------ACTCTTATTTTATTTGATGTA------TT-A-GG-------ATT-ATAACTATGTTAA-ATTATAGATAACAATA-------------------------------------------------------------------------------------------------------------------------------ATACAATAAGAATAAGATCTATCAGT--TA-GTTGTTAAGGTAATGGCTTAACAAGACTATGACGGATAACGGTATTACTTTGTAATATTCCGGAGAAGGAGC-CTGAGAGATTGCTACTAAGTCTAAGGATTGCAGCAGGGGCGAAACTTGACCTATGATA---TGATATTGAGGCAGTTATGAGTAGTA--TT-TT--------------------------TAATTATTG--------TAATATTGTAAGTATATACTACAAGATAAATC---GGAGGGCAAA-TCGAGTGCCAGCAGCCGCGGTAATACTTGTTCCGATAGTGTGTATGATGATTGATGCAGTTAAAAAGTCTGTAGT--TTA---------------------------------------------------------------------------------------------------------------------------------------------------------------------------------------------------------------------------------------------TGATTAATAAGCATTGTGAGGTATATTGTATGGTTAGGAGAGAGATGAAATG-TGATAACCCT-AACTGGATGAACAGAAGCGAAAGCTATATACTTAAATGTATTATTAGAACAAGGACGTAAGCTAGA-GGATCGAAGATGATT--AGATACCATTGTAGTTC-TA-GCAGTAAACTATGTTAAATCAT-AGATACA-T---T----------------------------------------------------TTAAT--ATGTATTTATGTAGAGAAATTA--AGATTATATTGACTCTGGGGATAGTATGATCGCAAGATTGAAAATTAAA--GAAATTGACGGAAGAATACCACAAGGAGTGGATTGTGCGGCTTAATTTGACTCAACGCGGG-GTAATTTACCAGG-------TATCA-CATG-ATATAA-----TA-TTTT-----ATC--------------ATGATAGTGGTGCATGGCCGTTTCCAATGGATGCTGTGAAGT-----------------------------------------------------------------------------------------------------------------------------------------------------------------------------------------------------------------------------------------------------------------------------------------------------------------------------------------------------------------------------------------------------------------------------------------------------------------------------------------------------------------------AAT--------------------------------------------------------------------------------------------------------------------------------------------------------------------------------------------------------

>>AY958070_ND_Cancer_magister_Nadelspora_canceri_VOS-05_1278pb_

-----TCCACCAGGTTGATTCTGCCTGACGGGGAAGCTAGTTTCACAGATTCAGCCATGCAAGTAG-----------------------TATGTATGT-ATACACAAGGCTCAG-TATCG-AGTATAGCTTTGCTCTC------CAAG-AT-------GTG-ATACTTTCAGGAA-ACAGAAAATAAAGCAT----------------------------------CTATCTTCTAGAGTATTTT------------------------------------------------------------------AGAGGAGAGGAGAAGAAGCGACTCACCTATCAGT--TA-GTAGGTATGGTAAGGGCATACCTAGACGAAGACGGGT-ACGGGGAAGGCAACTTCGATTCCGGAGAGGGCGCATTTAGAGATGGCGACCAGTTCTAAGGAGTGCAGCAGGCTCGAAACTTACCGAATTATA-----GATTAGAGGTAGTGATGAAACGTT--TA-----------------------------T-ATAGAAA---------TACTTGGTAAAGCAAGT----ATTAACAACT---GGAGGGTAAG-TCTGGTGCCAGCAGCCGCGGTAATACCAGCTCCAGGAGCTTCTTCGAT--ATGTTGCGGTTAAAACGTCCGTAGT--CGCGGCTTG------------------------------------------------------------------------------------GGACTGACCTGTA---------------------------ATCT------ATT--TGGTCAACA--------------GATA-----GATAGGGGCAGTA-GCAA------------------------------------------GCTGGAAAAGAGCAATTTGGTGTCAGCTAATGGTATGGGGAGGGGTGAAGTC-TGAGGATCCA-TGCAGGAGGAGCAAAGGCGAAAGCACTGACAAAGATTGATTCTGTTGATCAAGGACAGAGGCTAGA-GGATCGAATACGATT--AGATACCGTAGTAGTTC-TA-GCAGTGACCGATGATGATTTTG--------------------------------------------------------------------------CTTA-TGGCAATAGAGAAATC--AAAATA---GATCTCCGGGGGGAGTACATGCGCAAGCAAGAAACTTAAA--GAAATTGACGGAAGACTACCACAAGGTGTGGATTGTTCGGCTTAATTTGACTCAACGCGGG-AAAACTTACCAAGCTTA-TTTGTTC-AACG-AGTAT------TT-ATATTA--GAGC--------------AAAATGGTGGTGCATGGCCGTTCCTAACAGATGGAGTGATTT-------------------------------------------TGTGATTAACTTCCAT-AATCTGTGTAATCTCAGA------------------------------------------------------ATAGCTTGTT----CGAAAGAACAATTCGAGGCAAGAACA-GGTCAGTGATGTCCTTTGATAGCTTGGGCCGCACGCGCAATACAATGTTTT-A-----------TGTA-GTAAGATATA----------------------------GATAAAAAATAGAACATGAGGGATTGAGGGCTGAAA--AGCACTCATGAACACGGAATAGCTAGTAAT--------CGTCAGTTCAATATACGGCGATGAATATGT-CCCTGTTCTTTGTACACACCGCCCGTCGTTATCGAAGATGGAGTGATTTTTGAGTCAATT-------------------------------------------ATAATTGGCGAATGAGTCATTCTAAATCCGGTACAA-----GTCGTAACAAGG-TA--ACCAA---------------------------------------------------------------------------------------------------------------------------------------------------------

>>AF492593_ND_Astacus_fluviatilis_Thelohania_contejeani_LOM-01_1361pb_

-------CACCAGGTTGATTCTGCCTGACGTGGAAGCTATTCTTTAAGATTAAGCCATGCATGTGTAGAATGAAGTGAAGC-----CGTTAGGTGGAA-CAGCGAAAAGCTCAG-TAATACAATCATTATTTGGTCTAC-----AAGATAT-------AGA-ATAACCTTGATAA-ATTAAGGCTAAAGCTA-----------------------------------------------------------------------------------------------------------------------------TTGTAGAATAAGAGATTGACCTATCAGC--TA-GTATGTAGGGTAAGGGCCTACGTAGGCGATGACGGGTAACGGGGAATTAGGGTTCTATTCCGGAGAAGGAGG-CTGAGAGATGGCTACTAGGTCTAAGGAGAGCAGCAGGCGCGAAACTTACCCCAATGCTATTTAGTAGTGAGGTAGTTATAAGAAGTA--GT-----------------------------GAATGTATTTTACTGATTTTCCTTGTAAATATAGGA---AATGATAATT---GGAGGGCAAG-TCTGGTGCCAGCAGCCGCGGTAATTCCAGCTCCAAGAGTATCTATGAGTATTGCTGCAATTAAAAAGTTCGTRGT--TGATTA-------------------------------------------------------------------------------------------TTGTAATAA-------------------------------------TCTT---GTGATAA---------------GTA-------------TTAAA-TTAT------------------------------------------GATTARTGAAAGCCATGGAAGGAAATAGGATTAAATAGGGAGGGGTGAAATC-TGTAGATCTA-TTTAGGACTAACTGAAGCGAAAGCGATTTTCTATGTGGTATTTGACAATCAAGAACGAAAGCCGGA-GTATCGAAGACGATT--AGAGACCGTCGTAGTTC-CGYGCAGTAAACYATGTTATATTAT-TTGTAGT-A---TATATG----------------------------------------------AGTATA--TATTA-TGGATATATAGAAATTA-AGATATTATGAACTTTGGGGATAGTMCGAACGCAAGTTTTAAACTTAAAT-GAAATTGACGGAAGGACA-CACCAGGAGTGGAGTGTGCGGTTTAATTTGACTCAACGCGGG-ACAACTTACCATTTTTA-GAAGTGA-ATAT-GAATGATATTGTA-TCATGA-TTTTA--------------CTATGAGTGGTGCAYGGCCGTTAACAATACGTGATGTGAATTTTGAATTTGAATGAGTTATATATGAAAGTATATRATTCTGTATTAGTGTTAAATCCACC-AATGTGTGAGACCCATATAC------TAATTTTGA----------------------------GTTAGTAG-ACAGATGATG-TAAATCATAGGAAGGGATGGGCGATAACA-GGTCAGTGATGCCCTTAAATGAAATGGGCGACACGCGCACTACAATAGAAT-------------GATA-------TATT---------------------------------ATTCTAAGGAGTTGGGATTATTAATATGTAA--ATTTAATATGAACAAGGAATTCCTAGTAATATTTTTTTTGTGATGTTA-CAMCGAGATATGAATATGT-CCCTGTCCTTTGTACACACCGCCCGTCGTTATCTCAGATGGATATTAGGGTGAAATATTA------------------------------------------ATATAATAGAGAACTCTAATAACTAAATAAGGTACAA-----GTCGTAACAAGG-TA--ACC-----------------------------------------------------------------------------------------------------------------------------------------------------------

>>AJ438963_FR_Talorchestia_deshayesei_Msp-JES2002H_TER-04_1330pb_

------------GGTTGATTCTGCCTGACGTGGAAGCTATTCTTTAAGATTAAGCCATGCATGTGTAGAATGAAGTGAAGC-----CGTTAGGTGGAA-CAGCGAAAAGCTCAG-TAATACAATCATTATTTGGTCTAC-----AAGATAT-------AGA-ATAACCTTGATAA-ATTAAGGCTAAAGCTA-----------------------------------------------------------------------------------------------------------------------------TTGTAGAATAAGAGATTGACCTATCAGC--TA-GTATGTAGGGTAAGGGCCTACGTAGGCGATGACGGGTAACGGGGAATTAGGGTTCTATTCCGGAGAAGGAGG-CTGAGAGATGGCTACTAGGTCTAAGGAGAGCAGCAGGCGCGAAACTTACCCAATGCTA-TTTAGTAGTG---------TAGAGTGTA--GT-----------------------------GAATATTCT------------------AATGGAGAG---GCTAGTAA-------------------------AGCAACCATTGTAATTCCAGC----AAATCTATTACGAGTATTGCTGCAATTAAAAAGTTCGTAGT--TGATTA-------------------------------------------------------------------------------------------TTGTAATAA-------------------------------------TCTT---GTGATAA---------------GTA-------------TTAAA-TTAT------------------------------------------GATTAGTGAAAGCCATGGAAGGAAATAGGATTAAATAGGGAGGGGTGAAATC-TGTAGATCTA-TTTAGGACTAACTGAAGCGAAAGCGATTTTCTATGTGGTATTTGACAATCAAGAACGAAAGCCGGA-GTATCGAAGACGATT--AGAGACCGTCGTAGTTC-CGCGCAGTAAACCATGTTATATTAT-TTGTAGT-A---TATATG----------------------------------------------AGTATA--TATTA-TGGATATATAGAAATTA-AGATATTATGAACTTTGGGGATAGTACGAACGCAAGTTTTAAACTTAAAT-GAAATTGACGGAAGGACA-CACCAGGAGTGGAGTGTGCGGTTTAATTTGACTCAACGCGGG-ACAACTTACCATTTTTA-GAAGTGA-ATAT-GAATGATATT-TA-TCATGA-TTTTA--------------CTATGAGTGGTGCATGGCCGTTAACAATACGTGATGTGAATTTTGAATTTGAATGAGTTATATATGAAAGTATATAATTCTGTATTAGTGTTAAATCCACC-AATGTGTGAGACCCATATAC------TAATTTTGA----------------------------GTTAGTAG-ACAGATGATG-TAAATCATAGGAAGGGATGGGCGATAACA-GGTCAGTGATGCCCTTAAATGAAATGGGCGACACGCGCACTACAATAGAAT-------------GATA-------TATT---------------------------------ATTCTAAGGAGTTGGGATTATTAATATGTAA--ATTTAATATGAACAAGGAATTCCTAGTAATATTTTTTTTGTGATGTTAACAACGAGATATGAATATGT-CCCTGTCCTTTGTACACACCGCCCGTCGTTATCTCAGATGGATATTAGGGTGAAATATTG------------------------------------------ATATAATAGAGAACTCTAATAACTAAATAAGGTACCA-----GTCGTAACCAGG-TT--GACCTAAGTG------------------------AACTTGGGGCAGGATCAATA--------------------------------------------------------------------------------------------------------

>>AY635841_ND_ND_Basidiobolus_ranarum_JAM-06_1786pb_

---------------------------AGTCATATGCTTGTCTCAAAGATTAAGCCATGCATGTCTAAGTATAAACAAATT-----TGTACTGTGAAA-CTGCGAATGGCTCAT-TAAATCAGTTATAGTTTATTTGATAGTACCTTACTA-----CTTGG-ATAACCGTGGTAATTCTAGAGCTAATACAT--GCTAAAAATCCCGA------CTTCTGGAAGGGATGTATTTATTAGATAAAAAACCAACGTGGGCAACCACTTTTTAGGTGATTCATAATAACTTTTCGAATCGTATGACTTTACGTCGACGATGGTTCATTCAAATTTCTGCCCTATCAACTTTC-GATGGTAGGATAGAGGCCTACCATGGTTTTAACGGGTAACGGGGAATTAGGGTTCGATTCCGGAGAGGGAGC-CTGAGAAACGGCTACCACATCCAAGGAAGGCAGCAGGCGCGCAAATTACCCAATCCTG---ACACAGGGAGGTAGTGACAATAAATA--AC-AATACAGGGCTCTTTTGGGTCTTGTAATTGGAATGAG---------TACAATTTAAATCTCTTAACGAGGAACAATT---GGAGGGCAAG-TCTGGTGCCAGCAGCCGCGGTAATTCCAGCTCCAATAGCGTATATTAAAGTTGTTGCAGTTAAAAAGCTCGTAGT--TGAATTTTGGACCTCGGCCAGACGGTCTGCCTGTTTGGGTACGTACTGT----CTTGGCTAGGTCTTTCCTTCTGGAGAGCCTAGGTGTCGTTTACTCGGCGTCTAGGGGATCCAGGACTTTTACTTTGAA-AAAATT-----AGAG-TGTTTAAAGCAGGCTTACGCTTGAATACATTAGCATGGAATAATAGAATAGGACTTTGGTTCTATTTTGTTGGTTTCTAGGACCAAGGTAATGATTAATAGGGATAGTTGGGGGCATTAGTATTTAATTGTCAGAGGTGAAATT-CTTGGATTTA-TGAAAGACTAACTTCTGCGAAAGCATTTGCCAAGGATGTTTTCATTAATCAAGAACGAAAGTTAGG-GGATCGAAGACGATC--AGATACCGTCGTAGTCT-TA-ACCATAAACTATGCCGACTTGG-GATCGGT-C---AATGTTATTT---------------------------------------TATGACTTG--ATCGG-CACCATATGAGAAATC--AAAGTTTTTGGGTTCCGGGGGGAGTATGGTCGCAAGGCTGAAACTTAAA--GGAATTGACGGAAGGGCACCACCAGGAGTGGAGCCTGCGGCTTAATTTGACTCAACACGGG-GAAACTCACCAGGTCCA-GACATAG-TAAG-GATTGA-----CA-GATTGA-GAGCTCTTTCTTGATTCTATGGGTGGTGGTGCATGGCCGTTCTTAGTTGGTGGAGTGATTTG------------------------------------------TCTGGTTAATTCCGAT-AACGAACGAGACCTTAACCTGCTAAATAGTTACG--TTTACCTTTGTGTAGACGGACAACTTCTTAGAGGG-ACTGCTGATGTTTAATCAGTGGAAGTTTGAGGCAATAACA-GGTCTGTGATGCCCTTAGATGTTCTGGGCCGCACGCGCGCTACACTGATGA-A-----------ATCA-ACGAGTTTTTTCCTTGGCCGGAAGGTCTGGGTAATCTTGTAAAATTTCATCGTGCTGGGGATTGTCCATTGCAATTATTGGACATCAACGAGGAATTCCTAGTAAG--------CGCAAG-TCATCAGCTTGCGTTGATTACGT-CCCTGCCCTTTGTACACACCGCCCGTCGCTACTACCGATTGAATGGCTTAGTGAGACCTTCGGATTGGCGTTTGGCAGCTGGCAACAGCAGCTAATTGCTGAGAAGTTGGTCAAACTTGGTCATTTAGAGGAAGTAAAA-----GTCGTAACAAGG-TT--TCCGTAGGTG------------------------AACCTGCGGAAGGATCATTA--------------------------------------------------------------------------------------------------------

Alignment Figure 3 :

>>AJ011833_ND_Gdub-ce_Ngra_TER-99_787pb_AJ011833

--------------------------------ACTCTTATTTTATTTGATGTATTAGGATTATAACTATGTTAAATTATAGATAACAATAATACAATAAGAATAAGATCTATCAGTTAGTTGTTAAGGTAATGGCTTAACAAGACTATGACGGATAACGGTATTACTTTGTAATATTCCGGAGAAGGAGCCTGAGAGATTGCTACTAAGTCTAAGGATTGCAGCAGGGGCGAAACTTGACCTATGATATGATATTGAGGCAGTTATGAGTAGTATTTT-TAATTATTGTAATATTGTAAGTATATACTACAAGATAAATCGGAGGGCAAATCGAGTGCCAGCAGCCGCGGTAATACTTGTTCCGATAGTGTGTATGATGATTGATGCAGTTAAAAAGTCTGTAGTTTATGAT-TAATAAGCATTGTGAGGTATATTGTATGGTTAGGAGAGAGATGAAATGTGATAACCCTAACTGGATGAACAGAAGCGAAAGCTATATACTTAAATGTATTATTAGAACAAGGACGTAAGCTAGAGGATCGAAGATGATTAGATACCATTGTAGTTCTAGCAGTAAACTATGTTAAATCATAGATACA-TTT-TAATATGTATTTATGTAGAGAAATTAAGATTATATTGACTCTGGGGATAGTATGATCGCAAGATTGAAAATTAAAGAAATTGACGGAAGAATACCACAAGGAGTGGATTGTGCGGCTTAATTTGACTCAACGCGGGGTAATTTACCAGGTATCACATGATATAATATTTTATCATGATAGTGGTGCATGGCC--GTTTCCAATGGATGCTGTGAAGTAAT

>>PA-02_AT_Groe_Ngra01_ADR-18_687pb_PA-02-Ngra01

--------------------------------ACTCTTATTTTATTTGATGTATTAGGACTATAACTATGTTAAATTATAGATAACAATAATACAATAAGAATAAGATCTATCAGTTAGTTGTTAAGGTAATGGCTTAACAAGACTATGACGGATAACGGTATTACTTTGTAATATTCCGGAGAAGGAGCCTGAGAGATTGCTACTAAGTCTAAGGATTGCAGCAGGGGCGAAACTTGACCTATGATATGATATTGAGGCAGTTATGAGTAGTATTTT-TAATTATTGTAGTATTGTAAGTATATACTACAAGATAAATCGGAGGGCAAATCGAGTGCCAGCAGCCGCGGTAATACTTGTTCCGATAGTGTGTATGATGATTGATGCAGTTAAAAAGTCTGTAGTTTATGAT-TAATAAGCATTGTGAGGTATATTGTATGGTTAGGAGAGAGATGAAATGTGATAACCCTAACTGGATGAACAGAAGCGAAAGCTATATACTTAAATGTATTATTAGAACAAGGACGTAAGCTAGAGGATCGAAGATGATTAGATACCATTGTAGTTCTAGCAGTAAACTATGTTAAATCATAGATACA-TTT-TAATATGTATTTATGTAAAGAAATTAAGATTATATTGACTCTGGGGATAGTATGATCGCAAGATTGAAAATTAAAGAAATTGACGGAAGAATACCACAAGGAGTGGATTGTGCGGCTT------------------------------------------------------------------------------------------------------

>>GR11-06_GR_Groe_Ngra02_ADR-18_714pb_GR11-06-Ngra02

TTGAATATAAAGAAAAGACGAACAGCTCAGTAACTCTTATTTTATTTGATGTATTAGGATTATAACTATGTTAAATTATAGATAACAATAATACAATAAGAATAAGATCTATCAGTTAGTTGTTAAGGTAATGGCTTAACAAGACTATGACGGATAACGGTATTACTTTGTAATATTCCGGAGAAGGAGCCTGAGAGATTGCTACTAAGTCTAAGGATTGCAGCAGGGGCGAAACTTGACCTATGATATGATATTGAGGCAGTTATGAGTAGTATTTT-TAATTATTGTAGTATTGTAAGTATATACTACAAGATAAATCGGAGGGCAAATCGAGTGCCAGCAGCCGCGGTAATACTTGTTCCGATAGTGTGTATGATGATTGATGCAGTTAAAAAGTCTGTAGTTTATGAT-TAATAAGCATTGTGAGGTATATTGTATGGTTAGGAGAGAGATGAAATGTGATAACCCTAACTGGATGAACAGAAGCGAAAGCTATATACTTAAATGTATTATTAGAACAAGGACGTAAGCTAGAGGATCGAAGATGATTAGATACCATTGTAGTTCTAGCAGTAAACTATGTTAAATCATAGATACA-TTT-TAATATGTATTTATGTAAAGAAATTAAGATTATATTGACTCTGGGGATAGTATGATCGCAAGATTGAAAATTAAAGAAATTGACGGAAGAATACCACAAGGAGTGGATTGTGC-----------------------------------------------------------------------------------------------------------

>>JX213738_GB_Gdue_Ngra_IRO-13_316pb_JX213738

--------------------------------ACTCTTATTTTATTTGATGTATTAGGATTATAACTATGTTAAATTATAGATAACAATAATACAATAAGAATAAGATCTATCAGTTAGTTGTTAAGGTAATGGCTTAACAAGACTATGACGGATAACGGTATTACTTTGTAATATTCCGGAGAAGGAGCCTGAGAGATTGCTACTAAGTCTAAGGATTGCAGCAGGGGCGAAACTTGACCTATGATATGATATTGAGGCAGTTATGAGTAGTATTTT-TAATTATTGTAGTATTGTAAGTATATATTACAAGATAAATCGGAGGGCAAATCGAGTGCCAGCAGCCGCG--------------------------------------------------------------------------------------------------------------------------------------------------------------------------------------------------------------------------------------------------------------------------------------------------------------------------------------------------------------------------------------------------------------------------------------------------------------------------------------------

>>JX213744_GB_Gdue_Ngra_IRO-13_348pb_JX213744

TTGAATATAAAGAAAAGACGAACAGCTCAGTAACTCTTATTTTATTTGATGTATTAGGATTATAACTATGTTAAATTATAGATAACAATAATACAATAAGAATAAGATCTATCAGTTAGTTGTTAAGGTAATGGCTTAACAAGACTATGACGGATAACGGTATTACTTTGTAATATTCCGGAGAAGGAGCCTGAGAGATTGCTACTAAGTCTAAGGATTGCAGCAGGGGCGAAACTTGTCCTATGATATGATATTGAGGCAGTTATGAGTAGTATTTT-TAATTATTGTAGTATTGTAAGTATATATTACAAGATAAATCGGAGGGCAAATCGAGTGCCAGCAGCCGCG--------------------------------------------------------------------------------------------------------------------------------------------------------------------------------------------------------------------------------------------------------------------------------------------------------------------------------------------------------------------------------------------------------------------------------------------------------------------------------------------

>>JX213740_GB_Gdue_Ngra_IRO-13_316pb_JX213740

--------------------------------ACTCTTATTTTATTTGATGTATTAGGATTATAACTATGTTAAATTATAGATAACAATAATACAATAAGAATAAGATCTATCAGTTAGTTGTTAAGGTAATGGCTTAACAAGACTATGACGGATAACGGTATTACTTTGTAATATTCCGGAGAAGGAGCCTGAGAGATTGCTACTAAGTCTAAGGATTGCAGCAGGGGCGAAACTTGACCTATGATGTGATATTGAGGCAGTTATGAGTAGTATTTT-TAATTATTGTAGTATTGTAAGTATATACTACAGGATAAATCGGAGGGCAAATCGAGTGCCAGCAGCCGCG--------------------------------------------------------------------------------------------------------------------------------------------------------------------------------------------------------------------------------------------------------------------------------------------------------------------------------------------------------------------------------------------------------------------------------------------------------------------------------------------

>>GR25-02_GR_Groe_Ngra03_ADR-18_716pb_GR25-02-Ngra03

TTGAATATAAAGAAAAGACGAACAGCTCAGTAACTCTTATTTTATTTGATGTATTAGGATAATAACTATGTTAAATTATAGATAATAATAATACAATAAGAATAAGATCTATCAGTTAGTTGTTAAGGTAATGGCTTAACAAGACTATGACGGATAACGGTATTACTTTGTAATATTCCGGAGAAGGAGCCTGAGAGATTGCTACTAAGTCTAAGGATTGCAGCAGGGGCGAAACTTGACCTATGATATTATATTGAGGCAGTTATGAGTAGTATTTT-TAATTATTGTAATATTGTAAGTATATATTACAAGATAAATCGGAGGGCAAATCGAGTGCCAGCAGCCGCGGTAATACTTGTTCCGATAGTGTGTATGATGATTGATGCAGTTAAAAAGTCTGTAGTTTATTTTATAATAAGCATTGTGAGGTATATTGTATGGTTAGGAGAGAGATGAAATGTGATAACCCTAACTGGATGAACAGAAGCGAAAGCTATATACTTAAATGTATTATTAGAACAAGGACGTAAGCTAGAGGATCGAAGATGATTAGATACCATTGTAGTTCTAGCAGTAAACTATGTTAAATCATAGATATACTTT-TAATGTATATTTATGTAGAGAAATTAAGATTATATTGACTCTGGGGATAGTATGATCGCAAGATTGAAAATTAAAGAAATTGACGGAAGAATACCACAAGGAGTGGATTGTGC-----------------------------------------------------------------------------------------------------------

>>FN434088_IE_Gdub-ce_Ngra_KRE-10_789pb_FN434088

--------------------------------ACTCTTATTTAATTTGATGTATTAGGAAAATAACTATGTTAAATTATAGATAACAATAATACAATAAGAATAAGATCTATCAGTTAGTTGTTAAGGTAATGGCTTAACAAGACTATGACGGATAACGGTATTACTTTGTAATATTCCGGAGAAGGAGCCTGAGAGATTGCTACTAAGTCTAAGGATTGCAGCAGGGGCGAAACTTGACCTATGATATGATATTGAGGCAGTTATGAGTAGTATTTT-TAATTATTGTAGTATTGTAAGTATATACTACAAGATAAATCGGAGGGCAAATCGAGTGCCAGCAGCCGCGGTAATACTTGTTCCGATAGTGTGTATGATGATTGATGCAGTTAAAAAGTCTGTAGTTTATTAT-TAATAAGCATTGTGAGGTATATTGTATGGTTAGGAGAGAGATGAAATGTGATAACCCTAACTGGATGAACAGAAGCGAAAGCTATATACTTAAATGTATTATTAGAACAAGGACGTAAGCTAGAGGATCGAAGATGATTAGATACCATTGTAGTTCTAGCAGTAAACTATGTTAAATCATAGATACATTTTATAATATGTATTTATGTAAAGAAATTAAGATTATATTGACTCTGGGGATAGTATGATCGCAAGATTGAAAATTAAAGAAATTGACGGAAGAATACCACAAGGAGTGGATTGTGCGGCTTAATTTGACTCAACGCGGGGTAATTTACCAGGTATCACATGGTATAATATTTTATCATGATAGTGGTGCATGGCC--GTTTTCAATGGATGCTGTGAAGTAAT

>>KM657356_PL_Gpul_Ngra_WRO-UN_789pb_KM657356

--------------------------------ACTCTTATTTAATTTGATGTATTAGGAAAATAACTATGTTAAATTATAGATAACAATAATACAATAAGAATAAGATCTATCAGTTAGTTGTTAAGGTAATGGCTTAACAAGACTATGACGGATAACGGTATTACTTTGTAATATTCCGGAGAAGGAGCCTGAGAGATTGCTACTAAGTCTAAGGATTGCAGCAGGGGCGAAACTTGACCTATGATATGATATTGAGGCAGTTATGAGTAGTATTTT-TAATTATTGTAGTATTGTAAGTATATACTACAAGATAAATCGGAGGGCAAATCGAGTGCCAGCAGCCGCGGTAATACTTGTTCCGATAGTGTGTATGATGATTGATGCAGTTAAAAAGTCTGTAGTTTATTAT-TAATAAGCATTGTGAGGTATATTGTATGGTTAGGAGAGAGATGAAATGTGATAACCCTAACTGGATGAACAGAAGCGAAAGCTATATACTTAAATGTATTATTAGAACAAGGACGTAAGCTAGAGGATCGAAGATGATTAGATACCATTGTAGTTCTAGCAGTAAACTATGTTAAATCATAGATACATTTTATAATATGTATTTATGTAAAGAAATTAAGATTATATTGACTCTGGGGATAGTATGATCGCAAGATTGAAAATTAAAGAAATTGACGGAAGAATACCACAAGGAGTGGATTGTGCGGCTTAATTTGACTCAACGCGGGGTAATTTACCAGGTATCACATGATATAATATTTTATCATGATAGTGGTGCATGGCC--GTTTCCAATGGATGCTGTGAAGTAAT

>>DQ073396_ND_Antheraea_pernyi_Nant_WAN-06_790pb_Outgroup

--------------------------------ACTCTTATTTGATTTGATGTATTAGGACTCTAACTATGTTAAATTATAGGTAACAATAATACAATAAGAATAAGATCTATCAGTTAGTTGTTAAGGTAATGGCTTAACAAGACTATGACGGATAACGGTATTACTTTGTAATATTCCGGAGAAGGAGCCTGAGAGATTGCTACTAAGTCTAAGGATTGCAGCAGGGGCGAAACTTGACCTATGATATTATATTGAGGCAGTTATGAGTAGTATTTTATAATTATTGTAGTATTGTAAGTACATATTACAAGATAAATCGGAGGGCAAATCGAGTGCCAGCAGCCGCGGTAATACTTGTTCCGATAGTGTGTATGATGATTGATGCAGTTAAAAAGTCTGTAGTTTATTTA-TAATAAGCATTGTAAGGTATACTGTATGGTTAGGAGAGAGATGAAATGTGATAACCCTAACTGGATGAACAGAAGCGAAAGCTGTATACTTAAATGTATTATTAGAACAAGGACGTAAGCTAGAGGATCGAAGATGATTAGATACCATGGTAGTTCTAGCAGTAAACTATGTTGAATCATAGATATA-TTT-TGATATATATTTATGTAGAGAAATTAAGATTATATTGACTCTGGGGATAGTATGATCGCAAGATTGAAAATTAAAGAAATTGACGGAAGAATACCACAAGGAGTGGATTGTGCGGCTTAATTTGACTCAACGCGGGGTAATCTACCAGATATAACATGATATAATATTTTATCATGATAGTGGTGCATGGCCCGTTTCCCAATGGATGCTGTGAAGTAAT

Alignment Figure 4 :

>>AJ252961_US_Aedes_albopictus_Vavraia_culicis_CHE-00_1312pb_hap

--------------------CATGTGCAAGCGAAGCCGGTGGTGGAGCGGCGCAAGGCTCAGTAACGGGCGACTGATTTGATCTCCCGGTG-------TGGACAACCTCTATAACCGGAGGCCAAAACACAGGAGGCGCGTGACACGGG--CACGGGCGTGCAACC---------GGCAGGTGCGGGAGAGTAAGGAGCCATCCCATCAGTTAGTAAGTAGGGTAAGGGCCTACTTAGACGAAGACGGGTACGGGGAATTAGAGTTCGATTCCGGAGAGGGAGCCTGAGAGACGGCTACCAGGTCCAAGGACAGCAGCAGGCGCGGAAATTACCGAAGCCCGCGTTCGGGGCGGTAGTAAGGAGACGTG---AATA-CGATGTGCAGGTAAAATATGCACTGGTAACAGGAGGTCAAGACTGGTGCCAGCATCCGCGGTAATACCAGCTCCTGGAGTGTCTATGACGATTGTTGCAGTTAAAGAGTTCGTAGTCGGGCTGCATGACCGGCGCTGAAGGCTCCCTATCGAGGGGGGCAGGGCGCCGGAAAAG-CAGAGGATGAGGAGCGGCCGGGGGCCAGGTTATTAAGCGACGAGAGGTGAAATTTGATGACTCGCTTAGGAGCAGCAGAGGCGAAAGCGCTGGCCAGGGGCGAATCCGATGATAAAGGACGTAGGCTAGAGGATCGAAGACGATTAGAGACCGTTGTAGTTCTAGCAGTAAACGATGCCGATGCCGTGGGGCGGTGCAGCCGCCACGCGGAGGAGAAATTGAGTAGGGCCCTGGGGAGAGTACACGCGCAAGCGGGAAATTTAAAGGAAATTGACGGAAGAACACCACAAGGAGTGGAGTGTGCGGCTTAATTTGACTCAACGC-GGACAGCTTACCAGGCCCGACGGTCGCACGAGTGTGGTACACGATAGGCCGGAGAGTGGTGCATGGCCGTTAACGACAAGTGGGGTGACCTTTGGGTTAAGTCCGGGAAGTAGTGAGACCCCTGCCG-GCGCGCCGGGACAGGTGCTCAAAGCACAGGAAGGAAGGGTCAAGAACAGGTCAGTGATGCCCTTAGATGGTCTGGGCTGCACGCGCACTACAGTGGTCGCAGAAATCAG--------TGCCCGAGCGGCAATGGCGATCGAGAGGGAACGAGCTCTGGAAGGGGCTCGGGAACGAGGAATTGCTAGTAATCGTGGGCTCATTAAGACACGGTGAATACGTCCCTGTTCTTTGTACACACCGCCCGTCGTTATCGAAGATGGAGTCAGGCGCGAACAAGCGAGAGCGAGTGAGT---------------------------------------------------------

>>AJ438962_GB_Gche_Msp-JES2002G_TER-04_1327pb_AJ438962

---------ATTTAGCCATGCATGTGTAAGCGAA--CAATTAGGGAGCTGCGGACTGCTCAGTAACAGGCGATTAATTTAATCTTTACAAA-------CGGACAAACTCAGGAAACGGAGTGTAATACGTAAAAGATGATTTTTTATTTAAAAAAAGAAAACATTTTTAGCTTGAATAAAGCGGTAAAGAATAAGACGCCAACCCATCAGTTAGTAAGTAGGGTAAGGGCCTATTTAGACGAATACGGGTACGGGGAATTAGGGTTTGATTCCGGAGAGGGAGCCTGAGAGACGGCTACCAGGTCCAAGGACGGCAGCAGGCGCGAAAATTACCGAAGCTCA-AATAGAGGCGGTAGTAATGAGACGTATAAATAA-TAATATAAGGGTAAAAAACTTATTAATAACTGGAGGTCAAGTCTGGTGCCAGCATCCGCGGTAATACCAGCTCCAGGGGTGTCTATGATGATTGCTGCGATTAAAAGGTCCGTAGTCGAATTTAA--ATAATTGTTTGTAATATGTTAGATAAAATAATAAAAAGAACAATTAC-TTAAATGAAAGGAATAACAAGAGGTTGATTAATTGAGTAACGAGAGGTGAAATTTGATGACTTACTTAGGAGAAACAGAGGCGAAAGCGTCAATCAAGTGTAAATCCGATGATCAAGGACGTAGGCTGGAGTATCGAACACGATTAGATACCGTAGTAGTTCCAGCAGTAAACTATGCCTACGCCAATAAATAA---AAGTTTAATGGACGAGAAATCTAGAGTAGGGCTTTGGGGAGAGTACACGCGCAAGCGATAAATTTAAAGGAAATTGACGGAAGAACACCACAAGGAGTGGAGTGTGCGGCTTAATTTGACTCAACGCGGGACAGCTTACCAAACCCGAAAACTAAAAGAGTG-AATACACGATAAGTTTAAGAGTGGTGCATGGCCGTTATCGACGAGTGAAGTGATTTTATGGTTAAATCCGACAAGTCGTGAGACCCTATAAATAATTAATAAAACAGGTA-TGAAAATACAGGAAGGATAGGACAAGAACAGGTCAGTGATGCCCTTAAATGGTTTGGGCTGCACGCGCACTACAGTGGTTATAATAAAATATTATAGAAAATTAAATAAAGAAATATAATCAAGAGGGATTGAGTATTGAAAAATACCCATGAACGAGGAATTGCTAGTAATCGTAGGCTCAGTAAGATACGATGAATATGTCCCTGTTCTTTGTACACACCGCCCGTCGTTATCGAAGATGGAGTTTTGCCCGAACGAGCTTTAGCGAGTAAGTGTATGATTCTAGATCTGATACAAGTCGTAACAAGGCAGCTGTAGGAGAACCTGTAGC

>>KR871368_DE_Gpul_Msp-G_GRA-15_786pb_KR871368

------------------------------CGAA--CAATTAGGGAGCTGCGGACTGCTCAGTAACAGGCGATTAATTTAATCTTTACAAA-------CGGACAAACTCAGGAAACGGAGTGTAATACGTAAAAGATGATTTTTTATTT-AAAAAAGAAAACATTTTTAGCTTGAATAAAGCGGTAAAGAATAAGACGCCAACCCATCAGTTAGTAAGTAGGGTAAGGGCCTATTTAGACGAATACGGGTACGGGGAATTAGGGTTTGATTCCGGAGAGGGAGCCTGAGAGACGGCTACCAGGTCCAAGGACGGCAGCAGGCGCGAAAATTACCGAAGCTCA-AATAGAGGCGGTAGTAATGAGACGTATAAATAA-TAATATAAGGGTAAAAAACTTATTAATAACTGGAGGTCAAGTCTGGTGCCAGCATCCGCGGTAATACCAGCTCCAGGGGTGTCTATGATGATTGCTGCGATTAAAAGGTCCGTAGTCGAATTTAA--ATAATTGTTTGTAATATGTTAGATAAAATAATAAAAAGAACAATTAC-TTAAATGAAAGGAATAACAAGAGGTTGATTAATTGAGTAACGAGAGGTGAAATTTGATGACTTACTTAGGAGAAACAGAGGCGAAAGCGTCAATCAAGTGTAAATCCGATGATCAAGGACGTAGGCTGGAGTATCGAACACGATTAGATACCGTAGTAGTTCCAGCAGTAAACTATGCCTACGCCAATAAATAA---AAGTTTAATGGACGAGAAATCTAGAGTAGGGCTTTGGGGAGAGTACACGCGCAAGCGATAAATTTAAAGGAAATTGACGGAAGAA-----------------------------------------------------------------------------------------------------------------------------------------------------------------------------------------------------------------------------------------------------------------------------------------------------------------------------------------------------------------------------------------------------------------------------------------------------------------------------------------------------------------------------------------

>>ALP53-04_AT_Groe_Corn_ADR-18_407pb_ALP53-04-Corn

-------------------GCATGTGTAAGCGAA--CAATTAGGGAGCTGCGGACTGCTCAGTAACAGGCGATTAATTTAATCTTTACAAA-------CGGACAAACTCAGGAAACGGAGTGTAATACGTAAAAGATGATTTTTTATTT-AAAAAAGAAAACATTTTTAGCTTGAATAAAGCGGTAAAGAATAAGACGCCAACCCATCAGTTAGTAAGTAGGGTAAGGGCCTATTTAGACGAATACGGGTACGGGGAATTAGGGTTTGATTCCGGAGAGGGAGCCTGAGAGACGGCTACCAGGTCCAAGGACGGCAGCAGGCGCGAAAATTACCGAAGCTCA-AATAGAGGCGGTAGTAATGAGACGTATAAATAA-TAATATAAGGGTAAAAAACTTATTAATAACTGGAGGTCAAGTCTGGTGCCAGCAGCCGCGG-----------------------------------------------------------------------------------------------------------------------------------------------------------------------------------------------------------------------------------------------------------------------------------------------------------------------------------------------------------------------------------------------------------------------------------------------------------------------------------------------------------------------------------------------------------------------------------------------------------------------------------------------------------------------------------------------------------------------------------------------------------------------------------------------------------------------------------------------------------------------------------------------------------------------------------------------------

>>KR190602_GB_Dhae_Corn_BOJ-15_1186pb_KR190602

GTCTCATAGATTTAGCCATGCATGTGTAAGCGAA--CAATTAGGGAGCTGCGGACTGCTCAGTAACAGGCGATTAATTTAATCTTTACAAA-------TGGACAAACTCAGGAAACGGAGTGTAATACATAAAAAATGATTTTTTAAAT--AAAAAGAAAACATTTTTAGCTTGAAAAAAGCGGTAAAGAATAAGACGCCAACCCATCAGTTAGTAAGTAGGGTAAGGGCCTATTTAGACGAATACGGGTACGGGGAATTAGGGTTTGATTCCGGAGAGGGAGCCTGAGAGACGGCTACCAGGTCCAAGGACGGCAGCAGGCGCG-AAATTACCGAAGCTCA-AATAGAGGCGGTAGTAATGAGACGTATAAAAAA-TAATATAAGGGTAAAAAACTTATTAATAACTAGAGGTCAAGTCTGGTGCCAGCATCCGCGGTAATACCAGCTCTAGGGGTGTCTATGATGATTGCTGCGATTAAAAGGTCCGTAGTCGAATTTAA--ATAATTGTTTGTAATATGTTAGATAAAATAATAAAAAGAACAATTAC-TTAAATGAAAGGAATAACAAGAGGTTGATTAATTGAGTAACGAGAGGTGAAATTTGATGACTTACTTAGGAGAAACAGAGGCGAAAGCGTCAATCAAGTGTAAATCCGATGATCAAGGACGTAGGCTGGAGTATCGAACACGATTAGATACCGTAGTAGTTCCAGCAGTAAACTATGCCTACGCCAATGAATGA---AAGTTTGATGGACGAGAAATCTAGAGTAGGGCTTTGGGGAGAGTACACGCGCAAGCGATAAATTTAAAGGAAATTGACGGAAGAACACCACAAGGAGTGGAGTGTGCGGCTTAATTTGACTCAACGCGGGACAGCTTACCAAACCCGAAAACTAAAAGAGTG-AATACACGATAAGTTTAAGAGTGGTGCATGGCCGTTATCGACGAGTGAAGTGATTTTATGGTTAAATCCGACAAGTCGTGAGACCCTATAA------AATAAAACAGGTA-TGAAAATACAGGAAGGATAGGACAAGAACAGGTCAGTGATGCCCTTAGATGGTTTGGGCTGCACGCGCACTACAGTGGTTATAATAAAATA-TATAGAAAACTAAATAAAGAAATATAATCAAGAGGGATTGAGTATTGAAAAATACCCATGAACGAGGAATTGCTAGTAATCGTAGGCTCAGTAAGATACG--------------------------------------------------------------------------------------------------------------------------------------------

>>KR871369_DE_Dhae_Msp-G_GRA-15_960pb_KR871369

---------------------------------------TTAGGGAGCTGCGGACTGCTCAGTAACAGGCGATTAATTTAATCTTTACAAA-------TGGACAAACTCAGGAAACGGAGTGTAATACATAAAAAATGATTTTTTAAAT--AAAAAGAAAACATTTTTAGCTTGAAAAAAGCGGTAAAGAATAAGACGCCAACCCATCAGTTAGTAAGTAGGGTAAGGGCCTATTTAGACGAATACGGGTACGGGGAATTAGGGTTTGATTCCGGAGAGGGAGCCTGAGAGACGGCTACCAGGTCCAAGGACGGCAGCAGGCGCGAAAATTACCGAAGCTCA-AATAGAGGCGGTAGTAATGAGACGTATAAAAAA-TAATATAAGGGTAAAAAACTTATTAATAACTAGAGGTCAAGTCTGGTGCCAGCATCCGCGGTAATACCAGCTCTAGGGGTGTCTATGATGATTGCTGCGATTAAAAGGTCCGTAGTCGAATTTAA--ATAATTGTTTGTAATATGTTAGATAAAATAATAAAAAGAACAATTAC-TTAAATGAAAGGAATAACAAGAGGTTGATTAATTGAGTAACGAGAGGTGAAATTTGATGACTTACTTAGGAGAAACAGAGGCGAAAGCGTCAATCAAGTGTAAATCCGATGATCAAGGACGTAGGCTGGAGTATCGAACACGATTAGATACCGTAGTAGTTCCAGCAGTAAACTATGCCTACGCCAATGAATGA---AAGTTTGATGGACGAGAAATCTAGAGTAGGGCTTTGGGGAGAGTACACGCGCAAGCGATAAATTTAAAGGAAATTGACGGAAGAACACCACAAGGAGTGGAGTGTGCGGCTTAATTTGACTCAACGCGGGACAGCTTACCAAACCCGAAAACTAAAAGAGTG-AATACACGATAAGTTTAAGAGTGGTGCATGGCCGTTATCGACGAGTGAAGTGATTTTATGGTTAAATCCGACAAGTCGTGAGACCCTATAA------AATAAAACAGGTA-T-------------------------------------------------------------------------------------------------------------------------------------------------------------------------------------------------------------------------------------------------------------------------------------------------------------------------------------------

>>KR871366_DE_Dhae_Msp-G_GRA-15_940pb_KR871366

---------------------------------------TTAGGGAGCTGCGGACTGCTCAGTAACAGGCGATTAATTTAATCTTTACAGAAACGAGCGGAATAAACTCAGGAAACGGAGTGTAATACGTAAAAGATGAATTTTTTATTTTAAGAAGAAAACATTTTTAGCTTGAAAAAAGCGGTAAAGAATAAGACGCCAACCCATCAGTTAGTAAGTAGGGTAAGGGCCTATTTAGACGAATACGGGTACGGGGAATTAGGGTTTGATTCCGGAGAGGGAGCCTGAGAGACGGCTACCAGGTCCAAGGACGGCAGCAGGCGCGAAAATTACCGAAGCTCA-AATAGAGGCGGTAGTAATGAGACGTATAAAAAA-TAATATAAGGGTAAAAAACTTATTAATAACTAGAGGTCAAGTCTGGTGCCAGCATCCGCGGTAATACCAGCTCTAGGGGTGTCTATGATGATTGCTGCGATTAAAAGGTCCGTAGTCGAATTTAA--ATAATTGTTTGTAATATGTTAGATAAAATAATAAAAAGAACAATTAC-TTAAATGAAAGGAATAACAAGAGGTTGATTAATTGAGTAACGAGAGGTGAAATTTGATGACTTACTTAGGAGAAACAGAGGCGAAAGCGTCAATCAAGTGTAAATCCGATGATCAAGGACGTAGGCTGGAGTATCGAACACGATTAGATACCGTAGTAGTTCCAGCAGTAAACTATGCCTACGCCAATGAATGA---AAGTTTGATGGACGAGAAATCTAGAGTAGGGCTTTGGGGAGAGTACACGCGCAAGCGATAAATTTAAAGGAAATTGACGGAAGAACACCACAAGGAGTGGAGTGTGCGGCTTAATTTGACTCAACGCGGGACAGCTTACCAAACCCGAAAACTAAAAGAGTG-AATACACGATAAGTTTAAGAGTGGTGCATGGCCGTTATCGACGAGTGAAGTGATTTTATGGTTAAATCCGACAAGT-------------------------------------------------------------------------------------------------------------------------------------------------------------------------------------------------------------------------------------------------------------------------------------------------------------------------------------------------------------------------------

>>KR871367_DE_Groe_Msp-G_GRA-15_952pb_KR871367

---------------------------------------TTAGGGAGCTGCGGACTGCTCAGTAACAGGCGATTAATTTAATCTTTACAGAAACGAGCGGAATAAACTCAGGAAACGGAGTGTAATACGTAAAAGATGAATTTTTTATTTTAAGAAGAAAACATTTTTAGCTTGAATAAAGCGGTAAAGAATAAGACGCCAACCCATCAGTTAGTAAGTAGGGTAAGGGCCTATTTAGACGAATACGGGTACGGGGAATTAGGGTTTGATTCCGGAGAGGGAGCCTGAGAGACGGCTACCAGGTCCAAGGACGGCAGCAGGCGCGAAAATTACCGAAGCTCA-AATAGAGGCGGTAGTAATGAGACGTATAAATAA-TAATATAAGGGTAAAAAACTTATTAATAACTGGAGGTCAAGTCTGGTGCCAGCATCCGCGGTAATACCAGCTCCAGGGGTGTCTATGATGATTGCTGCGATTAAAAGGTCCGTAGTCGAATTTAA--ATAATTGTTTGTAATATGTTAGATAAAATAATAAAAAGAACAATTAC-TTAAATGAAAGGAATAACAAGAGGTTGATTAATTGAGTAACGAGAGGTGAAATTTGATGACTTACTTAGGAGAAACAGAGGCGAAAGCGTCAATCAAGTGTAAATCCGATGATCAAGGACGTAGGCTGGAGTATCGAACACGATTAGATACCGTAGTAGTTCCAGCAGTAAACTATGCCTACGCCAATAAATAA---AAGTTTAATGGACGAGAAATCTAGAGTAGGGCTTTGGGGAGAGTACACGCGCAAGCGATAAATTTAAAGGAAATTGACGGAAGAACACCACAAGGAGTGGAGTGTGCGGCTTAATTTGACTCAACGCGGGACAGCTTACCAAACCCGAAAACTAAAAGAGTG-AATACACGATAAGTTTAAGAGTGGTGCATGGCCGTTATCGACGAGTGAAGTGATTTTATGGTTAAATCCGACAAGTCGTGAGACCCTA-------------------------------------------------------------------------------------------------------------------------------------------------------------------------------------------------------------------------------------------------------------------------------------------------------------------------------------------------------------------

>>FN434092_GB_Gdub-du_Croe_KRE-10_1249pb_FN434092

-----------------------GTGTAAGCGAA---CGAGAGGGAGCTGCGGACTGCTCAGTAACAGGCGAATAATTTAATCTTTACAAA-------CGGACAAACTCAGGAAACGGAGTGAAATACGAAAAAGTTGATATTTTTAT---AAAAAGAAATCGAACTTAGCTTGAA-AAAGCGGTAAAGAATAAGACGCCAGCCCATCAGTTAGTAAGTAAGGTAAGGGCTTATTTAGACGAATACGGGTACGGGGAATTAGGGTTTGATTCCGGAGAGGGAGCCTGAGAGACGGCTACCAGGTCCAAGGACGGCAGCAGGCGCGAAAATTACCGAAGCTCA-AAAAGAGGCGGTAGTAATGAGACGTA-AAAAAATTAATACAAGGGTAAAAAACTTGTTAATAACTGGAGGTAAAGTCTGGTGCCAGCATCCGCGGTAATACCAGCTCCAGGGGTGTCTATGATGATTGCTGCGATTAAAAGGTCCGTAGTCGAAAATAG--ATAATTGTTTGTAAGATGTTATCTAAAATAATAGAAAGAACAATTAC-CTAATTGAAAGGAATAGCAAGAGGCTGATTAATCGAGTAACGAGAGGTGAAATTTGATGACTTACTTGGGAGAAACAGAGGCGAAAGCGTCAGTCAAGTGTAAATCCGATGATCAAGGACGTAGGCTGGAGTATCGAACACGATTAGATACCGTAGTAGTTCCAGCAGTAAACTATGCCTACGCCGTTGAATGA---AAGTTTGGCGGACGAGAAATTTAGAGTAGGGCTTTGGGGAGAGTACACGCGCAAGCGATAAATTTAAAGGAAATTGACGGAAGAACACCACAAGGAGTGGAGTGTGCGGCTTAATTTGACTCAACGCGGGACAGCTTACCAAACCCGAGGACCAAAAGAGTG-AATACACGATAGGTTTAAAAGTGGTGCATGGCCGTTATCGACGAGTGAAGTGATTTTATGGTTAAATCCGACAAGTCGTGAGACCCTAAAAAAA---AAATTAACAGGTA-TGAAAATACAGGAAGGATAGGACAAGAACAGGTCAGTGATGCCCTTAGATGGTTTGGGCTGCACGCGCACTACAGTGGTTATAAAAAAAAA-------GAAAAAAGAAAATAAATATAATCAAGAGGAATTGAGCACTGAAAAGTGCCCATGAACGAGGAATTGCTAGTAATCGTAGGCTCAGTAAGATACGATGAATATGTCCCTGTTCTTTGTACACACCGCCCGTCGTTATCGAAGATGGAGTTTTGCCCGAACGAGCTTTAGCGAGTAAGTGTATGATT-------------------------------------------------

>>KX137904_DE_Ephemera_danica_Msp-groupD_GRA-17_393pb_KX137904

-----------------------GTGTAAGCGAA---CGAGAGGGAGCTGCGGACTGCTCAGTAACAGGCGAATAATTTAATCTTTACAAA-------CGGACAAACTCAGGAAACGGAGTGAAATACGAAAAAGTTGATATTTTTAT---AAAAAGAAATCGAACTTAGCTTGAA-AAAGCGGTAAAGAATAAGACGCCAGCCCATCAGTTAGTAAGTAAGGTAAGGGCTTATTTAGACGAATACGGGTACGGGGAATTAGGGTTTGATTCCGGAGAGGGAGCCTGAGAGACGGCTACCAGGTCCAAGGACGGCAGCAGGCGCGAAAATTACCGAAGCTCA-AAAAGAGGCGGTAGTAATGAGACGTA-AAAAAATTAATACAAGGGTAAAAAACTTGTTAATAACTGGAGGTAAAGTCTGGTGCCAGCAC-----------------------------------------------------------------------------------------------------------------------------------------------------------------------------------------------------------------------------------------------------------------------------------------------------------------------------------------------------------------------------------------------------------------------------------------------------------------------------------------------------------------------------------------------------------------------------------------------------------------------------------------------------------------------------------------------------------------------------------------------------------------------------------------------------------------------------------------------------------------------------------------------------------------------------------------------------------

>>PL5-10_PL_Groe_Croe_ADR-18_818pb_PL5-10-Croe

------------------------TGTAAGCGAA---CGAGAGGGAGCTGCGGACTGCTCAGTAACAGGCGAATAATTTAATCTTTACAAA-------CGGACAAACTCAGGAAACGGAGTGAAATACGAAAAAGTTGATATTTTTAT---AAAAGAAAATCGAAACTAGCTTGAA-AAAGCGGTAAAGAATAAGACGCCAGCCCATCAGTTAGTAAGTAGGGTAAGGGCCTATTTAGACGAATACGGGTACGGGGAATTAGGGTTTGATTCCGGAGAGGGAGCCTGAGAGACGGCTACCAGGTCCAAGGACGGCAGCAGGCGCGAAAATTACCGAAGCTCG-AAATGAGGCGGTAGTAATGAGACGTA-AATAAA-TAATACAAGGGTAAAAAACTTGTTAATAACTGGAGGTCAAGTCTGGTGCCAGCATCCGCGGTAATACCAGCTCCAGGGGTGTCTATGATGATTGCTGCGATTAAAAGGTCCGTAGTCGAAAATAG--ATAATTGTTTGTAAGATGTTATCTAAAATAATAGAAAGAACAATTAC-CTAATTGAAAGGAATAGCAAGAGGCTGGTTAATCGAGTAACGAGAGGTGAAATTTGATGACTTACTTGGGAGAAACAGAGGCGAAAGCGCCAGTCAAGTGTAAATCCGATGATCAAGGACGTAGGCTGGAGTATCGAACACGATTAGATACCGTAGTAGTTCCAGCAGTAAACTATGCCTACGCCGTTGAATGA---AAGTTTGGCGGACGAGAAATTTAGAGTAGGGCTTTGGGGAGAGTACACGCGCAAGCGATAAATTTAAAGGAAATTGACGGAAGAACACCACAAGGAGTGGAGTGTGCGGCTTAATT----------------------------------------------------------------------------------------------------------------------------------------------------------------------------------------------------------------------------------------------------------------------------------------------------------------------------------------------------------------------------------------------------------------------------------------------------------------------------------------------------------

>>KY200851_PL_Groe_Croe_BOJ-17_823pb_KY200851

--------------------------------------------------------------------------------------------------------------------------------------------------------------------------------------------------------------------------------------------------------------------------------------------------GACGGCTACCAGGTCCAAGGACGGCAGCAGGCGCGAAAATTACCGAAGCTCG-AAATGAGGCGGTAGTAATGAGACGTA-AATAAA-TAATACAAGGGTAAAAAACTTGTTAATAACTGGAGGTCAAGTCTGGTGCCAGCATCCGCGGTAATACCAGCTCCAGGGGTGTCTATGATGATTGCTGCGATTAAAAGGTCCGTAGTCGAAAATAG--ATAATTGTTTGTAAGATGTTATTTAAAATAATAGAAAGAACAATTAC-CTAATTGAAAGGAATAGCAAGAGGCTGGTTAATCGAGTAACGAGAGGTGAAATTTGATGACTTACTTGGGAGAAACAGAGGCGAAAGCGCCAGTCAAGTGTAAATCCGATGATCAAGGACGTAGGCTGGAGTATCGAACACGATTAGATACCGTAGTAGTTCCAGCAGTAAACTATGCCTACGCCGTTGAATGA---AAGTTTGGCGGACGAGAAATTTAGAGTAGGGCTTTGGGGAGAGTACACGCGCAAGCGATAAATTTAAAGGAAATTGACGGAAGAACACCACAAGGAGTGGAGTGTGCGGCTTAATTTGACTCAACGCGGGACAGCTTACCAAACCCGAGGACCAAAAGAGTG-AATACACGATAGGTTTAAGAGTGGTGCATGGCCGTTATCGACGAGTGAAGTGATTTTATGGTTAAATCCGACAAGTCGTGAGACCCTA---------AAATAAACAGGTA-TGAAAATACAGGAAGGATAGGACAAGAACAGGTCAGTGATGCCCTTAGATGGTTTGGGCTGCACGCGCACTACAGTGGTTATAAAAAAAAA-------GAAAAAAGAAAATAAATAT-----------------------------------------------------------------------------------------------------------------------------------------------------------------------------------------------------------------------

>>GQ258752_PL_Dvil_Cdik_OVC-10_1291pb_GQ258752

--------------------CATGTGTAAGCGAA--CA-AGAGGAAGCTGCGGACTGCTCAGTAACAGACATATAATTTAATCTTTACAGAAACGAGCGGAATAAACTCAGGAAACAGAGTGCAATACGTAAAAGACGAATTTTTTATT--ATAAGAAATACGTTTTTAGCTTGAACAAAGCGGTAAAGAATAAGTTGTCAGCCTATCAGTTAGTAAGTAGGGTAAGGGCCTATTTAGACGAAGACGGGTACGGGGAATTAGAGTTTGATTCCGGAGAGGGAGCCTGAGAAATAGCTACCAGGTCCAAGGACGGCAGCAGGCGCGAAAATTACCGAAGCTCG-AATAGAGGCGGTAGTAATGAGACGTA-TTAATA-TAAAACAAGGGTAAAAAACTTGTTAGTAACTGGAGGTCAAGTCTGGTGCCAGCATCCGCGGTAATACCAGCTCCAGGGGTGTCTATGATGATTGCTGCGATTAAAAGGTCCGTAGTCGAATTTAT--ATAATTGTTTGTAATATGCTAGATAAAATAACAGAAAGAACAATTACTTTAAATGAAAGGAATAGTAAGGGGCTGATTAATTGAGCAACGAGAGGTGAAATTTGATGACTTGCTTAGGAGAAACAGAGGCGAAAGCGTCAGTCAAGTATAAATCCTATGATCAAGGACGTAGGCTAGAGTATCGAACACGATTAGATACCGTAGTAGTTCTAGCAGTAAACTATGCCTACACTATCGAATAA---AAGTTTGGTAGAAGAGAAATCTTAAGTAGGGCTTTGGGGAGAGTACACGCGCAAGCGATAAATTTAAAGGAAATTGACGGAAGAACACCACAAGGAGTGGAGTGTGCGGCTTAATTTGACTCAACGCGGGACAGCTTACCATACCCGAGGACTATAAGAGTG-AATACACGATAAGTCTAAAAGTGGTGCATGGCCGTTATCGACGAGTGAAGTGATTTTATGGTTAAATCCGACAAGTTGTGAGACCCTTATTT-A---AATACAGGTATTG-TTAAAATACAGGAAGGAAAGGACAAGAACAGGTCAGTGATGCCCTTAGATGGTATGGGCTGCACGCGCACTACAATGGTTATAATAATAAA------GATAATTAAAGTATAAATATAATCAAGAGGAATTGAGAACTGAAAAGTTCCCATGAACGAGGAATTGCTAGTAATCGTAGGCTCAGTAAGATACGATGAATATGTCCCTGTTCTTTGTACACACCGCCCGTCGTTATCGAAGATGGAGTTTTACCCGAACAAGCTTAAGCGAGTGAGTGTATGATTCTAGATCTGATACAAGTCGTAACAAGGCAG-------------------

>>GRR-15_FR_Groe_Cdik_ADR-18_385pb_GRR-15-Cdik

-----------------------------------------------CTGCGGACTGCTCAGTAACAGACATATAATTTAATCTTTACAGAAACGAGCGGAATAAACTCAGGAAACAGAGTGCAATACGTAAAAGACGAATTTTTTATT--ATAAGAAATACGTTTTTAGCTTGAACAAAGCGGTAAAGAATAAGTTGTCAGCCTATCAGTTAGTAAGTAGGGTAAGGGCCTATTTAGACGAAGACGGGTACGGGGAATTAGAGTTTGATTCCGGAGAGGGAGCCTGAGAAATAGCTACCAGGTCCAAGGACGGCAGCAGGCGCGAAAATTACCGAAGCTCG-AATAGAGGCGGTAGTAATGAGACGTA-TTAATA-TAAAACAAGGGTAAAAAACTTGTTAGTAACTGGAGGTCAAGTCTGGTGCCAGCAGCCGCG------------------------------------------------------------------------------------------------------------------------------------------------------------------------------------------------------------------------------------------------------------------------------------------------------------------------------------------------------------------------------------------------------------------------------------------------------------------------------------------------------------------------------------------------------------------------------------------------------------------------------------------------------------------------------------------------------------------------------------------------------------------------------------------------------------------------------------------------------------------------------------------------------------------------------------------------------

Alignment Figure 5 :

>>ALP40-04_DE_Groe_Dmue_ADR-18_822pb_ALP40-04

CATGTGTAAGCGAAGCTATATGTGGAGCGGTGAAAGGCTCAGTAACGGGCGATTTATTTAATCTCCTGGGGCGGACAACATCGGGAAACTGATGGGAAAACGTCTAAGTTGCA------T---T----AAT-TTTAGTGTGACGTAAACGATTAT-CGTGCAGGAGAGTAAGATGCCATCCTATCAGTTAGTAAGTAGGGTAAGGGCCTACTTAGACGAAGACGGGTACGGGGAATGAGGGTTTGATTCCGGAGAGGGAGCCTGAGAGATGGCTACCAGGACCAAGGTCAGCAGCAGGCGCGAAAATTATCGAAGCCCGCC-TAGGGGCGATAGTGAGGAGACGTGTATAACGAAGTACGTGTAAAGAACGTACTAATAACTGGAGGTCAAGTCTGGTGCCAGCATCCGCGGTAATTCCAGCTCCAGGGGTGTCTATGATGATTGCTGCGATTAAAAAGTCCGTAGTCAAGCTGCCTGACTGACCTGCAATG-TGATTGATTAAGGAACGAGCAGGGTTAGGAAAGCAGAGAATTAGGAGCGACCGAGGGCTAGAGTATTGAATGGCGAGAGGTGAAATTTGATGACCCATTCAGGAGTGACAAAGGCGAAGGCACTAGTCAAGGGCGAATCCGATGATCAAGGACGTAGGCTAGAGTTTCGAAAACGATTAGAGACCGGAGTAGTTCTAGCAGTAAACTATGCCGACGCCGTGGTATGGTTTTTTGTGGCTGTATTGCGGAAGAGAAATCAAGTAAGGCTTTGGGGAGAGTACGCGCGCAAGCGATAAATTTAAAGGAAATTGACGGAGGAACACCACAAGGAGTGGAGTGTGCGGCT----------------------------------------------------------------------------------------------------------------------------------------------------------------------------------------------------------------------------------------------------------------------------------------------------------------------------------------------------------------------------------------------------------------------------------------------------------------------------------------------------------------------

>>M16_GvarMB5_BY_Gvar_Dmue_KAR-18_1760pb_MG773238

-------------AGCTATATGTGGAGCGGTGAAAGGCTCAGTAACGGGCGATTTATTTAATCTCCTGGGGCGGACAACATCGGGAAACTGATGGGAAAACGTCTAAGTTGCA------T---T----AAT-TTTAGTGTGACGTAAACGATTAT-CGTGCAGGAGAGTAAGATGCCATCCTATCAGTTAGTAAGTAGGGTAAGGGCCTACTTAGACGAAGACGGGTACGGGGAATGAGGGTTTGATTCCGGAGAGGGAGCCTGAGAGATGGCTACCAGGACCAAGGTCAGCAGCAGGCGCGAAAATTATCGAAGCCCGCC-TAGGGGCGATAGTGAGGAGACGTGTATAACGAAGTACGTGTAAAGAACGTACTAATAACTGGAGGTCAAGTCTGGTGCCAGCATCCGCGGTAATTCCAGCTCCAGGGGTGTCTATGATGATTGCTGCGATTAAAAAGTCCGTAGTCAAGCTGCCTGACTGACCTGCAATG-TGATTGATTAAGGAACGAGCAGGGTTAGGAAAGCAGAGAATTAGGAGCGACCGAGGGCTAGAGTATTGAATGGCGAGAGGTGAAATTTGATGACCCATTCAGGAGTGACAAAGGCGAAGGCACTAGTCAAGGGCGAATCCGATGATCAAGGACGTAGGCTAGAGTTTCGAAAACGATTAGAGACCGGAGTAGTTCTAGCAGTAAAATATGCCGACGCCGTGGTATGGTTTTTTGTGGCTGTATTGCGGAAGAGAAATCAAGTAAGGCTTTGGGGAGAGTACGCGCGCAAGCGATAAATTTAAAGGAAATTGACGGAGGAACACCACAAGGAGTGGAGTGTGCGGCTTAATTTGACTCAACGCGGGACAGCTTACCAGGCCCGATAATCGAGCGAGCGTTGTACGCGATAGATTAAAGAGTGGTGCATGGCTGCTATCGACAGTTGGGGTGACC-TTAGGGTTAATTCCGGCAAGTAGTGAGACCCCTGCAGATAG--TGGACAGGTA-TTTTT-AAAATACAGGAAGGAAGGGACAAGAGCAGGTCAGTGATGCCCTTAGATGGCCTGGGCTGCACGCGCACTACAGTGGTCATTATAAGTAGAAGTTAGA-AGTAAAGATGATCGAGAGGGACTGAGCTTTGTAAGAGGCTCACGAACGAGGAATTGCTAGTAATCGTAGGCTCATTAAGATACGATGAATATGTCCCTGTACCTTGTACACACCGCCCGTCGTTATCGAAGATGGAATTGTGTGCGAACGAGCAACAAGCGAGTGAGCGCATAGTTCTAGATGTGATAAAAGTCGTAACAAGGCAACTGTAGGAGAACCTGCAGTTGGATCATACA

>>M18_GvarM48_HU_Gvar_Dmue_KAR-18_1759pb_MG773240

-------------AGCTATATGTGGAGCGGTGAAAGGCTCAGTAACGGGCGATTTATTTAATCTCCTGGGACGGACAACATCGGGAAACTGATGGGAAAACGTCTAAGTTGCA------T---T----AAT-GTTAGTGTGACGTAAACGATATC--GTGCAGGAGAGTAAGATGCCATCCTATCAGTTAGTAAGTAGGGTAAGGGCCTACTTAGACGAAGACGGGTACGGGGAATGAGGGTTTGATTCCGGAGAGGGAGCCTGAGAGATGGCTACCAGGACCAAGGTCAGCAGCAGGCGCGAAAATTATCGAAGCCCGCC-TAGGGGCGATAGTGAGGAGACGTGTATAACGAAGTACGTGTAAAGAACGTACTAATAACTGGAGGTCAAGTCTGGTGCCAGCATCCGCGGTAATTCCAGCTCCAGGGGTGTCTATGATGATTGCTGCGATTAAAAAGTCCGTAGTCAAGCTGACTGACTGACCTGCAATG-TGATTGATTAAGGAACGAGCAGGGTTAGGAAAGCAGAGAATTAGGAGCGACCGAGGGCTAGAGTATTGAATGGCGAGAGGTGAAATTTGATGACCCATTCAGGAGTGACAAAGGCGAAGGCACTAGTCAAGGGCGAATCCGATGATCAAGGACGTAGGCTAGAGTTTCGAAAACGATTAGAGACCGGAGTAGTTCTAGCAGTAAACTATGCCGACGCCGTGATATAGTTTTTTGTGGCTGTATTGCGGAAGAGAAATCAAGTAAGGCTTTGGGGAGAGTACGCGCGCAAGCGATAAATTTAAAGGAAATTGACGGAGGAACACCACAAGGAGTGGAGTGTGCGGCTTAATTTGACTCAACGCGGGACAGCTTACCAGGCCCGATAATCGAGCGAGCGTTGTACGCGATAGATTAAAGAGTGGTGCATGGCTGCTATCGACAGTTGGGGTGACCTTTAGGGTTAATTCCGGCAAGTAGTGAGACCCCTGCAGATAG--TGGACAGGTA-TTTTT-AAAATACAGGAAGGAAGGGACAAGAGCAGGTCAGTGATGCCCTTAGATGGCCTGGGCTGCACGCGCACTACAGTGGTCATTATAAGTAGAAGTTAGA-AGTAAAGATGATCGAGAGGGACTGAGCTTTGTAAGAGGCTCACGAACGAGGAATTGCTAGTAATCGTAGGCTCATTAAGATACGATGAATATGTCCCTGTACCTTGTACACACCGCCCGTCGTTATCGAAGATGGAATTGTGTGCGAACGAGCAACAAGCGAGTGAGCGCATAGTTCTAGATGTGATAAAAGTCGTAACAAGGCAACTGTAGGAGAACCTGTAGTTGGATCATACA

>>LOQ-05_FR_Groe_Droe-5_ADR-18_826pb_LOQ-05

CATGTGTAAGCGAAGCGATAAGTGGAGCGGTGAAAGGCTCAGTAACGGGCGATTTATTTGATCTCCTGGGACGGACAACATCGGGAAACTGATGGGAAAACGTCTAAGTTGCA------GTTTT----TTT-TTGATTGCGACGTAAACCTTTTG--GTGCAGGAGAGTAAGATGCCATCCTATCAGTTAGTAAGTAGGGTAAGGGCCTACTTAGACGAAGACGGGTACGGGGAATGAGGGTTTGATTCCGGAGAGGGAGCCTGAGAGATGGCTACCAGGACCAAGGTCAGCAGCAGGCGCGAAAATTATCGAAGCCCGCC-TAGGGGCGATAGTGAGGAGACGTGTATAACGAAGTACGTGTAAAGACCGTACTAATAACTGGAGGTCAAGTCTGGTGCCAGCATCCGCGGTAATTCCAGCTCCAGGGGTGTCTATGATGATTGCTGCGATTAAAAAGTCCGTAGTCGAGCTGACTGACTGACCTGCAATG-TGGTTGATTAAAAGACGAGCAGGGTTAGGAAAGCAGAGAATTAGGAGCGACCGAGGGCTAGAGTATTGAATGGCGAGAGGTGAAATTTGATGACCCATTCAGGAGTGACAAAGGCGAAGGCACTAGTCAAGGGCGAATCCGATGATCAAGGACGTAGGCTAGAGTTTCGAAAACGATTAGAGACCGGAGTAGTTCTAGCAGTAAACTATGCCGACACCGTGGTATTAATTTTT----TAGTATTGCGGAAGAGAAATCAAGTAAGGCTTTGGGGAGAGTACGCGCGCAAGCGATAAATTTAAAGGAAATTGACGGAGGAACACCACAAGGAGTGGAGTGTGCGGCTTAATTT----------------------------------------------------------------------------------------------------------------------------------------------------------------------------------------------------------------------------------------------------------------------------------------------------------------------------------------------------------------------------------------------------------------------------------------------------------------------------------------------------------------

>>LOQ-08_FR_Groe_Droe-4_ADR-18_801pb_LOQ-08

CATGTGTAAGCGAAGCGATAAGTGGAGCGGTGAAAGGCTCAGTAACGGGCGATTTATTTGATCTCCTGGGACGGACAACATCGGGAAACTGATGGGAAAACGTCTAAGTTGCA------GTTTT----TTT-TTGATTGCGACGTAAACCTTTTG--GTGCAGGAGAGTAAGATGCCATCCTATCAGTTAGTAAGTAGGGTAAGGGCCTACTTAGACGAAGACGGGTACGGGGAATGAGGGTTTGATTCCGGAGAGGGAGCCTGAGAGATGGCTACCAGGACCAAGGTCAGCAGCAGGCGCGAAAATTATCGAAGCCCGCC-TAGGGGCGATAGTGAGGAGACGTGTATAACGAAGTACGTGTAAAGACCGTACTAATAACTGGAGGTCAAGTCTGGTGCCAGCAGCCGCGGTAATTCCAGCTCCAGGGGTGTCTATGATGATTGCTGCGATTAAAAAGTCCGTAGTCGAGCTGACTGACTGACCTGCAATG-TGGTTGATTAAAAGACGAGCAGGGTTAGGAAAGCAGAGAATTAGGAGCGACCGAGGGCTAGAGTATTGAATGGCGAGAGGTGAAATTTGATGACCCATTCAGGAGTGACAAAGGCGAAGGCACTAGTCAAGGGCGAATCCGATGATCAAGGACGTAGGCTAGAGTTTCGAAAACGATTAGAGACCGGAGTAGTTCTAGCAGTAAACTATGCCGACACCGTGGTATTAATTTTT----TAGTATTGCGGAAGAGAAATCAAGTAAGGCTTTGGGGAGAGTACGCGCGCAAGCGATAAATTTAAAGGAAATTGACGGAGGAACACCACA-----------------------------------------------------------------------------------------------------------------------------------------------------------------------------------------------------------------------------------------------------------------------------------------------------------------------------------------------------------------------------------------------------------------------------------------------------------------------------------------------------------------------------------------

>>LOQ-07_FR_Groe_Droe-1_ADR-18_818pb_LOQ-07

CATGTGTAAGCGAAGCGATAAGTGGAGCGGTGAAAGGCTCAGTAACGGGCGATTTATTTGATCTCCTGGGACGGACAACATCGGGAAACTGATGGGAAAACGTCTAAGTTGCA------GTTTT----TTT-TTGATTGCGACGTAAACCTTTTG--GTGCAGGAGAGTAAGATGCCCTCCTATCAGTTAGTAAGTAGGGTAGGGGCCTACTTAGACGAAGACGGGTACGGGGAATGAGGGTTTGATTCCGGAGAGGGAGCCTGAGAGATGGCTACCAGGACCAAGGTCAGCAGCAGGCGCGAAAATTATCGAACCCCGCC-TAGGGGCGATAGTGAGGAGACGTGTATAACGAAGTACGTGTAAAGACCGTACTAATAACTGGAGGTCAAGTCTGGTGCCAGCATCCGCGGTAATTCCAGCTCCAGGGGTGTCTATGATGATTGCTGCGATTAAAAAGTCCGTAGTCGAGCTGACTGACTGACCTGCAATG-TGGTTGATTAAAAGACGAGCAGGGTTAGGAAAGCAGAGAATTAGGAGCGACCGAGGGCTAGAGTATTGAATGGCGAGAGGTGAAATTTGATGACCCATTCAGGAGTGACAAAGGCGAAGGCACTAGTCAAGGGCGAATCCGATGATCAAGGACGTAGGCTAGAGTTTCGAAAACGATTAGAGACCGGAGTAGTTCTAGCAGTAAACTATGCCGACACCGTGGTATTAATTTTT----TAGTATTGCGGAAGAGAAATCAAGTAAGGCTTTGGGGAGAGTACGCGCGCAAGCGATAAATTTAAAGGAAATTGACGGAGGAACACCACAAGGAGTGGAGTGTGCGG------------------------------------------------------------------------------------------------------------------------------------------------------------------------------------------------------------------------------------------------------------------------------------------------------------------------------------------------------------------------------------------------------------------------------------------------------------------------------------------------------------------------

>>VOL5.4-07_FR_Groe_Droe-2_ADR-18_818pb_VOL5.4-07

CATGTGTAAGCGAAGCGATAAGTGGAGCGGTGAAAGGCTCAGTAACGGGCGATTTATTTGATCTCCTGGGACGGACAACATCGGGAAACTGATGGGAAAACGTCTAAGTTGCA------GTTTT----TTT-TTGATTGCGACGTAAACCTTTTG--GTGCAGGAGAGTAAGATGCCCTCCTATCAGTTAGTAAGTAGGGTAAGGGCCTACTTAGACGAAGACGGGTACGGGGAATGAGGGTTTGATTCCGGAGAGGGAGCCTGAGAGATGGCTACCAGGACCAAGGTCAGCAGCAGGCGCGAAAATTATCGAACCCCGCC-TAGGGGCGATAGTGAGGAGACGTGTATAACGAAGTACGTGTAAAGACCGTACTAATAACTGGAGGTCAAGTCTGGTGCCAGCATCCGCGGTAATTCCAGCTCCAGGGGTGTCTATGATGATTGCTGCGATTAAAAAGTCCGTAGTCGAGCTGACTGACTGACCTGCAATG-TGGTTGATTAAAAGACGAGCAGGGTTAGGAAAGCAGAGAATTAGGAGCGACCGAGGGCTAGAGTATTGAATGGCGAGAGGTGAAATTTGATGACCCATTCAGGAGTGACAAAGGCGAAGGCACTAGTCAAGGGCGAATCCGATGATCAAGGACGTAGGCTAGAGTTTCGAAAACGATTAGAGACCGGAGTAGTTCTAGCAGTAAACTATGCCGACACCGTGGTATTAATTTTT----TAGTATTGCGGAAGAGAAATCAAGTAAGGCTTTGGGGAGAGTACGCGCGCAAGCGATAAATTTAAAGGAAATTGACGGAGGAACACCACAAGGAGTGGAGTGTGCGG------------------------------------------------------------------------------------------------------------------------------------------------------------------------------------------------------------------------------------------------------------------------------------------------------------------------------------------------------------------------------------------------------------------------------------------------------------------------------------------------------------------------

>>MAR6-4-01_FR_Groe_Droe-3_ADR-18_823pb_MAR6-4-01

CATGTGTAAGCGAAGCGATAAGTGGAGCGGTGAAAGGCTCAGTAACGGGCGATTTATTTGATCTCCTGGGACGGACAACATCGGGAAACTGATGGGAAAACGTCTAAGTTGCA------GTTTT----TTT-TTGATTGCGACGTAAACCTTTTG--GTGCAGGAGAGTAAGATGCCATCCTATCAGTTAGTAAGTAGGGTAGGGGCCTACTTAGACGAAGACGGGTACGGGGAATGAGGGTTTGATTCCGGAGAGGGAGCCTGAGAGATGGCTACCAGGACCAAGGTCAGCAGCAGGCGCGAAAATTATCGAACCCCGCC-TAGGGGCGATAGTGAGGAGACGTGTATAACGAAGTACGTGTAAAGACCGTACTAATAACTGGAGGTCAAGTCTGGTGCCAGCATCCGCGGTAATTCCAGCTCCAGGGGTGTCTATGATGATTGCTGCGATTAAAAAGTCCGTAGTCGAGCTGACTGACTGACCTGCAATG-TGGTTGATTAAAAGACGAGCAGGGTTAGGAAAGCAGAGAATTAGGAGCGACCGAGGGCTAGAGTATTGAATGGCGAGAGGTGAAATTTGATGACCCATTCAGGAGTGACAAAGGCGAAGGCACTAGTCAAGGGCGAATCCGATGATCAAGGACGTAGGCTAGAGTTTCGAAAACGATTAGAGACCGGAGTAGTTCTAGCAGTAAACTATGCCGACACCGTGGTATTAATTTTT----TAGTATTGCGGAAGAGAAATCAAGTAAGGCTTTGGGGAGAGTACGCGCGCAAGCGATAAATTTAAAGGAAATTGACGGAGGAACACCACAAGGAGTGGAGTGTGCGGCTTAA-------------------------------------------------------------------------------------------------------------------------------------------------------------------------------------------------------------------------------------------------------------------------------------------------------------------------------------------------------------------------------------------------------------------------------------------------------------------------------------------------------------------

>>KR871360_DE_Groe_Ddue_GRA-15_548pb_KR871360

-----------------------------------------------------------------------------------------------------------------------------------------------------------------------GTAAGATGCCATCCTATCAGTTAGTAAGTAGGGTAAGGGCCTACTTAGACGAAGACGGGTACGGGGAATGAGGGTTTGATTCCGGAGAGGGAGCCTGAGAGATGGCTACCAGGACCAAGGTCAGCAGCAGGCGCGAAAATTATCGAAGCCCGCC-TAGGGGCGATAGTGAGGAGACGTGTATAACGAAGTACGTGTAAAGACCGTACTAATAACTGGAGGTCAAGTCTGGTGCCAGCATCCGCGGTAATTCCAGCTCCAGGGGTGTCTATGATGATTGCTGCGATTAAAAAGTCCGTAGTCGAGCTGACTGACTGACCTGCAGTG-TGGTTGATTAAAAGACGAGCAGGGTTAGGAAAGCAGAGAATTAAGAGCGACCGAGGGCTAGAGTATTGAATGGCGAGAGGTGAAATTTGATGACCCATTCAAGAGTGACAAAGGCGAAGGCACTAGTCAAGGGCGAATCCGATGATCAAGGACGTACGCTAGAGTTTCGAAAACGATTAGAGACCGGAGTAGTTCTAGCAGTAAACTATGCCGACACCGTGGTA------------------------------------------------------------------------------------------------------------------------------------------------------------------------------------------------------------------------------------------------------------------------------------------------------------------------------------------------------------------------------------------------------------------------------------------------------------------------------------------------------------------------------------------------------------------------------------------------------------------------------------------------

>>KR871359_DE_Gpul_Ddue_GRA-15_1054pb_KR871359

---------------------GTGGAGCGGTGAAAGGCTCAGTAACGGGCGATTTATTTGATCTCCTGGGACGGACAACATCGGGAAACTGATGGGAAAACGTCTAAGTTGCA------G---T----TTA-TTGATTGTGACGTAAACCTATGT--GTGCAGGAGAGTAAGATGCCATCCTATCAGTTAGTAAGTAGGGTAAGGGCCTACTTAGACGAAGACGGGTACGGGGAATGAGGGTTTGATTCCGGAGAGGGAGCCTGAGAGATGGCTACCAGGACCAAGGTCAGCAGCAGGCGCGAAAATTATCGAAGCCCGCC-CAGGGGCGATAGTGAGGAGACGTGTATAACGAAGTACGTGTAAAGACCGTACTAATAACTGGAGGTCAAGTCTGGTGCCAGCATCCGCGGTAATTCCAGCTCCAGGGGTGTCTATGATGATTGCTGCGATTAAAAAGTCCGTAGTCGAGCTGACTGACTGACCTGCAATG-TGATTGATTAAAAGACGAGCAGGGTTAGGAAAGCAGAGAATTAGGAGCGACCGAGGGCTAGAGTATTGAATGGCGAGAGGTGAAATTTGATGACCCATTCAGGAGTGACAAAGGCGAAGGCACTAGTCAAGGGCGAATCCGATGATCAAGGACGTAGGCTAGAGTTTCGAAAACGATTAGAGACCGGAGTAGTTCTAGCAGTAAACTATGCCGACACCGTGGTATTAAATTT-----TAGTATTGCGGAAGAGAAATCAAGTAAGGCTTTGGGGAGAGTACGCGCGCAAGCGATAAATTTAAAGGAAATTGACGGAGGAACACCACAAGGAGTGGAGTGTGCGGCTTAATTTGACTCAACGCGGGACAGCTTACCAGGCCCGATAATCGAGCGAGCGTAGTACGCGATAGGTTAAAGAGTGGTGCATGGCTGCTATCGACAGTTGGGGTGACC-TTAGGGTTAATTCCGGCAAGTAGTGAGACCCCTGCAGATAG--TGGACAGGTA-TTTTT-AAGATACAGGAAGGAAGGGACAAGAGCAGGTCAGTGATGCCCTTAGATGGCCTGGGCTGCACGCGCACTACAGTGGTCATTATAAGTAGAAAGTAG----------------------------------------------------------------------------------------------------------------------------------------------------------------------------------------------------------------------------------------------

>>R5_GRR24_FR_Groe_Droe_KAR-18_1759pb_MG773219*

-------------AGCGATAAGTGGAGCGGTGAAAGGCTCAGTAACGGGCGATTTATTTGATCTCCTGGGACGGACAACATCGGGAAACTGATGGGAAAACGTCTAAGTTGCA------GTTTT----TTT-TTGATTGCGACGTAAACCTTTTG--GTGCAGGAGAGTAAGATGCCATCCTATCAGTTAGTAAGTAGGGTAAGGGCCTACTTAGACGAAGACGGGTACGGGGAATGAGGGTTTGATTCCGGAGAGGGAGCCTGAGAGATGGCTACCAGGACCAAGGTCAGCAGCAGGCGCGAAAATTATCGAAGCCCGCC-TAGGGGCGATAGTGAGGAGACGTGTAT-ACGAAGTACGTGTAAAGACCGTACTAATAACTGGAGGTCAAGTCTGGTGCCAGCATCCGCGGTAATTCCAGCTCCAGGGGTGTCTATGATGATTGCTGCGATTAAAAAGTCCGTAGTCGAGCTGACTGACTGACCTGCAATG-TGGTTGATTAAAAGACGAGCAGGGTTAGGAAAGCAGAGAATTAGGAGCGACCGAGGGCTAGAGTATTGAATGGCGAGAGGTGAAATTTGATGACCCATTCAGGAGTGACAAAGGCGAAGGCACTAGTCAAGGGCGAATCCGATGATCAAGGACGTAGGCTAGAGTTTCGAAAACGATTAGAGACCGGAGTAGTTCTAGCAGTAAACTATGCCGACACCGTGGTATTAATTTTT----TAGTATTGCGGAAGAGAAATCAAGTAAGGCTTTGGGGAGAGTACGCGCGCAAGCGATAAATTTAAAGGAAATTGACGGAGGAACACCACAAGGAGTGGAGTGTGCGGCTTAATTTGACTCAACGCGGGACAGCTTACCAGGCCCGATAATCGAGCGAGCGTAGTACGCGATAGATTAAAAAGTGGTGCATGGCTGCTATCGACAGTTGGGGTGACC-TTAGGGTTAATTCCGGCAAGTAGTGAGACCCCTGCAGATAG--TGGACAGGTA-TTTTT-AAGATACAGGAAGGAAGGGACAAGAGCAGGTCAGTGATGCCCTTAGATGGCCTGGGCTGCACGCGCACTACAGTGGTCATTATAAGTAGAAATTAGA-TATAAAGATGATCGAGAGGGACTGAGCTTTGTAAGAGGCTCACGAACGAGGAATTGCTAGTAATCGTAGGCTCATTAAGATACGATGAATATGTCCCTGTACCTTGTACACACCGCCCGTCGTTATCGAAGATGGAATTGTGTGCGAACGAGCATTAAGCGAGTGAGCGCATAGTTCTAGATGTGATAAAAGTCGTAACAAGGCAACTGTAGGAGAACCTGTAGTTGGATCATACA

>>R6_GbOv2_PL_Gbal_Droe_KAR-18_1755pb_MG773220

-------------AGCGATAAGTGGAGCGGTGAAAGGCTCAGTAACGGGCGATTTATTTGATCTCCTGGGACGGACAACATCGGGAAACTGATGGGAAAACGTCTAAGTTGCA------G---T----TGT-TTTATTGTGACGTAAACCTTTGT--GTGCAGGAGAGTAAGATGCCATCCTATCAGTTAGTAAGTAGGGTAAGGGCCTACTTAGACGAAGACGGGTACGGGGAATGAGGGTTTGATTCCGGAGAGGGAGCCTGAGAGATGGCTACCAGGACCAAGGTCAGCAGCAGGCGCGAAAATTATCGAAGCCCGCC-TAGGGGCGATAGTGAGGAGACGTGTAT-ACGAAGTACGTGTAAAGACCGTACTAATAACTGGAGGTCAAGTCTGGTGCCAGCATCCGCGGTAATTCCAGCTCCAGGGGTGTCTATGATGATTGCTGCGATTAAAAAGTCCGTAGTCGAGCTGACTGACTGACCTGCAATG-TGATTGATTAAAGAACGAGCAGGGTTAGGAAAGCAGAGGATTAGGAGCGACCGAGGGCTAGAGTATTGAATGGCGAGAGGTGAAATTTGATGACCCATTCAGGAGTGACAAAGGCGAAGGCACTAGTCAAGGGCGAATCCGATGATCAAGGACGTAGGCTAGAGTTTCGAAAACGATTAGAGACCGGAGTAGTTCTAGCAGTAAACTATGCCGACACCGTGGTATTAATTT------TAGTATTGCGGAAGAGAAATCAAGTAAGGCTTTGGGGAGAGTACGCGCGCAAGCGATAAATTTAAAGGAAATTGACGGAGGAACACCACAAGGAGTGGAGTGTGCGGCTTAATTTGACTCAACGCGGGACAGCTTACCAGGCCCGATAATCGAGCGAGCGTAGTACGCGATAGGTTAAAAAGTGGTGCATGGCTGCTATCGACAGTTGGGGTGACC-TTAGGGTTAATTCCGGCAAGTAGTGAGACCCCTGCAGATAG--TGGACAGGTA-TTTTT-AAGATACAGGAAGGAAGGGACAAGAGCAGGTCAGTGATGCCCTTAGATGGCCTGGGCTGCACGCGCACTACAGTGGTCATTATAAGTAGAAGTTAGA-TATAAAGATGATCGAGAGGGACTGAGCTTTGTAAGAGGCTCACGAACGAGGAATTGCTAGTAATCGTAGGCTCATTAAGATACGATGAATATGTCCCTGTACCTTGTACACACCGCCCGTCGTTATCGAAGATGGAATTGTGTGCGAACGAGCATTAAGCGAGTGAGCGCATAGTTCTAGATGTGATAAAAGTCGTAACAAGGCAACTGTAGGAGAACCTGTAGTTGGATCATACA

>>KR871355_DE_Gfos_Ddue_GRA-15_1090pb_KR871355

---------GC-GAGCTTTTAGTGGAGCGGTGAAAGGCTCAGTAACGGGCGATTTATTTGTTCTCCTGTGACGGACAACATCGGGAAACTGATGGGAAAACGTCTAAGTTGCA------G---T----TAA-TTGATTGTGACGTAAACGATATT--GTGCAGGAGAGTAAGATGCCATCCTATCAGTTAGTAAGTAGGGTAAGGGCCTACTTAGACGAAGACGGGTACGGGGAATGAGGGTTTGATTCCGGAGAGGGAGCCTGAGAGATGGCTACCAGGACCAAGGTCAGCAGCAGGCGCGAAAATTATCGAAGCCCGCC-TAGGGGCGATAGTGAGGAGACGTGTATAACGAAGTACGTGTAAAGAACGTGCTAATAACTGGAGGTCAAGTCTGGTGCCAGCATCCGCGGTAATTCCAGCTCCAGGGGTGTCTATGATGATTGCTGCGATTAAAAAGTCCGTAGTCAATCTGACTGACTGGCCTGCAATG-TGATTGATCAAAGAACGAGCAGGGTTAGGAAAGCAGAGAATCAGGAGCGACCGAGGGCTAGAGTATTGAATGGCGAGAGGTGAAATTTGATGACCCATTCAGGAGTGACAAAGGCGAAGGCACTAGTCAAGGGCGAATCCGATGATCAAGGACGTAGGCTAGAGTTTCGAAAACGATTAGAGACCGGAGTATTTCTAGCAGTAAACTATGCCGACGCCGTGGTATCGATT-------TGGTATTGCGGAAGAGAAATCAAGTAAGGCTTTGGGGAGAGTACGCGCGCAAGCGATAAATTTAAAGGAAATTGACGGAGGAACACCACAAGGAGTGGAGTGTGCGGCTTAATTTGACTCAACGCGGGACAGCTTACCAGGCCCGATAATCGAGCGAGCGTAGTACGCGATAGGTTAAAGAGTGGTGCATGGCTGCTATCGACAGTTGGGGTGACC-TTAGGGTTAATTCCGGCAAGTAGTGAGACCCCTGCAGATAG--TAGACAGGTA-TTTTT-AAAATACAGGAAGGAAGGGACAAGAGCAGGTCAGTGATGCCCTTAGATGGCCTGGGCTGCACGCGCACTACAGTGGTCATTATAAGTAGAAGTTAGA-TATAAAGATGATCGAAAGGGACTGAA------------------------------------------------------------------------------------------------------------------------------------------------------------------------------------------------------------------

>>KR871356_DE_Gpul_Ddue_GRA-15_1052pb_KR871356

---------------------GTGGAGCGGTGAAAGGCTCAGTAACGGGCGATTTATTTGTTCTCCTGTGACGGACAACATCGGGAAACTGATGGGAAAACGTCTAAGTTGCA------G---T----TAA-TTGATTGTGACGTAAACGATATT--GTGCAGGAGAGTAAGATGCCATCCTATCAGTTAGTAAGTAGGGTAAGGGCCTACTTAGACGAAGACGGGTACGGGGAATGAGGGTTTGATTCCGGAGAGGGAGCCTGAGAGATGGCTACCAGGACCAAGGTCAGCAGCAGGCGCGAAAATTATCGAAGCCCGCC-TAGGGGCGATAGTGAGGAGACGTGTATAACGAAGTACGTGTAAAGAACGTGCTAATAACTGGAGGTCAAGTCTGGTGCCAGCATCCGCGGTAATTCCAGCTCCAGGGGTGTCTATGATGATTGCTGCGATTAAAAAGTCCGTAGTCAATCTGACTGACTGGCCTGCAATG-TGATTGATTAAAGAACGAGCAGGGTTAGGAAAGCAGAGAATCAGGAGCGACCGAGGGCTAGAGTATTGAATGGCGAGAGGTGAAATTTGATGACCCATTCAGGAGTGACAAAGGCGAAGGCACTAGTCAAGGGCGAATCCGATGATCAAGGACGTAGGCTAGAGTTTCGAAAACGATTAGAGACCGGAGTAGTTCTAGCAGTAAACTATGCCGACGCCGTGGTATCATTT-------TGGTATTGCGGAAGAGAAATCAAGTAAGGCTTTGGGGAGAGTACGCGCGCAAGCGATAAATTTAAAGGAAATTGACGGAGGAACACCACAAGGAGTGGAGTGTGCGGCTTAATTTGACTCAACGCGGGACAGCTTACCAGGCCCGATAATCGAGCGAGCGTAGTACGCGATAGGTTAAAGAGTGGTGCATGGCTGCTATCGACAGTTGGGGTGACC-TTAGGGTTAATTCCGGCAAGTAGTGAGACCCCTGCAGATAG--TGGACAGGTA-TTTTT-AAAATACAGGAAGGAAGGGACAAGAGCAGGTCAGTGATGCCCTTAGATGGCCTGGGCTGCACGCGCACTACAGTGGTCATTATAAGTAGAAGTTAG----------------------------------------------------------------------------------------------------------------------------------------------------------------------------------------------------------------------------------------------

>>KP091740_GB_Gpul_Ddue_IRO-15_1280pb_KP091740

CATGTGTAAGCGAAGCTTTTAGTGGAGCGGTGAAAGGCTCAGTAACGGGCGATTTATTTGTTCTCCTGTGACGGACAACATCGGGAAACTGATGGGAAAACGTCTAAGTTGCA------G---T----TAA-TTGATTGTGACGTAAACGATATT--GTGCAGGAGAGTAAGATGCCATCCTATCAGTTAGTAAGTAGGGTAAGGGCCTACTTAGACGAAGACGGGTACGGGGAATGAGGGTTTGATTCCGGAGAGGGAGCCTGAGAGATGGCTACCAGGACCAAGGTCAGCAGCAGGCGCGAAAATTATCGAAGCCCGCC-TAGGGGCGATAGTGAGGAGACGTGTATAACGAAGTACGTGTAAAGAACGTGCTAATAACTGGAGGTCAAGTCTGGTGCCAGCATCCGCGGTAATTCCAGCTCCAGGGGTGTCTATGATGATTGCTGCGATTAAAAAGTCCGTAGTCAATCTGACTGACTGGCGTGCAATG-TGATTGATTAAAGAACGAGCAGGGTTAGGAAAGCAGAGAATCAGGAGCGACCGAGGGCTAGAGTATTGAATGGCGAGAGGTGAAATTTGATGACCCATTCAGGAGTGACAAAGGCGAAGGCACTAGTCAAGGGCGAATCCGATGATCAAGGACGTAGGCTAGAGTTTCGAAAACGATTAGAGACCGGAGTAGTTCTAGCAGTAAACTATGCCGACGCCGTGGTATCGATT-------TGGTATTGCGGAAGAGAAATCAAGTAAGGCTTTGGGGAGAGTACGCGCGCAAGCGATAAATTTAAAGGAAATTGACGGAGGAACACCACAAGGAGTGGAGTGTGCGGCTTAATTTGACTCAACGCGGGACAGCTTACCAGGCCCGATAATCGAGCGAGCGTAGTACGCGATAGGTTAAAGAGTGGTGCATGGCTGCTATCGACAGTTGGGGTGACC-TTAGGGTTAATTCCGGCAAGTAGTGAGACCCCTGCAGATAG--TGGACAGGTA-TTTTT-AAAATACAGGAAGGAAGGGACAAGAGCAGGTCAGTGATGCCCTTAGATGGCCTGGGCTGCACGCGCACTACAGTGGTCATTATAAGTAGAAGTTAGA-TATAAAGATGATCGAGAGGGACTGAGCTTTGTAAGAGGCTCACGAACGAGGAATTGCTAGTAATCGTAGGCTCATTAAGATACGATGAATATGTCCCTGTACCTTGTACACACCGCCCGTCGTTATCGAAGATGGAATTGTGTGCGAACGAGCAA---------------------------------------------------------------------------------

>>KR871357_DE_Eber_Ddue_GRA-15_1053pb_KR871357

---------------------GTGGAGCGGTATACGGCTCAGTAACGGGCGATTTATTTGTTCTCCTGGGACGGACAACATCGGGAAACTGATGGGAAAACGTCTAAGTTGCA------G---T----TAA-TTGACTGTGACGTAAACCTTAGT--GTGCAGGAGAGTAAGATGCCATCCTATCAGTTAGTAAGTAGGGTAAGGGCCTACTTAGACGAAGACGGGTACGGGGAATGAGGGTTTGATTCCGGAGAGGGAGCCTGAGAGATGGCTACCAGGACCAAGGTCAGCAGCAGGCGCGAAAATTATCGAAGCCCGCC-TAGGGGCGATAGTGAGGAGACGTGTATAACGAAGTACGTGTAAAGAACGTGCTAATAACTGGAGGTCAAGTCTGGTGCCAGCATCCGCGGTAATTCCAGCTCCAGGGGTGTCTATGATGATTGCTGCGATTAAAAAGTCCGTAGTCAATCTGACTGACTGACCTGCAATG-TGATTGATTAAAGAACGAGCAGGGTTAGGAAAGCAGAGAATTAGGAGCGACCGAGGGCTAGAGTATTGAATGGCGAGAGGTGAAATTTGATGACCCATTCAGGAGTGACAAAGGCGAAGGCACTAGTCAAGGGCGAATCCGATGATCAAGGACGTAGGCTAGAGTTTCGAAAACGATTAGAGACCGGAGTAGTTCTAGCAGTAAACTATGCCGACGCCGTGGTATCAACAA------TGGTATTGCGGAAGAGAAATCAAGTAAGGCTTTGGGGAGAGTACGCGCGCAAGCGATAAATTTAAAGGAAATTGACGGAGGAACACCACAAGGAGTGGAGTGTGCGGCTTAATTTGACTCAACGCGGGACAGCTTACCAGGCCCGATAATCGAGCGAGCGTAGTACGCGATAGGTTAAAGAGTGGTGCATGGCTGCTATCGACAGTTGGGGTGACC-TTAGGGTTAATTCCGGCAAGTAGTGAGACCCCTGCAGATAG--TGGACAGGTA-TTTTT-AAAATACAGGAAGGAAGGGACAAGAGCAGGTCAGTGATGCCCTTAGATGGCCTGGGCTGCACGCGCACTACAGTGGTCATTATAAGTAGAAGTTAG----------------------------------------------------------------------------------------------------------------------------------------------------------------------------------------------------------------------------------------------

>>KR871358_DE_Groe_Ddue_GRA-15_1053pb_KR871358

---------------------GTGGAGCGGTATACGGCTCAGTAACGGGCGATTTATTTGTTCTCCTGGGACGGACAACATCGGGAAACTGATGGGAAAACGTCTAAGTTGCA------G---T----TAA-TTGACTGTGACGTAAACCTTTGT--GTGCAGGAGAGTAAGATGCCATCCTATCAGTTAGTAAGTAGGGTAAGGGCCTACTTAGACGAAGACGGGTACGGGGAATGAGGGTTTGATTCCGGAGAGGGAGCCTGAGAGATGGCTACCAGGACCAAGGTCAGCAGCAGGCGCGAAAATTATCGAAGCCCGCC-TAGGGGCGATAGTGAGGAGACGTGTATAACGAAGTACGTGTAAAGAACGTGCTAATAACTGGAGGTCAAGTCTGGTGCCAGCATCCGCGGTAATTCCAGCTCCAGGGGTGTCTATGATGATTGCTGCGATTAAAAAGTCCGTAGTCAATCTGACTGACTGACCTGCAATG-TGATTGATTAAAGAACGAGCAGGGTTAGGAAAGCAGAGAATTAGGAGCGACCGAGGGCTAGAGTATTGAATGGCGAGAGGTGAAATTTGATGACCCATTCAGGAGTGACAAAGGCGAAGGCACTAGTCAAGGGCGAATCCGATGATCAAGGACGTAGGCTAGAGTTTCGAAAACGATTAGAGACCGGAGTAGTTCTAGCAGTAAACTATGCCGACGCCGTGGTATCAACAA------TGGTATTGCGGAAGAGAAATCAAGTAAGGCTTTGGGGAGAGTACGCGCGCAAGCGATAAATTTAAAGGAAATTGACGGAGGAACACCACAAGGAGTGGAGTGTGCGGCTTAATTTGACTCAACGCGGGACAGCTTACCAGGCCCGATAATCGAGCGAGCGTAGTACGCGATAGGTTAAAGAGTGGTGCATGGCTGCTATCGACAGTTGGGGTGACC-TTAGGGTTAATTCCGGCAAGTAGTGAGACCCCTGCAGATAG--TGGACAGGTA-TTTTT-AAAATACAGGAAGGAAGGGACAAGAGCAGGTCAGTGATGCCCTTAGATGGCCTGGGCTGCACGCGCACTACAGTGGTCATTATAAGTAGAAGTTAG----------------------------------------------------------------------------------------------------------------------------------------------------------------------------------------------------------------------------------------------

>>D3_DEB2_DE_Eberi_Ddue_KAR-18_1753pb_MG773214

-------------AGCTTATAGTGGAGCGGTATACGGCTCAGTAACGGGCGATTTATTTGTTCTCCTGGGACGGACAACATCGGGAAACTGATGGGAAAACGTCTAAGTTGCA------G---T----TAA-TTGACTGTGACGTAAACCTTTGT--GTGCAGGAGAGTAAGATGCCATCCTATCAGTTAGTAAGTAGGGTAAGGGCCTACTTAGACGAAGACGGGTACGGGGAATGAGGGTTTGATTCCGGAGAGGGAGCCTGAGAGATGGCTACCAGGACCAAGGTCAGCAGCAGGCGCGAAAATTATCGAAGCCCGCC-TAGGGGCGATAGTGAGGAGACGTGTATAACGAAGTACGTGTAAAGAACGTGCTAATAACTGGAGGTCAAGTCTGGTGCCAGCATCCGCGGTAATTCCAGCTCCAGGGGTGTCTATGATGATTGCTGCGATTAAAAAGTCCGTAGTCAATCTGACTGACTGACCTGCAATG-TGATTGATTAAAGAACGAGCAGGGTTAGGAAAGCAAAGAATTAGGAGCGACCGAGGGCTAGAGTATTGAATGGCGAGAGGTGAAATTTGATGACCCATTCAGGAGTGACAAAGGCGAAGGCACTAGTCAAGGGCGAATCCGATGATCAAGGACGTAGGCTAAAGTTTCGAAAACGATTAGAGACCGGAGTAGTTCTAGCAGTAAACTATGCCGACGCCGTGGTATCAACAA------TGGTATTGCGGAAGAGAAATCAAGTAAGGCTTTGGGGAGAGTACGCGCGCAAGCGATAAATTTAAAGGAAATTGACGGAGGAACACCACAAGGAGTGGAGTGTGCGGCTTAATTTGACTCAACGCGGGACAGCTTACCAGGCCCGATAATCGAGCGAGCGTAGTACGCGATAGGTTAAAGAGTGGTGCATGGCTGCTATCGACAGTTGGGGTGACC-TTAGGGTTAATTCCGGCAAGTAGTGAGACCCCTGCAGATAG--TGGACAGGTA-TTTTT-AAAATACAGGAAGGAAGGGACAAGAGCAGGTCAGTGATGCCCTTAGATGGCCTGGGCTGCACGCGCACTACAGTGGTCATTATAAGTAGAAGTTAGA-TATAAAGATGATCGAGAGGGACTGAGCTTTGTAAGAGGCTCACGAACGAGGAATTGCTAGTAATCGTAGGCTCATTAAGATACGATGAATATGTCCCTGTACCTTGTACACACCGCCCGTCGTTATCGAAGATGGAATTGTGTGCGAACGAGCAACAAGCGAGTGAGCGCATAGTTCTAGATGTGATAAAAGTCGTAACAAGGCAACTGTAGGAGAACCTGTAGTTGGATCATACA

>>R1_GFOS_GfOv_PL_Gfos_Droe_KAR-18_1751pb_MG773215

-------------AGCGATATGTGGAGCGGTGAAAGGCTCAGTAACGGGCGATTTATTTGATCTCCTGGGACGGACAACATCGGGAAACTGATGGGAAAACGTCTAAGTTGCA------G---T----TTT-CTGATTGCGACGTAAACCATAGT--GTGCAGGAGAGTAAGATGCCATCCTATCAGTTAGTAAGTAGGGTAAGGGCCTACTTAGACGAAGACGGGTACGGGGAATGAGGGTTTGATTCCGGAGAGGGAGCCTGAGAGATGGCTACCAGGACCAAGGTCAGCAGCAGGCGCGAAAATTATCGAAGCCCGCC-TAGGGGCGATAGTGAGGAGACGTGTAT-ACGAAGTACGTGTAAAGACCGTACTAATAACTGGAGGTCAAGTCTGGTGCCAGCATCCGCGGTAATTCCAGCTCCAGGGGTGTCTATGATGATTGCTGCGATTAAAAAGTCCGTAGTCGAGCTGACTGACTGACCTGCAATG-TGATTGATTAAAGAACGAGCAGGGTTAGGAAAGCAGAGAATTAGGAGCGACCGAGGGCTAGAGTATTGAATGGCGAGAGGTGAAATTTGATGACCCATTCAGGAGTGACAAAGGCGAAGGCACTAGTCAAGGGCGAATCCGATGATCAAGGACGTAGGCTAGAGTTTCGAAAACGATTAGAGACCGGAGTAGTTCTAGCAGTAAACTATGCCGACGCCGTGGTATTTTT--------TAGTATTGCGGAAGAGAAATCAAGTAAGGCTTTGGGGAGAGTACGCGCGCAAGCGATAAATTTAAAGGAAATTGACGGAGGAACACCACAAGGAGTGGAGTGTGCGGCTTAATTTGACTCAACGCGGGACAGCTTACCAGGCCCGATAATCGAGCGAGCGTAGTACGCGATAGGTTAAAAAGTGGTGCATGGCTGCTATCGACAGTTGGGGTGACC-TTAGGGTTAATTCCGGCAAGTAGTGAGACCCCTGCAGATAG--TGGACAGGTA-TTTTT-AAGATACAGGAAGGAAGGGACAAGAGCAGGTCAGTGATGCCCTTAGATGGCCTGGGCTGCACGCGCACTACAGTGGTCATTATAAGTAGAAGTTAGA-TATAAAGATGATCGAGAGGGACTGAGCTTTGTAAGAGGCTCACGAACGAGGAATTGCTAGTAATCGTAGGCTCATTAAGATACGATGAATATGTCCCTGTACCTTGTACACACCGCCCGTCGTTATCGAAGATGGAATTGTGTGCGAACGAGCATTAAGCGAGTGAGCGCATAGTTCTAGATGTGATAAAAGTCGTAACAAGGCAACTGTAGGAGAACCTGTAGTTGGATCATACA

>>R3_GvarM41_HU_Gvar_Droe_KAR-18_1756pb_MG773217

-------------AGCGATATGTGGAGCGGTGAAAGGCTCAGTAACGGGCGATTTATTTGATCTCCTGGGACGGACAACATCGGGAAACTGATGGGAAAACGTCTAAGTTGCA------G---T----TTT-TTGACTGTGACGTAAACCGTAGT--GTGCAGGAGAGTAAGATGCCATCCTATCAGTTAGTAAGTAGGGTAAGGGCCTACTTAGACGAAGACGGGTACGGGGAATGAGGGTTTGATTCCGGAGAGGGAGCCTGAGAGATGGCTACCAGGACCAAGGTCAGCAGCAGGCGCGAAAATTATCGAAGCCCGCC-TAGGGGCGATAGTGAGGAGACGTGT-TAACGAAGTACGTGTAAAGACCGTACTAATAACTGGAGGTCAAGTCTGGTGCCAGCATCCGCGGTAATTCCAGCTCCAGGGGTGTCTATGATGATTGCTGCGATTAAAAAGTCCGTAGTCGAGCTGACTGACTGACCTGCAATG-TGATTGATTAAAGAACGAGCAGGGTTAGGAAAGCAGAGGATTAGGAGCGACCGAGGGCTAGAGTATTGAATGGCGAGAGGTGAAATTTGATGACCCATTCAGGAGTGACAAAGGCGAAGGCACTAGTCAAGGGCGAATCCGATGATCAAGGACGTAGGCTAGAGTTTCGAAAACGATTAGAGACCGGAGTAGTTCTAGCAGTAAACTATGCCGACGCCGTGGTATTAGGTAT-----TAGTATTGCGGAAGAGAAATCAAGTAAGGCTTTGGGGAGAGTACGCGCGCAAGCGATAAATTTAAAGGAAATTGACGGAGGAACACCACAAGGAGTGGAGTGTGCGGCTTAATTTGACTCAACGCGGGACAGCTTACCAGGCCCGAT-ATCGAGCGAGCGTAGTACGCGATAGGTTAAGAAGTGGTGCATGGCTGCTATCGACAGTTGGGGTGACC-TTAGGGTTAATTCCGGCAAGTAGTGAGACCCCTGCAGATAG--TGGACAGGTA-TTTTC-AAGATACAGGAAGGAAGGGACAAGAGCAGGTCAGTGATGCCCTTAGATGGCCTGGGCTGCACGCGCACTACAGTGGTCATTATAAGTAGAAGTTAGA-TATAAAGATGATCGAGAGGGACTGAGCTTTGTAAGAGGCTCACGAACGAGGAATTGCTAGTAATCGTAGGCTCATTAAGATACGATGAATATGTCCCTGTACCTTGTACACACCGCCCGTCGTTATCGAAGATGGAATTGTGTGCGAACAAGCATTAAGCGAGTGAGCGCATAGTTCTAGATGTGATAAAAGTCGTAACAAGGCAACTGTAGGAGAACCTGTAGTTGGATCATACA

>>KR871361_DE_Gpul_Ddue_GRA-15_1075pb_KR871361

---------------------GTGGAGCGGTGAAAGGCTCAGTAACGGGCGATTTATTTAATCTCCTGGGACGGACAACATCGGGAAACTGATGGGAAAACGTCTAAGTTGCA------G---T----TTT-GTGAGTGCGACGTAAACGATAAT-CGTGCAGGAGAGTAAGATGCCATCCTATCAGTTAGTAAGTAGGGTAAGGGCCTACTTAGACGAAGACGGGTACGGGGAATGAGGGTTTGATTCCGGAGAGGGAGCCTGAGAGATGGCTACCAGGACCAAGGTCAGCAGCAGGCGCGAAAATTATCGAAGCCCGCC-TAGGGGCGATAGTGAGGAGACGTGTATATCGAAGTACGTGTAAAGAACGTACTAATAACTGGAGGTCAAGTCTGGTGCCAGCATCCGCGGTAATTCCAGCTCCAGGGGTGTCTATGATGATTGCTGCGATTAAAAAGTCCGTAGTCAAGCTGACTGACTGACCTGCAATG-TGATTGATTAAAGAACGAGCAGGGTTAGGAAAGCAGAGAATTAGGAGCGACCGAGGGCTAGAGTATTGAATGGCGAGAGGTGAAATTTGATGACCCATTCAGGAGTGACAAAGGCGAAGGCACTAGTCAAGGGCGAATCCGATGATCAAGGACGTAGGCTAGAGTTTCGAAAACGATTAGAAACCGGAGTAGTTCTAGCAGTAAACTATGCCGACGCCGTGATATTGTTTTTTGTGGCGGTATTGCGGAAGAGAAATCAAGTAGGGCTTTGGGGAGAGTACGCGCGCAAGCGATAAATTTAAAGGAAATTGACGGAGGAACACCACAAGGAGTGGAGTGTGCGGCTTAATTTGACTCAACGCGGGACAGCTTACCAGGCCCGATAATCGAGCGAGCGTAGTACGCGATAGATTAAAGAGTGGTGCATGGCTGCTATCGACAGTTGGGGTGACC-TTAGGGTTAATTCCGGCAAGTAGTGAGACCCCTGCAGATAG--TGGACAGGTATTTTTT-AAAATACAGGAAGGAAGGGACAAGAGCAGGTCAGTGATGCCCTTAGATGGCCTGGGCTGCACGCGCACTACAGTGGTCATTATAAGTAGAAGTTAGA-TATAAAGATGATC-------------------------------------------------------------------------------------------------------------------------------------------------------------------------------------------------------------------------------

>>M21_PrOv1_PL_Prob_Dmue_KAR-18_1759pb_MG773243

-------------AGCTATATGTGGAGCGGTGAAAGGCTCAGTAACGGGCGATTTATTTAATCTCCTGGGACGGACAACATCGGGAAACTGATGGGAAAACGTCTAAGTTGCA------G---T----TTT-GTTATTGCGACGTAAACGGTAAT-CGTGCAGGAGAGTAAGATGCCATCCTATCAGTTAGTAAGTAGGGTAAGGGCCTACTTAGACGAAGACGGGTACGGGGAATGAGGGTTTGATTCCGGAGAGGGAGCCTGAGAGATGGCTACCAGGACCAAGGTCAGCAGCAGGCGCGAAAATTATCGAAGCCCGCC-TAGGGGCGATAGTGAGGAGACGTGTAT-TCGAAGTACGTGTAAAGAACGTACTAATAACTGGAGGTCAAGTCTGGTGCCAGCATCCGCGGTAATTCCAGCTCCAGGGGTGTCTATGATGATTGCTGCGATTAAAAAGTCCGTAGTCAAGCTGACTGACTGACCTGCAATG-TGATTGATTAAAGAACGAGCAGGGTTAGGAAAGCAGAGAATTAGGAGCGACCGAGGGCTAGAGTATTGAATGGCGAGAGGTGAAATTTGATGACCCATTCAGGAGTGACAAAGGCGAAGGCACTAGTCAAGGGCGAATCCGATGATCAAGGACGTAGGCTAGAGTTTCGAAAACGATTAGAGACCGGAGTAGTTCTAGCAGTAAACTATGCCGACGCCGTGATATTGTTTTTTGTGGCGGTATTGCGGAAGAGAAATCAAGTAAAGCTTTGGGGAGAGTACGCGCGCAAGCGATAAATTTAAAGGAAATTGACGGAGG-ACACCACAAGGAGTGGAGTGTGCGGCTTAATTTGACTCAACGCGGGACAGCTTACCA-GCCCGATAATCGAGCGAGCGTAGTACGCGATAGATTAAAGAGTGGTGCATGGCTGCTATCGACAGTTGGGGTGACC-TTAGGGTTAATTCCGGCAAGTAGTGAGACCCCTGCAGATAG--TGGACAGGTA-TTTTTCAAAATACAGGAAGGAAGGGACAAGAGCAGGTCAGTGATGCCCTTAGATGGCCTGGGCTGCACGCGCACTACAGTGGTCATTATAAGTAGAAGTTAGA-TATAAAGATGATCGAGAGGGACTGAGCTTTGTAAGAGGCTCACGAACGAGGAATTGCTAGTAATCGTAGGCTCATTAAGATACGATGAATATGTCCCTGTACCTTGTACACACCGCCCGTCGTTATCGAAGATGGAATTGTGTGCGAACGAGCAACAAGCGAGTGAGCGCATAGTTCTAGATGTGATAAAAGTCGTAACAAGGCAACTGTAGGAGAACCTGTAGTTGGATCATACA

>>M9_ProbM33_HU_Prob_Dmue_KAR-18_1767pb_MG773232

-------------AGCTATATGTGGAGCGGTGAAAGGCTCAGTAACGGGCGATTTATTTAATCTCCTGGGACGGACAACATCGGGAAACTGATGGGAAAACGTCTAAGTTGCA------T---TAATATTA-GTTAGTGTGACGTAAACGGTAAT-CGTGCAGGAGAGTAAGATGCCATCCTATCAGTTAGTAAGTAGGGTAAGGGCCTACTTAGACGAAGACGGGTACGGGGAATGAGGGTTTGATTCCGGAGAGGGAGCCTGAGAGATGGCTACCAGGACCAAGGTCAGCAGCAGGCGCGAAAATTATCGAAGCCCGCC-TAGGGGCGATAGTGAGGAGACGTGTATAACGAAGTACGTGTAAAGAACGTACTAATAACTGGAGGTCAAGTCTGGTGCCAGCATCCGCGGTAATTCCAGCTCCAGGGGTGTCTATGATGATTGCTGCGATTAAAAAGTCCGTAGTCAAACTGACTGACTGACCTGCAATG-TGATTGATTAAAGAACGAGCAGGGTTAGGAAAGCAGAGAATTAGGAGCGACCGAGGGCTAGAGTATTGAATGGCGAGAGGTGAAATTTGATGACCCATTCAGGAGTGACAAAGGCGAAGGCACTAGTCAAGGGCGAATCCGATGATCAAGGACGTAGGCTAGAGTTTCGAAAACGATTAGAGACCGGAGTAGTTCTAGCAGTAAACTATGCCGACGCCGTGATATTGTTTTTTGTGGCAGTATTGCGGAAGAGAAATCAAGTAAAGCTTTGGGGAGAGTACGCGCGCAAGCGATAAATTTAAAGGAAATTGACGGAGGAACACCACAAGGAGTGGAGTGTGCGGCTTAATTTGACTCAACGCGGGACAGCTTACCAGGCCCGATAATCGAGCGAGCGTAGTACGCGATAGATTAAAGAGTGGTGCATGGCTGCTATCGACAGTTGGGGTGACC-TTAGGGTTAATTCCGGCAAGTAGTGAGACCCCTGCAGATAG--TGGACAGGTA-TTTTT-AAAATACAGGAAGGAAGGGACAAGAGCAGGTCAGTGATGCCCTTAGATGGCCTGGGCTGCACGCGCACTACAGTGGTCATTATAAGTAGAAATTAGA-AATAAAAATGATCGAGAGGGACTGAGCTTTGTAAGAGGCTCACGAACGAGGAATTGCTAGTAATCGTAGGCTCATTAAGATACGATGAATATGTCCCTGTACCTTGTACACACCGCCCGTCGTTATCGAAGATGGAATTGTGTGCGAACGAGCAACAAGCGAGTGAGCGCATAGTTCTAGATGTGATAAAAGTCGTAACAAGGCAACTGTAGGAGAACCTGTAGTTGGATCATACA

>>ALP43-01_DE_Groe_Dsp._N4_ADR-18_797pb_ALP43-01

---------------------GTGGAGCGGTGAAAGGCTCAGTAACGGGCGATTTATTTGATCTCCTGGGACGGACAACATCGGGAAACTGATGGGAAAACGTCTAAGTTGCA------G---T----TTT-GTGATTGTGACGTAAACCATAG---GTGCAGGAGAGTAAGATGCCATCCTATCAGTTAGTAAGTAGGGTAAGTGCCTACTTAGACGAAGACGGGTACGGGGAATGAGGGTTTGATTCCGGAGAGGGAGCCTGAGAGATGGCTACCAGGACCAAGGTCAGCAGCAGGCGCGAAAATTATCGAAGCCCGCC-GAGGGGCGATAGTGAGGAGACGTGTATAACGAAGTACGTGTAAAGAACGTGCTAATAACTGGAGGTCAAGTCTGGTGCCAGCATCCGCGGTAATTCCAGCTCCAGGGGTGTCTATGATGATTGCTGCGATTAAAAAGTCCGTAGTCAATCTGACTGACTGGCCTGCAATG-TGATTGATTAAAGAACGAGCAGGGTTAGGAAAGCAGAGAATTAGGAGCGACCGAGGGCTAGAGTATTGAATGGCGAGAGGTGAAATTTGATGACCCATTCAGGAGTGACAAAGGCGAAGGCACTAGTCAAGGGCGAATCCGATGATCAAGGACGTAGGCTAGAGTTTCGAAAACGATTAGAGACCGGAGTAGTTCTAGCAGTAAACTATGCCGACGCCGTGGTATCTAATTTATC--TGGTATTGCGGAAGAGAAATCAAGTAAGGCTTTGGGGAGAGTACGCGCGCAAGCGATAAATTTAAAGGAAATTGACGGAGGAACACCACAAGGAGTGGAGTGTGCGGCT----------------------------------------------------------------------------------------------------------------------------------------------------------------------------------------------------------------------------------------------------------------------------------------------------------------------------------------------------------------------------------------------------------------------------------------------------------------------------------------------------------------------

>>N1_PLZWC9_PL_Chae_i_D.sp._N1_KAR-18_1752pb_MG773222*

-------------CGTTTAACGTGGAGCGGTGAAAGGCTCAGTAACGGGCGATTTATTTGATCTCCTGGGACGGACAACATCGGGAAACTGATGGGAAAACGTCTAAGTTGCA------G---T----TGT-ATGATTGTGACGTAAACCTTTG---GTGCAGGAGAGTAAGATGCCATCCTATCAGTTAGTAAGTAGGGTAAGGGCCTACTTAGACGAAGACGGGTACGGGGAATGAGGGTTTGATTCCGGAGAGGGAGCCTGAGAGACGGCTACCAGGACCAAGGTCAGCAGCAGGCGCGAAAATTATCGAAGCCCGCC-GAGGGGCGATAGTGAGGAGACGTGTATAACGAAGTACGTGTAAAGAACGTGCTAACAACTGGAGGTCAAGTCTGGTGCCAGCATCCGCGGTAATTCCAGCTCCAGGGGTGTCTATGATGATTGCTGCGATTAAAAAGTCCGTAGTCAATCTGTCTGACTTGCCTGCAATG-TGATTGATTAAAGAACGAGCAGGGCTAGGAAAGCAGAGAATTAGGAGCGACCGAGGGCTAGAGTATTGAATGGCGAGAGGTGAAATTTGATGACCCATTCAGGAGTGACAAAGGCGAAGGCACTAGTCAAGGGCGAATCCGATGATCAAGGACGTAGGCTAGAGTTTCGAAAACGATTAGAGACCGGAGTAGTTCTAGCAGTAAACTATGCCGACGCCGTGGTATGTATTT------ATGTATTGCGGAAGAGAAATCAAGTAAGGCTTTGGGGAGAGTACGCGCGCAAGCGATAAATTTAAAGGAAATTGACGGAGGAACACCACAAGGAGTGGAGTGTGCGGCTTAATTTGACTCAACGCGGGACAGCTTACCAGGCCCGATAATCGAGCGAGCGTAGTACGCGATAGGTTAAAGAGTGGTGCATGGCTGCTATCGACAGTTGGGGTGACC-TTAGGGTTAATTCCGGCAAGTAGTGAGACCCCTGCAGATAG--TGGACAGGTA-TTTTA-AAAATACAGGAAGGAAGGGACAAGAGCAGGTCAGTGATGCCCTTAGATGGCCTGGGCTGCACGCGCACTACAGTGGTCATTATAAGACGAAAGTAGA-TATAAAGATGATCGAGAGGGACTGAGCTTTGTAAGAGGCTCACGAACGAGGAATTGCTAGTAATCGTAGGCTCATTAAGATACGATGAATATGTCCCTGTACCTTGTACACACCGCCCGTCGTTATCGAAGATGGAATTGTGTGCGAACGAGCAATAAGCGAGTGAGCGCATAGTTCTAGATGTGATAAAAGTCGTAACAAGGCAACTGTAGGAGAACCTGTAGTTGGATCATACA

>>PL1-08_PL_Groe_Dber1_ADR-18_249pb_PL1-08

CATGTGTAAGCGAAGCGTAACGTGGAGCGGTGAAAGGCTCAGTAACGGGCGAGTTATTTGTTCTCCTGGGACGGACAACACCGGGAAACTGGTGGGAAAACGTCTAAGTTGCG------G---T----TTT-TTAATCGTGGCGTAAACCATGTG--GTGCAGGAGAGTAAGCTGCCATCCTATCAGTTAGTAAGTAGGGTAAGGGCCTACTTAGACGAAGACGGGTACGGGGAATGAGGGTTTGATTCCGGAGAGGGAGCCTGA--------------------------------------------------------------------------------------------------------------------------------------------------------------------------------------------------------------------------------------------------------------------------------------------------------------------------------------------------------------------------------------------------------------------------------------------------------------------------------------------------------------------------------------------------------------------------------------------------------------------------------------------------------------------------------------------------------------------------------------------------------------------------------------------------------------------------------------------------------------------------------------------------------------------------------------------------------------------------------------------------------------------------------------------------------------------------------------------------------------------

>>AJ438957_GB_Echinogammarus_berilloni_Dber_TER-04_912pb_AJ438957

CATGTGTAAGCGAAGCGTAACGTGGAGCGGTGAAAGGCTCAGTAACGGGCGAGTTATTTGTTCTCCTGGGACGGACAACACCGGGAAACTGGTGGGAAAACGTCTAAGTTGCG------G---T----TTT-TTAATCGTGGCGTAAACCATTTG--GTGCAGGAGAGTAAGCTGCCATCCTATCAGTTAGTAAGTAGGGTAAGGGCCTACTTAGACGAAGACGGGTACGGGGAATGAGGGTTTGATTCCGGAGAGGGAGCCTGAGAGACGGCTACCAGGACCAAGGTCAGCAGCAGGCGCGAAAATTATCGAAGCCCGCA-TAGGGGCGATAGTGAGGAGACGTGTATTACGAAGTGTGTGTAAAGAACGCACTAATAACTGGAGGTCAAGTCTGGTGCCAGCATCCGCGGTAATTCCAGCTCCAGGGGTGTCTATGATGATTGCTGCGATTAAAAAGTCCGTAGTCAAGCTGACTGACTTGCCTGCAATG-TGACTGATTAAGAGACGAGCAGGGCTAGGAAAGCAGAGAATTAGGAGCGACCGAGGGCTAGAGTATTGAATGGCGAGAGGTGAAATTTGATGACCCATTCAGGAGTGACAAAGGCGAAGGCACTAGTCAAGGGCGAATCCGATGATCAAGGACGTAGGCTAGAGTTTCGAAAACGATTAGAGACCGGAGTAGTTCTAGCAGTAAACTATGCCGACGCCGTGGTATGGTATT------CTGTATTGCGGAAGAGAAATCAAGTAAGGCTTTGGGGAGAGTACGCGCGCAAGCGATAAATTTAAAGGAAATTGACGGAGGAACACCACAAGGAGTGGAGTGTGCGGCTTAATTTGACTCAACGCGGGACAGCTTACCAGGCCCGATAATCATACGAGCGTAGTACGCGATAGGTTAAAGAGTGGTGCATGGCTGCTATCGACAGT---------------------------------------------------------------------------------------------------------------------------------------------------------------------------------------------------------------------------------------------------------------------------------------------------------------------------------------------------------------------------------------------------------------------

>>B7_ProbM32_HU_Prob_Dber_KAR-18_1756pb_MG773251*

-------------AGCGTAACGTGGAGCGGTGAAAGGCTCAGTAACGGGCGAGTTATTTGTTCTCCTGGGACGGACAACACCGGGAAACTGGTGGGAAAACGTCTAAGTTGCG------G---T----TTT-TTAATCGTGGCGTAAACCATTTG--GTGCAGGAGAGTAAGCTGCCATCCTATCAGTTAGTAAGTAGGGTAAGGGCCTACTTAGACGAAGACGGGTACGGGGAATGAGGGTTTGATTCCGGAGAGGGAGCCTGAGAGACGGCTACCAGGACCAAGGTCAGCAGCAGGCGCGAAAATTATCGAAGCCCGCG-TAGGGGCGATAGTGAGGAGACGTGTATTACGAAGTGTGTGTAAAGAACGCACTAATAACTGGAGGTCAAGTCTGGTGCCAGCATCCGCGGTAATTCCAGCTCCAGGGGTGTCTATGATGATTGCTGCGATTAAAAAGTCCGTAGTCAAGCTGACTGACTTGCCTGCAATG-TGACTGATTAAGAGACGAGCAGGGCTAGGAAAGCAGAGAATTAGGAGCGACCGAGGGCTAGAGTATTGAATGGCGAGAGGTGAAATTTGATGACCCATTCAGGAGTGACAAAGGCGAAGGCACTAGTCAAGGGCGAATCCGATGATCAAGGACGTAGGCTAGAGTTTCGAAAACGATTAGAGACCGGAGTAGTTCTAGCAGTAAACTATGCCGACGCCGTGGTATGGTATT------CTGTATTGCGGAAGAGAAATCAAGTAAGGCTTTGGGGAGAGTACGCGCGCAAGCGATAAATTTAAAGGAAATTGACGGAGGAACACCACAAGGAGTGGAGTGTGCGGCTTAATTTGACTCAACGCGGGACAGCTTACCAGGCCCGATAATCATACGAGCGTAGTACGCGATAGGTTAGAGAGTGGTGCATGGCTGCTATCGACAGTTGGGGTGACC-TTAGGGTTAATTCCGGCAAGTAGTGAGACCTCTGCAGTTA---TGGACAGGTA-TTTTT-AAGATACAGGAAGGAAGAGACAAGAGCAGGTCAGTGATGCCCTTAGATGGCCTGGGCTGCACGCGCACTACAGTGGTCATTATAAGTAGAAGTTAGATTTTAAAGATGATCGAGAGGGACTGGGCTTTGTAAGAGGCCCAAGAACGAGGAATTGCTAGTAATCGTAGGCTCATTAAGATACGATGAATATGTCCCTGTACCTTGTACACACCGCCCGTCGTTATCGAAGATGGAATTGTGTGCGAACGAGCAACAAGCGAGTGAGCGCATAGTTCTAGATGTGATAAAAGTCGTAACAAGGCAACTGTAGGAGAACCTGTAGTTGGATCATACA

>>KF537632_US_Diporeia_sp._Ddip_WIN-14_1370pb_KF537632

CATGTGTAAGCGAAGCGTAACGTGGAGCGGTGAAAGGCTCAGTAACGGGCGATTTATTTGATCTCCTGGGATGGACAACACCGGGAAACTGGTGGGAAAACATCTAAGTTGCG------G---T----TCTATTGATCGTGATGTAAACCTATGTGGGTGCAGGAGAGTAAGATGCCATCCTATCAGTTAGTAAGTAGGGTAAGGGCCTACTTAGACGAAGACGGGTACGGGGAATGAGGGTTTGATTCCGGAGAGGGAGCCTGAGAGATGGCTACCAGGACCAAGGTCAGCAGCAGGCGCGAAAATTATCGAAGCCCACCATTGGGGCGATAGTGAGGAGACGTGTATAACGAAATACGGGTAAAGAACGTATGTATAACTGGAGGTCAAGTCTGGTGCCAGCATCCGCGGTAATTCCAGCTCCAGGGGTGTCTATGATGATTGCTGCGATTAAAAAGTCCGTAGTCAAGCTGACTGACTAACCTGTAATG-TGGTTGATTAAAAGACGAGAAGGGTTAGGAAAGCAGAGGATTAGGAGCGACCGAGGGCTAGAGTATTGAATGGCGAGAGGTGAAATTTGATGACCCATTCAGGAGTGACAAAGGCGAAGGCACTAGTCAAGGGCGAATCCGATGATCAAGGACGTAGGCTAGAGGTTCGAAAACGATTAGAGACCGGAGTAGTTCTAGCAGTAAACTATGCCGACGCCGTGGTATGTTTTTTA----ATGTATTGCGGAAGAGAAATCAAGTAAGGCTTTGGGGAGAGTACGCGCGCAAGCGATAAATTTAAAGGAAATTGACGGAGGAACACCACAAGGAGTGGAGTGTGTGGCTTAATTTGACTCAACGCGGGACAGCTTACCAGGCCCGATAATCGTACGAGCGTAGTACGCGATAGGTTAAAGAGTGGTGCATGGCTGCTATCGACAGTTGGGGTGACC-TTAGGGTTAATTCCGGCAAGTAGTGAGACCCCTGCTATTAAATAGGACAGGTA-TTTTT-AAAATACAGGAAGGAAGGGACAAGAGCAGGTCGGTGATGCCCTTAGATGGCCTGGGCTGCACGCGCACTACAGTGGTCATTATAATGAGTAAATAGA-AGTAAAAATGATCGAGAGGGACTGGGCTTTGTAAGAGGCCCACGAACGAGGAATTGCTAGTAATCGTAGGCTCATTAAGATACGATGAATATGTCCCTGTACCTTGTACACACCGCCCGTCGTTATCGAAGATGGAATTGTGTGCGAACGAGCATTAAGCGAGTGAGCGCATAGTTCTAGATGTGATAAAAGTCGTAACAAGGCAACTGTAGGAGAACCTGTAGTTGGATCATAC-

>>AJ438960_GB_Orchestia_cavimana_Dcav_TER-04_1326pb_Out_Group

CATGTGTAAGCGAAGC-TATTGTGGAGCGGTGAAAGGCTCAGTAACGGGCGATTTATTTAGTCTTCTGGGACGGACAACACCGGGAAACTGGTGGGAAAACGTCTAAGCTGCGAATCACG---C----TAT-GTGGTTGTGGCAGAAACTGTTA---GTGCAAAAAAGTAAGATGCCATCCTATCAGTTAGTAAGTAGGGTAAGGGCCTACTTAGACGAAGACGGGTACGGGGAATGAGGGTTTGATTCCGGAGAGGGAGCCTGAGAGACGGCTACCAGGACCAAGGTCAGCAGCAGGCGCGAAAATTATCGAAGCCCGCC-TAGGGGCGATAGTGAGGAGACGTGAAT-TTTAGGTGCGTGTAAAGAACGCACTAGCAACTGGAGGTCAAGTCTGGTGCCAGCATCCGCGGTAATTCCAGCTCCAGGGGTGTCTATGATGATTGCTGCGATTAAAAAGTCCGTAGTCAAGCCGCCAGACCAGTCTGGAATGTTTCTTGATCAAGAGACGAGCAGGGCTGGGAAAGCGGAGAATTAGGAGCGACCGAGGGCTAGAGTATTGGGTGGCGAGAGGTGAAATTTGATGACCCATCCAGGAGTGACAAAGGCGAAGGCACTAGTCAAGGGCGAATCCGATGATCAAGGACGTAGGCTAGAGTTTCGAAAACGATTAGAGACCGGAGTAGTTCTAGCAGTAAACTATGCCGACGCCGTGATATGATTTTG-----TTGTATTGCGGAAGAGAAATCAAGTAAGGCTTTGGGGAGAGTACGCGCGCAAGCGATAAATTTAAAGGAAATTGACGGAGGAACACCACAAGGAGTGGAGTGTGCGGCTTAATTTGACTCAACGCGGGACAGCTTACCAGGCCCGATAATCGTACGAGCGTAGTACGCGATAGATTAGAGAGTGGTGCATGGCTGCTATCGACAGTTGGGGTGACC-TTAGGGTTAATTCCGGCAAGTAGTGAGACCCCCGCAG-TAT--TGGACAGGCG-TCGTG-AAGATGCAGGAAGGAGGGGACAAGAGCAGGTCAGTGATGCCCTTAGATGGCCTGGGCTGCACGCGCACTACAGTGGTCATTATAAGGAGAAGTTAGA-AATAAAGATGATCGAGAGGGACTGGGCTTTGTAAGAGGCCCACGAACGAGGAATTGCTAGTAATCGCAGGCTCATTAGGATGCGATGAATATGTCCCTGTACCTTGTACACACCGCCCGTCGTTATCGAAGATGGAATTGTATGCGAACGAGCAGCAAGCGAGTGAGCGTATAGTTCTAGATGTGATAAAAGTCGTAACAAGGCAACTGTAGGAGAACCTGTAGTTGGATCACAC-

Alignment Figure S1 :

>>ALP40-04_DE_Groe_Dmue_ADR-18_822pb_ALP40-04

------------------------------CATGTGTAAGCGAAGCTATATGTGGAGCGGTGAAAGGCTCAGTAACGGGCGATTTATTTAATCTCCTGGGGCGGACAACATCGGGAAACTGATGGGAAAACGTCTAAGTTGCAT------T----AATTTTAGTGTGACGTAAA-CGA-TTATCGTGCAGGAGAGTAAGATGCCATCCTATCAGTTAGTAAGTAGGGTAAGGGCCTACTTAGACGAAGACGGGTACGGGGAATGAGGGTTTGATTCCGGAGAGGGAGCCTGAGAGATGGCTACCAGGACCAAGGTCAGCAGCAGGCGCGAAAATTATCGAAGCCCGCC-TAGGGGCGATAGTGAGGAGACGTGTATAACGAAGTACGTGTAAAGAACGTACTAATAACTGGA-GGTCAAGTCTGGTGCCAGCATCCGCGGTAATTCCAGCTCCAGGGGTGTCTATGATGATTGCTGCGATTAAAAAGTCCGTAGTCAAGCTGCCTG-ACTGACCTGCAATG-TGATTGATTAAGGAACGAGCAGGGTTAGGAAAGCAGAGAATTAGGAGCGACCGAGGGCTAGAGTATTGAATGGCGAGAGGTGAAATTTGATGACCCATTCAGGAGTGACAAAGGCGAAGGCACTAGTCAAGGGCGAATCCGATGATCAAGGACGTAGGCTAGAGTTTCGAAAACGATTAGAGACCGGAGTAGTTCTAGCAGTAAACTATGCCGACGCCGTGGTAT-GG---TTT----TTTGTGGCTGTATTGCGGAAGAGAAATCAAGT-AAGGCTTTGGGGAGAGTACGCGCGCAAGCGATAAATTTAAAGGAAATTGACGGAGGAACACCACAAGGAGTGGAGTGTGCGGCT--------------------------------------------------------------------------------------------------------------------------------------------------------------------------------------------------------------------------------------------------------------------------------------------------------------------------------------------------------------------------------------------------------------------------------------------------------------------------------------------------------------------------------

>>HQ681054_NO_Gdub_D-36_WIL-11_515pb_HQ681054

--------------------------------------------------------------------------------------------------------------------------------------------------------------------------------------------------------------------------------------------------------------------------------------------------------------CCAGGACCAAGGTCAGCAGCAGGCGCGAAAATTATCGAAGCCCGCC-TAGGGGCGATAGTGAGGAGACGTGTATAACGAAGTACGTGTAAAGAACGTACTAATAACTGGA-GGTCAAGTCTGGTGCCAGCATCCGTGGTAATTCCAGCTCCAGGGGTGTCTATGATGATTGCTGCGATTAAAAAGTCCGTAGTCAAGCTGCCTG-ACTGACCTGCAATG-TGATTGATTAAGGAACGAGCAGGGTTAGGAAAGCAGAGAATTAGGAGCGACCGAGGGCTAGAGTATTGAATGGCGAGAGGTGAAATTTGATGACCCATTCAGGAGTGACAAAGGCGAAGGCACTAGTCAAGGGCGAATCCGATGATCAAGGACGTAGGCTAGAGTTTCGAAAACGATTAGAGACCGGAGTAGTTCTAGCAGTAAACTATGCCGACGCCGTGGTAT-GG---TTT----TTTGTGGCTGTATTGCGGAAGAGAAATCAAGT-AAGGCTTTGGGGAGAGTACGCGCGCAAGCGATAAATT-------------------------------------------------------------------------------------------------------------------------------------------------------------------------------------------------------------------------------------------------------------------------------------------------------------------------------------------------------------------------------------------------------------------------------------------------------------------------------------------------------------------------------------------------------------------------------

>>M16_GvarMB5_BY_Gvar_Dmue_KAR-18_1760pb_MG773238

-------------------------------------------AGCTATATGTGGAGCGGTGAAAGGCTCAGTAACGGGCGATTTATTTAATCTCCTGGGGCGGACAACATCGGGAAACTGATGGGAAAACGTCTAAGTTGCAT------T----AATTTTAGTGTGACGTAAA-CGA-TTATCGTGCAGGAGAGTAAGATGCCATCCTATCAGTTAGTAAGTAGGGTAAGGGCCTACTTAGACGAAGACGGGTACGGGGAATGAGGGTTTGATTCCGGAGAGGGAGCCTGAGAGATGGCTACCAGGACCAAGGTCAGCAGCAGGCGCGAAAATTATCGAAGCCCGCC-TAGGGGCGATAGTGAGGAGACGTGTATAACGAAGTACGTGTAAAGAACGTACTAATAACTGGA-GGTCAAGTCTGGTGCCAGCATCCGCGGTAATTCCAGCTCCAGGGGTGTCTATGATGATTGCTGCGATTAAAAAGTCCGTAGTCAAGCTGCCTG-ACTGACCTGCAATG-TGATTGATTAAGGAACGAGCAGGGTTAGGAAAGCAGAGAATTAGGAGCGACCGAGGGCTAGAGTATTGAATGGCGAGAGGTGAAATTTGATGACCCATTCAGGAGTGACAAAGGCGAAGGCACTAGTCAAGGGCGAATCCGATGATCAAGGACGTAGGCTAGAGTTTCGAAAACGATTAGAGACCGGAGTAGTTCTAGCAGTAAAATATGCCGACGCCGTGGTAT-GG---TTT----TTTGTGGCTGTATTGCGGAAGAGAAATCAAGT-AAGGCTTTGGGGAGAGTACGCGCGCAAGCGATAAATTTAAAGGAAATTGACGGAGGAACACCACAAGGAGTGGAGTGTGCGGCTTAATTTGACTCAACGCGGGACAGCTTACCAGGCCCGATAATCGAGCGAGCGTTGTACGCGATAGATTAAAGAGTGGTGCATGGCTGCTATCGACAGTTGGGGTGACC-TTAGGGTTAATTCCGGCAAGTAGTGAGACCCCTGCAGATAG--TGGACAGGTATTTTT-AAAATACAGGAAGGAAGGGACAAGAGCAGGTCAGTGATGCCCTTAGATGGCCTGGGCTGCACGCGCACTACAGTGGTCATTATAAGTAGAAGTTAGA-AGTAAAGATGATCGAGAGGGACTGAGCTTTGTAAGAGGCTCACGAACGAGGAATTGCTAGTAATCGTAGGCTCATTAAGATACGATGAATATGTCCCTGTACCTTGTACACACCGCCCGTCGTTATCGAAGATGGAATTGTGTGCGAACGAGCAACAAGCGAGTGAGCGCATAGTTCTAGATGTGATAAAAGTCGTAACAAGGCAACTGTAGGAGAACCTGCAGTTGGATCATACAGATTTA-TTTT

>>M17_GvarMB6_BY_Gvar_Dmue_KAR-18_1760pb_MG773239

-------------------------------------------AGCTATATGTGGAGCGGTGAAAGGCTCAGTAACGGGCGATTTATTTAATCTCCTGGGGCGGACAACATCGGGAAACTGATGGGAAAACGTCTAAGTTGCAT------T----AATTTTAGTGAGACGTAAA-CGA-TTATCGTGCAGGAGAGTAAGATGCCATCCTATCAGTTAGTAAGTAGGGTAAGGGCCTACTTAGACGAAGACGGGTACGGGGAATGAGGGTTTGATTCCGGAGAGGGAGCCTGAGAGATGGCTACCAGGACCAAGGTCAGCAGCAGGCGCGAAAATTATCGAAGCCCGCC-TAGGGGCGATAGTGAGGAGACGTGTATAACGAAGTACGTGTAAAGAACGTACTAATAACTGGA-GGTCAAGTCTGGTGCCAGCATCCGCGGTAATTCCAGCTCCAGGGGTGTCTATGATGATTGCTGCGATTAAAAAGTCCGTAGTCAAGCTGCCTG-ACTGACCTGCAATG-TGATTGATTAAGGAACGAGCAGGGTTAGGAAAGCAGAGAATTAGGAGCGACCGAGGGCTAGAGTATTGAATGGCGAGAGGTGAAATTTGATGACCCATTCAGGAGTGACAAAGGCGAAGGCACTAGTCAAGGGCGAATCCGATGATCAAGGACGTAGGCTAGAGTTTTGAAAACGATTAGAGACCGGAGTAGTTCTAGCAGTAAACTATGCCGACGCCGTGGTAT-GG---TTT----TTTGTGGCTGTATTGCGGAAGAGAAATCAAGT-AAGGCTTTGGGGAGAGTACGCGCGCAAGCGATAAATTTAAAGGAAATTGACGGAGGAACACCACAAGGAGTGGAGTGTGCGGCTTAATTTGACTCAACGCGGGACAGCTTACCAGGCCCGATAATCGAGCGAGCGTTGTACGCGATAGATTAAAGAGTGGTGCATGGCTGCTATCGACAGTTGGGGTGACC-TTAGGGTTAATTCCGGCAAGTAGTGAGACCCCTGCAGATAG--TGGACAGGTATTTTT-AAAATACAGGAAGGAAGGGACAAGAGCAGGTCAGTGATGCCCTTAGATGGCCTGGGCTGCACGCGCACTACAGTGGTCATTATAAGTAGAAGTTAGA-AGTAAAGATGATCGAGAGGGACTGAGCTTTGTAAGAGGCTCACGAACGAGGAATTGCTAGTAATCGTAGGCTCATTAAGATACGATGAATATGTCCCTGTACCTTGTACACACCGCCCGTCGTTATCGAAGATGGAATTGTGTGCGAACGAGCAACAAGCGAGTGAGCGCATAGTTCTAGATGTGATAAAAGTCGTAACAAGGCAACTGTAGGAGAACCTGCAGTTGGATCATACAGATTTA-TTTT

>>KR871363_DE_Gpul_Ddue_GRA-15_898pb_KR871363

---------------------------------------------------GTGGAGCGGTGAAAGGCTCAGTAACGGGCGATTTATTTAATCTCCTGGGACGGACAACATCGGGAAACTGATGGGAAAACGTCTAAGTTGCAT------T----AATTTTAGTGTGACGTAAA-CGG-ATAACGTGCAGGAGAGTAAGATGCCATCCTATCAGTTAGTAAGTAGGGTAAGGGCCTACTTAGACGAAGACGGGTACGGGGAATGAGGGTTTGATTCCGGAGAGGGAGCCTGAGAGATGGCTACCAGGACCAAGGTCAGCAGCAGGCGCGAAAATTATCGAAGCCCGCC-TAGGGGCGATAGTGAGGAGACGTGTATAACGAAGTACGTGTAAAGAACGTACTAATAACTGGA-GGTCAAGTCTGGTGCCAGCATCCGCGGTAATTCCAGCTCCAGGGGTGTCTATGATGATTGCTGCGATTAAAAAGTCCGTAGTCAAGCTGACTG-ACTGACCTGCAATG-TGATTGATTAAGGAACGAGCAGGGTTAGGAAAGCAGAGAATTAGGAGCGACCGAGGGCTAGAGTATTGAATGGCGAGAGGTGAAATTTGATGACCCATTCAGGAGTGACAAAGGCGAAGGCACTAGTCAAGGGCGAATCCGATGATCAAGGACGTAGGCTAGAGTTTCGAAAACGATTAGAGACCGGAGTAGTTCTAGCAGTAAACTATGCCGACGCCGCGATAT-AG---TTT----TTTGTGGCTGTGTTGCGGAAGAGAAATCAAGT-AAGGCTTTGGGGAGAGTACGCGCGCAAGCGATAAATTTAAAGGAAATTGACGGAGGAACACCACAAGGAGTGGAGTGTGCGGCTTAATTTGACTCAACGCGGGACAGCTTACCAGGCCCGATAATCGAGCGAGCGTTGTACGCGATAGATTAAAGAGTGGTGCATGGCTGCTATCGACAGT-------------------------------------------------------------------------------------------------------------------------------------------------------------------------------------------------------------------------------------------------------------------------------------------------------------------------------------------------------------------------------------------------------------------------------

>>FN434090_FR_Gdub-du_Dmul_KRE-10_1272pb_FN434090

-----------------------------GCATGTGTAAGCGAAGCTAGATGTGGAGCGGTGAAAGGCTCAGTAACGGGCGATTTATTTAATCTCCTGGGACGGACAACATCGGGAAACTGATGGGAAAACGTCTAAGTTGCAT------T----AATTTTAGTGTGACGTAAA-CGG-ATAACGTGCAGGAGAGTAAGATGCCATCCTATCAGTTAGTAAGTAGGGTAAGGGCCTACTTAGACGAAGACGGGTACGGGGAATGAGGGTTTGATTCCGGAGAGGGAGCCTGAGAGATGGCTACCAGGACCAAGGTCAGCAGCAGGCGCGAAAATTATCGAAGCCCGCC-TAGGGGCGATAGTGAGGAGACGTGTATAACGAAGTACGTGTAAAGAACGTACTAATAACTGGA-GGTCAAGTCTGGTGCCAGCATCCGCGGTAATTCCAGCTCCAGGGGTGTCTATGATGATTGCTGCGATTAAAAAGTCCGTAGTCAAGCTGACTG-ACTGACCTGCAATG-TGATTGATTAAGGAACGAGCAGGGTTAGGAAAGCAGAGAATTAGGAGCGACCGAGGGCTAGAGTATTGAATGGCGAGAGGTGAAATTTGATGACCCATTCAGGAGTGACAAAGGCGAAGGCACTAGTCAAGGGCGAATCCGATGATCAAGGACGTAGGCTAGAGTTTCGAAAACGATTAGAGACCGGAGTAGTTCTAGCAGTAAACTATGCCGACGCCGTGATAT-AG---TTT----TTTGTGGCTGTGTTGCGGAAGAGAAATCAAGT-AAGGCTTTGGGGAGAGTACGCGCGCAAGCGATAAATTTAAAGGAAATTGACGGAGGAACACCACAAGGAGTGGAGTGTGCGGCTTAATTTGACTCAACGCGGGACAGCTTACCAGGCCCGATAATCGAGCGAGCGTTGTACGCGATAGATTAAAGAGTGGTGCATGGCTGCTATCGACAGTTGGGGTGACC-TTAGGGTTAATTCCGGCAAGTAGTGAGACCCCTGCAGATAG--TGGACAGGTATTTTT-AAAATACAGGAAGGAAGGGACAAGAGCAGGTCAGTGATGCCCTTAGATGGCCTGGGCTGCACGCGCACTACAGTGGTCATTATAAGTAGAATTTAGA-AGTAAAGATGATCGAGAGGGACTGAGCTTTGTAAGAGGCTCACGAACGAGGAATTGCTAGTAATCGTAGGCTCATTAAGATACGATGAATATGTCCCTGTACCTTGTACACACCGCCCGTCGTTATCGAAGATGGAATTGTGTGCGAACGAGCAACAAGCGAGTGAGCGCATAGTTCTAGATGTGATAA----------------------------------------------------------

>>M18_GvarM48_HU_Gvar_Dmue_KAR-18_1759pb_MG773240

-------------------------------------------AGCTATATGTGGAGCGGTGAAAGGCTCAGTAACGGGCGATTTATTTAATCTCCTGGGACGGACAACATCGGGAAACTGATGGGAAAACGTCTAAGTTGCAT------T----AATGTTAGTGTGACGTAAA-CGA--TATCGTGCAGGAGAGTAAGATGCCATCCTATCAGTTAGTAAGTAGGGTAAGGGCCTACTTAGACGAAGACGGGTACGGGGAATGAGGGTTTGATTCCGGAGAGGGAGCCTGAGAGATGGCTACCAGGACCAAGGTCAGCAGCAGGCGCGAAAATTATCGAAGCCCGCC-TAGGGGCGATAGTGAGGAGACGTGTATAACGAAGTACGTGTAAAGAACGTACTAATAACTGGA-GGTCAAGTCTGGTGCCAGCATCCGCGGTAATTCCAGCTCCAGGGGTGTCTATGATGATTGCTGCGATTAAAAAGTCCGTAGTCAAGCTGACTG-ACTGACCTGCAATG-TGATTGATTAAGGAACGAGCAGGGTTAGGAAAGCAGAGAATTAGGAGCGACCGAGGGCTAGAGTATTGAATGGCGAGAGGTGAAATTTGATGACCCATTCAGGAGTGACAAAGGCGAAGGCACTAGTCAAGGGCGAATCCGATGATCAAGGACGTAGGCTAGAGTTTCGAAAACGATTAGAGACCGGAGTAGTTCTAGCAGTAAACTATGCCGACGCCGTGATAT-AG---TTT----TTTGTGGCTGTATTGCGGAAGAGAAATCAAGT-AAGGCTTTGGGGAGAGTACGCGCGCAAGCGATAAATTTAAAGGAAATTGACGGAGGAACACCACAAGGAGTGGAGTGTGCGGCTTAATTTGACTCAACGCGGGACAGCTTACCAGGCCCGATAATCGAGCGAGCGTTGTACGCGATAGATTAAAGAGTGGTGCATGGCTGCTATCGACAGTTGGGGTGACCTTTAGGGTTAATTCCGGCAAGTAGTGAGACCCCTGCAGATAG--TGGACAGGTATTTTT-AAAATACAGGAAGGAAGGGACAAGAGCAGGTCAGTGATGCCCTTAGATGGCCTGGGCTGCACGCGCACTACAGTGGTCATTATAAGTAGAAGTTAGA-AGTAAAGATGATCGAGAGGGACTGAGCTTTGTAAGAGGCTCACGAACGAGGAATTGCTAGTAATCGTAGGCTCATTAAGATACGATGAATATGTCCCTGTACCTTGTACACACCGCCCGTCGTTATCGAAGATGGAATTGTGTGCGAACGAGCAACAAGCGAGTGAGCGCATAGTTCTAGATGTGATAAAAGTCGTAACAAGGCAACTGTAGGAGAACCTGTAGTTGGATCATACAGATTTA-ATTT

>>EF095540_NL_Dvil_Dmue_WAT-07_376pb_EF095540

--------------------------------------------------TGTGGAGCGGTGAAAGGCTCAGTAACGGGCGATTTATTTAATCTCCTGGGGCGGACAACATCCGGAAACTGATGGGAAAACGTCTAAGTTGCAT------T----AATTTTAGTGTGACGTAAA-CGA-TTATCGTGCAGGAGAGTAAGATGCCATCCTATCAGTTAGTAAGTAGGGTAAGGGCCTACTTAGACGAAGACGGGTACGGGGAATGAGGGTTTGATTCCGGAGAGGGAGCCTGAGAGATGGCTACCAGGACCAAGGTCAGCAGCAGGCGCGAAAATTATCGAAGCCCGCC-TAGGGGCGATAGTGAGGAGACGTGTATAACGAAGTACGTGTAAAGAACGTACTAATAACTGGA-GGTCAAGTCTGGTGCCAGCAGCCGCGG-------------------------------------------------------------------------------------------------------------------------------------------------------------------------------------------------------------------------------------------------------------------------------------------------------------------------------------------------------------------------------------------------------------------------------------------------------------------------------------------------------------------------------------------------------------------------------------------------------------------------------------------------------------------------------------------------------------------------------------------------------------------------------------------------------------------------------------------------------------------------------------------------------------------------------------------------------------------------------------

>>MF399468_GB_Gpul_Dsp_IRO-17_917pb_MF399468

------------------------------CATGTGTAAGCGAAGCTAGATGTGGAGCGGTGAAAGGCTCAGTAACGGGCGATTTATTTAATCTTCTGGGACGGACAACATCGGGAAACTGATGGGAAAACGTCTAAGTTGCAT------T----AATTTTAGTGTGACGTAAA-CGG-ATAACGTGCAGGAGAGTAAGATGCCATCCTATCAGTTAGTAAGTAGGGTAAGGGCCTACTTAGACGAAGACGGGTACGGGGAATGAGGGTTTGATTCCGGAGAGGGAGCCTGAGAGATGGCTACCAGGACCAAGGTCAGCAGCAGGCGCGAAAATTATCGAAGCCCGCC-TAGGGGCGATAGTGAGGAGACGTGTATAACGAAGTACGTGTAAAGAACGTACTAATAACTGGA-GGTCAAGTCTGGTGCCAGCATCCGCGGTAATTCCAGCTCCAGGGGTGTCTATGATGATTGCTGCGATTAAAAAGTCCGTAGTCAAGCTGACTG-ACTGACCTGCAATG-TGATTGATTAAGGAACGAGCAGGGTTAGGAAAGCAGAGAATTAGGAGCGACCGAGGGCTAGAGTATTGAATGGCGAGAGGTGAAATTTGATGACCCATTCAGGAGTGACAAAGGCGAAGGCACTAGTCAAGGGCGAATCCGATGATCAAGGACGTAGGCTAGAGTTTCGAAAACGATTAGAGACCGGAGTAGTTCTAGCAGTAAACTATGCCGACGCCGCGATAT-AG---TTT----TTTGTGGCTGTGTTGCGGAAGAGAAATCAAGT-AAGGCTTTGGGGAGAGTACGCGCGCAAGCGATAAATT--AAAGGAAATGACGGAGGAACACCACAAGGAGTGGAGTGTGCGGCTTAATTTGACTCAACGCGGGACAGCTTACCAGGCCCGATAATCGAGCGAGCGTTGTACGCGATAGATTAAAGAGTGGTGCATGGCTGCTATCGACAGT-------------------------------------------------------------------------------------------------------------------------------------------------------------------------------------------------------------------------------------------------------------------------------------------------------------------------------------------------------------------------------------------------------------------------------

>>KR871361_DE_Gpul_Ddue_GRA-15_1075pb_KR871361

---------------------------------------------------GTGGAGCGGTGAAAGGCTCAGTAACGGGCGATTTATTTAATCTCCTGGGACGGACAACATCGGGAAACTGATGGGAAAACGTCTAAGTTGCAG------T----TTTGTGAGTGCGACGTAAA-CGA-TAATCGTGCAGGAGAGTAAGATGCCATCCTATCAGTTAGTAAGTAGGGTAAGGGCCTACTTAGACGAAGACGGGTACGGGGAATGAGGGTTTGATTCCGGAGAGGGAGCCTGAGAGATGGCTACCAGGACCAAGGTCAGCAGCAGGCGCGAAAATTATCGAAGCCCGCC-TAGGGGCGATAGTGAGGAGACGTGTATATCGAAGTACGTGTAAAGAACGTACTAATAACTGGA-GGTCAAGTCTGGTGCCAGCATCCGCGGTAATTCCAGCTCCAGGGGTGTCTATGATGATTGCTGCGATTAAAAAGTCCGTAGTCAAGCTGACTG-ACTGACCTGCAATG-TGATTGATTAAAGAACGAGCAGGGTTAGGAAAGCAGAGAATTAGGAGCGACCGAGGGCTAGAGTATTGAATGGCGAGAGGTGAAATTTGATGACCCATTCAGGAGTGACAAAGGCGAAGGCACTAGTCAAGGGCGAATCCGATGATCAAGGACGTAGGCTAGAGTTTCGAAAACGATTAGAAACCGGAGTAGTTCTAGCAGTAAACTATGCCGACGCCGTGATAT-TG---TTT----TTTGTGGCGGTATTGCGGAAGAGAAATCAAGT-AGGGCTTTGGGGAGAGTACGCGCGCAAGCGATAAATTTAAAGGAAATTGACGGAGGAACACCACAAGGAGTGGAGTGTGCGGCTTAATTTGACTCAACGCGGGACAGCTTACCAGGCCCGATAATCGAGCGAGCGTAGTACGCGATAGATTAAAGAGTGGTGCATGGCTGCTATCGACAGTTGGGGTGACC-TTAGGGTTAATTCCGGCAAGTAGTGAGACCCCTGCAGATAG--TGGACAGGTATTTTTTAAAATACAGGAAGGAAGGGACAAGAGCAGGTCAGTGATGCCCTTAGATGGCCTGGGCTGCACGCGCACTACAGTGGTCATTATAAGTAGAAGTTAGA-TATAAAGATGATC------------------------------------------------------------------------------------------------------------------------------------------------------------------------------------------------------------------------------------------

>>KR871362_DE_Dhae_Ddue_GRA-15_1085pb_KR871362

---------------------------------------------------GTGGAGCGGTGAAAGGCTCAGTAACGGGCGATTTATTTAATCTCCTGGGACGGACAACATCGGGAAACTGATGGGAAAACGTCTAAGTTGCAG------T----TTTGTTATTGCGACGTAAA-CGG-TAATCGTGCAGGAGAGTAAGATGCCATCCTATCAGTTAGTAAGTAGGGTAAGGGCCTACTTAGACGAAGACGGGTACGGGGAATGAGGGTTTGATTCCGGAGAGGGAGCCTGAGAGATGGCTACCAGGACCAAGGTCAGCAGCAGGCGCGAAAATTATCGAAGCCCGCC-TAGGGGCGATAGTGAGGAGACGTGTATATCGAAGTACGTGTAAAGAACGTACTAATAACTGGA-GGTCAAGTCTGGTGCCAGCATCCGCGGTAATTCCAGCTCCAGGGGTGTCTATGATGATTGCTGCGATTAAAAAGTCCGTAGTCAAGCTGACTG-ACTGACCTGCAATG-TGATTGATTAAAGAACGAGCAGGGTTAGGAAAGCAGAGAATTAGGAGCGACCGAGGGCTAGAGTATTGAATGGCGAGAGGTGAAATTTGATGACCCATTCAGGAGTGACAAAGGCGAAGGCACTAGTCAAGGGCGAATCCGATGATCAAGGACGTAGGCTAGAGTTTCGAAAACGATTAGAGACCGGAGTAGTTCTAGCAGTAAACTATGCCGACGCCGTGATAT-TG---TTT----TTTGTGGCGGTATTGCGGAAGAGAAATCAAGT-AAGGCTTTGGGGAGAGTACGCGCGCAAGCGATAAATTTAAAGGAAATTGACGGAGGAACACCACAAGGAGTGGAGTGTGCGGCTTAATTTGACTCAACGCGGGACAGCTTACCAGGCCCGATAATCGAGCGAGCGTAGTACGCGATAGATTAAAGAGTGGTGCATGGCTGCTATCGACAGTTGGGGTGACC-TTAGGGTTAATTCCGGCAAGTAGTGAGACCCCTGCAGATAG--TGGACAGGTATTTTTCAAAATACAGGAAGGAAGGGACAAGAGCAGGTCAGTGATGCCCTTAGATGGCCTGGGCTGCACGCGCACTACAGTGGTCATTATAAGTAGAAGTTAGA-TATAAAGATGATCGAGAGGGACT--------------------------------------------------------------------------------------------------------------------------------------------------------------------------------------------------------------------------------

>>M20_DBP2_PL_Dhae_Dmue_KAR-18_1760pb_MG773242

-------------------------------------------AGCTATATGTGGAGCGGTGAAAGGCTCAGTAACGGGCGATTTATTTAATCTCCTGGGACGGACAACATCGGGAAACTGATGGGAAAACGTCTAAGTTGCAG------T----TTTGTTATTGCGACGTAAA-CGG-TAATCGTGCAGGAGAGTAAGATGCCATCCTATCAGTTAGTAAGTAGGGTAAGGGCCTACTTAGACGAAGACGGGTACGGGGAATGAGGGTTTGATTCCGGAGAGGGAGCCTGAGAGATGGCTACCAGGACCAAGGTCAGCAGCAGGCGCGAAAATTATCGAAGCCCGCC-TAGGGGCGATAGTGAGGAGACGTGTAT-TCGAAGTACGTGTAAAGAACGTACTAATAACTGGA-GGTCAAGTCTGGTGCCAGCATCCGCGGTAATTCCAGCTCCAGGGGTGTCTATGATGATTGCTGCGATTAAAAAGTCCGTAGTCAAGCTGACTG-ACTGACCTGCAATG-TGATTGATTAAAGAACGAGCAGGGTTAGGAAAGCAGAGAATTAGGAGCGACCGAGGGCTAGAGTATTGAATGGCGAGAGGTGAAATTTGATGACCCATTCAGGAGTGACAAAGGCGAAGGCACTAGTCAAGGGCGAATCCGATGATCAAGGACGTAGGCTAGAGTTTCGAAAACGATTAGAGACCGGAGTAGTTCTAGCAGTAAACTATGCCGACGCCGTGATAT-TG---TTT----TTTGTGGCGGTATTGCGGAAGAGAAATCAAGT-AAGGCTTTGGGGAGAGTACGCGCGCAAGCGATAAATTTAAAGGAAATTGACGGAGGAACACCACAAGGAGTGGAGTGTGCGGCTTAATTTGACTCAACGCGGGACAGCTTACCAGGCCCGATAATCGAGCGAGCGTAGTACGCGATAGATTAAAGAGTGGTGCATGGCTGCTATCGACAGTTGGGGTGACC-TTAGGGTTAATTCCGGCAAGTAGTGAGACCCCTGCAGATAG--TGGACAGGTATTTTTCAAAATACAGGAAGGAAGGGACAAGAGCAGGTCAGTGATGCCCTTAGATGGCCTGGGCTGCACGCGCACTACAGTGGTCATTATAAGTAGAAGTTAGA-TATAAAGATGATCGAGAGGGACTGAGCTTTGTAAGAGGCTCACGAACGAGGAATTGCTAGTAATCGTAGGCTCATTAAGATACGATGAATATGTCCCTGTACCTTGTACACACCGCCCGTCGTTATCGAAGATGGAATTGTGTGCGAACGAGCAACAAGCGAGTGAGCGCATAGTTCTAGATGTGATAAAAGTCGTAACAAGGCAACTGTAGGAGAACCTGTAGTTGGATCATACAGATTTATTTCT

>>M23_ZWPr1_PL_Prob_Dmue_KAR-18_1761pb_MG773245

-------------------------------------------AGCTATATGTGGAGCGGTGAAAGGCTCAGTAACGGGCGATTTATTTAATCTCCTGGGACGGACAACATCGGGAAACTGATGGGAAAACGTCTAAGTTGCAG------T----TTTGTTATTGTGACGTAAA-CGG-TAATCGTGCAGGAGAGTAAGATGCCATCCTATCAGTTAGTAAGTAGGGTAAGGGCCTACTTAGACGAAGACGGGTACGGGGAATGAGGGTTTGATTCCGGAGAGGGAGCCTGAGAGATGGCTACCAGGACCAAGGTCAGCAGCAGGCGCGAAAATTATCGAAGCCCGCC-TAGGGGCGATAGTGAGGAGACGTGTAT-TCGAAGTACGTGTAAAGAACGTACTAATAACTGGA-GGTCAAGTCTGGTGCCAGCATCCGCGGTAATTCCAGCTCCAGGGGTGTCTATGATGATTGCTGCGATTAAAAAGTCCGTAGTCAAGCTGACTG-ACTGACCTGCAATG-TGATTGATTAAAGAACGAGCAGGGTTAGGAAAGCAGAGAATTAGGAGCGACCGAGGGCTAGAGTATTGAATGGCGAGAGGTGAAATTTGATGACCCATTCAGGAGTGACAAAGGCGAAGGCACTAGTCAAGGGCGAATCCGATGATCAAGGACGTAGGCTAGAGTTTCGAAAACGATTAGAGACCGGAGTAGTTCTAGCAGTAAACTATGCCGACGCCGTGATAT-TG---TTT----TTTGTGGCGGTATTGCGGAAGAGAAATCAAGT-AAGGCTTTGGGGAGAGTACGCGCGCAAGCGATAAATTTAAAGGAAATTGACGGAGGAACACCACAAGGAGTGGAGTGTGCGGCTTAATTTGACTCAACGCGGGACAGCTTACCAGGCCCGATAATCGAGCGAGCGTAGTACGCGATAGATTAAAGAGTGGTGCATGGCTGCTATCGACAGTTGGGGTGACC-TTAGGGTTAATTCCGGCAAGTAGTGAGACCCCTGCAGATAG--TGGACAGGTATTTTTCAAAATACAGGAAGGAAGGGACAAGAGCAGGTCAGTGATGCCCTTAGATGGCCTGGGCTGCACGCGCACTACAGTGGTCATTATAAGTAGAAGTTAGA-TATAAAGATGATCGAGAGGGACTGAGCTTTGTAAGAGGCTCACGAACGAGGAATTGCTAGTAATCGTAGGCTCATTAAGATACGATGAATATGTCCCTGTACCTTGTACACACCGCCCGTCGTTATCGAAGATGGAATTGTGTGCGAACGAGCAACAAGCGAGTGAGCGCATAGTTCTAGATGTGATAAAAGTCGTAACAAGGCAACTGTAGGAGAACCTGTAGTTGGATCATACAGATTTATTTTT

>>M21_PrOv1_PL_Prob_Dmue_KAR-18_1759pb_MG773243

-------------------------------------------AGCTATATGTGGAGCGGTGAAAGGCTCAGTAACGGGCGATTTATTTAATCTCCTGGGACGGACAACATCGGGAAACTGATGGGAAAACGTCTAAGTTGCAG------T----TTTGTTATTGCGACGTAAA-CGG-TAATCGTGCAGGAGAGTAAGATGCCATCCTATCAGTTAGTAAGTAGGGTAAGGGCCTACTTAGACGAAGACGGGTACGGGGAATGAGGGTTTGATTCCGGAGAGGGAGCCTGAGAGATGGCTACCAGGACCAAGGTCAGCAGCAGGCGCGAAAATTATCGAAGCCCGCC-TAGGGGCGATAGTGAGGAGACGTGTAT-TCGAAGTACGTGTAAAGAACGTACTAATAACTGGA-GGTCAAGTCTGGTGCCAGCATCCGCGGTAATTCCAGCTCCAGGGGTGTCTATGATGATTGCTGCGATTAAAAAGTCCGTAGTCAAGCTGACTG-ACTGACCTGCAATG-TGATTGATTAAAGAACGAGCAGGGTTAGGAAAGCAGAGAATTAGGAGCGACCGAGGGCTAGAGTATTGAATGGCGAGAGGTGAAATTTGATGACCCATTCAGGAGTGACAAAGGCGAAGGCACTAGTCAAGGGCGAATCCGATGATCAAGGACGTAGGCTAGAGTTTCGAAAACGATTAGAGACCGGAGTAGTTCTAGCAGTAAACTATGCCGACGCCGTGATAT-TG---TTT----TTTGTGGCGGTATTGCGGAAGAGAAATCAAGT-AAAGCTTTGGGGAGAGTACGCGCGCAAGCGATAAATTTAAAGGAAATTGACGGAGG-ACACCACAAGGAGTGGAGTGTGCGGCTTAATTTGACTCAACGCGGGACAGCTTACCA-GCCCGATAATCGAGCGAGCGTAGTACGCGATAGATTAAAGAGTGGTGCATGGCTGCTATCGACAGTTGGGGTGACC-TTAGGGTTAATTCCGGCAAGTAGTGAGACCCCTGCAGATAG--TGGACAGGTATTTTTCAAAATACAGGAAGGAAGGGACAAGAGCAGGTCAGTGATGCCCTTAGATGGCCTGGGCTGCACGCGCACTACAGTGGTCATTATAAGTAGAAGTTAGA-TATAAAGATGATCGAGAGGGACTGAGCTTTGTAAGAGGCTCACGAACGAGGAATTGCTAGTAATCGTAGGCTCATTAAGATACGATGAATATGTCCCTGTACCTTGTACACACCGCCCGTCGTTATCGAAGATGGAATTGTGTGCGAACGAGCAACAAGCGAGTGAGCGCATAGTTCTAGATGTGATAAAAGTCGTAACAAGGCAACTGTAGGAGAACCTGTAGTTGGATCATACAGATTTATTTTT

>>M2_ALP23-22_CH_Dvil_Dmue_KAR-18_1767pb_MG773225

-------------------------------------------AGCTATATGTGGAGCGGTGAAAGGCTCAGTAACGGGCGATTTATTTAATCTCCTGGGACGGACAACATCGGGAAACTGATGGGAAAACGTTTTAGTTGCAT------TAATATTAGTTAGTGTGACGTAAA-CGG-TAATCGTGCAGGAGAGTAAGATGCCATCCTATCAGTTAGTAAGTAGGGTAAGGGCCTACTTAGACGAAGACGGGTACGGGGAATGAGGGTTTGATTCCGGAGAGGGAGCCTGAGAGATGGCTACCAGGACCAAGGTCAGCAGCAGGCGCGAAAATTATCGAAGCCCGCC-TAGGGGCGATAGTGAGGAGACGTGTATAACGAAGTACGTGTAAAGAACGTACTAATAACTGGA-GGTCAAGTCTGGTGCCAGCATCCGCGGTAATTCCAGCTCCAGGGGTGTCTATGATGATTGCTGCGATTAAAAAGTCCGTAGTCAAACTGACTG-ACTGACCTGCAATG-TGATTGATTAAAGAACGAGCAGGGTTAGGAAAGCAGAGAATTAGGAGCGACCGAGGGCTAGAGTATTGAATGGCGAGAGGTGAAATTTGATGACCCATTCAGGAGTGACAAAGGCGAAGGCACTAGTCAAGGGCGAATCCGATGATCAAGGACGTAGGCTAGAGTTTCGAAAACGATTAGAGACCGGAGTAGTTCTAGCAGTAAACTATGCCGACGCCGTGATAT-TG---TTT----TTTGTGGCAGTATTGCGGAAGAGAAATCAAGT-AAGGCTTTGGGGAGAGTACGCGCGCAAGCGATAAATTTAAAGGAAATTGACGGAGGAACACCACAAGGAGTGGAGTGTGCGGCTTAATTTGACTCAACGCGGGACAGCTTACCAGGCCCGATAATCGAGCGAGCGTAGTACGCGATAGATTAAAGAGTGGTGCATGGCTGCTATCGACAGTTGGGGTGACC-TTAGGGTTAATTCCGGCAAGTAGTGAGACCCCTGCAGATAG--TGGACAGGTATTTTT-AAAATACAGGAAGGAAGGGACAAGAGCAGGTCAGTGATGCCCTTAGATGGCCTGGGCTGCACGCGCACTACAGTGGTCATTATAAGTAGAAATTAGA-AGTAAAAATGATCGAGAGGGACTGAGCTTTGTAAGAGGCTCACGAACGAGGAATTGCTAGTAATCGTAGGCTCATTAAGATACGATGAATATGTCCCTGTACCTTGTACACACCGCCCGTCGTTATCGAAGATGGAATTGTGTGCGAACGAGCAACAAGCGAGTGAGCGCATAGTTCTAGATGTGATAAAAGTCGTAACAAGGCAACTGTAGGAGAACCTGTAGTTGGATCATACAGATTTA-TATT

>>M13_GaeqM34_HU_Gaeq_Dmue_KAR-18_1767pb_MG773236

-------------------------------------------AGCTATATGTGGAGCGGTGAAAGGCTCAGTAACGGGCGATTTATTTAATCTCCTGGGACGGACAACATCGGGAAACTGATGGGAAAACGTCTAAGTTGCAT------TAATATTAGTTAGTGTGACGTAAA-CGG-TAATCGTGCAGGAGAGTAAGATGCCATCCTATCAGTTAGTAAGTAGGGTAAGGGCCTACTTAGACGAAGACGGGTACGGGGAATGAGGGTTTGATTCCGGAGAGGGAGCCTGAGAGATGGCTACCAGGACCAAGGTCAGCAGCAGGCGCGAAAATTATCGAAGCCCGCC-TAGGGGCGATAGTGAGGAGACGTGTATAACGAAGTACGTGTAAAGAACGTACTAATAACTGGA-GGTCAAGTCTGGTGCCAGCATCCGCGGTAATTCCAGCTCCAGGGGTGTCTATGATGATTGCTGCGATTAAAAAGTCCGTAGTCAAACTGACTG-ACTGACCTGCAATG-TGATTGATTAAAGAACGAGCAGGGTTAGGAAAGCAGAGAATTAGGAGCGACCGAGGGCTAGAGTATTGAATGGCGAGAGGTGAAATTTGATGACCCATTCAGGAGTGACAAAGGCGAAGGCACTAGTCAAGGGCGAATCCGATGATCAAGGACGTAGGCTAGAGTTTCGAAAACGATTAGAGACCGGAGTAGTTCTAGCAGTAAACTATGCCGACGCCGTGATAT-TG---TTT----TTTGTGGCGGTATTGCGGAAGAGAAATCAAGT-AAGGCTTTGGGGAGAGTACGCGCGCAAGCGATAAATTTAAAGGAAATTGACGGAGGAACACCACAAGGAGTGGAGTGTGCGGCTTAATTTGACTCAACGCGGGACAGCTTACCAGGCCCGATAATCGAGCGAGCGTAGTACGCGATAGATTAAAGAGTGGTGCATGGCTGCTATCGACAGTTGGGGTGACC-TTAGGGTTAATTCCGGCAAGTAGTGAGACCCCTGCAGATAG--TGGACAGGTATTTTT-AAAATACAGGAAGGAAGGGACAAGAGCAGGTCAGTGATGCCCTTAGATGGCCTGGGCTGCACGCGCACTACAGTGGTCATTATAAGTAGAAATTAGA-AGTAAAAATGATCGAGAGGGACTGAGCTTTGTAAGAGGCTCACGAACGAGGAATTGCTAGTAATCGTAGGCTCATTAAGATACGATGAATATGTCCCTGTACCTTGTACACACCGCCCGTCGTTATCGAAGATGGAATTGTGTGCGAACGAGCAACAAGCGAGTGAGCGCATAGTTCTAGATGTGATAAAAGTCGTAACAAGGCAACTGTAGGAGAACCTGTAGTTGGATCATACAGATTTA-TATT

>>M3_ALP25-17_CH_Dvil_Dmue_KAR-18_1767pb_MG773226

-------------------------------------------AGCTATATGTGGAGCGGTGAAAGGCTCAGTAACGGGCGATTTATTTAATCTCCTGGGACGGACAACATCGGGAAACTGATGGGAAAACGTCTAAGTTGCAT------TAATATTAGTTAGTGTGACGTAAA-CGG-TAATCGTGCAGGAGAGTAAGATGCCATCCTATCAGTTAGTAAGTAGGGTAAGGGCCTACTTAGACGAAGACGGGTACGGGGAATGAGGGTTTGATTCCGGAGAGGGAGCCTGAGAGATGGCTACCAGGACCAAGGTCAGCAGCAGGCGCGAAAATTATCGAAGCCCGCC-TAGGGGCGATAGTGAGGAGACGTGTATAACGAAGTACGTGTAAAGAACGTACTAATAACTGGA-GGTCAAGTCTGGTGCCAGCATCCGCGGTAATTCCAGCTCCAGGGGTGTCTATGATGATTGCTGCGATTAAAAAGTCCGTAGTCAAACTGACTG-ACTGACCTGCAATG-TGATTGATTAAAGAACGAGCAGGGTTAGGAAAGCAGAGAATTAGGAGCGACCGAGGGCTAGAGTATTGAATGGCGAGAGGTGAAATTTGATGACCCATTCAGGAGTGACAAAGGCGAAGGCACTAGTCAAGGGCGAATCCGATGATCAAGGACGTAGGCTAGAGTTTCGAAAACGATTAGAGACCGGAGTAGTTCTAGCAGTAAACTATGCCGACGCCGTGATAT-TG---TTT----TTTGTGGCAGTATTGCGGAAGAGAAATCAAGT-AAGGCTTTGGGGAGAGTACGCGCGCAAGCGATAAATTTAAAGGAAATTGACGGAGGAACACCACAAGGAGTGGAGTGTGCGGCTTAATTTGACTCAACGCGGGACAGCTTACCAGGCCCGATAATCGAGCGAGCGTAGTACGCGATAGATTAAAGAGTGGTGCATGGCTGCTATCGACAGTTGGGGTGACC-TTAGGGTTAATTCCGGCAAGTAGTGAGACCCCTGCAGATAG--TGGACAGGTATTTTT-AAAATACAGGAAGGAAGGGACAAGAGCAGGTCAGTGATGCCCTTAGATGGCCTGGGCTGCACGCGCACTACAGTGGTCATTATAAGTAGAAATTAGA-AATAAAGATGATCGAGAGGGACTGAGCTTTGTAAGAGGCTCACGAACGAGGAATTGCTAGTAATCGTAGGCTCATTAAGATACGATGAATATGTCCCTGTACCTTGTACACACCGCCCGTCGTTATCGAAGATGGAATTGTGTGCGAACGAGCAACAAGCGAGTGAGCGCATAGTTCTAGATGTGATAAAAGTCGTAACAAGGCAACTGTAGGAGAACCTGTAGTTGGATCATACAGATTTA-TATT

>>M4_ALP31-21_CH_Dvil_Dmue_KAR-18_1767pb_MG773227*

-------------------------------------------AGCTATATGTGGAGCGGTGAAAGGCTCAGTAACGGGCGATTTATTTAATCTCCTGGGACGGACAACATCGGGAAACTGATGGGAAAACGTCTAAGTTGCAT------TAATATTAGTTAGTGTGACGTAAA-CGG-TAATCGTGCAGGAGAGTAAGATGCCATCCTATCAGTTAGTAAGTAGGGTAAGGGCCTACTTAGACGAAGACGGGTACGGGGAATGAGGGTTTGATTCCGGAGAGGGAGCCTGAGAGATGGCTACCAGGACCAAGGTCAGCAGCAGGCGCGAAAATTATCGAAGCCCGCC-TAGGGGCGATAGTGAGGAGACGTGTATAACGAAGTACGTGTAAAGAACGTACTAATAACTGGA-GGTCAAGTCTGGTGCCAGCATCCGCGGTAATTCCAGCTCCAGGGGTGTCTATGATGATTGCTGCGATTAAAAAGTCCGTAGTCAAACTGACTG-ACTGACCTGCAATG-TGATTGATTAAAGAACGAGCAGGGTTAGGAAAGCAGAGAATTAGGAGCGACCGAGGGCTAGAGTATTGAATGGCGAGAGGTGAAATTTGATGACCCATTCAGGAGTGACAAAGGCGAAGGCACTAGTCAAGGGCGAATCCGATGATCAAGGACGTAGGCTAGAGTTTCGAAAACGATTAGAGACCGGAGTAGTTCTAGCAGTAAACTATGCCGACGCCGTGATAT-TG---TTT----TTTGTGGCAGTATTGCGGAAGAGAAATCAAGT-AAGGCTTTGGGGAGAGTACGCGCGCAAGCGATAAATTTAAAGGAAATTGACGGAGGAACACCACAAGGAGTGGAGTGTGCGGCTTAATTTGACTCAACGCGGGACAGCTTACCAGGCCCGATAATCGAGCGAGCGTAGTACGCGATAGATTAAAGAGTGGTGCATGGCTGCTATCGACAGTTGGGGTGACC-TTAGGGTTAATTCCGGCAAGTAGTGAGACCCCTGCAGATAG--TGGACAGGTATTTTT-AAAATACAGGAAGGAAGGGACAAGAGCAGGTCAGTGATGCCCTTAGATGGCCTGGGCTGCACGCGCACTACAGTGGTCATTATAAGTAGAAATTAGA-AATAAAAATGATCGAGAGGGACTGAGCTTTGTAAGAGGCTCACGAACGAGGAATTGCTAGTAATCGTAGGCTCATTAAGATACGATGAATATGTCCCTGTACCTTGTACACACCGCCCGTCGTTATCGAAGATGGAATTGTGTGCGAACGAGCAACAAGCGAGTGAGCGCATAGTTCTAGATGTGATAAAAGTCGTAACAAGGCAACTGTAGGAGAACCTGTAGTTGGATCATACAGATTTA-TATT

>>M9_ProbM33_HU_Prob_Dmue_KAR-18_1767pb_MG773232

-------------------------------------------AGCTATATGTGGAGCGGTGAAAGGCTCAGTAACGGGCGATTTATTTAATCTCCTGGGACGGACAACATCGGGAAACTGATGGGAAAACGTCTAAGTTGCAT------TAATATTAGTTAGTGTGACGTAAA-CGG-TAATCGTGCAGGAGAGTAAGATGCCATCCTATCAGTTAGTAAGTAGGGTAAGGGCCTACTTAGACGAAGACGGGTACGGGGAATGAGGGTTTGATTCCGGAGAGGGAGCCTGAGAGATGGCTACCAGGACCAAGGTCAGCAGCAGGCGCGAAAATTATCGAAGCCCGCC-TAGGGGCGATAGTGAGGAGACGTGTATAACGAAGTACGTGTAAAGAACGTACTAATAACTGGA-GGTCAAGTCTGGTGCCAGCATCCGCGGTAATTCCAGCTCCAGGGGTGTCTATGATGATTGCTGCGATTAAAAAGTCCGTAGTCAAACTGACTG-ACTGACCTGCAATG-TGATTGATTAAAGAACGAGCAGGGTTAGGAAAGCAGAGAATTAGGAGCGACCGAGGGCTAGAGTATTGAATGGCGAGAGGTGAAATTTGATGACCCATTCAGGAGTGACAAAGGCGAAGGCACTAGTCAAGGGCGAATCCGATGATCAAGGACGTAGGCTAGAGTTTCGAAAACGATTAGAGACCGGAGTAGTTCTAGCAGTAAACTATGCCGACGCCGTGATAT-TG---TTT----TTTGTGGCAGTATTGCGGAAGAGAAATCAAGT-AAAGCTTTGGGGAGAGTACGCGCGCAAGCGATAAATTTAAAGGAAATTGACGGAGGAACACCACAAGGAGTGGAGTGTGCGGCTTAATTTGACTCAACGCGGGACAGCTTACCAGGCCCGATAATCGAGCGAGCGTAGTACGCGATAGATTAAAGAGTGGTGCATGGCTGCTATCGACAGTTGGGGTGACC-TTAGGGTTAATTCCGGCAAGTAGTGAGACCCCTGCAGATAG--TGGACAGGTATTTTT-AAAATACAGGAAGGAAGGGACAAGAGCAGGTCAGTGATGCCCTTAGATGGCCTGGGCTGCACGCGCACTACAGTGGTCATTATAAGTAGAAATTAGA-AATAAAAATGATCGAGAGGGACTGAGCTTTGTAAGAGGCTCACGAACGAGGAATTGCTAGTAATCGTAGGCTCATTAAGATACGATGAATATGTCCCTGTACCTTGTACACACCGCCCGTCGTTATCGAAGATGGAATTGTGTGCGAACGAGCAACAAGCGAGTGAGCGCATAGTTCTAGATGTGATAAAAGTCGTAACAAGGCAACTGTAGGAGAACCTGTAGTTGGATCATACAGATTTA-TATT

>>AJ438955_IE_Gdub-ce_Dmue_TER-04_1322pb_AJ438955

-----------------------------GCATGTGTAAGCGAAGCTATATGTGGAGCGGTGAAAGGCTCAGTAACGGGCGATTTATTTAATCTCCTGGGACGGACAACATCGGGAAACTGATGGGAAAACGTCTAAGTTGCAT------TAATATTAGTTAGTGTGACGTAAA-CGG-TAATCGTGCAGGAGAGTAAGATGCCATCCTATCAGTTAGTAAGTAGGGTAAGGGCCTACTTAGACGAAGACGGGTACGGGGAATGAGGGTTTGATTCCGGAGAGGGAGCCTGAGAGATGGCTACCAGGACCAAGGTCAGCAGCAGGCGCGAAAATTATCGAAGCCCGCC-TAGGGGCGATAGTGAGGAGACGTGTATAACGAAGTACGTGTAAAGAACGTACTAATAACTGGA-GGTCAAGTCTGGTGCCAGCATCCGCGGTAATTCCAGCTCCAGGGGTGTCTATGATGATTGCTGCGATTAAAAAGTCCGTAGTCAAACTGACTG-ACTGACCTGCAATG-TGATTGATTAAAGAACGAGCAGGGTTAGGAAAGCAGAGAATTAGGAGCGACCGAGGGCTAGAGTATTGAATGGCGAGAGGTGAAATTTGATGACCCATTCAGGAGTGACAAAGGCGAAGGCACTAGTCAAGGGCGAATCCGATGATCAAGGACGTAGGCTAGAGTTTCGAAAACGATTAGAGACCGGAGTAGTTCTAGCAGTAAACTATGCCGACGCCGTGATAT-TG---TTT----TTTGTGGCAGTATTGCGGAAGAGAAATCAAGT-AAGGCTTTGGGGAGAGTACGCGCGCAAGCGATAAATTTAAAGGAAATTGACGGAGGAACACCACAAGGAGTGGAGTGTGCGGCTTAATTTGACTCAACGCGGGACAGCTTACCAGGCCCGATAATCGAGCGAGCGTAGTACGCGATAGATTAAAGAGTGGTGCATGGCTGCTATCGACAGTTGGGGTGACC-TTAGGGTTAATTCCGGCAAGTAGTGAGACCCCTGCAGATAG--TGGACAGGTATTTTT-AAAATACAGGAAGGAAGGGACAAGAGCAGGTCAGTGATGCCCTTAGATGGCCTGGGCTGCACGCGCACTACAGTGGTCATTATAAGTAGAAATTAGA-AGTAAAAATGATCGAGAGGGACTGAGCTTTGTAAGAGGCTCACGAACGAGGAATTGCTAGTAATCGTAGGCTCATTAAGATACGATGAATATGTCCCTGTACCTTGTACACACCGCCCGTCGTTATCGAAGATGGAATTGTGTGCGAACGAGCAACAAGCGAGTGAGCGCATAGTTCTAGATGTGATAAAAGTCGTAACAAGGCAACTGTAGGAGAACCTGTAGTTGGATCATAC------------

>>HQ681061_SE_Gdue_D-44_WIL-11_515pb_HQ681061

--------------------------------------------------------------------------------------------------------------------------------------------------------------------------------------------------------------------------------------------------------------------------------------------------------------CCAGGACCAAGGTCAGCAGCAGGCGCGAAAATTATCGAAGCCCGCC-TAGGGGCGATAGTGAGGAGACGTGTATAACGAAGTACGTGTAAAGAACGTACTAATAACTGGA-GGTCAAGTCTGGTGCCAGCATCCGCGGTAATTCCAGCTCCAGGGGTGTCTATGATGATTGCTGCGATTAAAAAGTCCGTAGTCAAACTGACTG-ACTGACCTGCAATG-TGATTGATTAAAGAACGAGCAGGGTTAGGAAAGCAGAGAATTAGGAGCGACCGAGGGCTAGAGTATTGAATGGCGAGAGGTGAAATTTGATGACCCATTCAGGAGTGACAAAGGCGAGGGCACTAGTCAAGGGCGAATCCGATGATCAAGGACGTAGGCTAGAGTTTCGAAAACGATTAGAGACCGGAGTAGTTCTAGCAGTAAACTATGCCGACGCCGTGACAT-TG---TTT----TTTGTGGCAGTATTGCGGAAGAGAAATCAAGT-AAGGCTTTGGGGAGAGTACGCGCGCAAGCGATAAATT-------------------------------------------------------------------------------------------------------------------------------------------------------------------------------------------------------------------------------------------------------------------------------------------------------------------------------------------------------------------------------------------------------------------------------------------------------------------------------------------------------------------------------------------------------------------------------

>>HQ683743_PL_Dhae_Dsp-30_WIL-11_547pb_HQ683743

----------------------------------------------------------------------------------------------------------------------------------------------------------------------------------------------------------------------------------------------------------------------------------------------------------GCTACCAGGACCAAGGTCAGCAGCAGGCGCGAAAATTATCGAAGCCCGCC-TAGGGGCGATAGTGAGGAGACGTGTATAACGAAGTACGTGTAAAGAACGTACTAATAACTGGA-GGTCAAGTCTGGTGCCAGCATCCGCGGTAATTCCAGCTCCAGGGGTGTCTATGATGATTGCTGCGATTAAAAAGTCCGTAGTCAAACTGACTG-ACTGACCTGCAATG-TGATTGATTAAAGAACGAGCAGGGTTAGGAAAGCAGAGAATTAGGAGCGACCGAGGGCTAGAGTATTGAATGGCGAGAGGTGAAATTTGATGACCCATTCAGGAGTGACAAAGGCGAAGGCACTAGTCAAGGGCGAATCCGATGATCAAGGACGTAGGCTAGAGTTTCGAAAACGATTAGAGACCGGAGTAGTTCTAGCAGTAAACTATGCCGACGCCGTGATAT-TG---TTT----TTTGTGGCAGTATTGCGGAAGAGAAATCAAGT-AAGGCTTTGGGGAGAGTACGCGCGCAAGCGATAAATTTAAAGGAAA-TGACGGAGGAACACCACAA--------------------------------------------------------------------------------------------------------------------------------------------------------------------------------------------------------------------------------------------------------------------------------------------------------------------------------------------------------------------------------------------------------------------------------------------------------------------------------------------------------------------------------------------------

>>HQ683744_PL_Dhae_Dsp-31_WIL-11_547pb_HQ683744

----------------------------------------------------------------------------------------------------------------------------------------------------------------------------------------------------------------------------------------------------------------------------------------------------------GCTACCAGGACCAAGGTCAGCAGCAGGCGCGAAAATTATCGAAGCCCGCC-TAGGGGCGATAGTGAGGAGACGTGTATAACGAAGTACGTGTAAAGCACGCACTAATAACTGGA-GGTCAAGTCTGGTGCCAGCATCCGCGGTAATTCCAGCTCCAGGGGTGTCTATGATGATTGCTGCGATTAAAAAGTCCGTAGTCAAACTGACTG-ACTGACCTGCAATG-TGATTGATTAAAGAACGAGCAGGGTTAGGAAAGCAGAGAATTAGGAGCGACCGAGGGCTAGAGTATTGAATGGCGAGAGGTGAAATTTGATGACCCATTCAGGAGTGACAAAGGCGAAGGCACTAGTCAAGGGCGAATCCGATGATCAAGGACGTAGGCTAGAGTTTCGAAAACGATTAGAGACCGGAGTAGTTCTAGCAGTAAACTATGCCGACGCCGTGATAT-TG---TTT----TTTGTGGCAGTATTGCGGAAGAGAAATCAAGT-AAGGCTTTGGGGAGAGTACGCGCGCAAGCGATAAATTTAAAGGAAA-TGACGGAGGAACACCACAA--------------------------------------------------------------------------------------------------------------------------------------------------------------------------------------------------------------------------------------------------------------------------------------------------------------------------------------------------------------------------------------------------------------------------------------------------------------------------------------------------------------------------------------------------

>>M11_ALPM41-1_DE_Groe_Dmue_KAR-18_1763pb_MG773234

-------------------------------------------AGCTATATGTGGAGCGGTGAAAGGCTCAGTAACGGGCGATTTATTTAATCTCCTGGGACGGACAACATCGGGAAACTGATGGGAAAACGTCTAAGTTGCAT------T----AATGTTAGTGTGACGTAAA-CGG-TAATCGTGCAGGAGAGTAAGATGCCATCCTATCAGTTAGTAAGTAGGGTAAGGGCCTACTTAGACGAAGACGGGTACGGGGAATGAGGGTTTGATTCCGGAGAGGGAGCCTGAGAGATGGCTACCAGGACCAAGGTCAGCAGCAGGCGCGAAAATTATCGAAGCCCGCC-TAGGGGCGATAGTGAGGAGACGTGTATAACGAAGTACGTGTAAAGAACGTACTAATAACTGGA-GGTCAAGTCTGGTGCCAGCATCCGCGGTAATTCCAGCTCCAGGGGTGTCTATGATGATTGCTGCGATTAAAAAGTCCGTAGTCAAACTGACTG-ACTGACCTGCAATG-TGATTGATTAAAGAACGAGCAGGGTTAGGAAAGCAGAGAATTAGGAGCGACCGAGGGCTAGAGTATTGAATGGCGAGAGGTGAAATTTGATGACCCATTCAGGAGTGACAAAGGCGAAGGCACTAGTCAAGGGCGAATCCGATGATCAAGGACGTAGGCTAGAGTTTCGAAAACGATTAGAGACCGGAGTAGTTCTAGCAGTAAACTATGCCGACGCCGTGATAT-TG---TTT----TTTGTGGCGGTATTGCGGAAGAGAAATCAAGT-AAAGCTTTGGGGAGAGTACGCGCGCAAGCGATAAATTTAAAGGAAATTGACGGAGGAACACCACAAGGAGTGGAGTGTGCGGCTTAATTTGACTCAACGCGGGACAGCTTACCAGGCCCGATAATCGAGCGAGCGTAGTACGCGATAGATTAAAGAGTGGTGCATGGCTGCTATCGACAGTTGGGGTGACC-TTAGGGTTAATTCCGGCAAGTAGTGAGACCCCTGCAGATAG--TGGACAGGTATTTTT-AAAATACAGGAAGGAAGGGACAAGAGCAGGTCAGTGATGCCCTTAGATGGCCTGGGCTGCACGCGCACTACAGTGGTCATTATAAGTAGAAATTAGA-AGTAAAAATGATCGAGAGGGACTGAGCTTTGTAAGAGGCTCACGAACGAGGAATTGCTAGTAATCGTAGGCTCATTAAGATACGATGAATATGTCCCTGTACCTTGTACACACCGCCCGTCGTTATCGAAGATGGAATTGTGTGCGAACGAGCAACAAGCGAGTGAGCGCATAGTTCTAGATGTGATAAAAGTCGTAACAAGGCAACTGTAGGAGAACCTGTAGTTGGATCATACAGATTTA-TATT

>>M12_ZWH16_PL_Dhae_Dmue_KAR-18_1763pb_MG773235*

-------------------------------------------AGCTATATGTGGAGCGGTGAAAGGCTCAGTAACGGGCGATTTATTTAATCTCCTGGGACGGACAACATCGGGAAACTGATGGGAAAACGTCTAAGTTGCAT------T----AATGTTAGTGTGACGTAAA-CGG-TAATCGTGCAGGAGAGTAAGATGCCATCCTATCAGTTAGTAAGTAGGGTAAGGGCCTACTTAGACGAAGACGGGTACGGGGAATGAGGGTTTGATTCCGGAGAGGGAGCCTGAGAGATGGCTACCAGGACCAAGGTCAGCAGCAGGCGCGAAAATTATCGAAGCCCGCC-TAGGGGCGATAGTGAGGAGACGTGTATAACGAAGTACGTGTAAAGAACGTACTAATAACTGGA-GGTCAAGTCTGGTGCCAGCATCCGCGGTAATTCCAGCTCCAGGGGTGTCTATGATGATTGCTGCGATTAAAAAGTCCGTAGTCAAACTGACTG-ACTGACCTGCAATG-TGATTGATTAAAGAACGAGCAGGGTTAGGAAAGCAGAGAATTAGGAGCGACCGAGGGCTAGAGTATTGAATGGCGAGAGGTGAAATTTGATGACCCATTCAGGAGTGACAAAGGCGAAGGCACTAGTCAAGGGCGAATCCGATGATCAAGGACGTAGGCTAGAGTTTCGAAAACGATTAGAGACCGGAGTAGTTCTAGCAGTAAACTATGCCGACGCCGTGATAT-TG---TTT----TTTGTGGCGGTATTGCGGAAGAGAAATCAAGT-AAGGCTTTGGGGAGAGTACGCGCGCAAGCGATAAATTTAAAGGAAATTGACGGAGGAACACCACAAGGAGTGGAGTGTGCGGCTTAATTTGACTCAACGCGGGACAGCTTACCAGGCCCGATAATCGAGCGAGCGTAGTACGCGATAGATTAAAGAGTGGTGCATGGCTGCTATCGACAGTTGGGGTGACC-TTAGGGTTAATTCCGGCAAGTAGTGAGACCCCTGCAGATAG--TGGACAGGTATTTTT-AAAATACAGGAAGGAAGGGACAAGAGCAGGTCAGTGATGCCCTTAGATGGCCTGGGCTGCACGCGCACTACAGTGGTCATTATAAGTAGAAATTAGA-AGTAAAAATGATCGAGAGGGACTGAGCTTTGTAAGAGGCTCACGAACGAGGAATTGCTAGTAATCGTAGGCTCATTAAGATACGATGAATATGTCCCTGTACCTTGTACACACCGCCCGTCGTTATCGAAGATGGAATTGTGTGCGAACGAGCAACAAGCGAGTGAGCGCATAGTTCTAGATGTGATAAAAGTCGTAACAAGGCAACTGTAGGAGAACCTGTAGTTGGATCATACAGATTTA-TATT

>>KR871380_DE_Gfos_Ddue_GRA-14_819pb_KR871380

-------------------------------------TAGCGAAGCTTTTAGTGGAGCGGTGAAAGGCTCAGTAACGGGCGATTTATTTGTTCTCCTGTGACGGACAACATCGGGAAACTGATGGGAAAACGTCTAAGTTGCAG------T----TAATTGATTGTGACGTAAA-CGA--TATTGTGCAGGAGAGTAAGATGCCATCCTATCAGTTAGTAAGTAGGGTAAGGGCCTACTTAGACGAAGACGGGTACGGGGAATGAGGGTTTGATTCCGGAGAGGGAGCCTGAGAGATGGCTACCAGGACCAAGGTCAGCAGCAGGCGCGAAAATTATCGAAGCCCGCC-TAGGGGCGATAGTGAGGAGACGTGTATAACGAAGTACGTGTAAAGAACGTGCTAATAACTGGA-GGTCAAGTCTGGTGCCAGCATCCGCGGTAATTCCAGCTCCAGGGGTGTCTATGATGATTGCTGCGATTAAAAAGTCCGTAGTCAATCTGACTG-ACTGGCCTGCAATG-TGATTGATCAAAGAACGAGCAGGGTTAGGAAAGCAGAGAATCAGGAGCGACCGAGGGCTAGAGTATTGAATGGCGAGAGGTGAAATTTGATGACCCATTCAGGAGTGACAAAGGCGAAGGCACTAGTCAAGGGCGAATCCGATGATCAAGGACGTAGGCTAGAGTTTCGAAAACGATTAGAGACCGGAGTAGTTCTAGCAGTAAACTATGCCGACGCCGTGGTAT-CG---ATT----T-------GGTATTGCGGAAGAGAAATCAAGT-AAGGCTTTGGGGAGAGTACGCGCGCAAGCGATAAATTTAAAGGAAATTGACGGAGGAACACCACAAGGAGTGGATTGTGCGGCTTAATTT-------------------------------------------------------------------------------------------------------------TGACTC-------------------------------------------------------------------------------------------------------------------------------------------------------------------------------------------------------------------------------------------------------------------------------------------------------------------------------------------------------------------------------------------------------

>>KR871355_DE_Gfos_Ddue_GRA-15_1090pb_KR871355

---------------------------------------GC-GAGCTTTTAGTGGAGCGGTGAAAGGCTCAGTAACGGGCGATTTATTTGTTCTCCTGTGACGGACAACATCGGGAAACTGATGGGAAAACGTCTAAGTTGCAG------T----TAATTGATTGTGACGTAAA-CGA--TATTGTGCAGGAGAGTAAGATGCCATCCTATCAGTTAGTAAGTAGGGTAAGGGCCTACTTAGACGAAGACGGGTACGGGGAATGAGGGTTTGATTCCGGAGAGGGAGCCTGAGAGATGGCTACCAGGACCAAGGTCAGCAGCAGGCGCGAAAATTATCGAAGCCCGCC-TAGGGGCGATAGTGAGGAGACGTGTATAACGAAGTACGTGTAAAGAACGTGCTAATAACTGGA-GGTCAAGTCTGGTGCCAGCATCCGCGGTAATTCCAGCTCCAGGGGTGTCTATGATGATTGCTGCGATTAAAAAGTCCGTAGTCAATCTGACTG-ACTGGCCTGCAATG-TGATTGATCAAAGAACGAGCAGGGTTAGGAAAGCAGAGAATCAGGAGCGACCGAGGGCTAGAGTATTGAATGGCGAGAGGTGAAATTTGATGACCCATTCAGGAGTGACAAAGGCGAAGGCACTAGTCAAGGGCGAATCCGATGATCAAGGACGTAGGCTAGAGTTTCGAAAACGATTAGAGACCGGAGTATTTCTAGCAGTAAACTATGCCGACGCCGTGGTAT-CG---ATT----T-------GGTATTGCGGAAGAGAAATCAAGT-AAGGCTTTGGGGAGAGTACGCGCGCAAGCGATAAATTTAAAGGAAATTGACGGAGGAACACCACAAGGAGTGGAGTGTGCGGCTTAATTTGACTCAACGCGGGACAGCTTACCAGGCCCGATAATCGAGCGAGCGTAGTACGCGATAGGTTAAAGAGTGGTGCATGGCTGCTATCGACAGTTGGGGTGACC-TTAGGGTTAATTCCGGCAAGTAGTGAGACCCCTGCAGATAG--TAGACAGGTATTTTT-AAAATACAGGAAGGAAGGGACAAGAGCAGGTCAGTGATGCCCTTAGATGGCCTGGGCTGCACGCGCACTACAGTGGTCATTATAAGTAGAAGTTAGA-TATAAAGATGATCGAAAGGGACTGAA-----------------------------------------------------------------------------------------------------------------------------------------------------------------------------------------------------------------------------

>>KR871356_DE_Gpul_Ddue_GRA-15_1052pb_KR871356

---------------------------------------------------GTGGAGCGGTGAAAGGCTCAGTAACGGGCGATTTATTTGTTCTCCTGTGACGGACAACATCGGGAAACTGATGGGAAAACGTCTAAGTTGCAG------T----TAATTGATTGTGACGTAAA-CGA--TATTGTGCAGGAGAGTAAGATGCCATCCTATCAGTTAGTAAGTAGGGTAAGGGCCTACTTAGACGAAGACGGGTACGGGGAATGAGGGTTTGATTCCGGAGAGGGAGCCTGAGAGATGGCTACCAGGACCAAGGTCAGCAGCAGGCGCGAAAATTATCGAAGCCCGCC-TAGGGGCGATAGTGAGGAGACGTGTATAACGAAGTACGTGTAAAGAACGTGCTAATAACTGGA-GGTCAAGTCTGGTGCCAGCATCCGCGGTAATTCCAGCTCCAGGGGTGTCTATGATGATTGCTGCGATTAAAAAGTCCGTAGTCAATCTGACTG-ACTGGCCTGCAATG-TGATTGATTAAAGAACGAGCAGGGTTAGGAAAGCAGAGAATCAGGAGCGACCGAGGGCTAGAGTATTGAATGGCGAGAGGTGAAATTTGATGACCCATTCAGGAGTGACAAAGGCGAAGGCACTAGTCAAGGGCGAATCCGATGATCAAGGACGTAGGCTAGAGTTTCGAAAACGATTAGAGACCGGAGTAGTTCTAGCAGTAAACTATGCCGACGCCGTGGTAT-CA---TTT----T-------GGTATTGCGGAAGAGAAATCAAGT-AAGGCTTTGGGGAGAGTACGCGCGCAAGCGATAAATTTAAAGGAAATTGACGGAGGAACACCACAAGGAGTGGAGTGTGCGGCTTAATTTGACTCAACGCGGGACAGCTTACCAGGCCCGATAATCGAGCGAGCGTAGTACGCGATAGGTTAAAGAGTGGTGCATGGCTGCTATCGACAGTTGGGGTGACC-TTAGGGTTAATTCCGGCAAGTAGTGAGACCCCTGCAGATAG--TGGACAGGTATTTTT-AAAATACAGGAAGGAAGGGACAAGAGCAGGTCAGTGATGCCCTTAGATGGCCTGGGCTGCACGCGCACTACAGTGGTCATTATAAGTAGAAGTTAG---------------------------------------------------------------------------------------------------------------------------------------------------------------------------------------------------------------------------------------------------------

>>KP091740_GB_Gpul_Ddue_IRO-15_1280pb_KP091740

GTGGACGCTAGTCTCACAGATTTAGCCATGCATGTGTAAGCGAAGCTTTTAGTGGAGCGGTGAAAGGCTCAGTAACGGGCGATTTATTTGTTCTCCTGTGACGGACAACATCGGGAAACTGATGGGAAAACGTCTAAGTTGCAG------T----TAATTGATTGTGACGTAAA-CGA--TATTGTGCAGGAGAGTAAGATGCCATCCTATCAGTTAGTAAGTAGGGTAAGGGCCTACTTAGACGAAGACGGGTACGGGGAATGAGGGTTTGATTCCGGAGAGGGAGCCTGAGAGATGGCTACCAGGACCAAGGTCAGCAGCAGGCGCGAAAATTATCGAAGCCCGCC-TAGGGGCGATAGTGAGGAGACGTGTATAACGAAGTACGTGTAAAGAACGTGCTAATAACTGGA-GGTCAAGTCTGGTGCCAGCATCCGCGGTAATTCCAGCTCCAGGGGTGTCTATGATGATTGCTGCGATTAAAAAGTCCGTAGTCAATCTGACTG-ACTGGCGTGCAATG-TGATTGATTAAAGAACGAGCAGGGTTAGGAAAGCAGAGAATCAGGAGCGACCGAGGGCTAGAGTATTGAATGGCGAGAGGTGAAATTTGATGACCCATTCAGGAGTGACAAAGGCGAAGGCACTAGTCAAGGGCGAATCCGATGATCAAGGACGTAGGCTAGAGTTTCGAAAACGATTAGAGACCGGAGTAGTTCTAGCAGTAAACTATGCCGACGCCGTGGTAT-CG---ATT----T-------GGTATTGCGGAAGAGAAATCAAGT-AAGGCTTTGGGGAGAGTACGCGCGCAAGCGATAAATTTAAAGGAAATTGACGGAGGAACACCACAAGGAGTGGAGTGTGCGGCTTAATTTGACTCAACGCGGGACAGCTTACCAGGCCCGATAATCGAGCGAGCGTAGTACGCGATAGGTTAAAGAGTGGTGCATGGCTGCTATCGACAGTTGGGGTGACC-TTAGGGTTAATTCCGGCAAGTAGTGAGACCCCTGCAGATAG--TGGACAGGTATTTTT-AAAATACAGGAAGGAAGGGACAAGAGCAGGTCAGTGATGCCCTTAGATGGCCTGGGCTGCACGCGCACTACAGTGGTCATTATAAGTAGAAGTTAGA-TATAAAGATGATCGAGAGGGACTGAGCTTTGTAAGAGGCTCACGAACGAGGAATTGCTAGTAATCGTAGGCTCATTAAGATACGATGAATATGTCCCTGTACCTTGTACACACCGCCCGTCGTTATCGAAGATGGAATTGTGTGCGAACGAGCAA--------------------------------------------------------------------------------------------

>>MG029379_PL_Gpul_Dsp_IRO-17_838pb_MG029379

GTGGACGCTAGTCTCACAGATTTAGCCATGCATGTGTAAGCGAAGCTTTTAGTGGAGCGGTGAAAGGCTCAGTAACGGGCGATTTATTTGTTCTCCTGTGACGGACAACATCGGGAAACTGATGGGAAAACGTCTAAGTTGCAG------T----TAATTGATTGTGACGTAAA-CGA--TATTGTGCAGGAGAGTAAGATGCCATCCTATCAGTTAGTAAGTAGGGTAAGGGCCTACTTAGACGAAGACGGGTACGGGGAATGAGGGTTTGATTCCGGAGAGGGAGCCTGAGAGATGGCTACCAGGACCAAGGTCAGCAGCAGGCGCGAAAATTATCGAAGCCCGCC-TAGGGGCGATAGTGAGGAGACGTGTATAACGAAGTACGTGTAAAGAACGTGCTAATAACTGGA-GGTCAAGTCTGGTGCCAGCATCCGCGGTAATTCCAGCTCCAGGGGTGTCTATGATGATTGCTGCGATTAAAAAGTCCGTAGTCAATCTGACTG-ACTGGCCTGCAATG-TGATTGATTAAAGAACGAGCAGGGTTAGGAAAGCAGAGAATCAGGAGCGACCGAGGGCTAGAGTATTGAATGGCGAGAGGTGAAATTTGATGACCCATTCAGGAGTGACAAAGGCGAAGGCACTAGTCAAGGGCGAATCCGATGATCAAGGACGTAGGCTAGAGTTTCGAAAACGATTAGAGACCGGAGTAGTTCTAGCAGTAAACTATGCCGACGCCGTGGTAT-CA---TTT----T-------GGTATTGCGGAAGAGAAATCAAGT-AAGGCTTTGGGGAGAGTACGCGCGCAAGCGATAAATTTAAAGGAAA-TGACGGAG-------------------------------------------------------------------------------------------------------------------------------------------------------------------------------------------------------------------------------------------------------------------------------------------------------------------------------------------------------------------------------------------------------------------------------------------------------------------------------------------------------------------------------------------------------------

>>MG029380_PL_Gpul_Dsp_IRO-17_767pb_MG029380

-----------------------------------------------TTTAGTGGAGCGGTGAAAGGCTCAGTAACGGGCGATTTATTTGTTCTCCTGTGACGGACAACATCGGGAAACTGATGGGAAAACGTCTAAGTTGCAG------T----TAATTGATTGTGACGTAAA-CGA--TATTGTGCAGGAGAGTAAGATGCCATCCTATCAGTTAGTAAGTAGGGTAAGGGCCTACTTAGACGAAGACGGGTACGGGGAATGAGGGTTTGATTCCGGAGAGGGAGCCTGAGAGATGGCTACCAGGACCAAGGTCAGCAGCAGGCGCGAAAATTATCGAAGCCCGCC-TAGGGGCGATAGTGAGGAGACGTGTATAACGAAGTACGTGTAAAGAACGTGCTAATAACTGGA-GGTCAAGTCTGGTGCCAGCATCCGCGGTAATTCCAGCTCCAGGGGTGTCTATGATGATTGCTGCGATTAAAAAGTCCGTAGTCAATCTGACTG-ACTGGCCTGCAATG-TGATTGATTAAAGAACGAGCAGGGTTAGGAAAGCAGAGAATTAGGAGCGACCGAGGGCTAGAGTATTGAATGGCGAGAGGTGAAATTTGATGACCCATTCAGGAGTGACAAAGGCGAAGGCACTAGTCAAGGGCGAATCCGATGATCAAGGACGTAGGCTAGAGTTTCGAAAACGATTAGAGACCGGAGTAGTTCTAGCAGTAAACTATGCCGACGCCGTGGTAT-CA---TTT----T-------GGTATTGCGGAAGAGAAATCAAGT-AAGGCTTTGGGGAGAGTACGCGCGCAAGCGATAAATTTAAAGGAAA-TGACGGAG-------------------------------------------------------------------------------------------------------------------------------------------------------------------------------------------------------------------------------------------------------------------------------------------------------------------------------------------------------------------------------------------------------------------------------------------------------------------------------------------------------------------------------------------------------------

>>HQ681048_GB_Gdue_D-15_WIL-11_508pb_HQ681048

--------------------------------------------------------------------------------------------------------------------------------------------------------------------------------------------------------------------------------------------------------------------------------------------------------------CCAGGACCAAGGTCAGCAGCAGGCGCGAAAATTATCGAAGCCCGCC-TAGGGGCGATAGTGAGGAGACGTGTATAACGAAGTACGTGTAAAGAACGTGCTAATAACTGGA-GGTCAAGTCTGGTGCCAGCATCCGCGGTAATTCCAGCTCCAGGGGTGTCTATGATGATTGCTGCGATTAAAAAGTCCGTAGTCAATCTGACTG-ACTGGCCTGCAATG-TGATTGATTAAAGAACGAGCAGGGTTAGGAAAGCAGAGAATTAGGAGCGACCGAGGGCTAGAGTATTGAATGGCGAGAGGTGAAATTTGATGACCCATTCAGGAGTGACAAAGGCGAAGGCACTAGTCAAGGGCGAATCCGATGATCAAGGACGTAGGCTAGAGTTTCGAAAACGATTAGAGACCGGAGTAGTTCTAGCAGTAAACTATGCCGACGCCGTGGTAT-CA---GAT----T-------GGTATTGCGGAAGAGAAATCAAGT-AAGGCTTTGGGGAGAGTACGCGCGCAAGCGATAAATT-------------------------------------------------------------------------------------------------------------------------------------------------------------------------------------------------------------------------------------------------------------------------------------------------------------------------------------------------------------------------------------------------------------------------------------------------------------------------------------------------------------------------------------------------------------------------------

>>JQ673483_GB_Echinogammarus_marinus_Ddue_YAN-11_1191pb_JQ673483

GTGGACGCTAGTCTCACAGATTTAGCCATGCATGTGTAAGCGAAGCTTTTAGTGGAGCGGTGAAAGGCTCAGTAACGGGCGATTTATTTGTTCTCCTGGGACGGACAACATCGGGAAACTGATGGGAAAACGTCTAAGTTGCAG------T----TAAATGATTGTGACGTAAACCAA--TTGTGTGCAGGAGAGTAAGATGCCATCCTATCAGTTAGTAAGTAGGGTAAGGGCCTACTTAGACGAAGACGGGTACGGGGAATGAGGGTTTGATTCCGGAGAGGGAGCCTGAGAGACGGCTACCAGGACCAAGGTCAGCAGCAGGCGCGAAAATTATCGAAGCCCGCC-TAGGGGCGATAGTGAGGAGACGTGTATAACGAAGTACGTGTAAAGAACGTGCTAATAACTGGA-GGTCAAGTCTGGTGCCAGCATCCGCGGTAATTCCAGCTCCAGGGGTGTCTATGATGACTGCTGCGATTAAAAAGTCCGTAGTCAATCTGACTG-ACTGGCCTGCAATG-TGATTGATTAAAGAACGAGCAGGGTTAGGAAAGCAGAGAATTAGGAGCGACCGAGGGCTAGAGTATTGAATGGCGAGAGGTGAAATTTGATGACCCATTCAGGAGTGACAAAGGCGAAGGCACTAGTCAAGGGCGAATCCGATGATCAAGGACGTAGGCTAGAGTTTCGAAAACGATTAGAGACCGGAGTAGTTCTAGCAGTAAACTATGCCGACGCCGTGGTAT-CA---GAT----T-------GGTATTGCGGAAGAGAAATCAAGT-AAGGCTTTGGGGAGAGTACGCGCGCAAGCGATAAATTTAAAGGAAATTGACGGAGGAACACCACAAGGAGTGGAGTGTGCGGCTTAATTTGACTCAACGCGGGACAGCTTACCAGGCCCGATAACCGAGCGAGCGTTGTACGCGATAGGTTAAAGAGTGGTGCATGGCTGCTATCGACAGTTGGGGTGACC-TTAGGGTTAATTCCGGCAAGTAGTGAGACCCCTGCAGATAG--TGGACAGGTATTTTT-AAAATACAGGAAGGAAGGGACAAGAGCAGGTCAGTGATGCCCTTAGATGGCCTGGGCTGCACGCGCACTACAGTGGTCATTATAAGTAGAAGTTAGA-TATAAAGATGATCGAGAGGGACTGAGCTTTGTAAGAGGCTCACGAACGAGGAATTGCTAGTAATCGTAGGCTC------------------------------------------------------------------------------------------------------------------------------------------------------------------------------

>>MG029378_PL_Gpul_Dsp_IRO-17_839pb_MG029378

GTGGACGCTAGTCTCACAGATTTAGCCATGCATGTGTAAGCGAAGCTTTTAGTGGAGCGGTGAAAGGCTCAGTAACGGGCGATTTATTTGTTCTCCTGTGACGGACAACATCGGGAAACTGATGGGAAAACGTCTAAGTTGCAG------T----TAATTGATTGTGACGTAAA-CGA--TATTGTGCAGGAGAGTAAGATGCCATCCTATCAGTTAGTAAGTAGGGTAAGGGCCTACTTAGACGAAGACGGGTACGGGGAATGAGGGTTTGATTCCGGAGAGGGAGCCTGAGAGATGGCTACCAGGACCAAGGTCAGCAGCAGGCGCGAAAATTATCGAAGCCCGCC-TAGGGGCGATAGTGAGGAGACGTGTATAACGAAGTACGTGTAAAGAACGTGCTAATAACTGGA-GGTCAAGTCTGGTGCCAGCATCCGCGGTAATTCCAGCTCCAGGGGTGTCTATGATGATTGCTGCGATTAAAAAGTCCGTAGTCAATCTGACTG-ACTGGCCTGCAATG-TGATTGATTAAAGAACGAGCAGGGTTAGGAAAGCAGAGAATCAGGAGCGACCGAGGGCTAGAGTATTGAATGGCGAGAGGTGAAATTTGATGACCCATTCAGGAGTGACAAAGGCGAAGGCACTAGTCAAGGGCGAATCCGATGATCAAGGACGTAGGCTAGAGTTTCGAAAACGATTAGAGACCGGAGTAGTTCTAGCAGTAAACTATGCCGACGCCGTGGTAT-CA---TTT----T-------GGTATTGCGGAAGAGAAATCAAGT-AAGGCTTTGGGGAGAGTACGCGCGCAAGCGATAAATTTAAAGGAAATTGACCGGAG------------------------------------------------------------------------------------------------------------------------------------------------------------------------------------------------------------------------------------------------------------------------------------------------------------------------------------------------------------------------------------------------------------------------------------------------------------------------------------------------------------------------------------------------------------

>>MG029381_PL_Gpul_Dsp_IRO-17_838pb_MG029381

GTGGGCGCTAGTCTCACAGATTTAGCCATGCATGTGTAAGCGAAGCTTTTAGTGGAGCGGTGAAAGGCTCAGTAGCGGGCGATTTATTTGTTCTCCTGTGACGGACAACATCGGGAAACTGATGGGAAAACGTCTAAGTTGCAG------T----TAATTGATTGTGACGTAAA-CGA--TATTGTGCAGGAGAGTAAGATGCCATCCTATCAGTTAGTAAGTAGGGTAAGGGCCTACTTAGACGAAGACGGGTACGGGGAATGAGGGTTTGATTCCGGAGAGGGAGCCTGAGAGATGGCTACCAGGACCAAGGTCAGCAGCAGGCGCGAAAATTATCGAAGCCCGCC-TAGGGGCGATAGTGAGGAGACGTGTATAACGAAGTACGTGTAAAGAACGTGCTAATAACTGGA-GGTCAAGTCTGGTGCCAGCATCCGCGGTAATTCCAGCTCCAGGGGTGTCTATGATGATTGCTGCGATTAAAAAGTCCGTAGTCAATCTGACTG-ACTGGCCTGCAATG-TGATTGATTAAAGAACGAGCAGGGTTAGGAAAGCAGAGAATCAGGAGCGACCGAGGGCTAGAGTATTGAATGGCGAGAGGTGAAATTTGATGACCCATTCAGGAGTGACAAAGGCGAAGGCACTAGTCAAGGGCGAATCCGATGATCAAGGACGTAGGCTAGAGTTTCGAAAACGATTAGAGACCGGAGTAGTTCTAGCAGTAAACTATGCCGACGCCGTGGTAT-CG---TTT----T-------GGTATTGCGGAAGAGAAATCAAGT-AAGGCTTTGGGGAGAGTACGCGCGCAAGCGATAAATT--AAAGGAAATGACGGAG-------------------------------------------------------------------------------------------------------------------------------------------------------------------------------------------------------------------------------------------------------------------------------------------------------------------------------------------------------------------------------------------------------------------------------------------------------------------------------------------------------------------------------------------------------------

>>KR871357_DE_Eber_Ddue_GRA-15_1053pb_KR871357

---------------------------------------------------GTGGAGCGGTATACGGCTCAGTAACGGGCGATTTATTTGTTCTCCTGGGACGGACAACATCGGGAAACTGATGGGAAAACGTCTAAGTTGCAG------T----TAATTGACTGTGACGTAAA-CCT--TAGTGTGCAGGAGAGTAAGATGCCATCCTATCAGTTAGTAAGTAGGGTAAGGGCCTACTTAGACGAAGACGGGTACGGGGAATGAGGGTTTGATTCCGGAGAGGGAGCCTGAGAGATGGCTACCAGGACCAAGGTCAGCAGCAGGCGCGAAAATTATCGAAGCCCGCC-TAGGGGCGATAGTGAGGAGACGTGTATAACGAAGTACGTGTAAAGAACGTGCTAATAACTGGA-GGTCAAGTCTGGTGCCAGCATCCGCGGTAATTCCAGCTCCAGGGGTGTCTATGATGATTGCTGCGATTAAAAAGTCCGTAGTCAATCTGACTG-ACTGACCTGCAATG-TGATTGATTAAAGAACGAGCAGGGTTAGGAAAGCAGAGAATTAGGAGCGACCGAGGGCTAGAGTATTGAATGGCGAGAGGTGAAATTTGATGACCCATTCAGGAGTGACAAAGGCGAAGGCACTAGTCAAGGGCGAATCCGATGATCAAGGACGTAGGCTAGAGTTTCGAAAACGATTAGAGACCGGAGTAGTTCTAGCAGTAAACTATGCCGACGCCGTGGTAT-CA--ACAA----T-------GGTATTGCGGAAGAGAAATCAAGT-AAGGCTTTGGGGAGAGTACGCGCGCAAGCGATAAATTTAAAGGAAATTGACGGAGGAACACCACAAGGAGTGGAGTGTGCGGCTTAATTTGACTCAACGCGGGACAGCTTACCAGGCCCGATAATCGAGCGAGCGTAGTACGCGATAGGTTAAAGAGTGGTGCATGGCTGCTATCGACAGTTGGGGTGACC-TTAGGGTTAATTCCGGCAAGTAGTGAGACCCCTGCAGATAG--TGGACAGGTATTTTT-AAAATACAGGAAGGAAGGGACAAGAGCAGGTCAGTGATGCCCTTAGATGGCCTGGGCTGCACGCGCACTACAGTGGTCATTATAAGTAGAAGTTAG---------------------------------------------------------------------------------------------------------------------------------------------------------------------------------------------------------------------------------------------------------

>>HQ681040_NO_Brandtia_latissima_Dsp-5-JEI-2011_WIL-11_509pb_HQ681040

--------------------------------------------------------------------------------------------------------------------------------------------------------------------------------------------------------------------------------------------------------------------------------------------------------------CCAGGACCAAGGTCAGCAGCAGGCGCGAAAATTATCGAAGCCCGCC-TAGGGGCGATAGTGAGGAGACGTGTATAACGAAGTACGTGTAAAGAACGTGCTAATAACTGGA-GGTCAAGTCTGGTGCCAGCATCCGCGGTAATTCCAGCTCCAGGGGTGTCTATGATGATTGCTGCGATTAAAAAGTCCGTAGTCAATCTGACTG-ACTGACCTGCAATG-TGATTGATTAAAGAACGAGCAGGGTTAGGAAAGCAGAGAATTAGGAGCGACCGAGGGCTAGAGTATTGAATGGCGAGAGGTGAAATTTGATGACCCATTCAGGAGTGACAAAGGCGAAGGCACTAGTCAAGGGCGAATCCGATGATCAAGGACGTAGGCTAGAGTTTCGAAAACGATTAGAGACCGGAGTAGTTCTAGCAGTAAACTATGCCGACGCCGTGGTAT-CA--AYAA----T-------GGTATTGCGGAAGAGAAATCAAGT-AAGGCTTTGGGGAGAGTACGCGCGCAAGCGATAAATT-------------------------------------------------------------------------------------------------------------------------------------------------------------------------------------------------------------------------------------------------------------------------------------------------------------------------------------------------------------------------------------------------------------------------------------------------------------------------------------------------------------------------------------------------------------------------------

>>KR871358_DE_Groe_Ddue_GRA-15_1053pb_KR871358

---------------------------------------------------GTGGAGCGGTATACGGCTCAGTAACGGGCGATTTATTTGTTCTCCTGGGACGGACAACATCGGGAAACTGATGGGAAAACGTCTAAGTTGCAG------T----TAATTGACTGTGACGTAAA-CCT--TTGTGTGCAGGAGAGTAAGATGCCATCCTATCAGTTAGTAAGTAGGGTAAGGGCCTACTTAGACGAAGACGGGTACGGGGAATGAGGGTTTGATTCCGGAGAGGGAGCCTGAGAGATGGCTACCAGGACCAAGGTCAGCAGCAGGCGCGAAAATTATCGAAGCCCGCC-TAGGGGCGATAGTGAGGAGACGTGTATAACGAAGTACGTGTAAAGAACGTGCTAATAACTGGA-GGTCAAGTCTGGTGCCAGCATCCGCGGTAATTCCAGCTCCAGGGGTGTCTATGATGATTGCTGCGATTAAAAAGTCCGTAGTCAATCTGACTG-ACTGACCTGCAATG-TGATTGATTAAAGAACGAGCAGGGTTAGGAAAGCAGAGAATTAGGAGCGACCGAGGGCTAGAGTATTGAATGGCGAGAGGTGAAATTTGATGACCCATTCAGGAGTGACAAAGGCGAAGGCACTAGTCAAGGGCGAATCCGATGATCAAGGACGTAGGCTAGAGTTTCGAAAACGATTAGAGACCGGAGTAGTTCTAGCAGTAAACTATGCCGACGCCGTGGTAT-CA--ACAA----T-------GGTATTGCGGAAGAGAAATCAAGT-AAGGCTTTGGGGAGAGTACGCGCGCAAGCGATAAATTTAAAGGAAATTGACGGAGGAACACCACAAGGAGTGGAGTGTGCGGCTTAATTTGACTCAACGCGGGACAGCTTACCAGGCCCGATAATCGAGCGAGCGTAGTACGCGATAGGTTAAAGAGTGGTGCATGGCTGCTATCGACAGTTGGGGTGACC-TTAGGGTTAATTCCGGCAAGTAGTGAGACCCCTGCAGATAG--TGGACAGGTATTTTT-AAAATACAGGAAGGAAGGGACAAGAGCAGGTCAGTGATGCCCTTAGATGGCCTGGGCTGCACGCGCACTACAGTGGTCATTATAAGTAGAAGTTAG---------------------------------------------------------------------------------------------------------------------------------------------------------------------------------------------------------------------------------------------------------

>>AF397404_GB_Gdue_Ddue_HOG-02_1323pb_AF397404

-----------------AGATTTAGCCATGCATGTGTAAGCGAAGCTTTTAGTGGAGCGGTATACGGCTCAGTAACGGGCGATTTATTTGTTCTCCTGGGACGGACAACATCGGGAAACTGATGGGAAAACGTCTAAGTTGCAG------T----TAATTGACTGTGACGTAAA-CCT--TCGTGTGCAGGAGAGTAAGATGCCATCCTATCAGTTAGTAAGTAGGGTAAGGGCCTACTTAGACGAAGACGGGTACGGGGAATGAGGGTTTGATTCCGGAGAGGGAGCCTGAGAGATGGCTACCAGGACCAAGGTCAGCAGCAGGCGCGAAAATTATCGAAGCCCGCC-TAGGGGCGATAGTGAGGAGACGTGTATAACGAAGTACGTGTAAAGAACGTGCTAATAACTGGA-GGTCAAGTCTGGTGCCAGCATCCGCGGTAATTCCAGCTCCAGGGGTGTCTATGATGATTGCTGCGATTAAAAAGTCCGTAGTCAATCTGACTG-ACTGACCTGCAATG-TGATTGATTAAAGAACGAGCAGGGTTAGGAAAGCAGAGAATTAGGAGCGACCGAGGGCTAGAGTATTGAATGGCGAGAGGTGAAATTTGATGACCCATTCAGGAGTGACAAAGGCGAAGGCACTAGTCAAGGGCGAATCCGATGATCAAGGACGTAGGCTAGAGTTTCGAAAACGATTAGAGACCGGAGTAGTTCTAGCAGTAAACTATGCCGACGCCGTGGTAT-CA--ACAA----T-------GGTATTGCGGAAGAGAAATCAAGT-AAGGCTTTGGGGAGAGTACGCGCGCAAGCGATAAATTTAAAGGAAATTGACGGAGGAACACCACAAGGAGTGGAGTGTGCGGCTTAATTTGACTCAACGCGGGACAGCTTACCAGGCCCGATAATCGAGCGAGCGTAGTACGCGATAGGTTAAAGAGTGGTGCATGGCTGCTATCGACAGTTGGGGTGACC-TTAGGGTTAATTCCGGCAAGTAGTGAGACCCCTGCAGATAG--TGGACAGGTATTTTT-AAAATACAGGAAGGAAGGGACAAGAGCAGGTCAGTGATGCCCTTAGATGGCCTGGGCTGCACGCGCACTACAGTGGTCATTATAAGTAGAAGTTAGA-TATAAAGATGATCGAGAGGGACTGAGCTTTGTAAGAGGCTCACGAACGAGGAATTGCTAGTAATCGTAGGCTCATTAAGATACGATGAATATGTCCCTGTACCTTGTACACACCGCCCGTCGTTATCGAAGATGGAATTGTGTGCGAACGAGCAACAAGCGAGTGAGCGCATAGTTCTAGATGTGATAAAAGTCGTAACAAGGCAACTGTAGGAGAACCTGTAGTTGGATCATAC------------

>>D2_UA29A-62_UH_Dvil_Ddue_KAR-18_1753pb_MG773213*

-------------------------------------------AGCTTTTAGTGGAGCGGTATACGGCTCAGTAACGGGCGATTTATTTGTTCTCCTGGGACGGACAACATCGGGAAACTGATGGGAAAACGTCTAAGTTGCAG------T----TAATTGATTGTGACGTAAA-CCT--TCGTGTGCAGGAGAGTAAGATGCCATCCTATCAGTTAGTAAGTAGGGTAAGGGCCTACTTAGACGAAGACGGGTACGGGGAATGAGGGTTTGATTCCGGAGAGGGAGCCTGAGAGATGGCTACCAGGACCAAGGTCAGCAGCAGGCGCGAAAATTATCGAAGCCCGCC-TAGGGGCGATAGTGAGGAGACGTGTATAACGAAGTACGTGTAAAGAACGTGCTAATAACTGGA-GGTCAAGTCTGGTGCCAGCATCCGCGGTAATTCCAGCTCCAGGGGTGTCTATGATGATTGCTGCGATTAAAAAGTCCGTAGTCAATCTGACTG-ACTGACCTGCAATG-TGATTGATTAAAGAACGAGCAGGGTTAGGAAAGCAGAGAATTAGGAGCGACCGAGGGCTAGAGTATTGAATGGCGAGAGGTGAAATTTGATGACCCATTCAGGAGTGACAAAGGCGAAGGCACTAGTCAAGGGCGAATCCGATGATCAAGGACGTAGGCTAGAGTTTCGAAAACGATTAGAGACCGGAGTAGTTCTAGCAGTAAACTATGCCGACGCCGTGGTAT-CA--ACAA----T-------GGTATTGCGGAAGAGAAATCAAGT-AAGGCTTTGGGGAGAGTACGCGCGCAAGCGATAAATTTAAAGGAAATTGACGGAGGAACACCACAAGGAGTGGAGTGTGCGGCTTAATTTGACTCAACGCGGGACAGCTTACCAGGCCCGATAATCGAGCGAGCGTAGTACGCGATAGGTTAAAGAGTGGTGCATGGCTGCTATCGACAGTTGGGGTGACC-TTAGGGTTAATTCCGGCAAGTAGTGAGACCCCTGCAGATAG--TGGACAGGTATTTTT-AAAATACAGGAAGGAAGGGACAAGAGCAGGTCAGTGATGCCCTTAGATGGCCTGGGCTGCACGCGCACTACAGTGGTCATTATAAGTAGAAGTTAGA-TATAAAGATGATCGAGAGGGACTGAGCTTTGTAAGAGGCTCACGAACGAGGAATTGCTAGTAATCGTAGGCTCATTAAGATACGATGAATATGTCCCTGTACCTTGTACACACCGCCCGTCGTTATCGAAGATGGAATTGTGTGCGAACGAGCAACAAGCGAGTGAGCGCATAGTTCTAGATGTGATAAAAGTCGTAACAAGGCAACTGTAGGAGAACCTGTAGTTGGATCATACAGATTTT-CTTA

>>FN434091_IS_Gdub-du_Ddue_KRE-10_1293pb_FN434091

GTGGACGCTAGTCTCACAGATTTAGCCATGCATGTGTAAGCGAAGCTTATAGTGGAGCGGTATACGGCTCAGTAACGGGCGATTTATTTGTTCTCCTGGGACGGACAACATCGGGAAACTGATGGGAAAACGTCTAAGTTGCAG------T----TAATTGACTGTGACGTAAA-CCT--T-GTGTGCAGGAGAGTAAGATGCCATCCTATCAGTTAGTAAGTAGGGTAAGGGCCTACTTAGACGAAGACGGGTACGGGGAATGAGGGTTTGATTCCGGAGAGGGAGCCTGAGAGATGGCTACCAGGACCAAGGTCAGCAGCAGGCGCGAAAATTATCGAAGCCCGCC-TAGGGGCGATAGTGAGGAGACGTGTATAACGAAGTACGTGTAAAGAACGTGCTAATAACTGGA-GGTCAAGTCTGGTGCCAGCATCCGCGGTAATTCCAGCTCCAGGGGTGTCTATGATGATTGCTGCGATTAAAAAGTCCGTAGTCAATCTGACTG-ACTGACCTGCAATG-TGATTGATTAAAGAACGAGCAGGGTTAGGAAAGCAGAGAATTAGGAGCGACCGAGGGCTAGAGTATTGAATGGCGAGAGGTGAAATTTGATGACCCATTCAGGAGTGACAAAGGCGAAGGCACTAGTCAAGGGCGAATCCGATGATCAAGGACGTAGGCTAGAGTTTCGAAAACGATTAGAGACCGGAGTAGTTCTAGCAGTAAACTATGCCGACGCCGTGGTAT-CA--ACAA----T-------GGTATTGCGGAAGAGAAATCAAGT-AAGGCTTTGGGGAGAGTACGCGCGCAAGCGATAAATTTAAAGGAAATTGACGGAGGAACACCACAAGGAGTGGAGTGTGCGGCTTAATTTGACTCAACGCGGGACAGCTTACCAGGCCCGATAATCGAGCGAGCGTAGTACGCGATAGGTTAAAGAGTGGTGCATGGCTGCTATCGACAGTTGGGGTGACC-TTAGGGTTAATTCCGGCAAGTAGTGAGACCCCTGCAGATAG--TGGACAGGTATTTTT-AAAATACAGGAAGGAAGGGACAAGAGCAGGTCAGTGATGCCCTTAGATGGCCTGGGCTGCACGCGCACTACAGTGGTCATTATAAGTAGAAGTTAGA-TATAAAGATGATCGAGAGGGACTGAGCTTTGTAAGAGGCTCACGAACGAGGAATTGCTAGTAATCGTAGGCTCATTAAGATACGATGAATATGTCCCTGTACCTTGTACACACCGCCCGTCGTTATCGAAGATGGAATTGTGTGCGAACGAGCAACAAGCGAGTGAGCGCATAGTTCTAGATGTGATAA----------------------------------------------------------

>>HQ681037_NO_Gdue_D-2_WIL-11_509pb_HQ681037

--------------------------------------------------------------------------------------------------------------------------------------------------------------------------------------------------------------------------------------------------------------------------------------------------------------CCAGGACCAAGGTCAGCAGCAGGCGCGAAAATTATCGAAGCCCGCC-TAGGGGCGATAGTGAGGAGACGTGTATAACGAAGTACGTGTAAAGGACGTGCTAATAACTGGA-GGTCAAGTCTGGTGCCAGCATCCGCGGTAATTCCAGCTCCAGGGGTGTCTATGATGATTGCTGCGATTAAAAAGTCCGTAGTCAATCTGACTG-ACTGACCTGCAATG-TGATTGATTAAAGAACGAGCAGGGTTAGGAAAGCAGAGAATTAGGAGCGACCGAGGGCTAGAGTATTGAATGGCGAGAGGTGAAATTTGATGACCCATTCAGGAGTGACAAAGGCGAAGGCACTAGTCAAGGGCGAATCCGATGATCAAGGACGTAGGCTAGAGTTTCGAAAACGATTAGAGACCGGAGTAGTTCTAGCAGTAAACTATGCCGACGCCGTGGTAT-CA--ACAA----T-------GGTATTGCGGAAGAGAAATCAAGT-AAGGCTTTGGGGAGAGTACGCGCGCAAGCGATAAATT-------------------------------------------------------------------------------------------------------------------------------------------------------------------------------------------------------------------------------------------------------------------------------------------------------------------------------------------------------------------------------------------------------------------------------------------------------------------------------------------------------------------------------------------------------------------------------

>>D3_DEB2_DE_Eberi_Ddue_KAR-18_1753pb_MG773214

-------------------------------------------AGCTTATAGTGGAGCGGTATACGGCTCAGTAACGGGCGATTTATTTGTTCTCCTGGGACGGACAACATCGGGAAACTGATGGGAAAACGTCTAAGTTGCAG------T----TAATTGACTGTGACGTAAA-CCT--TTGTGTGCAGGAGAGTAAGATGCCATCCTATCAGTTAGTAAGTAGGGTAAGGGCCTACTTAGACGAAGACGGGTACGGGGAATGAGGGTTTGATTCCGGAGAGGGAGCCTGAGAGATGGCTACCAGGACCAAGGTCAGCAGCAGGCGCGAAAATTATCGAAGCCCGCC-TAGGGGCGATAGTGAGGAGACGTGTATAACGAAGTACGTGTAAAGAACGTGCTAATAACTGGA-GGTCAAGTCTGGTGCCAGCATCCGCGGTAATTCCAGCTCCAGGGGTGTCTATGATGATTGCTGCGATTAAAAAGTCCGTAGTCAATCTGACTG-ACTGACCTGCAATG-TGATTGATTAAAGAACGAGCAGGGTTAGGAAAGCAAAGAATTAGGAGCGACCGAGGGCTAGAGTATTGAATGGCGAGAGGTGAAATTTGATGACCCATTCAGGAGTGACAAAGGCGAAGGCACTAGTCAAGGGCGAATCCGATGATCAAGGACGTAGGCTAAAGTTTCGAAAACGATTAGAGACCGGAGTAGTTCTAGCAGTAAACTATGCCGACGCCGTGGTAT-CA--ACAA----T-------GGTATTGCGGAAGAGAAATCAAGT-AAGGCTTTGGGGAGAGTACGCGCGCAAGCGATAAATTTAAAGGAAATTGACGGAGGAACACCACAAGGAGTGGAGTGTGCGGCTTAATTTGACTCAACGCGGGACAGCTTACCAGGCCCGATAATCGAGCGAGCGTAGTACGCGATAGGTTAAAGAGTGGTGCATGGCTGCTATCGACAGTTGGGGTGACC-TTAGGGTTAATTCCGGCAAGTAGTGAGACCCCTGCAGATAG--TGGACAGGTATTTTT-AAAATACAGGAAGGAAGGGACAAGAGCAGGTCAGTGATGCCCTTAGATGGCCTGGGCTGCACGCGCACTACAGTGGTCATTATAAGTAGAAGTTAGA-TATAAAGATGATCGAGAGGGACTGAGCTTTGTAAGAGGCTCACGAACGAGGAATTGCTAGTAATCGTAGGCTCATTAAGATACGATGAATATGTCCCTGTACCTTGTACACACCGCCCGTCGTTATCGAAGATGGAATTGTGTGCGAACGAGCAACAAGCGAGTGAGCGCATAGTTCTAGATGTGATAAAAGTCGTAACAAGGCAACTGTAGGAGAACCTGTAGTTGGATCATACAGATTTT-CTTA

>>MG029376_GB_Gdue_Dsp_IRO-17_930pb_MG029376

---------------------------------------------------------------------------------------------------------------------------------------------------------------------------------------------------------------------------------------------------------------------------------------------------------------------------------------------------------------GGGCCGATAGTGAGGAGACGTGTATAACGAAGTACGTGTAAAGAACGTGCTAATAACTGGA-GGTCAAGTCTGGTGCCAGCATCCGCGGTAATTCCAGCTCCAGGGGTGTCTATGATGATTGCTGCGATTAAAAAGTCCGTAGTCAATCTGACTG-ACTGACCTGCAATG-TGATTGATTAAAGAACGAGCAGGGTTAGGAAAGCAGAGAATTAGGAGCGACCGAGGGCTAGAGTATTGAATGGCGAGAGGTGAAATTTGATGACCCATTCAGGAGTGACAAAGGCGAAGGCACTAGTCAAGGGCGAATCCGATGATCAAGGACGTAGGCTAGAGTTTCGAAAACGATTAGAGACCGGAGTAGTTCTAGCAGTAAACTATGCCGACGCCGTGGTAT-CA--ACAA----T-------GGTATTGCGGAAGAGAAATCAAGT-AAGGCTTTGGGGAGAGTACGCGCGCAAGCGATAAATTTAAAGGAAATTGACGGAGGAACACCACAAGGAGTGGAGTGTGCGGCTTAATTTGACTCAACGCGGGACAGCTTACCAGGCCCGATAATCGAGCGAGCGTAGTACGCGATAGGTTAAAGAGTGGTGCATGGCTGCTATCGACAGTTGGGGTGACC-TTAGGGTTAATTCCGGCAAGTAGTGAGACCCCTGCAGATAG--TGGACAGGTATTTTT-AAAATACAGGAAGGAAGGGACAAGAGCAGGTCAGTGATGCCCTTAGATGGCCTGGGCTGCACGCGCACTACAGTGGTCATTATAAGTAGAAGTTAGA-TATAAAGATGATCGAGAGGGACTGAGCTTTGTAAGAGGCTCACGAACGAGGAATTGCTAGTAATCGTAGGCTCATTAAGATACGATGAATATGTCCCTGTACCTTGTACACACCGCCCGTCGTTATCGAAGATGGAATTGTGTGCGAACGAGCAACAAGCGA-------------------------------------------------------------------------------------

>>HQ681038_NO_Gdue_D-3_WIL-11_509pb_HQ681038

--------------------------------------------------------------------------------------------------------------------------------------------------------------------------------------------------------------------------------------------------------------------------------------------------------------CCAGGCCCAAGGTCAGCAGCAGGCGCGAAAATTATCGAAGCCCGCC-TAGGGGCGATAGTGAGGAGACGTGTATAACGAAGTACGTGTAAAGAACGTGCTAATAACTGGA-GGTCAAGTCTGGTGCCAGCATCCGCGGTAATTCCAGCTCCAGGGGTGTCTATGATGATTGCTGCGATTAAAAAGTCCGTAGTCAATCTGACTG-ACTGACCTGCAATG-TGATTGATTAAAGAACGAGCAGGGTTAGGAAAGCAGAGAATTAGGAGCGACCGAGGGCTAGAGTATTGAATGGCGAGAGGTGAAATTTGATGACCCATTCAGGAGTGACAAAGGCGAAGGCACTAGTCAAGGGCGAATCCGATGATCAAGGACGTAGGCTAGAGTTTCGAAAACGATTAGAGACCGGAGTAGTTCTAGCAGTAAACTATGCCGACGCCGTGGTAT-CA--ACAA----T-------GGTATTGCGGAAGAGAAATCAAGT-AAGGCTTTGGGGAGAGTACGCGCGCAAGCGATAAATT-------------------------------------------------------------------------------------------------------------------------------------------------------------------------------------------------------------------------------------------------------------------------------------------------------------------------------------------------------------------------------------------------------------------------------------------------------------------------------------------------------------------------------------------------------------------------------

>>HQ681039_NO_Gdue_D-4_WIL-11_509pb_HQ681039

--------------------------------------------------------------------------------------------------------------------------------------------------------------------------------------------------------------------------------------------------------------------------------------------------------------CCAGGACCAAGGTCAGCAGCAGGCGCGAAAATTATCGAAGCCCGCC-TAGGGGCGATAGTGAGGAGACGTGTATAACGAAGTACGTGTAAAGAACGTGCTAATAACTGGA-GGTCAAGTCTGGTGCCAGCATCCGCGGTAATTCCAGCTCCAGGGGTGTCTATGATGATTGCTGCGATTAAAAAGTCCGTAGTCAATCTGACTG-ACTGRCCTGCAATG-TGATTGATTAAAGAACGAGCAGGGTTAGGAAAGCAGAGAATTAGGAGCGACCGAGGGCTAGAGTATTGAATGGCKAGAGGTGAAATTYGATGACCCATTCAGGAGTGACAAAGGCGAAGGCACTAGTCAAGGGCGAATCCGATGATCAAGGACGTAGGCTAGAGTTTCGAAAACGATTAGAGACCGGAGTAGTTCTAGCAGTAAACTATGCCGACGCCGTGGTAT-CA--ACAA----T-------GGTATTGCGGAAGAGAAATCAAGT-GAGGCTTTGGGGAGAGTACGCGCGCAAGCGATAAATT-------------------------------------------------------------------------------------------------------------------------------------------------------------------------------------------------------------------------------------------------------------------------------------------------------------------------------------------------------------------------------------------------------------------------------------------------------------------------------------------------------------------------------------------------------------------------------

>>HM991451_US_Gpse_D-GPM1_RYA-10_1346pb_HM991451

GTGGACGCTAGTCTCACAGATTTAGCCATGCATGTGTAAGCGAAGCTTTTAGTGGAGCGGTGAAAGGCTCAGTAACGGGCGATTTATTTGTTCTCCTGGGACGGACAACATCGGGAAACTGATGGGAAAACGTCTAAGTTGCAG------T----TTATTGATTGTGACGTAAA-CCA--TAGTGTGCAGGAGAGTAAGATGCCATCCTATCAGTTAGTAAGTAGGGTAAGGGCCTACTTAGACGAAGACGGGTACGGGGAATGAGGGTTTGATTCCGGAGAGGGAGCCTGAGAGACGGCTACCAGGACCAAGGTCAGCAGCAGGCGCGAAAATTATCGAAGCCCGCC-TAGGGGCGATAGTGAGGAGACGTGTATAACGAAGTACGTGTAAAGAACGTGCTAATAACTGGA-GGTCAAGTCTGGTGCCAGCATCCGCGGTAATTCCAGCTCCAGGGGTGTCTATGATGATTGCTGCGATTAAAAAGTCCGTAGTCAATCTGACTG-ACTGACCTGTAATG-TGATTGATTAAAGAACGAACAGGGTTAGGAAAGCAGAGAATTAGGAGCGACCGAGGGCTAGAGTATTGAATGGCGAGAGGTGAAATTTGATGACCCATTCAGGAGTGACAAAGGCGAAGGCACTAGTCAAGGGCGAATCCGATGATCAAGGACGTAGGCTAGAGTTTCGAAAACGATTAGAGACCGGAGTAGTTCTAGCAGTAAACTATGCCGACGCCGTGGTAT-CA--AGTT----T-------GGTATTGCGGAAGAGAAATCAAGT-AAGGCTTTGGGGAGAGTACGCGCGCAAGCGATAAATTTAAAGGAAATTGACGGAGGAACACCACAAGGAGTGGAGTGTGCGGCTTAATTTGACTCAACGCGGGACAGCTTACCAGGCCCGATAACCGAGCGAGCGTAGTACGCGATAGGTTAAA-AGTGGTGCATGGCTGCTATCGACAGTTGGGGTGACC-TTAGGGT--AATCCGGCAAGTAATGAGA-CCCTGCAGATAG--TGGACAGGTATTT----AAATACAGGAAGGAAGGGACAAGAGCAGGTCAGTGATGCCCTTAGATGGCCTGGGCTGCACGCGCACTACAGTGGTCATTATAAGTAGAAGTTAGA-TATAAAGATGATCGAGAGGGACTGAGCTTTGTAAGAGGCTCACGAACGAGGAATTGCTAGTAATCGTAGGCTCATTAAGATACGATGAATATGTCCCTGTACCTTGTACACACCGCCCGTCGTTATCGAAGATGGAATTGTGTGCGAACGAGCAACAAGCGAGTGAGCGCATAGTTCTAGATGTGATAAAAGTCGTAACAAGGCAACTGTAGGAGAACCTGTAGTTGGATCATAC------------

>>HQ681042_SE_Gdue_D-8_WIL-11_509pb_HQ681042

--------------------------------------------------------------------------------------------------------------------------------------------------------------------------------------------------------------------------------------------------------------------------------------------------------------CCAGGACCAAGGTCAGCAGCAGGCGCGAAAATTATCGAAGCCCGCC-TAGGGGCGATAGTGAGGAGACGTGTATAACGAAGTACGTGTAAAGAACGTGCTAATAACTGGA-GGTCAAGTCTGGTGCCAGCATCCGCGGTAATTCCAGCTCCAGGGGTGTCTATGATGATTGCTGCGATTAAAAAGTCCGTAGTCAATCTGACTG-ACTGACCTGCAATG-TGATTGATTAAAGAACGAGCAGGGTTAGGAAAGCAGAGAATTAGGAGCGACCGAGGGCTAGAGTATTGAATGGCGAGAGGTGAAATTTGATGACCCATTCAGGAGTGACAAAGGCGAAGGCACTAGTCAAGGGCGAATCCGATGATCAAGGACGTAGGCTAGAGTTTCGAAAACGATTAGAGACCGGAGTAGTTCTAGCAGTAAACTATGCCGACGCCGTGGTAT-CA--TTTT----T-------GGTATTGCGGAAGAGAAATCAAGT-AAGGCTTTGGGGAGAGTACGCGCGCAAGCGATAAATT-------------------------------------------------------------------------------------------------------------------------------------------------------------------------------------------------------------------------------------------------------------------------------------------------------------------------------------------------------------------------------------------------------------------------------------------------------------------------------------------------------------------------------------------------------------------------------

>>HQ681041_IM_Gdue_D-7_WIL-11_509pb_HQ681041

--------------------------------------------------------------------------------------------------------------------------------------------------------------------------------------------------------------------------------------------------------------------------------------------------------------CCAGGACCAAGGTCAGCAGCAGGCGCGAAAATTATCGAAGCCCGCC-TAGGGGCGATAGTGAGGAGACGTGTATAACGAAGTACGTGTAAAGAACGTGCTAATAACTGGA-GGTCAAGTCTGGTGCCAGCATCCGCGGTAATTCCAGCTCCAGGGGTGTCTATGATGATTGCTGCGATTAAAAAGTCCGTAGTCAATCTGACTG-ACTGACCTGCAATG-TGATTGATTAAAGAACGAGCAGGGTTAGGAAAGCAGAGAATTAGGAGCGACCGAGGGCTAGAGTATTGAATGGCGAGAGGTCAAATTTGATGACCCATTCAGGAGTGACAAAGGCGAAGGCACTAGTCAAGGGCGAATCCGATGATCAAGGACGTAGGCTAGAGTTTCGAAAACGATTAGAGACCGGAGTAGTTCTAGCAGTAAACTATGCCGACGCCGTGGTAT-CA--ATTA----T-------GGTATTGCGGAAGAGAAATCAAGT-AAGGCTTTGGGGAGAGTACGCGCGCAAGCGATAAATT-------------------------------------------------------------------------------------------------------------------------------------------------------------------------------------------------------------------------------------------------------------------------------------------------------------------------------------------------------------------------------------------------------------------------------------------------------------------------------------------------------------------------------------------------------------------------------

>>M19_GvarM56_HU_Gvar_Dmue_KAR-18_1753pb_MG773241

-------------------------------------------AGCTATATGTGGAGCGGTGAAAGGCTCAGTAACGGGCGATTTATTTAATCTCCTGGGACGGACAACATCGGGAAACTGATGGGAAAACGTCTAAGTTGCAT------T----AATGTTAGTGTGACGTAAA-CGA--TATCGTGCAGGAGAGTAAGATGCCATCCTATCAGTTAGTAAGTAGGGTAAGGGCCTACTTAGACGAAGACGGGTACGGGGAATGAGGGTTTGATTCCGGAGAGGGAGCCTGAGAGATGGCTACCAGGACCAAGGTCAGCAGCAGGCGCGAAAATTATCGAAGCCCGCC-TAGGGGCGATAGTGAGGAGACGTGTATAACGAAGTACGTGTAAAGAACGTACTAATAACTGGA-GGTCAAGTCTGGTGCCAGCATCCGCGGTAATTCCAGCTCCAGGGGTGTCTATGATGATTGCTGCGATTAAAAAGTCCGTAGTCAAGCTGACTG-ACTGACCTGCAATG-TGATTGATTAAGGAACGAGCAGGGTTAGGAAAGCAGAGAATTAGGAGCGACCGAGGGCTAGAGTATTGAATGGCGAGAGGTGAAATTTGATGACCCATTCAGGAGTGACAAAGGCGAAGGCACTAGTCAAGGGCGAATCCGATGATCAAGGACGTAGGCTAGAGTTTCGAAAACGATTAGAGACCGGAGTAGTTCTAGCAGTAAACTATGCCGACGCCGTGATAT-AG--TTTG----T-------GGTATTGCGGAAGAGAAATCAAGT-AAGGCTTTGGGGAGAGTACGCGCGCAAGCGATAAATTTAAAGGAAATTGACGGAGGAACACCACAAGGAGTGGAGTGTGCGGCTTAATTTGACTCAACGCGGGACAGCTTACCAGGCCCGATAATCGAGCGAGCGTTGTACGCGATAGATTAAAGAGTGGTGCATGGCTGCTATCGACAGTTGGGGTGACC-TTAGGGTTAATTCCGGCAAGTAGTGAGACCCCTGCAGATAG--TGGACAGGTATTTTT-AAAATACAGGAAGGAAGGGACAAGAGCAGGTCAGTGATGCCCTTAGATGGCCTGGGCTGCACGCGCACTACAGTGGTCATTATAAGTAGAAGTTAGA-AGTAAAGATGATCGAGAGGGACTGAGCTTTGTAAGAGGCTCACGAACGAGGAATTGCTAGTAATCGTAGGCTCATTAAGATACGATGAATATGTCCCTGTACCTTGTACACACCGCCCGTCGTTATCGAAGATGGAATTGTGTGCGAACGAGCAACAAGCGAGTGAGCGCATAGTTCTAGATGTGATAAAAGTCGTAACAAGGCAACTGTAGGAGAACCTGTAGTTGGATCATACAGATTTA-ATTT

>>LOQ-05_FR_Groe_Droe-5_ADR-18_826pb_LOQ-05

------------------------------CATGTGTAAGCGAAGCGATAAGTGGAGCGGTGAAAGGCTCAGTAACGGGCGATTTATTTGATCTCCTGGGACGGACAACATCGGGAAACTGATGGGAAAACGTCTAAGTTGCAGTT---TT----TTTTTGATTGCGACGTAAA-CCT--TTTGGTGCAGGAGAGTAAGATGCCATCCTATCAGTTAGTAAGTAGGGTAAGGGCCTACTTAGACGAAGACGGGTACGGGGAATGAGGGTTTGATTCCGGAGAGGGAGCCTGAGAGATGGCTACCAGGACCAAGGTCAGCAGCAGGCGCGAAAATTATCGAAGCCCGCC-TAGGGGCGATAGTGAGGAGACGTGTATAACGAAGTACGTGTAAAGACCGTACTAATAACTGGA-GGTCAAGTCTGGTGCCAGCATCCGCGGTAATTCCAGCTCCAGGGGTGTCTATGATGATTGCTGCGATTAAAAAGTCCGTAGTCGAGCTGACTG-ACTGACCTGCAATG-TGGTTGATTAAAAGACGAGCAGGGTTAGGAAAGCAGAGAATTAGGAGCGACCGAGGGCTAGAGTATTGAATGGCGAGAGGTGAAATTTGATGACCCATTCAGGAGTGACAAAGGCGAAGGCACTAGTCAAGGGCGAATCCGATGATCAAGGACGTAGGCTAGAGTTTCGAAAACGATTAGAGACCGGAGTAGTTCTAGCAGTAAACTATGCCGACACCGTGGTAT-TAATTTTT----T-------AGTATTGCGGAAGAGAAATCAAGT-AAGGCTTTGGGGAGAGTACGCGCGCAAGCGATAAATTTAAAGGAAATTGACGGAGGAACACCACAAGGAGTGGAGTGTGCGGCTTAATTT--------------------------------------------------------------------------------------------------------------------------------------------------------------------------------------------------------------------------------------------------------------------------------------------------------------------------------------------------------------------------------------------------------------------------------------------------------------------------------------------------------------------------

>>LOQ-08_FR_Groe_Droe-4_ADR-18_801pb_LOQ-08

------------------------------CATGTGTAAGCGAAGCGATAAGTGGAGCGGTGAAAGGCTCAGTAACGGGCGATTTATTTGATCTCCTGGGACGGACAACATCGGGAAACTGATGGGAAAACGTCTAAGTTGCAGTT---TT----TTTTTGATTGCGACGTAAA-CCT--TTTGGTGCAGGAGAGTAAGATGCCATCCTATCAGTTAGTAAGTAGGGTAAGGGCCTACTTAGACGAAGACGGGTACGGGGAATGAGGGTTTGATTCCGGAGAGGGAGCCTGAGAGATGGCTACCAGGACCAAGGTCAGCAGCAGGCGCGAAAATTATCGAAGCCCGCC-TAGGGGCGATAGTGAGGAGACGTGTATAACGAAGTACGTGTAAAGACCGTACTAATAACTGGA-GGTCAAGTCTGGTGCCAGCAGCCGCGGTAATTCCAGCTCCAGGGGTGTCTATGATGATTGCTGCGATTAAAAAGTCCGTAGTCGAGCTGACTG-ACTGACCTGCAATG-TGGTTGATTAAAAGACGAGCAGGGTTAGGAAAGCAGAGAATTAGGAGCGACCGAGGGCTAGAGTATTGAATGGCGAGAGGTGAAATTTGATGACCCATTCAGGAGTGACAAAGGCGAAGGCACTAGTCAAGGGCGAATCCGATGATCAAGGACGTAGGCTAGAGTTTCGAAAACGATTAGAGACCGGAGTAGTTCTAGCAGTAAACTATGCCGACACCGTGGTAT-TAATTTTT----T-------AGTATTGCGGAAGAGAAATCAAGT-AAGGCTTTGGGGAGAGTACGCGCGCAAGCGATAAATTTAAAGGAAATTGACGGAGGAACACCACA---------------------------------------------------------------------------------------------------------------------------------------------------------------------------------------------------------------------------------------------------------------------------------------------------------------------------------------------------------------------------------------------------------------------------------------------------------------------------------------------------------------------------------------------------

>>LOQ-07_FR_Groe_Droe-1_ADR-18_818pb_LOQ-07

------------------------------CATGTGTAAGCGAAGCGATAAGTGGAGCGGTGAAAGGCTCAGTAACGGGCGATTTATTTGATCTCCTGGGACGGACAACATCGGGAAACTGATGGGAAAACGTCTAAGTTGCAGTT---TT----TTTTTGATTGCGACGTAAA-CCT--TTTGGTGCAGGAGAGTAAGATGCCCTCCTATCAGTTAGTAAGTAGGGTAGGGGCCTACTTAGACGAAGACGGGTACGGGGAATGAGGGTTTGATTCCGGAGAGGGAGCCTGAGAGATGGCTACCAGGACCAAGGTCAGCAGCAGGCGCGAAAATTATCGAACCCCGCC-TAGGGGCGATAGTGAGGAGACGTGTATAACGAAGTACGTGTAAAGACCGTACTAATAACTGGA-GGTCAAGTCTGGTGCCAGCATCCGCGGTAATTCCAGCTCCAGGGGTGTCTATGATGATTGCTGCGATTAAAAAGTCCGTAGTCGAGCTGACTG-ACTGACCTGCAATG-TGGTTGATTAAAAGACGAGCAGGGTTAGGAAAGCAGAGAATTAGGAGCGACCGAGGGCTAGAGTATTGAATGGCGAGAGGTGAAATTTGATGACCCATTCAGGAGTGACAAAGGCGAAGGCACTAGTCAAGGGCGAATCCGATGATCAAGGACGTAGGCTAGAGTTTCGAAAACGATTAGAGACCGGAGTAGTTCTAGCAGTAAACTATGCCGACACCGTGGTAT-TAATTTTT----T-------AGTATTGCGGAAGAGAAATCAAGT-AAGGCTTTGGGGAGAGTACGCGCGCAAGCGATAAATTTAAAGGAAATTGACGGAGGAACACCACAAGGAGTGGAGTGTGCGG----------------------------------------------------------------------------------------------------------------------------------------------------------------------------------------------------------------------------------------------------------------------------------------------------------------------------------------------------------------------------------------------------------------------------------------------------------------------------------------------------------------------------------

>>VOL5.4-07_FR_Groe_Droe-2_ADR-18_818pb_VOL5.4-07

------------------------------CATGTGTAAGCGAAGCGATAAGTGGAGCGGTGAAAGGCTCAGTAACGGGCGATTTATTTGATCTCCTGGGACGGACAACATCGGGAAACTGATGGGAAAACGTCTAAGTTGCAGTT---TT----TTTTTGATTGCGACGTAAA-CCT--TTTGGTGCAGGAGAGTAAGATGCCCTCCTATCAGTTAGTAAGTAGGGTAAGGGCCTACTTAGACGAAGACGGGTACGGGGAATGAGGGTTTGATTCCGGAGAGGGAGCCTGAGAGATGGCTACCAGGACCAAGGTCAGCAGCAGGCGCGAAAATTATCGAACCCCGCC-TAGGGGCGATAGTGAGGAGACGTGTATAACGAAGTACGTGTAAAGACCGTACTAATAACTGGA-GGTCAAGTCTGGTGCCAGCATCCGCGGTAATTCCAGCTCCAGGGGTGTCTATGATGATTGCTGCGATTAAAAAGTCCGTAGTCGAGCTGACTG-ACTGACCTGCAATG-TGGTTGATTAAAAGACGAGCAGGGTTAGGAAAGCAGAGAATTAGGAGCGACCGAGGGCTAGAGTATTGAATGGCGAGAGGTGAAATTTGATGACCCATTCAGGAGTGACAAAGGCGAAGGCACTAGTCAAGGGCGAATCCGATGATCAAGGACGTAGGCTAGAGTTTCGAAAACGATTAGAGACCGGAGTAGTTCTAGCAGTAAACTATGCCGACACCGTGGTAT-TAATTTTT----T-------AGTATTGCGGAAGAGAAATCAAGT-AAGGCTTTGGGGAGAGTACGCGCGCAAGCGATAAATTTAAAGGAAATTGACGGAGGAACACCACAAGGAGTGGAGTGTGCGG----------------------------------------------------------------------------------------------------------------------------------------------------------------------------------------------------------------------------------------------------------------------------------------------------------------------------------------------------------------------------------------------------------------------------------------------------------------------------------------------------------------------------------

>>MAR6-4-01_FR_Groe_Droe-3_ADR-18_823pb_MAR6-4-01

------------------------------CATGTGTAAGCGAAGCGATAAGTGGAGCGGTGAAAGGCTCAGTAACGGGCGATTTATTTGATCTCCTGGGACGGACAACATCGGGAAACTGATGGGAAAACGTCTAAGTTGCAGTT---TT----TTTTTGATTGCGACGTAAA-CCT--TTTGGTGCAGGAGAGTAAGATGCCATCCTATCAGTTAGTAAGTAGGGTAGGGGCCTACTTAGACGAAGACGGGTACGGGGAATGAGGGTTTGATTCCGGAGAGGGAGCCTGAGAGATGGCTACCAGGACCAAGGTCAGCAGCAGGCGCGAAAATTATCGAACCCCGCC-TAGGGGCGATAGTGAGGAGACGTGTATAACGAAGTACGTGTAAAGACCGTACTAATAACTGGA-GGTCAAGTCTGGTGCCAGCATCCGCGGTAATTCCAGCTCCAGGGGTGTCTATGATGATTGCTGCGATTAAAAAGTCCGTAGTCGAGCTGACTG-ACTGACCTGCAATG-TGGTTGATTAAAAGACGAGCAGGGTTAGGAAAGCAGAGAATTAGGAGCGACCGAGGGCTAGAGTATTGAATGGCGAGAGGTGAAATTTGATGACCCATTCAGGAGTGACAAAGGCGAAGGCACTAGTCAAGGGCGAATCCGATGATCAAGGACGTAGGCTAGAGTTTCGAAAACGATTAGAGACCGGAGTAGTTCTAGCAGTAAACTATGCCGACACCGTGGTAT-TAATTTTT----T-------AGTATTGCGGAAGAGAAATCAAGT-AAGGCTTTGGGGAGAGTACGCGCGCAAGCGATAAATTTAAAGGAAATTGACGGAGGAACACCACAAGGAGTGGAGTGTGCGGCTTAA-----------------------------------------------------------------------------------------------------------------------------------------------------------------------------------------------------------------------------------------------------------------------------------------------------------------------------------------------------------------------------------------------------------------------------------------------------------------------------------------------------------------------------

>>KR871360_DE_Groe_Ddue_GRA-15_548pb_KR871360

--------------------------------------------------------------------------------------------------------------------------------------------------------------------------------------------------GTAAGATGCCATCCTATCAGTTAGTAAGTAGGGTAAGGGCCTACTTAGACGAAGACGGGTACGGGGAATGAGGGTTTGATTCCGGAGAGGGAGCCTGAGAGATGGCTACCAGGACCAAGGTCAGCAGCAGGCGCGAAAATTATCGAAGCCCGCC-TAGGGGCGATAGTGAGGAGACGTGTATAACGAAGTACGTGTAAAGACCGTACTAATAACTGGA-GGTCAAGTCTGGTGCCAGCATCCGCGGTAATTCCAGCTCCAGGGGTGTCTATGATGATTGCTGCGATTAAAAAGTCCGTAGTCGAGCTGACTG-ACTGACCTGCAGTG-TGGTTGATTAAAAGACGAGCAGGGTTAGGAAAGCAGAGAATTAAGAGCGACCGAGGGCTAGAGTATTGAATGGCGAGAGGTGAAATTTGATGACCCATTCAAGAGTGACAAAGGCGAAGGCACTAGTCAAGGGCGAATCCGATGATCAAGGACGTACGCTAGAGTTTCGAAAACGATTAGAGACCGGAGTAGTTCTAGCAGTAAACTATGCCGACACCGTGGTA-------------------------------------------------------------------------------------------------------------------------------------------------------------------------------------------------------------------------------------------------------------------------------------------------------------------------------------------------------------------------------------------------------------------------------------------------------------------------------------------------------------------------------------------------------------------------------------------------------------------------------------------------------------------

>>GU196256_GB_Glac_D-GL_WIL-11_1250pb_GU196256

-------------------------------ATGTGTAAGCGAAGCGGTATGTGGAGCGGTGAAAGGCTCAGTAACGGGCGATTTATTTGATCTCCTGGGACGGACAACATCGGGAAACTGATGGGAAAACGTCTAAGTTGCAG------T----TTGTTGATTGTGACGTAAA-CCT--ATGTGTGCAGGAGAGTAAGATGCCATCCTATCAGTTAGTAAGTAGGGTAAGGGCCTACTTAGACGAAGACGGGTACGGGGAATGAGGGTTTGATTCCGGAGAGGGAGCCTGAGAGATGGCTACCAGGACCAAGGTCAGCAGCAGGCGCGAAAATTATCGAAGCCCGCC-TAGGGGCGATAGTGAGGAGACGTGTATAACGAAGTACGTGTAAAGACCGTACTAATAACTGGA-GGTCAAGTCTGGTGCCAGCATCCGCGGTAATTCCAGCTCCAGGGGTGTCTATGATGATTGCTGCGATTAAAAAGTCCGTAGTCGAGCTGACTG-ACTGACCTGCAATG-TGATTGATTAAAGAACGAGCAGGGTTAGGAAAGCAGAGAATTAGGAGCGACCGAGGGCTAGAGTATTGAATGGCGAGAGGTGAAATTTGATGACCCATTCAGGAGTGACAAAGGCGAAGGCACTAGTCAAGGGCGAATCCGATGATCAAGGACGTAGGCTAGAGTTTCGAAAACGATTAGAGACCGGAGTAGTTCTAGCAGTAAACTATGCCGACACCGTGGTAT-TAG-TTTTTAAAT-------AGTATTGCGGAAGAGAAATCAAGT-AAGGCTTTGGGGAGAGTACGCGCGCAAGCGATAAATTTAAAGGAAATTGACGGAGGAACACCACAAGGAGTGGAGTGTGCGGCTTAATTTGACTCAACGCGGGACAGCTTACCAGGCCCGATAATCATGCGAGCGTAGTACGCGATAGGTTAAAAAGTGGTGCATGGCTGCTATCGACCGTTGGGGTGACC-TTAGGGTTAATTCCGGCAAGTAGTGAGACCCCTGCAGATAG--TGGACAGGTATTTTT-AAGATACAGGAAGGAAGGGACAAGAGCAGGTCAGTGATGCCCTTAGATGGCCTGGGCTGCACGCGCACTACAGTGGTCATTATAAGTAGAAGTTAGA-TATAAAGATGATCGAGAGGGACTGAGCTTTGTAAGAGGCTCACGAACGAGGAATTGCTAGTAATCGTAGGCTCATTAAGATACGATGAATATGTCCCTGTACCTTGTACACACCGCCCGTCGTTATCGAAGATGGAATTGTGTGCGAACGAGCATTAAGCGAGTGAGCGCA----------------------------------------------------------------------------

>>KR871359_DE_Gpul_Ddue_GRA-15_1054pb_KR871359

---------------------------------------------------GTGGAGCGGTGAAAGGCTCAGTAACGGGCGATTTATTTGATCTCCTGGGACGGACAACATCGGGAAACTGATGGGAAAACGTCTAAGTTGCAG------T----TTATTGATTGTGACGTAAA-CCT--ATGTGTGCAGGAGAGTAAGATGCCATCCTATCAGTTAGTAAGTAGGGTAAGGGCCTACTTAGACGAAGACGGGTACGGGGAATGAGGGTTTGATTCCGGAGAGGGAGCCTGAGAGATGGCTACCAGGACCAAGGTCAGCAGCAGGCGCGAAAATTATCGAAGCCCGCC-CAGGGGCGATAGTGAGGAGACGTGTATAACGAAGTACGTGTAAAGACCGTACTAATAACTGGA-GGTCAAGTCTGGTGCCAGCATCCGCGGTAATTCCAGCTCCAGGGGTGTCTATGATGATTGCTGCGATTAAAAAGTCCGTAGTCGAGCTGACTG-ACTGACCTGCAATG-TGATTGATTAAAAGACGAGCAGGGTTAGGAAAGCAGAGAATTAGGAGCGACCGAGGGCTAGAGTATTGAATGGCGAGAGGTGAAATTTGATGACCCATTCAGGAGTGACAAAGGCGAAGGCACTAGTCAAGGGCGAATCCGATGATCAAGGACGTAGGCTAGAGTTTCGAAAACGATTAGAGACCGGAGTAGTTCTAGCAGTAAACTATGCCGACACCGTGGTAT-TAA-ATTT----T-------AGTATTGCGGAAGAGAAATCAAGT-AAGGCTTTGGGGAGAGTACGCGCGCAAGCGATAAATTTAAAGGAAATTGACGGAGGAACACCACAAGGAGTGGAGTGTGCGGCTTAATTTGACTCAACGCGGGACAGCTTACCAGGCCCGATAATCGAGCGAGCGTAGTACGCGATAGGTTAAAGAGTGGTGCATGGCTGCTATCGACAGTTGGGGTGACC-TTAGGGTTAATTCCGGCAAGTAGTGAGACCCCTGCAGATAG--TGGACAGGTATTTTT-AAGATACAGGAAGGAAGGGACAAGAGCAGGTCAGTGATGCCCTTAGATGGCCTGGGCTGCACGCGCACTACAGTGGTCATTATAAGTAGAAAGTAG---------------------------------------------------------------------------------------------------------------------------------------------------------------------------------------------------------------------------------------------------------

>>HQ681050_FR_Gdue_D-23_WIL-11_510pb_HQ681050

--------------------------------------------------------------------------------------------------------------------------------------------------------------------------------------------------------------------------------------------------------------------------------------------------------------CCAGGACCAAGGTCAGCAGCAGGCGCGAAAATTATCGAAGCCCGCC-TAGGGGCGATAGTGAGGAGACGTGTATAACGAAGTACGTGTAAAGACCGTACTAATAACTGGA-GGTCAAGTCTGGTGCCAGCATCCGCGGTAATTCCAGCTCCAGGGGTGTCTATGATGATTGCTGCGATTAAAAAGTCCGTAGTCGAGCTGACTG-ACTGACCTGCAATG-TGATTGATTAAAGAACGAGCAGGGTTAGGAAAGCAGAGGATTAGGAGCGACCGAGGGCTAGAGTATTGAATGGCGAGAGGTGAAATTTGATGACCCATTCAGGAGTGACAAAGGCGAAGGCACTAGTCAAGGGCGAATCCGATGATCAAGGACGTAGGCTAGAGTTTCGAAAACGATTAGAGACCGGAGTAGTTCTAGCAGTAAACTATGCCGACGCCGTGGTAT-TAA-ATAT----T-------AGTATTGCGGAAGAGAAATCAAGT-AAGGCTTTGGGGAGAGTACGCGCGCAAGCGATAAATT-------------------------------------------------------------------------------------------------------------------------------------------------------------------------------------------------------------------------------------------------------------------------------------------------------------------------------------------------------------------------------------------------------------------------------------------------------------------------------------------------------------------------------------------------------------------------------

>>GU196258_CZ_Gfos_D-GF_WIL-11_910pb_GU196258

------------------------------CATGTGTAAGCGAAGCGATATGTGGAGCGGTGAAAGGCTCAGTAACGGGCGATTTATTTGATCTCCTGGGACGGACAACATCGGGAAACTGATGGGAAAACGTCTAAGTTGCAG------T----TTTCTGATTGCGACGTAAA-CCA--TAGTGTGCAGGAGAGTAAGATGCCATCCTATCAGTTAGTAAGTAGGGTAAGGGCCTACTTAGACGAAGACGGGTACGGGGAATGAGGGTTTGATTCCGGAGAGGGAGCCTGAGAGATGGCTACCAGGACCAAGGTCAGCAGCAGGCGCGAAAATTATCGAAGCCCGCC-TAGGGGCGATAGTGAGGAGACGTGTATAACGAAGTACGTGTAAAGACCGTACTAATAACTGGA-GGTCAAGTCTGGTGCCAGCATCCGCGGTAATTCCAGCTCCAGGGGTGTCTATGATGATTGCTGCGATTAAAAAGTCCGTAGTCGAGCTGACTG-ACTGACCTGCAATG-TGATTGATTAAAGAACGAGCAGGGTTAGGAAAGCAGAGGATTAGGAGCGACCGAGGGCTAGAGTATTGAATGGCGAGAGGTGAAATTTGATGACCCATTCAGGAGTGACAAAGGCGAAGGCACTAGTCAAGGGCGAATCCGATGATCAAGGACGTAGGCTAGAGTTTCGAAAACGATTAGAGACCGGAGTAGTTCTAGCAGTAAACTATGCCGACGCCGTGGTAT-TA----TT----T-------AGTATTGCGGAAGAGAAATCAAGT-AAGGCTTTGGGGAGAGTACGCGCGCAAGCGATAAATTTAAAGGAAATTGACGGAGGAACACCACAAGGAGTGGAGTGTGCGGCTTAATTTGACTCAACGCGGGACAGCTTACCAGGCCCGATAATCGAGCGAGCGTAGTACGCGATAGGTTAAAAAGTGGTGCATGGCTGCTATCGACAGT-------------------------------------------------------------------------------------------------------------------------------------------------------------------------------------------------------------------------------------------------------------------------------------------------------------------------------------------------------------------------------------------------------------------------------

>>HQ681047_GB_Gdue_D-13_WIL-11_510pb_HQ681047

--------------------------------------------------------------------------------------------------------------------------------------------------------------------------------------------------------------------------------------------------------------------------------------------------------------CCAGGACCAAGGTCAGCAGCAGGCGCGAAAATTATCGAAGCCCGCC-TAGGGGCGATAGTGAGGAGACGTGTATAACGAAGTACGTGTAAAGACCGTACTAATAACTGGA-GGTCAAGTCTGGTGCCAGCATCCGCGGTAATTCCAGCTCCAGGGGTGTCTATGATGATTGCTGCGATTAAAAAGTCCGTAGTCAAGCTGACTG-ACTGACCTGCAATG-TGATTGATTAAAGAACGAGCAGGGTTAGGAAAGCAGAGAATTAGGAGCGACCGAGGGCTAGAGTATTGAATGGCGAGAGGTGAAATTTGATGACCCATTCAGGAGTGACAAAGGCGAAGGCACTAGTCAAGGGCGAATCCGATGATCAAGGACGTAGGCTAGAGTTTCGAAAACGATTAGAGACCGGAGTAGTTCTAGCAGTAAACTATGCCGACGCCGCGGTAT-CAT-TWTT----T-------GGTATTGCGGAAGAGAAATCAAGT-AAGGCTTTGGGGAGAGTACGCGCGCAAGCGATAAATT-------------------------------------------------------------------------------------------------------------------------------------------------------------------------------------------------------------------------------------------------------------------------------------------------------------------------------------------------------------------------------------------------------------------------------------------------------------------------------------------------------------------------------------------------------------------------------

>>R2_GvarM40_HU_Gvar_Droe_KAR-18_1758pb_MG773216

-------------------------------------------AGCGATATGTGGAGCGGTGAAAGGCTCAGTAACGGGCGATTTATTTGATCTCCTGGGACGGACAACATCGGGAAACTGATGGGAAAACGTCTAAGTTGCAG------T----TTTTTGACTGTGACGTAAA-CCG--TAGTGTGCAGGAGAGTAAGATGCCATCCTATCAGTTAGTAAGTAGGGTAAGGGCCTACTTAGACGAAGACGGGTACGGGGAATGAGGGTTTGATTCCGGAGAGGGAGCCTGAGAGATGGCTACCAGGACCAAGGTCAGCAGCAGGCGCGAAAATTATCGAAGCCCGCC-TAGGGGCGATAGTGAGGAGACGTGT-TAACGAAGTACGTGTAAAGACCGTACTAATAACTGGA-GGTCAAGTCTGGTGCCAGCATCCGCGGTAATTCCAGCTCCAGGGGTGTCTATGATGATTGCTGCGATTAAAAAGTCCGTAGTCGAGCTGACTG-ACTGACCTGCAATG-TGATTGATTAAAGAACGAGCAGGGTTAGGAAAGCAGAGGATTAGGAGCGACCGAGGGCTAGAGTATTGAATGGCGAGAGGTGAAATTTGATGACCCATTCAGGAGTGACAAAGGCGAAGGCACTAGTCAAGGGCGAATCCGATGATCAAGGACGTAGGCTAGAGTTTCGAAAACGATTAGAGACCGGAGTAGTTCTAGCAGTAAACTATGCCGACGCCGTGGTAT-TAG-GTAT----T-------AGTATTGCGGAAGAGAAATCAAGTAAAGGCTTTGGGGAGAGTACGCGCGCAAGCGATAAATTTAAAGGAAATTGACGGAGGAACACCACAAGGAGTGGAGTGTGCGGCTTAATTTGACTCAACGCGGGACAGCTTACCAGGCCCGATAATCGAGCGAGCGTAGTACGCGATAGGTTAAGAAGTGGTGCATGGCTGCTATCGACAGTTGGGGTGACC-TTAGGGTTAATTCCGGCAAGTAGTGAGACCCCTGCAGATAG--TGGACAGGTATTTTC-AAGATACAGGAAGGAAGGGACAAGAGCAGGTCAGTGATGCCCTTAGATGGCCTGGGCTGCACGCGCACTACAGTGGTCATTATAAGTAGAAGTTAGA-TATAAAGATGATCGAGAGGGACTGAGCTTTGTAAGAGGCTCACGAACGAGGAATTGCTAGTAATCGTAGGCTCATTAAGATACGATGAATATGTCCCTGTACCTTGTACACACCGCCCGTCGTTATCGAAGATGGAATTGTGTGCGAACAAGCATTAAGCGAGTGAGCGCATAGTTCTAGATGTGATAAAAGTCGTAACAAGGCAACTGTAGGAGAACCTGTAGTTGGATCATACAGATTGT-TTTT

>>R3_GvarM41_HU_Gvar_Droe_KAR-18_1756pb_MG773217

-------------------------------------------AGCGATATGTGGAGCGGTGAAAGGCTCAGTAACGGGCGATTTATTTGATCTCCTGGGACGGACAACATCGGGAAACTGATGGGAAAACGTCTAAGTTGCAG------T----TTTTTGACTGTGACGTAAA-CCG--TAGTGTGCAGGAGAGTAAGATGCCATCCTATCAGTTAGTAAGTAGGGTAAGGGCCTACTTAGACGAAGACGGGTACGGGGAATGAGGGTTTGATTCCGGAGAGGGAGCCTGAGAGATGGCTACCAGGACCAAGGTCAGCAGCAGGCGCGAAAATTATCGAAGCCCGCC-TAGGGGCGATAGTGAGGAGACGTGT-TAACGAAGTACGTGTAAAGACCGTACTAATAACTGGA-GGTCAAGTCTGGTGCCAGCATCCGCGGTAATTCCAGCTCCAGGGGTGTCTATGATGATTGCTGCGATTAAAAAGTCCGTAGTCGAGCTGACTG-ACTGACCTGCAATG-TGATTGATTAAAGAACGAGCAGGGTTAGGAAAGCAGAGGATTAGGAGCGACCGAGGGCTAGAGTATTGAATGGCGAGAGGTGAAATTTGATGACCCATTCAGGAGTGACAAAGGCGAAGGCACTAGTCAAGGGCGAATCCGATGATCAAGGACGTAGGCTAGAGTTTCGAAAACGATTAGAGACCGGAGTAGTTCTAGCAGTAAACTATGCCGACGCCGTGGTAT-TAG-GTAT----T-------AGTATTGCGGAAGAGAAATCAAGT-AAGGCTTTGGGGAGAGTACGCGCGCAAGCGATAAATTTAAAGGAAATTGACGGAGGAACACCACAAGGAGTGGAGTGTGCGGCTTAATTTGACTCAACGCGGGACAGCTTACCAGGCCCGAT-ATCGAGCGAGCGTAGTACGCGATAGGTTAAGAAGTGGTGCATGGCTGCTATCGACAGTTGGGGTGACC-TTAGGGTTAATTCCGGCAAGTAGTGAGACCCCTGCAGATAG--TGGACAGGTATTTTC-AAGATACAGGAAGGAAGGGACAAGAGCAGGTCAGTGATGCCCTTAGATGGCCTGGGCTGCACGCGCACTACAGTGGTCATTATAAGTAGAAGTTAGA-TATAAAGATGATCGAGAGGGACTGAGCTTTGTAAGAGGCTCACGAACGAGGAATTGCTAGTAATCGTAGGCTCATTAAGATACGATGAATATGTCCCTGTACCTTGTACACACCGCCCGTCGTTATCGAAGATGGAATTGTGTGCGAACAAGCATTAAGCGAGTGAGCGCATAGTTCTAGATGTGATAAAAGTCGTAACAAGGCAACTGTAGGAGAACCTGTAGTTGGATCATACAGATTGT-TTTT

>>R1_GFOS_GfOv_PL_Gfos_Droe_KAR-18_1751pb_MG773215

-------------------------------------------AGCGATATGTGGAGCGGTGAAAGGCTCAGTAACGGGCGATTTATTTGATCTCCTGGGACGGACAACATCGGGAAACTGATGGGAAAACGTCTAAGTTGCAG------T----TTTCTGATTGCGACGTAAA-CCA--TAGTGTGCAGGAGAGTAAGATGCCATCCTATCAGTTAGTAAGTAGGGTAAGGGCCTACTTAGACGAAGACGGGTACGGGGAATGAGGGTTTGATTCCGGAGAGGGAGCCTGAGAGATGGCTACCAGGACCAAGGTCAGCAGCAGGCGCGAAAATTATCGAAGCCCGCC-TAGGGGCGATAGTGAGGAGACGTGTAT-ACGAAGTACGTGTAAAGACCGTACTAATAACTGGA-GGTCAAGTCTGGTGCCAGCATCCGCGGTAATTCCAGCTCCAGGGGTGTCTATGATGATTGCTGCGATTAAAAAGTCCGTAGTCGAGCTGACTG-ACTGACCTGCAATG-TGATTGATTAAAGAACGAGCAGGGTTAGGAAAGCAGAGAATTAGGAGCGACCGAGGGCTAGAGTATTGAATGGCGAGAGGTGAAATTTGATGACCCATTCAGGAGTGACAAAGGCGAAGGCACTAGTCAAGGGCGAATCCGATGATCAAGGACGTAGGCTAGAGTTTCGAAAACGATTAGAGACCGGAGTAGTTCTAGCAGTAAACTATGCCGACGCCGTGGTAT-----TTTT----T-------AGTATTGCGGAAGAGAAATCAAGT-AAGGCTTTGGGGAGAGTACGCGCGCAAGCGATAAATTTAAAGGAAATTGACGGAGGAACACCACAAGGAGTGGAGTGTGCGGCTTAATTTGACTCAACGCGGGACAGCTTACCAGGCCCGATAATCGAGCGAGCGTAGTACGCGATAGGTTAAAAAGTGGTGCATGGCTGCTATCGACAGTTGGGGTGACC-TTAGGGTTAATTCCGGCAAGTAGTGAGACCCCTGCAGATAG--TGGACAGGTATTTTT-AAGATACAGGAAGGAAGGGACAAGAGCAGGTCAGTGATGCCCTTAGATGGCCTGGGCTGCACGCGCACTACAGTGGTCATTATAAGTAGAAGTTAGA-TATAAAGATGATCGAGAGGGACTGAGCTTTGTAAGAGGCTCACGAACGAGGAATTGCTAGTAATCGTAGGCTCATTAAGATACGATGAATATGTCCCTGTACCTTGTACACACCGCCCGTCGTTATCGAAGATGGAATTGTGTGCGAACGAGCATTAAGCGAGTGAGCGCATAGTTCTAGATGTGATAAAAGTCGTAACAAGGCAACTGTAGGAGAACCTGTAGTTGGATCATACAGATTGT-TTAT

>>R4_GbalM60_HU_Gbal_Droe_KAR-18_1755pb_MG773218*

-------------------------------------------AGCGATATGTGGAGCGGTGAAAGGCTCAGTAACGGGCGATTTATTTGATCTCCTGGGACGGACAACATCGGGAAACTGATGGGAAAACGTCTAAGTTGCAG------T----TAAATGATTGTGACGTAAA-CCA--TAGTGTGCAGGAGAGTAAGATGCCATCCTATCAGTTAGTAAGTAGGGTAAGGGCCTACTTAGACGAAGACGGGTACGGGGAATGAGGGTTTGATTCCGGAGAGGGAGCCTGAGAGATGGCTACCAGGACCAAGGTCAGCAGCAGGCGCGAAAATTATCGAAGCCCGCC-TAGGGGCGATAGTGAGGAGACGTGTAT-ACGAAGTACGTGTAAAGACCGTACTAATAACTGGA-GGTCAAGTCTGGTGCCAGCATCCGCGGTAATTCCAGCTCCAGGGGTGTCTATGATGATTGCTGCGATTAAAAAGTCCGTAGTCGAGCTGACTG-ACTGACCTGCAATG-TGATTGATTAAAGAACGAGCAGGGTTAGGAAAGCAGAGGATTAGGAGCGACCGAGGGCTAGAGTATTGAATGGCGAGAGGTGAAATTTGATGACCCATTCAGGAGTGACAAAGGCGAAGGCACTAGTCAAGGGCGAATCCGATGATCAAGGACGTAGGCTAGAGTTTCGAAAACGATTAGAGACCGGAGTAGTTCTAGCAGTAAACTATGCCGACGCCGTGGTAT-AG--GCTA----T-------AGTATTGCGGAAGAGAAATCAAGT-AAGGCTTTGGGGAGAGTACGCGCGCAAGCGATAAATTTAAAGGAAATTGACGGAGGAACACCACAAGGAGTGGAGTGTGCGGCTTAATTTGACTCAACGCGGGACAGCTTACCAGGCCCGATAATCGAGCGAGCGTAGTACGCGATAGGTTAAAAAGTGGTGCATGGCTGCTATCGACAGTTGGGGTGACC-TTAGGGTTAATTCCGGCAAGTAGTGAGACCCCTGCAGATAG--TGGACAGGTATTTTT-AAGATACAGGAAGGAAGGGACAAGAGCAGGTCAGTGATGCCCTTAGATGGCCTGGGCTGCACGCGCACTACAGTGGTCATTATAAGTAGAAGTTAGA-TATAAAGATGATCGAGAGGGACTGAGCTTTGTAAGAGGCTCACGAACGAGGAATTGCTAGTAATCGTAGGCTCATTAAGATACGATGAATATGTCCCTGTACCTTGTACACACCGCCCGTCGTTATCGAAGATGGAATTGTGTGCGAACGAGCATTAAGCGAGTGAGCGCATAGTTCTAGATGTGATAAAAGTCGTAACAAGGCAACTGTAGGAGAACCTGTAGTTGGATCATACAGATTGT-TTAT

>>R5_GRR24_FR_Groe_Droe_KAR-18_1759pb_MG773219*

-------------------------------------------AGCGATAAGTGGAGCGGTGAAAGGCTCAGTAACGGGCGATTTATTTGATCTCCTGGGACGGACAACATCGGGAAACTGATGGGAAAACGTCTAAGTTGCAGTT---TT----TTTTTGATTGCGACGTAAA-CCT--TTTGGTGCAGGAGAGTAAGATGCCATCCTATCAGTTAGTAAGTAGGGTAAGGGCCTACTTAGACGAAGACGGGTACGGGGAATGAGGGTTTGATTCCGGAGAGGGAGCCTGAGAGATGGCTACCAGGACCAAGGTCAGCAGCAGGCGCGAAAATTATCGAAGCCCGCC-TAGGGGCGATAGTGAGGAGACGTGTAT-ACGAAGTACGTGTAAAGACCGTACTAATAACTGGA-GGTCAAGTCTGGTGCCAGCATCCGCGGTAATTCCAGCTCCAGGGGTGTCTATGATGATTGCTGCGATTAAAAAGTCCGTAGTCGAGCTGACTG-ACTGACCTGCAATG-TGGTTGATTAAAAGACGAGCAGGGTTAGGAAAGCAGAGAATTAGGAGCGACCGAGGGCTAGAGTATTGAATGGCGAGAGGTGAAATTTGATGACCCATTCAGGAGTGACAAAGGCGAAGGCACTAGTCAAGGGCGAATCCGATGATCAAGGACGTAGGCTAGAGTTTCGAAAACGATTAGAGACCGGAGTAGTTCTAGCAGTAAACTATGCCGACACCGTGGTAT-TAATTTTT----T-------AGTATTGCGGAAGAGAAATCAAGT-AAGGCTTTGGGGAGAGTACGCGCGCAAGCGATAAATTTAAAGGAAATTGACGGAGGAACACCACAAGGAGTGGAGTGTGCGGCTTAATTTGACTCAACGCGGGACAGCTTACCAGGCCCGATAATCGAGCGAGCGTAGTACGCGATAGATTAAAAAGTGGTGCATGGCTGCTATCGACAGTTGGGGTGACC-TTAGGGTTAATTCCGGCAAGTAGTGAGACCCCTGCAGATAG--TGGACAGGTATTTTT-AAGATACAGGAAGGAAGGGACAAGAGCAGGTCAGTGATGCCCTTAGATGGCCTGGGCTGCACGCGCACTACAGTGGTCATTATAAGTAGAAATTAGA-TATAAAGATGATCGAGAGGGACTGAGCTTTGTAAGAGGCTCACGAACGAGGAATTGCTAGTAATCGTAGGCTCATTAAGATACGATGAATATGTCCCTGTACCTTGTACACACCGCCCGTCGTTATCGAAGATGGAATTGTGTGCGAACGAGCATTAAGCGAGTGAGCGCATAGTTCTAGATGTGATAAAAGTCGTAACAAGGCAACTGTAGGAGAACCTGTAGTTGGATCATACAGATTGT-TTAA

>>R6_GbOv2_PL_Gbal_Droe_KAR-18_1755pb_MG773220

-------------------------------------------AGCGATAAGTGGAGCGGTGAAAGGCTCAGTAACGGGCGATTTATTTGATCTCCTGGGACGGACAACATCGGGAAACTGATGGGAAAACGTCTAAGTTGCAG------T----TGTTTTATTGTGACGTAAA-CCT--TTGTGTGCAGGAGAGTAAGATGCCATCCTATCAGTTAGTAAGTAGGGTAAGGGCCTACTTAGACGAAGACGGGTACGGGGAATGAGGGTTTGATTCCGGAGAGGGAGCCTGAGAGATGGCTACCAGGACCAAGGTCAGCAGCAGGCGCGAAAATTATCGAAGCCCGCC-TAGGGGCGATAGTGAGGAGACGTGTAT-ACGAAGTACGTGTAAAGACCGTACTAATAACTGGA-GGTCAAGTCTGGTGCCAGCATCCGCGGTAATTCCAGCTCCAGGGGTGTCTATGATGATTGCTGCGATTAAAAAGTCCGTAGTCGAGCTGACTG-ACTGACCTGCAATG-TGATTGATTAAAGAACGAGCAGGGTTAGGAAAGCAGAGGATTAGGAGCGACCGAGGGCTAGAGTATTGAATGGCGAGAGGTGAAATTTGATGACCCATTCAGGAGTGACAAAGGCGAAGGCACTAGTCAAGGGCGAATCCGATGATCAAGGACGTAGGCTAGAGTTTCGAAAACGATTAGAGACCGGAGTAGTTCTAGCAGTAAACTATGCCGACACCGTGGTAT-TA--ATTT----T-------AGTATTGCGGAAGAGAAATCAAGT-AAGGCTTTGGGGAGAGTACGCGCGCAAGCGATAAATTTAAAGGAAATTGACGGAGGAACACCACAAGGAGTGGAGTGTGCGGCTTAATTTGACTCAACGCGGGACAGCTTACCAGGCCCGATAATCGAGCGAGCGTAGTACGCGATAGGTTAAAAAGTGGTGCATGGCTGCTATCGACAGTTGGGGTGACC-TTAGGGTTAATTCCGGCAAGTAGTGAGACCCCTGCAGATAG--TGGACAGGTATTTTT-AAGATACAGGAAGGAAGGGACAAGAGCAGGTCAGTGATGCCCTTAGATGGCCTGGGCTGCACGCGCACTACAGTGGTCATTATAAGTAGAAGTTAGA-TATAAAGATGATCGAGAGGGACTGAGCTTTGTAAGAGGCTCACGAACGAGGAATTGCTAGTAATCGTAGGCTCATTAAGATACGATGAATATGTCCCTGTACCTTGTACACACCGCCCGTCGTTATCGAAGATGGAATTGTGTGCGAACGAGCATTAAGCGAGTGAGCGCATAGTTCTAGATGTGATAAAAGTCGTAACAAGGCAACTGTAGGAGAACCTGTAGTTGGATCATACAGATTGT-TTAT

>>R7_GbOv3_PL_Gbal_Droe_KAR-18_1755pb_MG773221

-------------------------------------------AGCGATAAGTGGAGCGGTGAAAGGCTCAGTAACGGGCGATTTATTTGATCTCCTGGGACGGACAACATCGGGAAACTGATGGGAAAACGTCTAAGTTGCAG------T----AAATTTATTGTGACGTAAA-CCT--TTGTGTGCAGGAGAGTAAGATGCCATCCTATCAGTTAGTAAGTAGGGTAAGGGCCTACTTAGACGAAGACGGGTACGGGGAATGAGGGTTTGATTCCGGAGAGGGAGCCTGAGAGATGGCTACCAGGACCAAGGTCAGCAGCAGGCGCGAAAATTATCGAAGCCCGCC-TAGGGGCGATAGTGAGGAGACGTGTAT-ACGAAGTACGTGTAAAGACCGTACTAATAACTGGA-GGTCAAGTCTGGTGCCAGCATCCGCGGTAATTCCAGCTCCAGGGGTGTCTATGATGATTGCTGCGATTAAAAAGTCCGTAGTCGAGCTGACTG-ACTGACCTGCAATG-TGATTGATTAAAGAACGAGCAGGGTTAGGAAAGCAGAGGATTAGGAGCGACCGAGGGCTAGAGTATTGAATGGCGAGAGGTGAAATTTGATGACCCATTCAGGAGTGACAAAGGCGAAGGCACTAGTCAAGGGCGAATCCGATGATCAAGGACGTAGGCTAGAGTTTCGAAAACGATTAGAGACCGGAGTAGTTCTAGCAGTAAACTATGCCGACACCGTGGTAT-TA--ATTT----T-------AGTATTGCGGAAGAGAAATCAAGT-AAGGCTTTGGGGAGAGTACGCGCGCAAGCGATAAATTTAAAGGAAATTGACGGAGGAACACCACAAGGAGTGGAGTGTGCGGCTTAATTTGACTCAACGCGGGACAGCTTACCAGGCCCGATAATCGAGCGAGCGTAGTACGCGATAGGTTAAAAAGTGGTGCATGGCTGCTATCGACAGTTGGGGTGACC-TTAGGGTTAATTCCGGCAAGTAGTGAGACCCCTGCAGATAG--TGGACAGGTATTTTT-AAGATACAGGAAGGAAGGGACAAGAGCAGGTCAGTGATGCCCTTAGATGGCCTGGGCTGCACGCGCACTACAGTGGTCATTATAAGTAGAAGTTAGA-TATAAAGATGATCGAGAGGGACTGAGCTTTGTAAGAGGCTCACGAACGAGGAATTGCTAGTAATCGTAGGCTCATTAAGATACGATGAATATGTCCCTGTACCTTGTACACACCGCCCGTCGTTATCGAAGATGGAATTGTGTGCGAACGAGCATTAAGCGAGTGAGCGCATAGTTCTAGATGTGATAAAAGTCGTAACAAGGCAACTGTAGGAGAACCTGTAGTTGGATCATACAGATTGT-TTAT

>>MF403132_PL_Gpul_Dsp_BOJ-UN_684pb_MF403132

-------------------------------------------------------------------------------------------------------------------------------------------------------------------------------------------------------------------------------------------------------------------------------------------------------------------------------------------------------------------------------------------------CGTGTAAAGAACGTACTAATAACTGGA-GGTCAAGTCTGGTGCCAGCATCCGCGGTAATTCCAGCTCCAGGGGTGTCTATGATGATTGCTGCGATTAAAAAGTCCGTAGTCAAGCTGACTGAACTGACCTGCAATG-TGATTGATTAAGGAACGAGCAGGGTTAGGAAAGCAGAGAATTAGGAGCGACCGAGGGCTAGAGTATTGAATGGCGAGAGGTGAAATTTGATGACCCATTCAGGAGTGACAAAGGCGAAGGCACTAGTCAAGGGCGAATCCGATGATCAAGGACGTAGGCTAGAGTTTCGAAAACGATTAGAGACCGGAGTAGTTCTAGCAGTAAACTATGCCGACGCCGCGATAT-AG---TTT----TTTGTGGCTGTGTTGCGGAAGAGAAATCAAGT-AAGGCTTTGGGGAGAGTACGCGCGCAAGCGATAAATTTAAAGGAAATTGACGGAGGAACACCACAAGGAGTGGAGTGTGCGGCTTAATTTGACTCAACGCGGGACAGCTTACCAGGCCCGATAATCGAGCGAGCGTTGTACGCGATAGATTAAAGAGTGGTGCATGGCTGCTATCGACAGTTGGGGTGACC-TTAGGGTTAATTCCGGCAAGTAGTGAGACCCCTGCAGATAG--TGGACAGGTATTTTT-AAAATACAGGAAGGAAGGGACAAGAGCAGGTCAGTGATGC-----------------------------------------------------------------------------------------------------------------------------------------------------------------------------------------------------------------------------------------------------------------------------------------------------------------

>>MG029377_GB_Gdue_Dsp_IRO-17_880pb_MG029377

-----------------------------------------------------------------------------------------------------------------------------------------------------------------------------------------------------------------------------------------------------------------------------------------------------------------------------------------------------------------------------------------------------------------TAATAACTGGAGGGTCAAGTCTGGTGCCAGCATCCGCGGTAATTCCAGCTCCAGGGGTGTCTATGATGATTGCTGCGATTAAAAAGTCCGTAGTCAATCTGACTG-ACTGA-CTGCAATG-TGATTGATTAAAGAACGAGCAGGGTTAGGAAAGCAGAGAATTAGGAGCGACCGAGGGCTAGAGTATTGAATGGCGAGAGGTGAAATTTGATGACCCATTCAGGAGTGACAAAGGCGAAGGCACTAGTCAAGGGCGAATCCGATGATCAAGGACGTAGGCTAGAGTTTCGAAAACGATTAGAGACCGGAGTAGTTCTAGCAGTAAACTATGCCGACGCCGTGGTAT-CA--ACAA----T-------GGTATTGCGGAAGAGAAATCAAGT-AAGGCTTTGGGGAGAGTACGCGCGCAAGCGATAAATTTAAAGGAAATTGACGGAGGAACACCACAAGGAGTGGAGTGTGCGGCTTAATTTGACTCAACGCGGGACAGCTTACCAGGCCCGATAATCGAGCGAGCGTAGTACGCGATAGGTTAAAGAGTGGTGCATGGCTGCTATCGACAGTTGGGGTGACC-TTAGGGTTAATTCCGGCAAGTAGTGAGACCCCTGCAGATAG--TGGACAGGTATTTTT-AAAATACAGGAAGGAAGGGACAAGAGCAGGTCAGTGATGCCCTTAGATGGCCTGGGCTGCACGCGCACTACAGTGGTCATTATAAGTAGAAGTTAGA-TATAAAGATGATCGAGAGGGACTGAGCTTTGTAAGAGGCTCACGAACGAGGAATTGCTAGTAATCGTAGGCTCATTAAGATACGATGAATATGTCCCTGTACCTTGTACACACCGCCCGTCGTTATCGAAGATGGAATTGTGTGCGAACGAGCAACAAGCGA-------------------------------------------------------------------------------------

>>ALP43-01_DE_Groe_Dsp._N4_ADR-18_797pb_ALP43-01

---------------------------------------------------GTGGAGCGGTGAAAGGCTCAGTAACGGGCGATTTATTTGATCTCCTGGGACGGACAACATCGGGAAACTGATGGGAAAACGTCTAAGTTGCAG------T----TTTGTGATTGTGACGTAAA-CCA---TAGGTGCAGGAGAGTAAGATGCCATCCTATCAGTTAGTAAGTAGGGTAAGTGCCTACTTAGACGAAGACGGGTACGGGGAATGAGGGTTTGATTCCGGAGAGGGAGCCTGAGAGATGGCTACCAGGACCAAGGTCAGCAGCAGGCGCGAAAATTATCGAAGCCCGCC-GAGGGGCGATAGTGAGGAGACGTGTATAACGAAGTACGTGTAAAGAACGTGCTAATAACTGGA-GGTCAAGTCTGGTGCCAGCATCCGCGGTAATTCCAGCTCCAGGGGTGTCTATGATGATTGCTGCGATTAAAAAGTCCGTAGTCAATCTGACTG-ACTGGCCTGCAATG-TGATTGATTAAAGAACGAGCAGGGTTAGGAAAGCAGAGAATTAGGAGCGACCGAGGGCTAGAGTATTGAATGGCGAGAGGTGAAATTTGATGACCCATTCAGGAGTGACAAAGGCGAAGGCACTAGTCAAGGGCGAATCCGATGATCAAGGACGTAGGCTAGAGTTTCGAAAACGATTAGAGACCGGAGTAGTTCTAGCAGTAAACTATGCCGACGCCGTGGTAT-CT--AATTTATCT-------GGTATTGCGGAAGAGAAATCAAGT-AAGGCTTTGGGGAGAGTACGCGCGCAAGCGATAAATTTAAAGGAAATTGACGGAGGAACACCACAAGGAGTGGAGTGTGCGGCT--------------------------------------------------------------------------------------------------------------------------------------------------------------------------------------------------------------------------------------------------------------------------------------------------------------------------------------------------------------------------------------------------------------------------------------------------------------------------------------------------------------------------------

>>N1_PLZWC9_PL_Chae_i_D.sp._N1_KAR-18_1752pb_MG773222*

-------------------------------------------CGTTTAACGTGGAGCGGTGAAAGGCTCAGTAACGGGCGATTTATTTGATCTCCTGGGACGGACAACATCGGGAAACTGATGGGAAAACGTCTAAGTTGCAG------T----TGTATGATTGTGACGTAAA-CCT---TTGGTGCAGGAGAGTAAGATGCCATCCTATCAGTTAGTAAGTAGGGTAAGGGCCTACTTAGACGAAGACGGGTACGGGGAATGAGGGTTTGATTCCGGAGAGGGAGCCTGAGAGACGGCTACCAGGACCAAGGTCAGCAGCAGGCGCGAAAATTATCGAAGCCCGCC-GAGGGGCGATAGTGAGGAGACGTGTATAACGAAGTACGTGTAAAGAACGTGCTAACAACTGGA-GGTCAAGTCTGGTGCCAGCATCCGCGGTAATTCCAGCTCCAGGGGTGTCTATGATGATTGCTGCGATTAAAAAGTCCGTAGTCAATCTGTCTG-ACTTGCCTGCAATG-TGATTGATTAAAGAACGAGCAGGGCTAGGAAAGCAGAGAATTAGGAGCGACCGAGGGCTAGAGTATTGAATGGCGAGAGGTGAAATTTGATGACCCATTCAGGAGTGACAAAGGCGAAGGCACTAGTCAAGGGCGAATCCGATGATCAAGGACGTAGGCTAGAGTTTCGAAAACGATTAGAGACCGGAGTAGTTCTAGCAGTAAACTATGCCGACGCCGTGGTAT-GT--ATTT----A-------TGTATTGCGGAAGAGAAATCAAGT-AAGGCTTTGGGGAGAGTACGCGCGCAAGCGATAAATTTAAAGGAAATTGACGGAGGAACACCACAAGGAGTGGAGTGTGCGGCTTAATTTGACTCAACGCGGGACAGCTTACCAGGCCCGATAATCGAGCGAGCGTAGTACGCGATAGGTTAAAGAGTGGTGCATGGCTGCTATCGACAGTTGGGGTGACC-TTAGGGTTAATTCCGGCAAGTAGTGAGACCCCTGCAGATAG--TGGACAGGTATTTTA-AAAATACAGGAAGGAAGGGACAAGAGCAGGTCAGTGATGCCCTTAGATGGCCTGGGCTGCACGCGCACTACAGTGGTCATTATAAGACGAAAGTAGA-TATAAAGATGATCGAGAGGGACTGAGCTTTGTAAGAGGCTCACGAACGAGGAATTGCTAGTAATCGTAGGCTCATTAAGATACGATGAATATGTCCCTGTACCTTGTACACACCGCCCGTCGTTATCGAAGATGGAATTGTGTGCGAACGAGCAATAAGCGAGTGAGCGCATAGTTCTAGATGTGATAAAAGTCGTAACAAGGCAACTGTAGGAGAACCTGTAGTTGGATCATACAGATATT-GTCA

>>MF399467_SE_Glac_Dsp_IRO-17_1194pb_MF399467

------------------------------------------------------------------------------------TATTTGTTCTCCTGTGACGGACAACATCGGGAAATTGATGGGAAAACGTCTAAGTTGCAG------T----TAATTGATTGTGACGTAAA-CGA--TTTTGTGCAGGAGAGTAAGATGCCATCCTATCAGTTAGTAAGTAGGGTAAGGGCCTACTTAGACGAAGACGGGTACGGGGAATGAGGGTTTGATTCCGGAGAGGGAGCCTGAGAGATGGCTACCAGGACCAAGGTCAGCAGCAGGCGCGAAAATTATCGAAGCCCGCC-TAGGGGCGATAGTGAGGAGACGTGTATAACGAAGTACGTGTAAAGAACGTGCTAATAACTGGA-GGTCAAGTCTGGTGCCAGCATCCGCGGTAATTCCAGCTCCAGGGGTGTCTATGATGATTGCTGCGATTAAAAAGTCCGTAGTCAATCTGACTG-ACTGACCTGCAATG-TGATTGATTAAAGAACGAGCAGGGTTAGGAAAGCAGAGAATTAGGAGCGACCGAGGGCTAGAGTATTGAATGGCGAGAGGTGAAATTTGATGACCCATTCAGGAGTGACAAAGGCGAAGGCACTAGTCAAGGGCGAATCCGATGATCAAGGACGTAGGCTAGAGTTTCGAAAACGATTAAAAGCCGGAGGAATTTTAACCATTAAATAATCCGGCCCCGGGAAAA-AG---TTT----TTTGTGGTTGTATTGCGGAAGAGAAATCAAGT-AAGGCTTTGGGGAGAGTACGCGCGCAAGCGATAAATTTAAAGGAAATTGACGGAGGAACACCACAAGGAGTGGAGTGTGCGGCTTAATTTGACTCAACGCGGGACAGCTTACCAGGCCCGATAATCGAGCGAGCGTAGTACGCGATAGATTAAAGAGTGGTGCATGGCTGCTATCGACAGTTGGGGTGACC-TTAGGGTTAATTCCGGCAAGTAGTGAGACCCCTGCAGATAG--TGGACAGGTATTTTT-AAAATACAGGAAGGAAGGGACAAGAGCAGGTCAGTGATGCCCTTAGATGGCCTGGGCTGCACGCGCACTACAGTGGTCATTATAAGTAGAAGTTAGA-AGTAAAGATGATCGAGAGGGACTGAGCTTTGTAAGAGGCTCACGAACGAGGAATTGCTAGTAATCGTAGGCTCATTAAGATACGATGAATATGTCCCTGTACCTTGTACACACCGCCCGTCGTTATCGAAGATGGAATTGTGTGCGAACGAGCAACAAGCGAG-GAGC-------------------------------------------------------------------------------

>>MG029372_GB_Gpul_Dsp_IRO-17_583pb_MG029372

-----------------------------------------------------------------------------------------------------------------------------------------------------------------------------------------------------------------------------------------------------------------------------------------------------------------------------------------------------------------------------------------------------------------------------------------------------------------------------------------------------------------------------------------------------------------------------------------------------------------------------------------------------------------------------------------------------------------------------------GGAGTAGTTCTAGCAGTAAAATATGCCGACGCCGCGATAT-AG---TTT----TTTGTGGCTGTGTTGCGGAAGAGAAATCAAGT-AAGGCTTTGGGGAGAGTACGCGCGCAAGCGATAAATTTAAAGGAAATTGACGGAGGAACACCACAAGGAGTGGAGTGTGCGGCTTAATTTGACTCAACGCGGGACAGCTTACCAGGCCCGATAATCGAGCGAGCGTTGTACGCGATAGATTAAAGAGTGGTGCATGGCTGCTATCGACAGTTGGGGTGACC-TTAGGGTTAATTCCGGCAAGTAGTGAGACCCCTGCAGATAG--TGGACAGGTATTTTT-AAAATACAGGAAGGAAGGGACAAGAGCAGGTCAGTGATGCCCTTAGATGGCCTGGGCTGCACGCGCACTACAGTGGTCATTATAAGTAGAATTTAGA-AGTAAAGATGATCGAGAGGGACTGAGCTTTGTAAGAGGCTCACGAACGAGGAATTGCTAGTAATCGTAGGCTCATTAAGATACGATGAATATGTCCCTGTACCTTGTACACACCGCCCGTCGTTATCGAAGATGGAATTGTGTGCGAACGAGCAACAAGCGA-------------------------------------------------------------------------------------

>>HQ683742_PL_Dhae_Dsp-33_WIL-11_545pb_HQ683742

--------------------------------------------------------------------------------------------------------------------------------------------GC--------T-------------------------------------------------------------------------------------------------------------------------------------------------------CCAGGACCAAGGTCAGCACCAGGCGCGAAAATTATCGAAGCCCGCC-TAGGGGCGATAGTGAGGAGACGTGTATAACGAAGTACGTGTAAAG-ACGTCCTAATACCTGGA-GGTCAAGTCTGGTGCCAGCATCCGCGGTAATTCCAGCTCCAGGGGTGTCTATGATGATTGCTGCGATTAAAAAGTCCGTAGTCAAACTGACTG-ACTGACCTGCAATG-TGATTGATTAAAGAACGAGCAGGGTTAGGAAAGCAGAGAATTAGGAGCGACCGAGGGCTAGAGTATTGAATGGCGAGAGGTGAAATTTGATGACCCATTCAGGAGTGACAAAGGCGAAGGCACTAGTCAAGGGCGAATCCGATGATCAAGGACGTAGGCTAGAGTTTCGAAAACGATTAGAGACCGGAGTAGTTCTAGCAGTAAACTATGCCGACGCCGTGATAT-TG---TTT----TTTGTGGCAGTATTGCGGAAGAGAAATCAAGT-AAGGCTTTGGGGAGAGTACGCGCGCAAGCGATAAATTTAAAGGAAA-TGACGGAGGAACACCACAA--------------------------------------------------------------------------------------------------------------------------------------------------------------------------------------------------------------------------------------------------------------------------------------------------------------------------------------------------------------------------------------------------------------------------------------------------------------------------------------------------------------------------------------------------

>>PL1-08_PL_Groe_Dber1_ADR-18_249pb_PL1-08

------------------------------CATGTGTAAGCGAAGCGTAACGTGGAGCGGTGAAAGGCTCAGTAACGGGCGAGTTATTTGTTCTCCTGGGACGGACAACACCGGGAAACTGGTGGGAAAACGTCTAAGTTGCGG------T----TTTTTAATCGTGGCGTAAA-CCA--TGTGGTGCAGGAGAGTAAGCTGCCATCCTATCAGTTAGTAAGTAGGGTAAGGGCCTACTTAGACGAAGACGGGTACGGGGAATGAGGGTTTGATTCCGGAGAGGGAGCCTGA-----------------------------------------------------------------------------------------------------------------------------------------------------------------------------------------------------------------------------------------------------------------------------------------------------------------------------------------------------------------------------------------------------------------------------------------------------------------------------------------------------------------------------------------------------------------------------------------------------------------------------------------------------------------------------------------------------------------------------------------------------------------------------------------------------------------------------------------------------------------------------------------------------------------------------------------------------------------------------------------------------------------------------------------------------------------------------------------------------------------------------------------

>>B2_D08_Ct-17_NL_Chae_t_Dber_KAR-18_1755pb_MG773246

-------------------------------------------AGCGTGGAGCGGAGCAGTGAAAGGCTCAGTAACGGGCGAGTTATTTGTTCTCCTGGGACGGACAACACCGGGAAACTGGTGGGAAAACGTCTAAGTTGCGG------T----TTTTTAATCGTGGCGTAAA-CCA--TTTGGTGCAGGAGAGTAAGCTGCCATCCTATCAGTTAGTAAGTAGGGTAAGGGCCTACTTAGACGAAGACGGGTACGGGGAATGAGGGTTTGATTCCGGAGAGGGAGCCTGAGAGACGGCTACCAGGACCAAGGTCAGCAGCAGGCGCGAAAATTATCGAAGCCCGCG-TAGGGGCGATAGTGAGGAGACGTGTATTACGAAGTGTGTGTAAAGAACGCACTAATAACTGGA-GGTCAAGTCTGGTGCCAGCATCCGCGGTAATTCCAGCTCCAGGGGTGTCTATGATGATTGCTGCGATTAAAAAGTCCGTAGTCAAGCTGACTG-ACTTGCCTGCAATG-TGACTGATTAAGAGACGAGCAGGGCTAGGAAAGCAGAGAATTAGGAGCGACCGAGGGCTAGAGTATTGAATGGCGAGAGGTGAAATTTGATGACCCATTCAGGAGTGACAAAGGCGAAGGCACTAGTCAAGGGCGAATCCGATGATCAAGGACGTAGGCTAGAGTTTCGAAAACGATTAGAGACCGGAGTAGTTCTAGCAGTAAACTATGCCGACGCCGTGGTAT-GG--TATT----C-------TGTATTGCGGAAGAGAAATCAAGT-AAGGCTTTGGGGAGAGTACGCGCGCAAGCGATAAATTTAAAGGAAATTGACGGAGGAACACCACAAGGAGTGGAGTGTGCGGCTTAATTTGACTCAACGCGGGACAGCTTACCAGGCCCGATAATCATACGAGCGTAGTACGCGATAGGTTAGAGAGTGGTGCATGGCTGCTATCGACAGTTGGGGTGACC-TTAGGGTTAATTCCGGCAAGTAGTGAGACCTCTGCAGTTA---TGGACAGGTATTTTT-AAGATACAGGAAGGAAGAGACAAGAGCAGGTCAGTGATGCCCTTAGATGGCCTGGGCTGCACGCGCACTACAGTGGTCATTATAAGTAGAAGTTAGATTTTAAAGATGATCGAGAGGGACTGGGCTTTGTAAGAGGCCCAAGAACGAGGAATTGCTAGTAATCGTAGGCTCATTAAGATACGATGAATATGTCCCTGTACCTTGTACACACCGCCCGTCGTTATCGAAGATGGAATTGTGTGCGAACGAGCAACAAGCGAGTGAGCGCATAGTTCTAGATGTGATAAAAGTCGTAACAAGGCAACTGTAGGAGAACCTGTAGTTGGATCATACAGATATA-ATAA

>>HQ681058_LV_Pontogammarus_robustoides_Dsp-41-JEI-2011_WIL-11_509pb_HQ681058

--------------------------------------------------------------------------------------------------------------------------------------------------------------------------------------------------------------------------------------------------------------------------------------------------------------CCAGGRCCAAGGTCAGCAGCAGGCGCGAAAATTATCGAAGCCCGCG-TAGGGGCGATAGTGAGGAGACGTGTATTACGAAGTGTGTGTAAAGAACGCACTAATAACTGGA-GGTCAAGTCTGGTGCCAGCATCCGCGGTAATTCCAGCTCCAGGGGTGTCTATGATGATTGCTGCGATTAAAAAGTCCGTAGTCAAGCTGACTG-ACTTGCCTGCAATG-TGACTGATTAAGAGACGAGCAGGGCTAGGAAAGCAGAGAATTAGGAGCGACCGAGGGCTAGAGTATTGAATGGCGAGAGGTGAAATTTGATGACCCATTCAGGAGTGACAAAGGCGAAGGCACTAGTCAAGGGCGAATCCGATGATCAAGGACGTAGGCTAGAGTTTCGAAAACGATTAGAGACCGGAGTAGTTCTAGCAGTAAACTATGCCGACGCCGTGGTAT-GG--TATT----T-------TGTATTGCGGAAGAGAAATCAAGT-AAGGCTTTGGGGAGAGTACGCGCGCAAGCGATAAATT-------------------------------------------------------------------------------------------------------------------------------------------------------------------------------------------------------------------------------------------------------------------------------------------------------------------------------------------------------------------------------------------------------------------------------------------------------------------------------------------------------------------------------------------------------------------------------

>>B6_ZS59_PL_Dvil_Dber_KAR-18_1756pb_MG773250*

-------------------------------------------AGCGTAACGTGGAGCGGTGAAAGGCTCAGTAACGGGCGAGTTATTTGTTCTCCTGGGACGGACAACACCGGGAAACTGGTGGGAAAACGTCTAAGTTGCGG------T----TTTTTAATCGTGGCGTAAA-CCA--TTTGGTGCAGGAGAGTAAGCTGCCATCCTATCAGTTAGTAAGTAGGGTAAGGGCCTACTTAGACGAAGACGGGTACGGGGAATGAGGGTTTGATTCCGGAGAGGGAGCCTGAGAGACGGCTACCAGGACCAAGGTCAGCAGCAGGCGCGAAAATTATCGAAGCCCGCG-TAGGGGCGATAGTGAGGAGACGTGTATTACGAAGTGTGTGTAAAGAACGCACTAATAACTGGA-GGTCAAGTCTGGTGCCAGCATCCGCGGTAATTCCAGCTCCAGGGGTGTCTATGATGATTGCTGCGATTAAAAAGTCCGTAGTCAAGCTGACTG-ACTTGCCTGCAATG-TGACTGATTAAGAGACGAGCAGGGCTAGGAAAGCAGAGAATTAGGAGCGACCGAGGGCTAGAGTATTGAATGGCGAGAGGTGAAATTTGATGACCCATTCAGGAGTGACAAAGGCGAAGGCACTAGTCAAGGGCGAATCCGATGATCAAGGACGTAGGCTAGAGTTTCGAAAACGATTAGAGACCGGAGTAGTTCTAGCAGTAAACTATGCCGACGCCGTGGTAT-GG--TATT----C-------TGTATTGCGGAAGAGAAATCAAGT-AAGGCTTTGGGGAGAGTACGCGCGCAAGCGATAAATTTAAAGGAAATTGACGGAGGAACACCACAAGGAGTGGAGTGTGCGGCTTAATTTGACTCAACGCGGGACAGCTTACCAGGCCCGATAATCATACGAGCGTAGTACGCGATAGGTTAGAGAGTGGTGCATGGCTGCTATCGACAGTTGGGGTGACC-TTAGGGTTAATTCCGGCAAGTAGTGAGACCTCTGCAGTTA---TGGACAGGTATTTTT-AAGATACAGGAAGGAAGAGACAAGAGCAGGTCAGTGATGCCCTTAGATGGCCTGGGCTGCACGCGCACTACAGTGGTCATTATAAGTAGAAGTTAGATTTTAAAAATGATCGAGAGGGACTGGGCTTTGTAAGAGGCCCAAGAACGAGGAATTGCTAGTAATCGTAGGCTCATTAAGATACGATGAATATGTCCCTGTACCTTGTACACACCGCCCGTCGTTATCGAAGATGGAATTGTGTGCGAACGAGCAACAAGCGAGTGAGCGCATAGTTCTAGATGTGATAAAAGTCGTAACAAGGCAACTGTAGGAGAACCTGTAGTTGGATCATACAGATATA-ATAA

>>B12_DOP6_PL_Dhae_Dber_KAR-18_1756pb_MG773256*

-------------------------------------------AGCGTAACGTGGAGCGGTGAAAGGCTCAGTAACGGGCGAGTTATTTGTTCTCCTGGGACGGACAACACCGGGAAACTGGTGGGAAAACGTCTAAGTTGCGG------T----TTTTTAATCGTGGCGTAAA-CCA--TGTGGTGCAGGAGAGTAAGCTGCCATCCTATCAGTTAGTAAGTAGGGTAAGGGCCTACTTAGACGAAGACGGGTACGGGGAATGAGGGTTTGATTCCGGAGAGGGAGCCTGAGAGACGGCTACCAGGACCAAGGTCAGCAGCAGGCGCGAAAATTATCGAAGCCCGCG-TAGGGGCGATAGTGAGGAGACGTGTATTACGAAGTGTGTGTAAAGAACGCACTAATAACTGGA-GGTCAAGTCTGGTGCCAGCATCCGCGGTAATTCCAGCTCCAGGGGTGTCTATGATGATTGCTGCGATTAAAAAGTCCGTAGTCAAGCTGACTG-ACTTGCCTGCAATG-TGACTGATTAAGAGACGAGCAGGGCTAGGAAAGCAGAGAATTAGGAGCGACCGAGGGCTAGAGTATTGAATGGCGAGAGGTGAAATTTGATGACCCATTCAGGAGTGACAAAGGCGAAGGCACTAGTCAAGGGCGAATCCGATGATCAAGGACGTAGGCTAGAGTTTCGAAAACGATTAGAGACCGGAGTAGTTCTAGCAGTAAACTATGCCGACGCCGTGGTAT-GG--TATT----C-------TGTATTGCGGAAGAGAAATCAAGT-AAGGCTTTGGGGAGAGTACGCGCGCAAGCGATAAATTTAAAGGAAATTGACGGAGGAACACCACAAGGAGTGGAGTGTGCGGCTTAATTTGACTCAACGCGGGACAGCTTACCAGGCCCGATAATCATACGAGCGTAGTACGCGATAGGTTAGAGAGTGGTGCATGGCTGCTATCGACAGTTGGGGTGACC-TTAGGGTTAATTCCGGCAAGTAGTGAGACCTCTGCAGTTA---TGGACAGGTATTTTT-AAGATACAGGAAGGAAGAGACAAGAGCAGGTCAGTGATGCCCTTAGATGGCCTGGGCTGCACGCGCACTACAGTGGTCATTATAAGTAGAAGTTAGATTTTAAAAATGATCGAGAGGGACTGGGCTTTGTAAGAGGCCCAAGAACGAGGAATTGCTAGTAATCGTAGGCTCATTAAGATACGATGAATATGTCCCTGTACCTTGTACACACCGCCCGTCGTTATCGAAGATGGAATTGTGTGCGAACGAGCAACAAGCGAGTGAGCGCATAGTTCTAGATGTGATAAAAGTCGTAACAAGGCAACTGTAGGAGAACCTGTAGTTGGATCATACAGATATA-ATAA

>>B7_ProbM32_HU_Prob_Dber_KAR-18_1756pb_MG773251*

-------------------------------------------AGCGTAACGTGGAGCGGTGAAAGGCTCAGTAACGGGCGAGTTATTTGTTCTCCTGGGACGGACAACACCGGGAAACTGGTGGGAAAACGTCTAAGTTGCGG------T----TTTTTAATCGTGGCGTAAA-CCA--TTTGGTGCAGGAGAGTAAGCTGCCATCCTATCAGTTAGTAAGTAGGGTAAGGGCCTACTTAGACGAAGACGGGTACGGGGAATGAGGGTTTGATTCCGGAGAGGGAGCCTGAGAGACGGCTACCAGGACCAAGGTCAGCAGCAGGCGCGAAAATTATCGAAGCCCGCG-TAGGGGCGATAGTGAGGAGACGTGTATTACGAAGTGTGTGTAAAGAACGCACTAATAACTGGA-GGTCAAGTCTGGTGCCAGCATCCGCGGTAATTCCAGCTCCAGGGGTGTCTATGATGATTGCTGCGATTAAAAAGTCCGTAGTCAAGCTGACTG-ACTTGCCTGCAATG-TGACTGATTAAGAGACGAGCAGGGCTAGGAAAGCAGAGAATTAGGAGCGACCGAGGGCTAGAGTATTGAATGGCGAGAGGTGAAATTTGATGACCCATTCAGGAGTGACAAAGGCGAAGGCACTAGTCAAGGGCGAATCCGATGATCAAGGACGTAGGCTAGAGTTTCGAAAACGATTAGAGACCGGAGTAGTTCTAGCAGTAAACTATGCCGACGCCGTGGTAT-GG--TATT----C-------TGTATTGCGGAAGAGAAATCAAGT-AAGGCTTTGGGGAGAGTACGCGCGCAAGCGATAAATTTAAAGGAAATTGACGGAGGAACACCACAAGGAGTGGAGTGTGCGGCTTAATTTGACTCAACGCGGGACAGCTTACCAGGCCCGATAATCATACGAGCGTAGTACGCGATAGGTTAGAGAGTGGTGCATGGCTGCTATCGACAGTTGGGGTGACC-TTAGGGTTAATTCCGGCAAGTAGTGAGACCTCTGCAGTTA---TGGACAGGTATTTTT-AAGATACAGGAAGGAAGAGACAAGAGCAGGTCAGTGATGCCCTTAGATGGCCTGGGCTGCACGCGCACTACAGTGGTCATTATAAGTAGAAGTTAGATTTTAAAGATGATCGAGAGGGACTGGGCTTTGTAAGAGGCCCAAGAACGAGGAATTGCTAGTAATCGTAGGCTCATTAAGATACGATGAATATGTCCCTGTACCTTGTACACACCGCCCGTCGTTATCGAAGATGGAATTGTGTGCGAACGAGCAACAAGCGAGTGAGCGCATAGTTCTAGATGTGATAAAAGTCGTAACAAGGCAACTGTAGGAGAACCTGTAGTTGGATCATACAGATATA-ATAA

>>B10_DZT3_PL_Dhae_Dber_KAR-18_1756pb_MG773254*

-------------------------------------------AGCGTAACGTGGAGCGGTGAAAGGCTCAGTAACGGGCGAGTTATTTGTTCTCCTGGGACGGACAACACCGGGAAACTGGTGGGAAAACGTCTAAGTTGCGG------T----TTTTTAATCGTGGCGTAAA-CCA--TGTGGTGCAGGAGAGTAAGCTGCCATCCTATCAGTTAGTAAGTAGGGTAAGGGCCTACTTAGACGAAGACGGGTACGGGGAATGAGGGTTTGATTCCGGAGAGGGAGCCTGAGAGACGGCTACCAGGACCAAGGTCAGCAGCAGGCGCGAAAATTATCGAAGCCCGCG-TAGGGGCGATAGTGAGGAGACGTGTATTACGAAGTGTGTGTAAAGAACGCACTAATAACTGGA-GGTCAAGTCTGGTGCCAGCATCCGCGGTAATTCCAGCTCCAGGGGTGTCTATGATGATTGCTGCGATTAAAAAGTCCGTAGTCAAGCTGACTG-ACTTGCCTGCAATG-TGACTGATTAAGAGACGAGCAGGGCTAGGAAAGCAGAGAATTAGGAGCGACCGAGGGCTAGAGTATTGAATGGCGAGAGGTGAAATTTGATGACCCATTCAGGAGTGACAAAGGCGAAGGCACTAGTCAAGGGCGAATCCGATGATCAAGGACGTAGGCTAGAGTTTCGAAAACGATTAGAGACCGGAGTAGTTCTAGCAGTAAACTATGCCGACGCCGTGGTAT-GG--TATT----C-------TGTATTGCGGAAGAGAAATCAAGT-AAGGCTTTGGGGAGAGTACGCGCGCAAGCGATAAATTTAAAGGAAATTGACGGAGGAACACCACAAGGAGTGGAGTGTGCGGCTTAATTTGACTCAACGCGGGACAGCTTACCAGGCCCGATAATCATACGAGCGTAGTACGCGATAGGTTAGAGAGTGGTGCATGGCTGCTATCGACAGTTGGGGTGACC-TTAGGGTTAATTCCGGCAAGTAGTGAGACCTCTGCAGTTA---TGGACAGGTATTTTT-AAGATACAGGAAGGAAGAGACAAGAGCAGGTCAGTGATGCCCTTAGATGGCCTGGGCTGCACGCGCACTACAGTGGTCATTATAAGTAGAAGTTAGATTTTAAAGATGATCGAGAGGGACTGGGCTTTGTAAGAGGCCCAAGAACGAGGAATTGCTAGTAATCGTAGGCTCATTAAGATACGATGAATATGTCCCTGTACCTTGTACACACCGCCCGTCGTTATCGAAGATGGAATTGTGTGCGAACGAGCAACAAGCGAGTGAGCGCATAGTTCTAGATGTGATAAAAGTCGTAACAAGGCAACTGTAGGAGAACCTGTAGTTGGATCATACAGATATA-ATAA

>>B15_DOP13_PL_Dhae_Dber_KAR-18_1756pb_MG773259

-------------------------------------------AGCGTAACGTGGAGCGGTGAAAGGCTCAGTAACGGGCGAGTTATTTGTTCTCCTGGGACGGACAACACCGGGAAACTGGTGGGAAAACGTCTAAGTTGCGG------T----TTTTTAATCGTGGCGTAAA-CCA--TGTGGTGCAGGAGAGTAAGCTGCCATCCTATCAGTTAGTAAGTAGGGTAAGGGCCTACTTAGACGAAGACGGGTACGGGGAATGAGGGTTTGATTCCGGAGAGGGAGCCTGAGAGACGGCTACCAGGACCAAGGTCAGCAGCAGGCGCGAAAATTATCGAAGCCCGCG-TAGGGGCGATAGTGAGGAGACGTGTATTACGAAGTGTGTGTAAAGAACGCACTAATAACTGGA-GGTCAAGTCTGGTGCCAGCATCCGCGGTAATTCCAGCTCCAGGGGTGTCTATGATGATTGCTGCGATTAAAAAGTCCGTAGTCAAGCTGACTG-ACTTGCCTGCAATG-TGACTGATTAAGAGACGAGCAGGGCTAGGAAAGCAGAGAATTAGGAGCGACCGAGGGCTAGAGTATTGAATGGCGAGAGGTGAAATTTGATGACCCATTCAGGAGTGACAAAGGCGAAGGCACTAGTCAAGGGCGAATCCGATGATCAAGGACGTAGGCTAGAGTTTCGAAAACGATTAGAGACCGGAGTAGTTCTAGCAGTAAACTATGCCGACGCCGTGATAT-GG--TATT----C-------TGTATTGCGGAAGAGAAATCAAGT-AAGGCTTTGGGGAGAGTACGCGCGCAAGCGATAAATTTAAAGGAAATTGACGGAGGAACACCACAAGGAGTGGAGTGTGCGGCTTAATTTGACTCAACGCGGGACAGCTTACCATGCCCGATAATCATACGAGCGTAGTACGCGATAGGTTAGAGAGTGGTGCATGGCTGCTATCGACAGTTGGGGTGACC-TTAGGGTTAATTCCGGCAAGTAGTGAGACCTCTGCAGTTA---TGGACAGGTATTTTT-AAGATACAGGAAGGAAGAGACAAGAGCAGGTCAGTGATGCCCTTAGATGGCCTGGGCTGCACGCGCACTACAGTGGTCATTATAAGTAGAAGTTAGATTTTAAAGATGATCGAGAGGGACTGGGCTTTGTAAGAGGCCCAAGAACGAGGAATTGCTAGTAATCGTAGGCTCATTAAGATACGATGAATATGTCCCTGTACCTTGTACACACCGCCCGTCGTTATCGAAGATGGAATTGTGTGCGAACGAGCAACAAGCGAGTGAGCGCATAGTTCTAGATGTGATAAAAGTCGTAACAAGGCAACTGTAGGAGAACCTGTAGTTGGATCATACAGATATA-ATAA

>>B16_DOR9_PL_Dhae_Dber_KAR-18_1756pb_MG773260

-------------------------------------------AGCGTAACGTGGAGCGGTGAAAGGCTCAGTAACGGGCGAGTTATTTGTTCTCCTGGGACGGACAACACCGGGAAACTGGTGGGAAAACGTCTAAGTTGCGG------T----TTTTTAATCGTGGCGTAAA-CCA--TGTGGTGCAGGAGAGTAAGCTGCCATCCTATCAGTTAGTAAGTAGGGTAAGGGCCTACTTAGACGAAGACGGGTACGGGGAATGAGGGTTTGATTCCGGAGAGGGAGCCTGAGAGACGGCTACCAGGACCAAGGTCAGCAGCAGGCGCGAAAATTATCGAAGCCCGCG-TAGGGGCGATAGTGAGGAGACGTGTATTACGAAGTGTGTGTAAAGAACGCACTAATAACTGGA-GGTCAAGTCTGGTGCCAGCATCCGCGGTAATTCCAGCTCCAGGGGTGTCTATGATGATTGCTGCGATTAAAAAGTCCGTAGTCAAGCTGACTG-ACTTGCCTGCAATG-TGACTGATTAAGAGACGAGCAGGGCTAGGAAAGCAGAGAATTAGGAGCGACCGAGGGCTAGAGTATTGAATGGCGAGAGGTGAAATTTGATGACCCATTCAGGAGTGACAAAGGCGAAGGCACTAGTCAAGGGCGAATCCGATGATCAAGGACGTAGGCTAGAGTTTCGAAAACGATTAGAGACCGGAGTAGTTCTAGCAGTAAACTATGCCGACGCCGTGGTAT-GG--TATT----C-------TGTATTGCGGAAGAGAAATCAAGT-AAGGCTTTGGGGAGAGTACGCGCGCAAGCGATAAATTTAAAGGAAATTGACGGAGGAACACCACAAGGAGTGGAGTGTGCGGCTTAATTTGACTCAACGCGGGACAGCTTACCATGCCCGATAATCATACGAGCGTAGTACGCGATAGGTTAGAGAGTGGTGCATGGCTGCTATCGACAGTTGGGGTGACC-TTAGGGTTAATTCCGGCAAGTAGTGAGACCTCTGCAGTTA---TGGACAGGTATTTTT-AAGATACAGGAAGGAAGAGACAAGAGCAGGTCAGTGATGCCCTTAGATGGCCTGGGCTGCACGCGCACTACAGTGGTCATTATAAGTAGAAGTTAGATTTTAAAAATGATCGAGAGGGACTGGGCTTTGTAAGAGGCCCAAGAACGAGGAATTGCTAGTAATCGTAGGCTCATTAAGATACGATGAATATGTCCCTGTACCTTGTACACACCGCCCGTCGTTATTGAAGATGGAATTGTGTGCGAACGAGCAACAAGCGAGTGAGCGCATAGTTCTAGATGTGATAAAAGTCGTAACAAGGCAACTGTAGGAGAACCTGTAGTTGGATCATACAGATATA-ATAA

>>AJ438957_GB_Echinogammarus_berilloni_Dber_TER-04_912pb_AJ438957

------------------------------CATGTGTAAGCGAAGCGTAACGTGGAGCGGTGAAAGGCTCAGTAACGGGCGAGTTATTTGTTCTCCTGGGACGGACAACACCGGGAAACTGGTGGGAAAACGTCTAAGTTGCGG------T----TTTTTAATCGTGGCGTAAA-CCA--TTTGGTGCAGGAGAGTAAGCTGCCATCCTATCAGTTAGTAAGTAGGGTAAGGGCCTACTTAGACGAAGACGGGTACGGGGAATGAGGGTTTGATTCCGGAGAGGGAGCCTGAGAGACGGCTACCAGGACCAAGGTCAGCAGCAGGCGCGAAAATTATCGAAGCCCGCA-TAGGGGCGATAGTGAGGAGACGTGTATTACGAAGTGTGTGTAAAGAACGCACTAATAACTGGA-GGTCAAGTCTGGTGCCAGCATCCGCGGTAATTCCAGCTCCAGGGGTGTCTATGATGATTGCTGCGATTAAAAAGTCCGTAGTCAAGCTGACTG-ACTTGCCTGCAATG-TGACTGATTAAGAGACGAGCAGGGCTAGGAAAGCAGAGAATTAGGAGCGACCGAGGGCTAGAGTATTGAATGGCGAGAGGTGAAATTTGATGACCCATTCAGGAGTGACAAAGGCGAAGGCACTAGTCAAGGGCGAATCCGATGATCAAGGACGTAGGCTAGAGTTTCGAAAACGATTAGAGACCGGAGTAGTTCTAGCAGTAAACTATGCCGACGCCGTGGTAT-GG--TATT----C-------TGTATTGCGGAAGAGAAATCAAGT-AAGGCTTTGGGGAGAGTACGCGCGCAAGCGATAAATTTAAAGGAAATTGACGGAGGAACACCACAAGGAGTGGAGTGTGCGGCTTAATTTGACTCAACGCGGGACAGCTTACCAGGCCCGATAATCATACGAGCGTAGTACGCGATAGGTTAAAGAGTGGTGCATGGCTGCTATCGACAGT-------------------------------------------------------------------------------------------------------------------------------------------------------------------------------------------------------------------------------------------------------------------------------------------------------------------------------------------------------------------------------------------------------------------------------

>>B9_DML3_PL_Dhae_Dber_KAR-18_1755pb_MG773253

-------------------------------------------AGCGTAACGTGGAGCGGTGAAAGGCTCAGTAACGGGCGAGTTATTTGTTCTCCTGGGACGGACAACACCGGGAAACTGGTGGGAAAACGTCTAAGTTGCGG------T----TTTTTAATCGTGGCGTAAA-CCA--TGTGGTGCAGGAGAGTAAGCTGCCATCCTATCAGTTAGTAAGTAGGGTAAGGGCCTACTTAGACGAAGACGGGTACGGGGAATGAGGGTTTGATTCCGGAGAGGGAGCCTGAGAGACGGCTACCAGGACCAAGGTCAGCAGCAGGCGCGAAAATTATCGAAGCCCGCG-TAGGGGCGATAGTGAGGAGACGTGTATTACGAAGTGTGTGTAAAGAACGCACTAATAACTGGA-GGTCAAGTCTGGTGCCAGCATCCGCGGTAATTCCAGCTCCAGGGGTGTCTATGATGATTGCTGCGATTAAAAAGTCCGTA-TCAAGCTGACTG-ACTTGCCTGCAATG-TGACTGATTAAGAGACGAGCAGGGCTAGGAAAGCAGAGAATTAGGAGCGACCGAGGGCTAGAGTATTGAATGGCGAGAGGTGAAATTTGATGACCCATTCAGGAGTGACAAAGGCGAAGGCACTAGTCAAGGGCGAATCCGATGATCAAGGACGTAGGCTAGAGTTTCGAAAACGATTAGAGACCGGAGTAGTTCTAGCAGTAAACTATGCCGACGCCGTGGTAT-GG--TATT----C-------TGTATTGCGGAAGAGAAATCAAGT-AAGGCTTTGGGGAGAGTACGCGCGCAAGCGATAAATTTAAAGGAAATTGACGGAGGAACACCACAAGGAGTGGAGTGTGCGGCTTAATTTGACTCAACGCGGGACAGCTTACCAGGCCCGATAATCATACGAGCGTAGTACGCGATAGGTTAGAGAGTGGTGCATGGCTGCTATCGACAGTTGGGGTGACC-TTAGGGTTAATTCCGGCAAGTACTGAGACCTCTGCAGTTA---TGGACAGGTATTTTT-AAGATACAGGAAGGAAGAGACAAGAGCAGGTCAGTGATGCCCTTAGATGGCCTGGGCTGCACGCGCACTACAGTGGTCATTATAAGTAGAAGTTAGATTTTAAAGATGATCGAGAGGGACTGGGCTTTGTAAGAGGCCCAAGAACGAGGAATTGCTAGTAATCGTAGGCTCATTAAGATACGATGAATATGTCCCTGTACCTTGTACACACCGCCCGTCGTTATCGAAGATGGAATTGTGTGCGAACGAGCAACAAGCGAGTGAGCGCATAGTTCTAGATGTGATAAAAGTCGTAACAAGGCAACTGTAGGAGAACCTGTAGTTGGATCATACAGATATA-ATAA

>>HQ681059_FR_Echinogammarus_berilloni_Dsp-42-JEI-2011_WIL-11_509pb_HQ681059

--------------------------------------------------------------------------------------------------------------------------------------------------------------------------------------------------------------------------------------------------------------------------------------------------------------CCAGGACCAAGGTCAGCAGCAGGCGCGAAAATTATCGAAGCCCGCG-TAGGGGCGATAGTGAGGAGACGTGTATTACGAAGTGTGTGTAAAGAACGCACTAATAACTGGA-GGTCAAGTCTGGTGCCAGCATCCGCGGTAATTCCAGCTCCAGGGGTGTCTATGATGATTGCTGCGATTAAAAAGTCCGTAGTCAAGCTGACTG-ACTTGCCTGCAATG-TGAGTGATTAAGAGACGAGCAGGGGTAGGAAAGCAGAGAATTAGGAGCGACCGAGGGCTAGAGTATTGAATGGCGAGAGGTGAAATTTGATGACCCATTCAGGAGTGACAAAGGCGAAGGCACTAGTCAAGGGCGAATCCGATGATCAAGGACGTAGGCTAGAGTTTCGAAAACGATTAGAGACCGGAGTAGTTCTAGCAGTAAACTATGCCGACGCCGTGGTGT-GG--TATT----C-------TGTATTGCGGAAGAGAAATCAAGT-AAGGCTTTGGGGAGAGTACGCGCGCAAGCGATAAATT-------------------------------------------------------------------------------------------------------------------------------------------------------------------------------------------------------------------------------------------------------------------------------------------------------------------------------------------------------------------------------------------------------------------------------------------------------------------------------------------------------------------------------------------------------------------------------

>>JQ673481_GB_Echinogammarus_marinus_Dber_YAN-11_912pb_JQ673481

------------------------------CATGTGTAAGCGAAGCGTAACGTGGAGCGGTGAAAGGCTCAGTAACGGGCGAGTTATTTGTTCTCCTGGGACGGACAACACCGGGAAACTGGTGGGAAAACGTCTAAGTTGCGG------T----TTTCTAATCGCGGCGTAAA-CCA---TTGGTGCAGGAGAGTAAGCTGCCATCCTATCAGTTAGTAAGTAGGGTAAGGGCCTACTTAGACGAAGACGGGTACGGGGAATGAGGGTTTGATTCCGGAGAGGGAGCCTGAGAGACGGCTACCAGGACCAAGGTCAGCAGCAGGCGCGAAAATTATCGAAGCCCGCA-TAGGGGCGATAGTGAGGAGACGTGTATTACGAAGTGTGTGTAAAGAACGCACTAATAACTGGA-GGTCAAGTCTGGTGCCAGCATCCGCGGTAATTCCAGCTCCAGGGGTGTCTATGATGATTGCTGCGATTAAAAAGTCCGTAGTCAAGCTGACTG-ACTTGCCTGCAATG-TGACTGATTAAGAGACGAGCAGGGCTAGGAAAGCAGAGAATTAGGAGCGACCGAGGGCTAGAGTATTGAATGGCGAGAGGTGAAATTTGATGACCCATTCAGGAGTGACAAAGGCGAAGGCACTAGTCAAGGGCGAATCCGATGATCAAGGACGTAGGCTAGAGTTTCGAAAACGATTAGAGACCGGAGTAGTTCTAGCAGTAAACTATGCCGACGCCGTGGTATGGG--TTTT----C-------TGTATTGCGGAAGAGAAATCAAGT-AAGGCTTTGGGGAGAGTACGCGCGCAAGCGATAAATTTAAAGGAAATTGACGGAGGAACACCACAAGGAGTGGAGTGTGCGGCTTAATTTGACTCAACGCGGGACAGCTTACCAGGCCCGATAATCGTACGAGCGTAGTACGCGATAGGTTAGAGAGTGGTGCATGGCTGCTATCGACAGT-------------------------------------------------------------------------------------------------------------------------------------------------------------------------------------------------------------------------------------------------------------------------------------------------------------------------------------------------------------------------------------------------------------------------------

>>EF119216_BE_Dvil_Dber_WAT-07_325pb_EF119216

---------------------------------------------------------------------------------------------------GACGGACAACACCGGGAAACTGGTGGGAAAACGTCTAAGTTGCGG------T----TTTCTAATCGTGGCGTAAA-CCA--TCTGGTGCAGGAGAGTAAGCTGCCATCCTATCAGTTAGTAAGTAGGGTAAGGGCCTACTTAGACGAAGACGGGTACGGGGAATGAGGGTTTGATTCCGGAGAGGGAGCCTGAGAGACGGCTACCAGGACCAAGGTCAGCAGCAGGCGCGAAAATTATCGAAGCCCGCA-TAGGGGCGATAGTGAGGAGACGTGTATTACGAAGTGTGTGTAAAGAACGCACTAATAACTGGA-GGTCAAGTCTGGTGCCAGCAGCCGCG--------------------------------------------------------------------------------------------------------------------------------------------------------------------------------------------------------------------------------------------------------------------------------------------------------------------------------------------------------------------------------------------------------------------------------------------------------------------------------------------------------------------------------------------------------------------------------------------------------------------------------------------------------------------------------------------------------------------------------------------------------------------------------------------------------------------------------------------------------------------------------------------------------------------------------------------------------------------------------------

>>KF537632_US_Diporeia_sp._Ddip_WIN-14_1370pb_KF537632

GTGGACGCTAGTCTCACAGATTTAGCCATGCATGTGTAAGCGAAGCGTAACGTGGAGCGGTGAAAGGCTCAGTAACGGGCGATTTATTTGATCTCCTGGGATGGACAACACCGGGAAACTGGTGGGAAAACATCTAAGTTGCGG------TT---CTATTGATCGTGATGTAAA-CCTATGTGGGTGCAGGAGAGTAAGATGCCATCCTATCAGTTAGTAAGTAGGGTAAGGGCCTACTTAGACGAAGACGGGTACGGGGAATGAGGGTTTGATTCCGGAGAGGGAGCCTGAGAGATGGCTACCAGGACCAAGGTCAGCAGCAGGCGCGAAAATTATCGAAGCCCACCATTGGGGCGATAGTGAGGAGACGTGTATAACGAAATACGGGTAAAGAACGTATGTATAACTGGA-GGTCAAGTCTGGTGCCAGCATCCGCGGTAATTCCAGCTCCAGGGGTGTCTATGATGATTGCTGCGATTAAAAAGTCCGTAGTCAAGCTGACTG-ACTAACCTGTAATG-TGGTTGATTAAAAGACGAGAAGGGTTAGGAAAGCAGAGGATTAGGAGCGACCGAGGGCTAGAGTATTGAATGGCGAGAGGTGAAATTTGATGACCCATTCAGGAGTGACAAAGGCGAAGGCACTAGTCAAGGGCGAATCCGATGATCAAGGACGTAGGCTAGAGGTTCGAAAACGATTAGAGACCGGAGTAGTTCTAGCAGTAAACTATGCCGACGCCGTGGTAT-GT---TTT----TTAA----TGTATTGCGGAAGAGAAATCAAGT-AAGGCTTTGGGGAGAGTACGCGCGCAAGCGATAAATTTAAAGGAAATTGACGGAGGAACACCACAAGGAGTGGAGTGTGTGGCTTAATTTGACTCAACGCGGGACAGCTTACCAGGCCCGATAATCGTACGAGCGTAGTACGCGATAGGTTAAAGAGTGGTGCATGGCTGCTATCGACAGTTGGGGTGACC-TTAGGGTTAATTCCGGCAAGTAGTGAGACCCCTGCTATTAAATAGGACAGGTATTTTT-AAAATACAGGAAGGAAGGGACAAGAGCAGGTCGGTGATGCCCTTAGATGGCCTGGGCTGCACGCGCACTACAGTGGTCATTATAATGAGTAAATAGA-AGTAAAAATGATCGAGAGGGACTGGGCTTTGTAAGAGGCCCACGAACGAGGAATTGCTAGTAATCGTAGGCTCATTAAGATACGATGAATATGTCCCTGTACCTTGTACACACCGCCCGTCGTTATCGAAGATGGAATTGTGTGCGAACGAGCATTAAGCGAGTGAGCGCATAGTTCTAGATGTGATAAAAGTCGTAACAAGGCAACTGTAGGAGAACCTGTAGTTGGATCATAC------------

>>AJ438960_GB_Orchestia_cavimana_Dcav_TER-04_1326pb_Out_Group

------------------GATTTAGCCATGCATGTGTAAGCGAAGC-TATTGTGGAGCGGTGAAAGGCTCAGTAACGGGCGATTTATTTAGTCTTCTGGGACGGACAACACCGGGAAACTGGTGGGAAAACGTCTAAGCTGCGAATCACGC----TATGTGGTTGTGGCAGAAA-CTG---TTAGTGCAAAAAAGTAAGATGCCATCCTATCAGTTAGTAAGTAGGGTAAGGGCCTACTTAGACGAAGACGGGTACGGGGAATGAGGGTTTGATTCCGGAGAGGGAGCCTGAGAGACGGCTACCAGGACCAAGGTCAGCAGCAGGCGCGAAAATTATCGAAGCCCGCC-TAGGGGCGATAGTGAGGAGACGTGAAT-TTTAGGTGCGTGTAAAGAACGCACTAGCAACTGGA-GGTCAAGTCTGGTGCCAGCATCCGCGGTAATTCCAGCTCCAGGGGTGTCTATGATGATTGCTGCGATTAAAAAGTCCGTAGTCAAGCCGCCAG-ACCAGTCTGGAATGTTTCTTGATCAAGAGACGAGCAGGGCTGGGAAAGCGGAGAATTAGGAGCGACCGAGGGCTAGAGTATTGGGTGGCGAGAGGTGAAATTTGATGACCCATCCAGGAGTGACAAAGGCGAAGGCACTAGTCAAGGGCGAATCCGATGATCAAGGACGTAGGCTAGAGTTTCGAAAACGATTAGAGACCGGAGTAGTTCTAGCAGTAAACTATGCCGACGCCGTGATAT-GA---TTT----T-----GTTGTATTGCGGAAGAGAAATCAAGT-AAGGCTTTGGGGAGAGTACGCGCGCAAGCGATAAATTTAAAGGAAATTGACGGAGGAACACCACAAGGAGTGGAGTGTGCGGCTTAATTTGACTCAACGCGGGACAGCTTACCAGGCCCGATAATCGTACGAGCGTAGTACGCGATAGATTAGAGAGTGGTGCATGGCTGCTATCGACAGTTGGGGTGACC-TTAGGGTTAATTCCGGCAAGTAGTGAGACCCCCGCAG-TAT--TGGACAGGCGTCGTG-AAGATGCAGGAAGGAGGGGACAAGAGCAGGTCAGTGATGCCCTTAGATGGCCTGGGCTGCACGCGCACTACAGTGGTCATTATAAGGAGAAGTTAGA-AATAAAGATGATCGAGAGGGACTGGGCTTTGTAAGAGGCCCACGAACGAGGAATTGCTAGTAATCGCAGGCTCATTAGGATGCGATGAATATGTCCCTGTACCTTGTACACACCGCCCGTCGTTATCGAAGATGGAATTGTATGCGAACGAGCAGCAAGCGAGTGAGCGTATAGTTCTAGATGTGATAAAAGTCGTAACAAGGCAACTGTAGGAGAACCTGTAGTTGGATCACAC------------
