# Supplementary material for: Microsporidian infections in the species complex Gammarus roeselii (Amphipoda) over its geographical range: evidence for both host–parasite co-diversification and recent host shifts
Source: Parasit Vectors. 2019 Jun 28;12:327. doi: 10.1186/s13071-019-3571-z (PMC6599290; doi:10.1186/s13071-019-3571-z)
Supplement: Supplementary file 6 — Additional file 6: Data S3. Sequences under 200 bp for which no GenBank number can be attributed. [file 13071_2019_3571_MOESM6_ESM.docx]

Microsporidian infections in the Amphipoda species complex *Gammarus roeselii* over its geographic range: evidence for both host-parasite co-diversification and recent host-shifts.

Adrien Quiles, Karolina Bacela-Spychalska, Maria Teixeira, Nicolas Lambin, Michal Grabowski, Thierry Rigaud & Rémi A. Wattier

Additional file 6 - Data S3. Sequences under 200pb for which no GenBank number can be attributed. See Additional file 1, Table S1 for details

>C.orn._H18-03

CATGTGTAAGCGAACAATTAGGGAGCTGCGGACTGCTCAGTAACAGGCGATTAATTTAATCTTTACAAACGGACAAACTCAGGAAACGGAGTGTAATACGTAAAAGATGATTTTTTATTTAAAAAAGAAAACATTTTTAGCTTGAATAAAGCGGTAAAGAATAAGACGCCAACCCATCAG

>C.orn._COL-338R

GCATGTGTAAGCGAACAATTAGGGAGCTGCGGACTGCTCAGTAACAGGCGATTAATTTAATCTTTACAAACGGACAAACTCAGGAAACGGAGTGTAATACGTAAAAGATGATTTTTTATTTAAAAAAGAAAACATTTTTAGCTTGAATAAAGCGGTAAAGAATAAGACGCCAACCCATCAG

>C.orn._PL4-06

GCATGTGTAAGCGAACAATTAGGGAGCTGCGGACTGCTCAGTAACAGGCGATTAATTTAATCTTTACAAACGGACAAACTCAGGAAACGGAGTGTAATACGTAAAAGATGATTTTTTATTTAAAAAAGAAAACATTTTTAGCTTGAATAAAGCGGTAAAGAATAAGACGCCAACCCATCAG

>C.orn._BG01-02

GCATGTGTAAGCGAACAATTAGGGAGCTGCGGACTGCTCAGTAACAGGCGATTAATTTAATCTTTACAAACGGACAAACTCAGGAAACGGAGTGTAATACGTAAAAGATGATTTTTTATTTAAAAAAGAAAACATTTTTAGCTTGAATAAAGCGGTAAAGAATAAGACGCCAACCCATCAG

>C.orn._BG01-05

GCATGTGTAAGCGAACAATTAGGGAGCTGCGGACTGCTCAGTAACAGGCGATTAATTTAATCTTTACAAACGGACAAACTCAGGAAACGGAGTGTAATACGTAAAAGATGATTTTTTATTTAAAAAAGAAAACATTTTTAGCTTGAATAAAGCGGTAAAGAATAAGACGCCAACCCATCAG

>C.orn._BG01-09

GCATGTGTAAGCGAACAATTAGGGAGCTGCGGACTGCTCAGTAACAGGCGATTAATTTAATCTTTACAAACGGACAAACTCAGGAAACGGAGTGTAATACGTAAAAGATGATTTTTTATTTAAAAAAGAAAACATTTTTAGCTTGAATAAAGCGGTAAAGAATAAGACGCCAACCCATCAG

>C.orn._BG01-10

GCATGTGTAAGCGAACAATTAGGGAGCTGCGGACTGCTCAGTAACAGGCGATTAATTTAATCTTTACAAACGGACAAACTCAGGAAACGGAGTGTAATACGTAAAAGATGATTTTTTATTTAAAAAAGAAAACATTTTTAGCTTGAATAAAGCGGTAAAGAATAAGACGCCAACCCATCAG

>C.orn._H18-02

GCATGTGTAAGCGAACAATTAGGGAGCTGCGGACTGCTCAGTAACAGGCGATTAATTTAATCTTTACAAACGGACAAACTCAGGAAACGGAGTGTAATACGTAAAAGATGATTTTTTATTTAAAAAAGAAAACATTTTTAGCTTGAATAAAGCGGTAAAGAATAAGACGCCAACCCATCAG

>C.orn._D10-13

TGCATGTGTAAGCGAACAATTAGGGAGCTGCGGACTGCTCAGTAACAGGCGATTAATTTAATCTTTACAAACGGACAAACTCAGGAAACGGAGTGTAATACGTAAAAGATGATTTTTTATTTAAAAAAGAAAACATTTTTAGCTTGAATAAAGCGGTAAAGAATAAGACGCCAACCCATCAG

>C.orn._H18-11

TGCATGTGTAAGCGAACAATTAGGGAGCTGCGGACTGCTCAGTAACAGGCGATTAATTTAATCTTTACAAACGGACAAACTCAGGAAACGGAGTGTAATACGTAAAAGATGATTTTTTATTTAAAAAAGAAAACATTTTTAGCTTGAATAAAGCGGTAAAGAATAAGACGCCAACCCATCAG
