# Supplementary figures and images for: Microsporidian infections in the species complex Gammarus roeselii (Amphipoda) over its geographical range: evidence for both host–parasite co-diversification and recent host shifts
Source: Parasit Vectors. 2019 Jun 28;12:327. doi: 10.1186/s13071-019-3571-z (PMC6599290; doi:10.1186/s13071-019-3571-z)

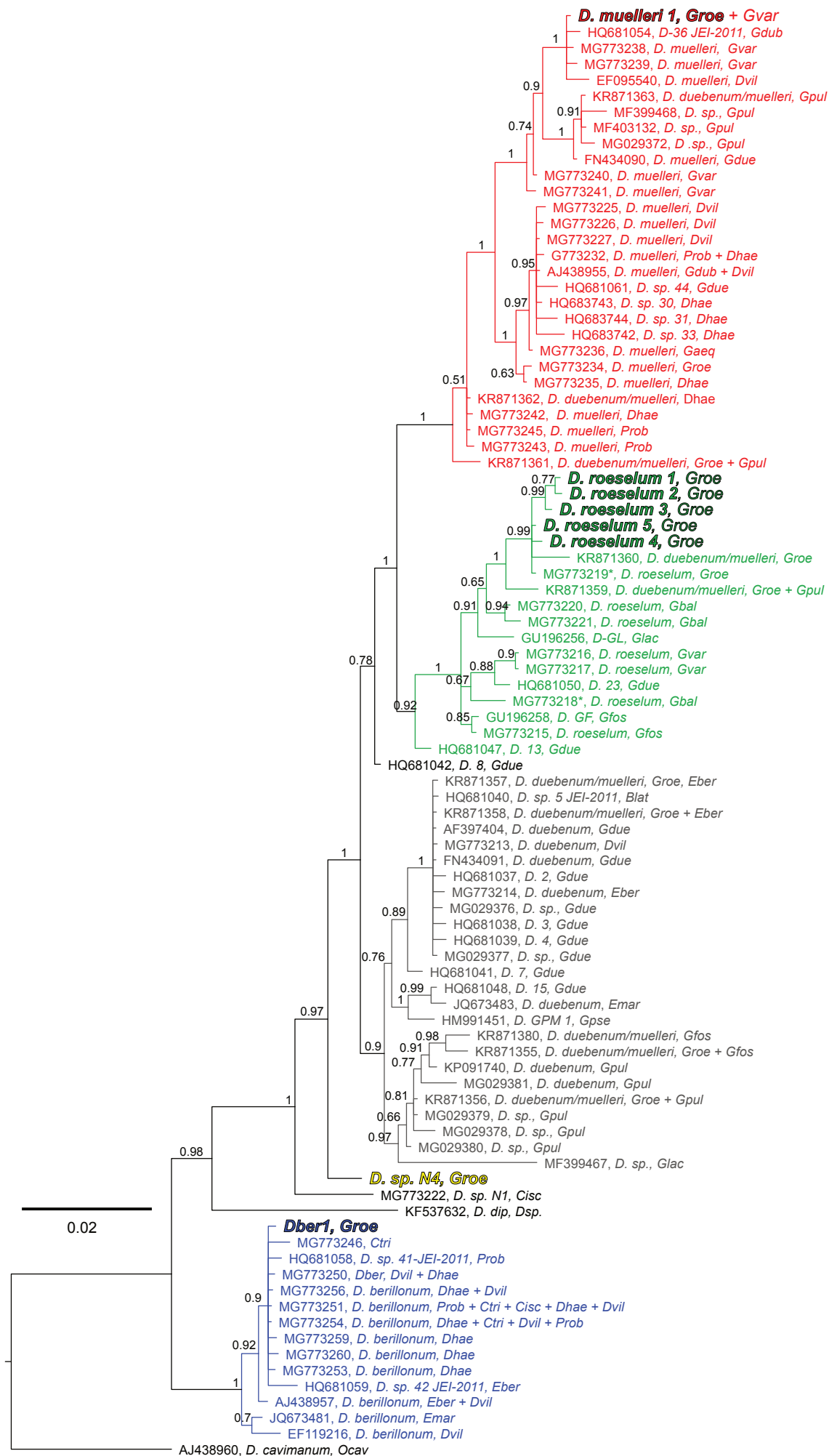

Supplement: Supplementary file 7 — Additional file 7: Figure S1. Phylogenetic tree for infections by microsporidians of the genus Dictyocoela in European freshwater amphipods, including all haplogroups. Bayesian phylogenetic reconstruction based on small ribosomal subunit rDNA alignment of Dictyocoela spp. Dictyocoela cavimanum was used as outgroup. Divergent lineages were ascribed to the same color code as Fig. 5, and follow recent reassessment of the genus taxonomy by Bacela-Spychalska et al. [24]. Sequences from the present study are in bold and labels include haplogroup names, and the hosts found infected by the haplogroup. Sequences from GenBank are all other Dictyocoela haplogroups (Additional file 4: Table S3). Labels include, in this order, the accession number, the microsporidia species name given in the associated publication and the species abbreviated name(s). For abbreviations of host species names: see Additional file 4: Table S3. Numbers on the branches indicates Bayesian posterior probability. [file 13071_2019_3571_MOESM7_ESM.pdf]
